# Proteomic Changes in Rat Spermatogenesis in Response to *In Vivo* Androgen Manipulation; Impact on Meiotic Cells

**DOI:** 10.1371/journal.pone.0041718

**Published:** 2012-07-30

**Authors:** Peter G. Stanton, Pavel Sluka, Caroline F H. Foo, Andrew N. Stephens, A. Ian Smith, Robert I. McLachlan, Liza O’Donnell

**Affiliations:** 1 Prince Henry’s Institute, Monash Medical Centre, Clayton, Victoria, Australia; 2 Department of Biochemistry and Molecular Biology, Monash University, Clayton, Victoria, Australia; 3 Department of Obstetrics and Gynaecology, Monash University, Clayton, Victoria, Australia; 4 Department of Anatomy and Developmental Biology, Monash University, Clayton, Victoria, Australia; Baylor College of Medicine, United States of America

## Abstract

The production of mature sperm is reliant on androgen action within the testis, and it is well established that androgens act on receptors within the somatic Sertoli cells to stimulate male germ cell development. Mice lacking Sertoli cell androgen receptors (AR) show late meiotic germ cell arrest, suggesting Sertoli cells transduce the androgenic stimulus co-ordinating this essential step in spermatogenesis. This study aimed to identify germ cell proteins responsive to changes in testicular androgen levels and thereby elucidate mechanisms by which androgens regulate meiosis. Testicular androgen levels were suppressed for 9 weeks using testosterone and estradiol-filled silastic implants, followed by a short period of either further androgen suppression (via an AR antagonist) or the restoration of intratesticular testosterone levels. Comparative proteomics were performed on protein extracts from enriched meiotic cell preparations from adult rats undergoing androgen deprivation and replacement *in vivo.* Loss of androgenic stimulus caused changes in proteins with known roles in meiosis (including Nasp and Hsp70–2), apoptosis (including Diablo), cell signalling (including 14-3-3 isoforms), oxidative stress, DNA repair, and RNA processing. Immunostaining for oxidised DNA adducts confirmed spermatocytes undergo oxidative stress-induced DNA damage during androgen suppression. An increase in PCNA and an associated ubiquitin-conjugating enzyme (Ubc13) suggested a role for PCNA-mediated regulation of DNA repair pathways in spermatocytes. Changes in cytoplasmic SUMO1 localisation in spermatocytes were paralleled by changes in the levels of free SUMO1 and of a subunit of its activating complex, suggesting sumoylation in spermatocytes is modified by androgen action on Sertoli cells. We conclude that Sertoli cells, in response to androgens, modulate protein translation and post-translational events in spermatocytes that impact on their metabolism, survival, and completion of meiosis.

## Introduction

The production of sperm, known as spermatogenesis, requires androgen action. Spermatogenesis takes place in the seminiferous tubules, where the somatic Sertoli cells co-ordinate the development of germ cells though the various phases of development. The most immature germ cells, the diploid spermatogonia, proliferate prior to their entry into meiosis. Spermatocytes then proceed through meiosis where genetic information is exchanged via homologous chromosome recombination, and the final meiotic divisions produce haploid spermatids. Spermatids then undergo complex remodelling during spermiogenesis to produce the mature streamlined spermatid form. Sertoli cells provide structural and nutritional support to the developing germ cells by establishing a unique microenvironment within the tubules; this includes regulation of paracrine factors and cell-surface protein expression, reviewed in [1].

Quantitatively normal sperm production requires the action of both androgens and follicle-stimulating hormone (FSH), reviewed in [2]–[4]; FSH is particularly important for establishing a normal, functional Sertoli cell population, whereas androgen action is needed for the completion of germ cell development. Androgens can influence testis function via effects on Leydig cells, peritubular myoid cells and Sertoli cells, but are not considered to act directly on germ cells, reviewed in [5]. Transgenic mouse models show that the stimulation of spermatogenesis by androgen requires a direct action on androgen receptors (AR) in Sertoli cells [6], [7]. AR action in other testicular somatic cells, including peritubular myoid cells [8] and Leydig cells [9] is also essential for normal spermatogenesis, indicating that germ cell development depends on androgen signalling via a number of different somatic cell types [5], [10]. It is well established that germ cell development is highly sensitive to small changes in testicular androgens, and that there are different “thresholds” for androgen action [11]. For example, low levels of testicular androgens that support the completion of meiosis cannot support the completion of spermiogenesis [12], a finding supported by observations in mice expressing a hypomorphic AR in Sertoli cells [13]. A better understanding of the molecular mechanisms by which androgens regulate spermatogenesis is needed, as current approaches to hormonal male contraception in part rely upon full suppression of intratesticular androgen action.

The completion of meiosis is well known to require androgen action transduced via Sertoli cell AR, reviewed in [5] and to be uniquely sensitive to testicular androgen levels [12]. Meiosis begins when spermatogonia divide into preleptotene spermatocytes which replicate their DNA during S phase, reviewed in [14]. Prophase I proceeds with the initiation of double strand breaks in DNA followed by homologous chromosome pairing in leptotene and zygotene spermatocytes, respectively, and then follows a long period (more than 2 weeks in the rat) of chromosomal crossover in pachytene spermatocytes, which ensures genetic diversity of the gametes. Desynapsis of chromosomes occurs during the diplotene phase, and thereafter the first meiotic division (Meiosis I) proceeds rapidly to produce secondary spermatocytes. These spermatocytes then quickly (∼15 hr) enter the second meiotic division (Meiosis II) to produce haploid round spermatids.

Pachytene spermatocytes initiate the transcription of genes involved in the completion of meiosis in the mid-spermatogenic stages VII and VIII [15], [16], when Sertoli cell AR expression is highest, reviewed in [2], [3]. Androgen action on the Sertoli cell is well known to influence the survival of pachytene spermatocytes. A small proportion of the pachytene spermatocyte population is lost via apoptosis, reviewed in [17], in stages VII and VIII during suppression of gonadotropins and/or intratesticular androgen [18]–[20]. Mice lacking AR in Sertoli cells (SCARKO mice) show a progressive loss of pachytene spermatocytes between stages VI-XII, a reduced number of spermatocytes entering the diplotene phase [6], and few post-meiotic cells (<0.5% of wildtype) are produced [21], suggesting that if spermatocytes survive the pachytene phase, they are largely unable to undergo normal meiotic division.

Taken together, the above studies suggest that Sertoli cells, upon androgen stimulation, secrete factors and/or express cell surface proteins that influence pachytene spermatocyte survival and ability to complete meiosis. Progress has been made towards understanding how androgens act on their receptors in Sertoli cells, reviewed in [3], 22 and identifying genes that are modulated in these cells in response to AR modulation, such as Rhox5 [23] and genes involved in retinoic acid metabolism and cellular adhesion, reviewed in [24]. It is clear that androgens support numerous Sertoli cell processes that are essential for germ cell development, including the function of the blood testis barrier and the development of a functionally mature Sertoli cell phenotype necessary to support germ cells [25], [26]. However the mechanism by which the Sertoli cells “transduce” the androgenic stimulus to germ cells, and what processes in germ cells respond to this stimulus, is unclear. Previous studies have shown that germ cells exhibit complex cell- and stage-specific transcriptional changes in response to androgen and FSH suppression [16], however the effects on proteins in these cells is unknown. We hypothesised that androgens act on AR expressing cells in the testis, particularly Sertoli cells, which in turn modulate pachytene spermatocyte gene transcription, translation and post-translational protein modification to ensure spermatocyte survival and completion of meiosis. This study aimed to investigate the proteomic changes occurring in meiotic cells during testicular androgen suppression and replacement in vivo, in order to better understand the response of meiotic cells to somatic cell-mediated androgen action. The results demonstrate that meiotic spermatocytes respond to changes in testicular androgens, indirectly, by modulating the expression and post-translational modification of proteins involved in oxidative metabolism, DNA repair, RNA processing, apoptosis and meiotic division.

## Results and Discussion

### Rationale for Androgen Suppression and Replacement Treatments

**Figure 1:**
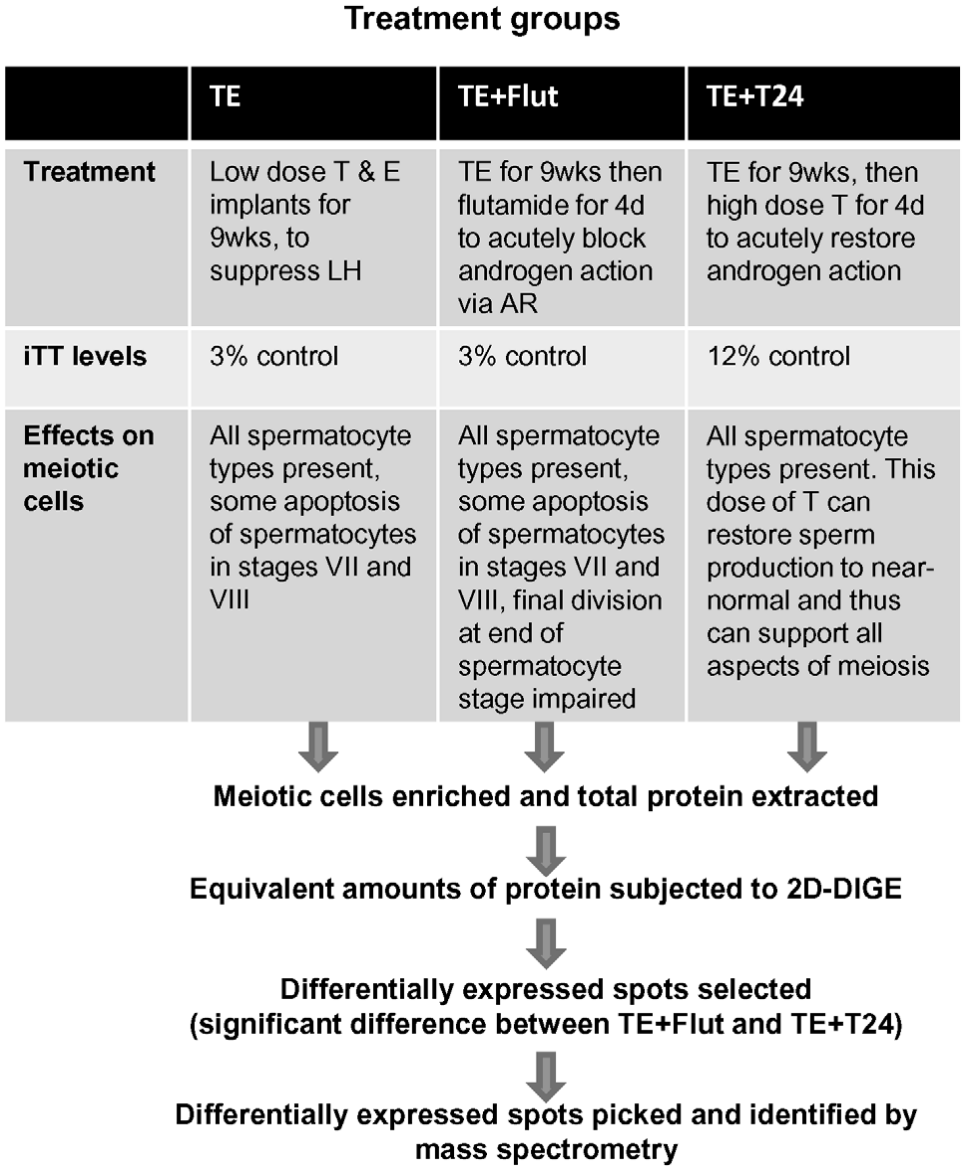
Schematic diagram of study design and rationale. Three treatment groups (TE, TE+Flut, TE+T24) were utilised in this study, as well as an untreated control group. Each group consisted of 4–5 adult rats. These treatments have been used previously, and their effects on intratesticular testosterone levels (iTT) and spermatogenic cell populations have been described, see *Results*. At completion of treatment, enriched meiotic cell preparations were prepared from each animal and total protein was isolated. Equal amounts of protein from each rat was subjected to 2-dimensional Difference In-Gel Electrophoresis (2D-DIGE) analysis. Protein spots were considered to be differentially expressed in response to in vivo androgen manipulation if they showed a statistical (p<0.05) difference between the TE+Flut and TE+T24 groups. For full details, see *Materials and Methods.*

For an overview of the experimental design and study rationale, see Figure 1. Testicular androgen suppression was achieved by 9 weeks of testosterone and estradiol (TE) treatment [27], [28]. We have previously shown that this treatment suppresses gonadotropin secretion and lowers intratesticular testosterone (iTT) levels to ∼3% of control [27], [28]. TE treatment decreased testis weights as expected to 36% of control [control; 1.92±0.16 g,TE; 0.63±0.12 g, (mean ± SD, n = 4, (P<0.001)]. We have previously shown that TE treatment impairs the survival of pachytene spermatocytes in stages VII and VIII leading to a small reduction in the number of pachytene spermatocytes in the later stages (IX-XIV) of spermatogenesis [18], [19]. However, with this treatment, the conversion of spermatocytes into haploid round spermatids (a measure of meiotic division) is not significantly altered [19]. Thus TE treatment is expected to induce some degree of pachytene spermatocyte apoptosis, but the final meiotic division is preserved. More complete androgen suppression was achieved by the addition of the AR antagonist, flutamide, during the final 4 days of TE treatment (TE+Flut) [19], [29]. This treatment blocks the residual androgen action in the TE-treated testis and as expected reduced testis weight to 26% of control (TE+Flut, 0.50±0.05 g, n = 4, NS compared to TE). We have previously shown that the addition of flutamide does not change testicular testosterone levels [19]. This treatment causes more profound effects on pachytene survival [19], [29], and also significantly impairs the final meiotic division at the end of the spermatocyte phase, as assessed by the conversion of pachytene spermatocytes in stages IX-XIV to round spermatids [29]. The restoration of testicular androgen action in TE animals was achieved by the administration of high dose testosterone (T24 cm) (TE+T24) for 4 days. We have previously shown that this dose of testosterone, when given to TE-treated animals, significantly restores testicular testosterone levels (to ∼12% of control) as well as late pachytene spermatocyte and round spermatid populations [27], [30] and, subsequently, sperm production to near-normal levels [31]. This treatment thus restores pachytene spermatocyte survival and allows the successful completion of meiotic division.

Enriched meiotic cell preparations were prepared from control (untreated rats), androgen-suppressed rats (from TE alone and TE+Flutamide groups) and androgen-replaced rats (TE+T24). Cell were enriched from whole testis lysates and subjected to elutriation, which separates cells on the basis of size. Because all stages of spermatocytes are represented in each treatment group [18], [19], [27], [28] and whole testis lysates were used, we expect minimal differences in the types of spermatocytes between each preparation. After protein extraction, equivalent protein concentrations from each group were loaded onto the gels in order to correct for cell number.

### Overview of Proteomic Changes in Meiotic Cells in Response to in vivo Androgen Manipulation

**Figure 2:**
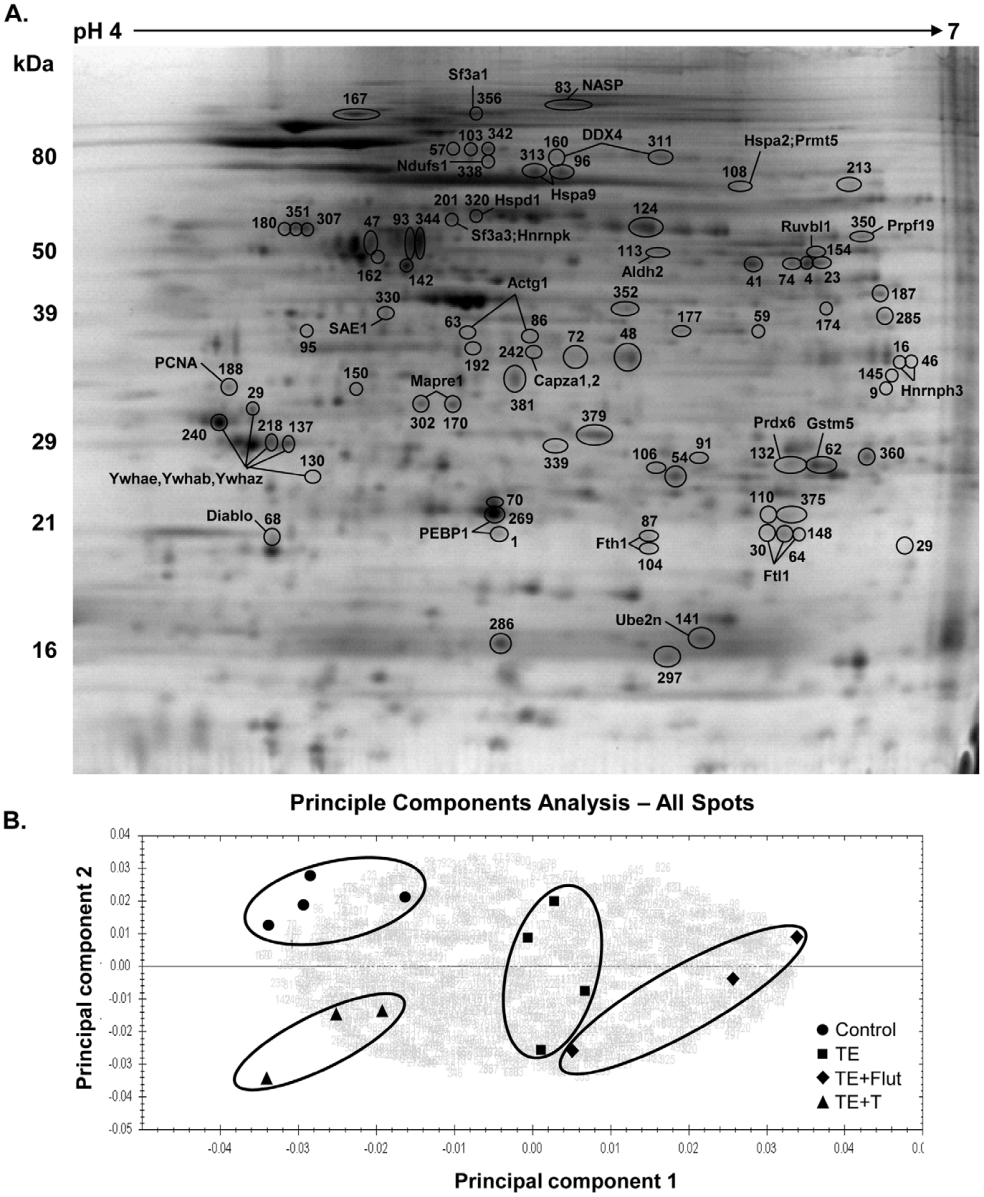
Proteomic analysis of enriched meiotic cell preparations. **A.** Representative 2D-DIGE image of rat spermatocyte proteins (first dimension pH 4–7, second dimension 8–16% polyacrylamide gradient). The image shown is the Cy2-labelled internal standard which was added to all gels, and was prepared by mixing equal amounts of all samples (see *Materials and Methods* for details). Proteins shown to be significantly different (p<0.05) between groups by image analysis and subsequently identified by mass spectrometry are indicated (see Table 1 for details of spot identity). **B.** Principle component analysis of the expression patterns of all 738 spots identified four separate clusters corresponding to the four treatment groups.

Comparative proteomics were performed on enriched meiotic cell preparations from each of the four treatment groups (see Materials and Methods). Image analysis of the 7 2D-DIGE gels revealed a total of 738 protein spots present on all gels (Figure 2A) which could be evaluated statistically. Unsupervised statistical examination of the expression patterns of all 738 spots using principle component analysis (PCA, Figure 2B) showed four distinct clusters of spots which corresponded to the four treatment groups, thus providing an independent measure that each treatment group contained differentially expressed proteins. It is noteworthy that groups with high androgen (control, TE+T24) were well separated from low androgen groups (TE, TE+Flut) in the PCA (Figure 2B). In order to identify proteins that were significantly changed during androgen suppression and replacement, we analysed significant differences between spots in the high androgen (TE+T24) versus low androgen (TE+Flut) groups. A comparison with controls, which also have high testicular testosterone levels, was not used in this instance, to avoid potential confounding effects due to the presence of more mature spermatid populations in the testis compared to the TE+T24 group.

Using the above approach, 263 spots were shown to be statistically different between the highest and lowest androgen groups with false discovery values (q) <0.03 and power values >0.75; both parameters indicative of high quality data (Table S1 and S2). Differences in fold changes in expression varied from 0.56 (spot#57) to 2.56 fold (spot#1, Table S2). Of the 263 spots, 116 were able to be analysed by mass spectrometry, and 82 spots were identified, with average sequence coverages of 33–40% for peptide mass fingerprinting and MS/MS analyses, and 15% for LC-MS/MS analyses (Tables S2 and S3). Some spots mapped to multiple IDs indicative of i) protein isoforms that were not distinguishable based on the identified peptide sequences (see for example spot 47 containing various tubulin beta chains), or ii) multiple proteins of similar isoelectric point (pI) and molecular weight found within a single spot (see for example spot 9 in Table S2). For these examples, verification of androgen-dependency of individual proteins would require additional experimentation, for example by western blot (see below). The total number of proteins with unique accession numbers identified was 88 (Table S1, S2). For some proteins, various isoforms of the same gene/protein were identified (for example see *Immt* in Table 1).

**Table 1:** Known and predicted functions of androgen-responsive proteins in enriched meiotic cell preparations†.

Gene	Protein name (alias)	SpotNo.	Normalised spot volume				Known functions a	Role in meioticcells? b
			C	TE	TE+Flut	TE+T24		
*Acrbp*	Acrosin-binding protein	95	0.99	0.69	0.66	1.04	May be involved in packaging and condensation of the acrosin zymogen in the acrosome	
*Actg1*	Actin, cytoplasmic 2 (Gamma-actin)	63b, 86	1.34	1.08	0.97	1.72	Role in cytoskeleton, scaffolding, cell movement, cell cycle	Meiotic division, cell structure
*Actr1a*	Alpha-centractin(Arp1, Ctrn1)	187	1.09	0.90	0.77	1.06	Key component of the dynactin complex involved in microtubule-based motility. Associated with centrosome and likely involved in chromosome organisation and spindle dynamics	Meiotic division
*Akr1b1*	Aldose reductase (Alr)	9a, 145	1.57	0.81	0.68	1.05	Oxidoreductase, activates stress signalling pathways	Response to oxidative stress
*Aldh2*	Aldehyde dehydrogenase, mitochondrial precursor	113a	1.13	0.91	0.73	0.91	Metabolic enzyme present in mitochondrial matrix, protector of oxidative stress	Response to oxidative stress
*Atp5a1*	ATP synthase subunit alpha, mitochondrial precursor (Chain A, Rat Liver F1-Atpase)	113b	1.13	0.91	0.73	0.91	Component of ATPase machinery in mitochondria	Response to oxidative stress
*Atp5b*	ATP synthase subunit beta, mitochondrial precursor, OR ATP synthase beta subunit c	162a	1.08	0.74	0.90	0.97	A component of ATPase machinery in mitochondria	Response to oxidative stress
*Capza1*	F-actin capping protein alpha-1 subunit (CapZa1)	242b	1.23	1.10	0.92	1.19	Subunit of Capz F-actin capping protein which regulates actin filament assembly and disassembly	Meiotic division, cell structure
*Capza2*	F-actin-capping protein subunit alpha-2 (CapZa2)	242a	1.23	1.10	0.92	1.19	Subunit of Capz F-actin capping protein which regulates actin filament assembly and disassembly	Meiotic division, cell structure
*Cops4/Csn4*	COP9 signalosome complex subunit 4	352	1.00	1.08	1.06	0.88	Component of COP9 signalosome complex (CSN), involved in various cellular and developmental processes. CSN is an essential regulator of ubiquitin conjugation pathway and has known roles in cell cycle and in meiosis. Other COP subunits are highly expressed in PSC	Meiosis
*Crym*	Mu-chrystallin homolog	192	0.86	0.96	1.00	1.22	Binds thyroid hormone and NADPH. Multiple alternatively spliced transcript variants have been found for this gene	
*Ddx4*	Probable ATP-dependent RNA helicase DDX4 (Mvh)	160b, 311	0.89	0.95	1.14	0.78	Germ cell specific RNA helicase DEAD box proteins, null mutation in mice causes arrest at zygotene spermatocytes. Involved in miRNA processing and germ cell specification in chromatoid body. Localises to site of microtubule assembly	Meiosis, RNA processing
*Diablo*	Diablo homolog (Drosophila) (larger C-terminal fragment) (Smac/Diablo)	68	1.76	1.43	1.08	1.88	Mitochondrial protein involved in apoptosis; upon apoptotic stimulus binds to inhibitors of apoptosis and prevents their interaction with caspases	Apoptosis
*Eno1*	Alpha-enolase (Eno1)	4, 23, 41, 74	1.49	0.95	0.59	0.83	Subunit of a multifunctional enzyme with roles in glycolysis, hypoxia tolerance, allergic responses	Indicator of metabolic stress
*Fabp9*	Fatty acid binding protein 9, testis	297	0.81	0.99	1.04	0.93	Testis specific fatty acid binding protein, is increased during germ cell apoptosis [90]	Apoptosis
*Fth1*	Ferritin heavy chain (Fth1)	87, 104	0.85	1.37	1.26	0.97	Component of ferritin complex, involved in iron storage and delivery	Response to oxidative stress
*Ftl1*	Ferritin light chain 1 (Ftl1)	30, 64, 148	0.59	0.93	1.21	1.05	Component of ferritin complex, involved in iron storage and delivery	Response to oxidative stress
*Galk1*	Galactokinase 1	63c	1.34	1.08	0.97	1.72	Galactose/carbohdryate metabolism	Cell metabolism
*Gstm5*	Glutathione S-transferase Mu 5	62	1.49	1.05	0.98	0.84	Subunit of glutathione S-transferase (GST) enzymes. This subunit likely functionally distinct from other mu class subunits, is primarily expressed in testis. GSTs have antioxidant properties and are involved in oxidative stress response	Response to oxidative stress
*Hnrnpk*	Heterogeneous nuclear ribo-nucleoprotein K (hnRNP K)	201b	0.83	1.05	1.16	0.93	Multifunctional protein with roles in splicing, transcription, translation, chromatin remodeling and apoptosis	Apoptosis, RNA splicing
*Hnrph3*	Heterogeneous nuclear ribo-nucleoprotein H3 (HnRNP H) *OR* mCG11326, isoform CRA_b [Mus musculus]	16b, 46a, 46b	1.25	0.74	0.56	1.05	Heterogeneous nuclear ribonucleoprotein, role in RNA splicing, component of spliceosome	RNA splicing
*Hspa2*	Heat shock-related 70 kDa protein 2 (Hsp70-2)	108b	1.14	0.96	1.06	0.74	Molecular chaperone with known role in meiosis: present in the synaptonemal complex and is necessary for activation of CDC2 and entry into meiotic division. Null mice are infertile due to pachytene spermatocyte arrest and apoptosis [91]	Meiotic division
*Hspa9*	Stress-70 protein, mitochondrial precursor (Grp75, mortalin)	96, 313	1.35	1.07	0.85	1.20	Multifunctional chaperone and mitochondrial protein involved in cell proliferation, stress response and cellular aging	Response to cell stress, meiosis
*Hspd1*	Heat shock protein 1 (chaperonin, Hsp60)	320	0.81	0.96	1.02	0.94	Mitochondrial protein import and protein folding. Known roles in apoptosis and response to cellular stress	Apoptosis, response to cell stress
*Iah1*	Isoamyl acetate-hydrolyzing esterase 1 homolog	379b	1.18	1.20	1.13	1.34	Probable lipase	
*Idi1*	Isopentenyl-diphosphate Delta-isomerase 1	106	1.42	1.08	0.91	1.33	Ubiquitously expressed isomerase	
*Immt*	Inner membrane protein, mitochondrial (Mitofilin)	342	0.94	1.08	1.16	0.98	Mitochondrial protein involved in maintenance of mitochondrial morphology, shown to be changed during apoptosis, oxidative stress & various disease states	Response to oxidative stress, apoptosis
*Immt*	Inner membrane protein, mitochondrial, isoform CRA_a (Mitofilin)	57, 103	0.76	1.03	1.23	0.68	Mitochondrial protein involved in maintenance of mitochondrial morphology, shown to be changed during apoptosis, oxidative stress & various disease states	Response to oxidative stress, apoptosis
*Ipo5*	predicted: Importin 5 (Putative uncharacterized protein Ipo5) (Imp5, Ranbp5)	167	1.00	1.14	1.26	0.87	Functions in nuclear protein import as nuclear transport receptor	
*Itpa*	Inosine triphosphatase [Rattus norvegicus]	70b, 269c	1.72	1.43	1.20	1.00	Hydrolyzes inosine and deoxyinosine triphosphate	
*Lap3*	Leucine aminopeptidase 3 (Cytosol aminopeptidase)	350a	1.10	0.92	0.90	0.95	Catalyzes removal of unsubstituted N-terminal amino acids from various peptides, presumably involved in processing and turnover of intracellular proteins	
*Ldhb*	L-lactate dehydrogenase B (Ldh-B)	48, 72	1.74	1.28	1.06	0.95	Cytoplasmic oxidoreductase involved in glycolysis	Cell metabolism, response to oxidative stress
*Ldhc*	L-lactate dehydrogenase C chain (Ldh3, LDH testis subunit)	9c	1.57	0.81	0.68	1.05	Cytoplasmic oxidoreductase involved in glycolysis, sperm motility	Cell metabolism
*Lztfl1*	Leucine zipper transcription factor-like protein 1	63a	1.34	1.08	0.97	1.72	Tumour suppressive activity, role in ciliary protein trafficking	
*Mapre1*	Microtubule-associated protein RP/EB family member 1 (EB1)	170b, 302	1.38	1.10	0.96	1.38	Role in microtubule polymerization and spindle function during cell division. Associates with centrosomes, Anaphase Promoting Complex and dynactin complex	Meiotic division
*Nasp*	Nuclear autoantigenic sperm protein (Nasp)	83	0.74	0.98	1.21	0.76	Linker histone chaperone required for DNA replication, normal cell cycle progression and cell proliferation. Interacts with Hspa2 in spermatocytes, involved in meiotic checkpoint	Meiotic division
*Ndufs1*	NADH dehydrogenase (ubiquinone) Fe-S protein 1, 75 kDa	338	1.21	0.97	0.99	1.19	Core subunit of the mitochondrial membrane respiratory chain NADH dehydrogenase (Complex I). Roles in oxidative metabolism. Cleaved by caspases during initial apoptotic response	Response to oxidative stress, apoptosis
*P4hb/* *Pdia1/*	Protein disulfide-isomerase precursor	180,307, 351	0.92	0.91	0.90	1.14	Multifunctional protein catalyzes the formation, breakage and rearrangement of disulfide bonds. Involved in ER stress response	Response to cell stress
*Pafah-1b2*	Platelet-activating factor acetylhydrolase 1B subunit beta (Pafahb)	379a	1.18	1.20	1.13	1.34	Subunit of the enzyme that metabolizes Platelet Activating Factor (PAF), a biologically active phospholipid with diverse biologic effects. Mice with a null mutation infertile due pachytene arrest [92]	Meiosis, apoptosis
*Park7* */Cap1*	Protein DJ-1, Contraception-associated protein 1 (DJ1, SP22, Cap1)	54, 110, 375b	1.53	1.13	0.85	0.96	Multifunctional molecular chaperone with roles in oxidative stress, apoptosis, fertilization, androgen-receptor dependent transcription, cancer	Apoptosis, response to oxidative stress
*Pcna*	Proliferating cell nuclear antigen (PCNA)	188	0.93	1.22	1.23	1.32	Auxiliary protein of DNA polymerase delta involved in DNA replication and post-replicative DNA repair pathways. Mice expressing PCNA that cannot be mono-ubiquitylated are infertile due to spermatocyte arrest [81]	DNA repair
*Pdhb*	Pyruvate dehydrogenase E1 component subunit beta, mitochondrial precursor	381	1.22	1.12	1.04	1.10	Component of pyruvate dehydrogenase, located in the mitochondrial matrix. Role in oxidation reduction.	Cell metabolism, response to oxidative stress
*Pdia3*	Protein disulfide-isomerase A3 precursor	124	1.18	0.79	0.81	1.21	Isomerase with roles in cell (and ER) stress and apoptosis	Apoptosis, response to cell stress
*Pdia6*	Protein disulfide-isomerase A6 precursor (CaBP1)^c^	142a	1.23	1.10	0.93	1.38	Isomerase present in the ER and on cell surface. Identified in various proteomic screens of cells under stress	Response to cell stress
*Pebp1*	Phosphatidyl-ethanolamine-binding protein 1 (Pebp1/Rkip)	1, 269a	1.85	0.62	0.64	1.64	Inhibits signalling pathways (MAPK, NFKB and GCPR), various roles including apoptosis and cell cycle. Component of sperm plasma membrane	Apoptosis, cell cycle
*Pgls*	6- phospho-gluconolactonase (6PGL)	339	1.09	0.98	0.88	0.99	Enzyme involved in carbohydrate metabolism	Cell metabolism
*Ppt1*	Palmitoyl-protein thioesterase 1 (Ppt1)	9b	1.57	0.81	0.68	1.05	Lysosomal protein, involved in apoptosis, cell stress	Apoptosis, response to cell stress
*Prdx6*	Peroxiredoxin-6	132	1.39	0.95	1.03	0.93	Cytosolic member of peroxiredoxin family of antioxidant proteins, protects cells from oxidative damage	Response to oxidative stress
*Prmt5*	Protein arginine N-methyl-transferase 5 (Prmt5)	108a	1.14	0.96	1.06	0.74	Methyltransferase that dimethylates arginine residues in proteins including components of the spliceosome and Piwi proteins in meiotic nuage and chromatoid body. Null mutation of *Drosophila* homolog causes spermatocyte arrest [66]	RNA splicing, meiosis
*Prpf19*	Pre-mRNA-processing factor 19 (Nuclear matrix protein SNEV [Mus musculus]) (Prp19, SNEV)	350b	1.10	0.92	0.90	0.95	Roles in DNA double-strand break repair, spliceosome assembly, localized to mitotic spindle during anaphase, participates in protein degradation in the proteosome	DNA repair, RNA splicing, meiotic division
*Psma6*	Proteasome subunit alpha type 6	360	1.12	1.02	0.93	0.95	Component of the proteosome	Protein degradation
*Psmb4*	Proteasome subunit beta type 4 precursor (Psmb4)	91	1.44	1.11	0.98	0.91	Beta7 subunit of 20 S proteasome, binds to other proteins including Prp19, role in targeted protein degradation	Meiosis, cell signalling, protein degradation
*Rab2a*	Ras-related protein Rab-2A (Rab2)	375a	1.03	1.19	1.14	1.22	Required for protein transport from the endoplasmic reticulum to the Golgi complex. Component of acrosome	
*Rab6a*	Ras-related protein Rab-6A [Mus musculus] (Rab6)	70a, 269a	1.72	1.20	1.00	1.43	Regulator of membrane traffic from the Golgi apparatus towards the endoplasmic reticulum	
*Ropn1l*	Ropporin-like protein (AKAP-associated sperm protein) [Mus musculus] (Asp)	70c	1.72	1.43	1.20	1.00	Testis specific protein. Predicted to interact with AKAP3 which is present in round spermatid acrosome	
*Ruvbl1*	RuvB-like 1	154	1.26	1.03	0.86	0.93	DNA helicase. Component of the NuA4 histone acetyltransferase complex which is involved in transcriptional activation of select genes. Associates with tubulins in the mitotic spindle and centrosome	Meiotic division, apoptosis
*Sae1*	SUMO-activating enzyme subunit 1	330	1.20	1.21	0.97	1.12	Subunit of sumo activating enzyme, participates in post-translational sumoylation of target proteins. SUMO modification regulates activity of numerous transcription factors with roles in cell-cycle, apoptosis and development	Meiosis, general cell function
*Sdha*	Succinate dehydrogenase [ubiquinone] flavoprotein subunit, mitochondrial precursor (Fp)	213	0.93	0.91	1.02	0.74	Flavoprotein (FP) subunit of succinate dehydrogenase involved in mitochondrial electron transport chain. Known to be changed in response to cellular stress	Response to cell stress
*Sept2*	Septin-2	174	1.19	1.02	0.96	0.83	Required for normal cell division	Meiotic division
*Sf3a1*	Splicing factor 3a, subunit 1 (SAP114)	356	0.91	1.07	1.10	0.94	Component of splicing factor SF3A, functions early during pre-mRNA splicing	RNA splicing
*Sf3a3*	Splicing factor 3a, subunit 3 [Mus musculus] (SAP61)	201a	0.83	1.05	1.16	0.93	Component of splicing factor SF3A, functions early during pre-mRNA splicing	RNA splicing
*Sfrs7*	Splicing factor, arginine/serine-rich 7 *(9G8)*	150b	0.79	0.92	1.00	1.16	Well known component of human spliceosome and is required for pre-mRNA splicing	RNA splicing
*Tbca*	Tubulin cofactor a	286	1.03	1.19	1.16	1.34	Involved in tubulin folding, acts as a molecular chaperone for tubulins	Meiotic division, general cell function
*Thop1*	Thimet oligopeptidase	160a	0.89	0.95	1.14	0.78	Involved in small peptide metabolism, broad substrate specificity	
*Tpm3*	Tropomyosin alpha-3 chain	290	1.22	1.03	0.95	1.18	Binds and stabilises actin filaments, required for cell division in some cells	Meiotic division
*Tsta3*	Tissue specific transplantation antigen P35B	285	1.14	1.03	0.88	0.97	An NADP(H)-binding protein that catalyses reactions in GDP-D-mannose metabolism, converting GDP-4-keto-6-D-deoxymannose to GDP-L-fucose	
*Tuba1c*	Tubulin alpha-1C chain **^d^**	93a	1.39	1.03	0.87	0.89	Constituent of microtubules, roles in meiotic division	Meiotic division, general cell function
*Tuba3a*	Tubulin alpha-3 chain **^d^**	344a	1.18	1.04	0.96	0.98	Constituent of microtubules, roles in meiotic division	Meiotic division, general cell function
*Tubb2c*	Tubulin beta-2C chain **^d^**	47a	1.34	0.93	0.90	0.73	Constituent of microtubules, roles in meiotic division	Meiotic division, general cell function
*Ube2N*	Ubiquitin-conjugating enzyme E2N (Ubc13)	141	0.59	0.83	0.85	0.88	Role in poly-ubiquitination that mediates transcriptional activation. Roles in cell cycle and survival, DNA repair. Participates in polyubiquitination of PCNA which is required for DNA repair	DNA repair
*Ywhab*	14-3-3 protein beta/alpha	137a	1.58	1.15	1.06	1.25	Participate in protein-protein interactions to control a wide variety of processes. Known to inhibit testis specific kinase 1 (TESK1) which is expressed in pachytene spermatocytes [93]	Meiosis, apoptosis, cell signalling
*Ywhae*	14-3-3 protein epsilon (14-3-3e)	29, 130, 240	1.32	0.84	0.64	1.30	Participate in protein-protein interactions to control a wide variety of processes including cell cycle and apoptosis	Meiosis, apoptosis, cell signalling
*Ywhag*	14-3-3 protein gamma	218b	1.44	1.04	1.05	1.22	Participate in protein-protein interactions to control a wide variety of processes including cell cycle and apoptosis	Meiosis, apoptosis, cell signalling
*Ywhaz*	14-3-3 protein zeta/delta	137b, 218a	1.58	1.15	1.06	1.25	Participate in protein-protein interactions to control a wide variety of processes including cell cycle and apoptosis	Meiosis, apoptosis, cell signalling

† Further information on protein identity, fold changes between treatment groups, false discovery rate, power value, and localisation of mRNA expression in the seminiferous epithelium is given in Table S1 and S2. Information on peptide identification scores, number of peptides identified and % sequence coverage is shown in Table S2. Actual mass spectra for all identified proteins are shown in Table S3. Abbreviations: PSC  =  pachytene spermatocytes.

a Summary of likely major functions based on information from UniProt, GO annotations, Entrez Gene ID and PubMed.

b Brief overview of potential role in meiotic cells based on known functions and/or previously described roles in the testis.

c Tubulin isoforms identified in the same spot, see Table S1 and S2.

To identify biological processes in spermatocytes that might be changed during testicular androgen manipulation, protein accession numbers (Table S1) were analysed using Metacore 5.0 software (GeneGo), and processes (based on GO ontology and GeneGo annotations) showing significant (p<0.05) associations were examined. Processes of relevance to meiotic cells were significantly enriched in the dataset (Table S4), and included cell division, microtubule-based movement and spindle assembly, regulation of apoptosis, spermatid development and nuclear mRNA splicing.

In order to shed light on the likely role of each protein in the context of spermatocyte function, literature searches were performed to examine known cellular function(s) and whether a role in spermatocytes has been described (Table 1). Many of the proteins had not been previously described in pachytene spermatocytes, although there was evidence of mRNA expression in these cells (Table S1). Based on previously published information and protein annotations, at least 37 of the proteins identified (Table 1) are likely to play roles in apoptosis and/or cell/meiotic division, and could thus be involved in the survival of pachytene spermatocytes and/or meiotic division. Throughout the Results and Discussion, gene names as well as protein names are given, so as to avoid confusion with different protein nomenclatures.

### Androgen-responsive Proteins Associated with Cellular Stress and Apoptosis

It was noted that many of the proteins showing significant changes between the high and low testicular androgen groups had roles in metabolism and response to cellular stress (Table 1 and Table S4). Androgen manipulation caused changes in several protein disulfide-isomerases (*P4hb1*/*Pdia1*, *Pdia3* and *Pdia6*, Table 1), and heat shock proteins (*Hspd1*, *Hspa2*, *Hspa9*, Table 1), which are modulated in other models of cellular stress (e.g.[32]–[34]). GeneGo analysis demonstrated a significant association of these proteins with various processes related to cell stress, including oxidative stress, unfolded protein response and DNA damage (Table S4), suggesting that androgen-deprivation is associated with the induction of cellular stress in meiotic cells.

Testicular testosterone suppression has been shown to adversely influence antioxidant activity in testicular cells [35], and various proteins implicated in managing oxidative stress were identified in the current study (Table 1). For example, androgen deprivation was associated with an increase in heavy and light ferritin subunits (*Fth1*, *Ftl1*, Table 1). Both heavy and light chains of the ferritin complex are induced in response to oxidative stress in HeLa cells, and overexpression of these proteins reduces reactive oxygen species (ROS) generation after oxidative stress challenge [36]. *Hspd1* (Hsp60 protein, see Table 1) is a well known molecular chaperone with a multitude of functions, including roles in response to oxidative stress [34] and apoptosis, was also induced during androgen suppression. This finding is consistent with a previous study showing it is increased in the testis in response to flutamide [37].

Proteins with known roles as protectors of oxidative stress were identified in spermatocytes, and were lower during androgen deprivation (*Aldh2*, *Prdx6*, *Gstm5*, Table 1). Deficiencies in mitochondrial aldehyde dehydrogenase (*Aldh2*) are associated with an increased susceptibility to oxidative stress [38], whereas peroxiredoxin 6 (*Prdx6*) is a member of a family of antioxidant proteins which protect cells from oxidative stress. *Gstm5* is a member of the glutathione S transferases (GSTs) that inactivate various metabolites produced during oxidative stress. *Gstm5* is a divergent member of the GST family expressed primarily in testis and may serve to protect spermatogenic cells from oxidative stress [39]. Thus testicular androgen suppression caused an increase in meiotic cell proteins known to be induced during oxidative stress and a decrease in proteins known to protect against oxidative stress.

**Figure 3:**
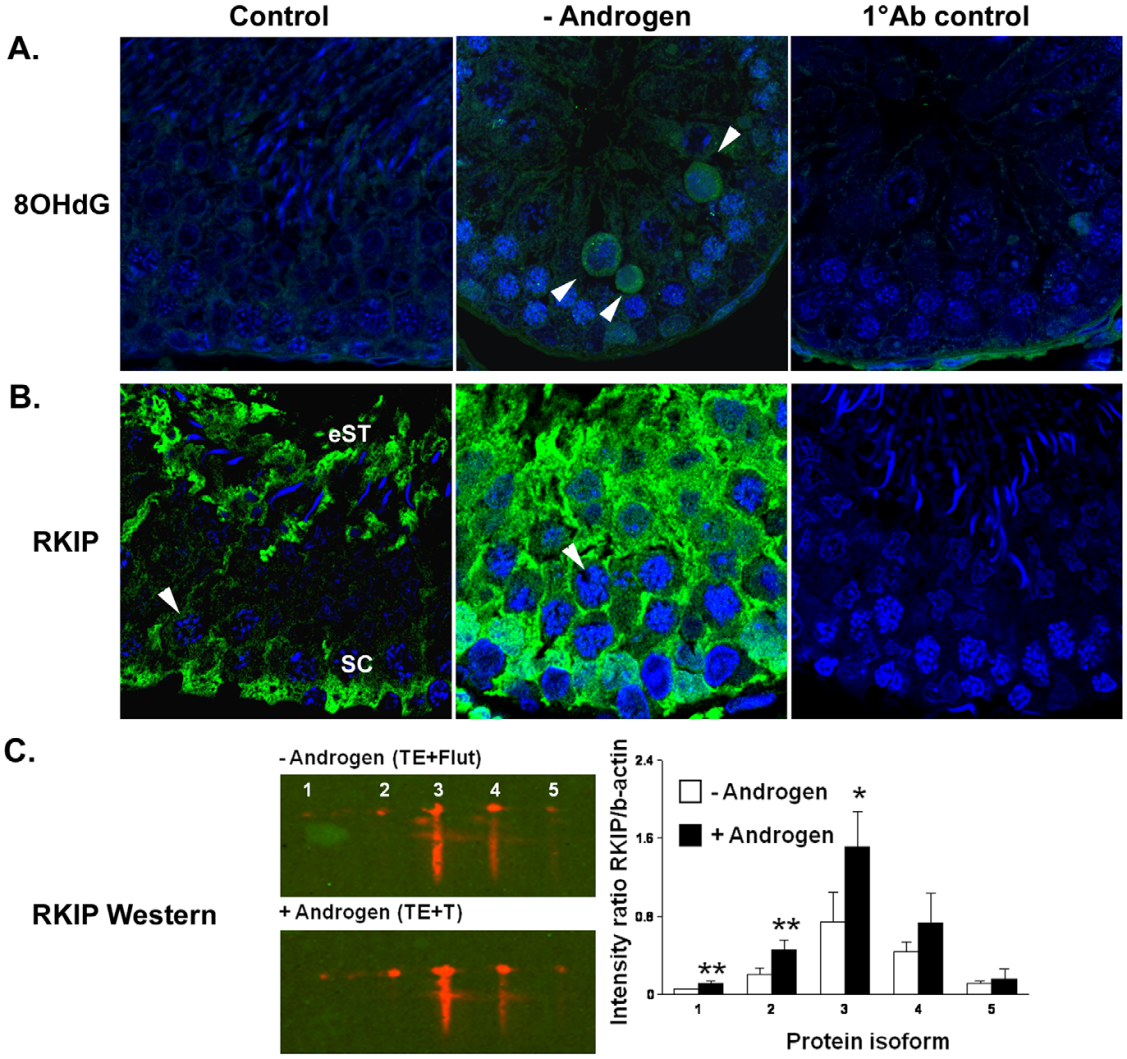
Localisation of oxidised DNA adducts and RKIP during androgen manipulation *in vivo*. **A.** Immunohistochemical localisation of oxidised DNA adducts as detected by 8OHdG (green) labelling in testis from *Control* and *- Androgen* (androgen suppressed, TE+Flutamide) rats. A negative control for the primary antibody is also shown (*1°Ab Control*) in a TE+Flutamide-treated testis. Positively labelled pachytene spermatocytes were only apparent during androgen suppression (arrowheads). **B.** Immunohistochemical localisation of RKIP (green) in testis from *Control* and *- Androgen* (androgen suppressed, TE+Flutamide). A negative control for the primary antibody is also shown (*1°Ab Control*). In controls, staining was most apparent in Sertoli cells (SC) and elongating spermatid cytoplasm (eST), but was faintly present in pachytene spermatocyte cytoplasm (arrowheads). During androgen suppression (*- Androgen*) a marked increase in immunostaining for RKIP was noted throughout the epithelium, with cytoplasmic staining more obvious in pachytene spermatocytes (arrowhead). In A and B, nuclei are labelled blue (TOPRO). **C.** Confirmation of changes in expression of androgen-responsive RKIP isoforms; the left panel shows representative images of the 2D-Western during androgen blockade (-Androgen, TE+Flutamide) compared to androgen replacement (+ Androgen, TE+T24). Blots were performed on pooled samples from the same individual animals used for the 2D-DIGE proteomics. Five distinct isoforms (#1– # 5) were resolved. Results of the densitometric analysis (right panel) from 2D western blots revealed that 3 isoforms showed significant (* p<0.05, ** p<0.01) differences between the –Androgen and + Androgen groups. Data is shown as mean ± SD (n = 4 separate experiments).

Whether oxidative stress occurs in spermatocytes during androgen withdrawal *in vivo* was examined using immunostaining for 8-hydroxydeoxyguanosine (8OHdG) which labels oxidised DNA adducts (Figure 3A). Very few cells with positively stained nuclei were apparent in controls, however they were frequently observed during androgen deprivation. The morphology of the labelled cells was consistent with pachytene spermatocytes undergoing apoptosis. This suggests that spermatocytes are subjected to oxidative DNA damage during androgen withdrawal, and that such damage may result in apoptosis. Of relevance was the demonstration that Ndufs1 protein was decreased during androgen suppression (see *Ndufs1* in Table 1, spot #334 in Tables S1, S2). This protein, as well as playing a role in oxidative metabolism, is integrally involved in the initial apoptotic response of mitochondria [40]. Cleavage of mitochondrial Ndufs1 by caspases leads to the production of ROS and subsequent permeabilisation of the mitochondrial membrane. There have been no descriptions of Ndufs1 in the testis, however its mRNA is maximally expressed in pachytene spermatocytes (Table S1) and thus it is possible that cleavage of Ndufs1 may participate in the apoptotic and oxidative stress response in these cells.

Consistent with the fact that androgen-suppression causes apoptosis of pachytene spermatocytes, androgen-responsive proteins with roles in apoptosis were identified (Table 1, Table S4). Testicular androgen suppression was associated with down-regulation of a 19 kDa protein identified as Diablo (or Smac) (Table 1). Diablo is produced as a 29 kDa form and N-terminally processed to a 23 kDa protein resident in the mitochondria; upon an apoptotic stimulus 23 kDa Diablo is released into cytoplasm where it binds to and inhibits IAPs (inhibitors of apoptosis) preventing their inhibitory interaction with caspases and facilitating apoptosis [41]. Consistent with this, androgen and FSH suppression causes an induction of Diablo protein in testis cytosolic fractions, and translocation from the mitochondria to the nucleus in pachytene spermatocytes [20], indicating that Diablo translocation is involved in androgen-dependent apoptosis of pachytene spermatocytes. Smaller molecular weight, alternatively spliced forms of Diablo/Smac that are functionally distinct yet pro-apoptotic have been described in humans, Smacβ (21 kDa) [42] and Smac3 (19 kDa) [43]. It remains to be determined whether the androgen-responsive 19 kDa form of Diablo observed in the current study (spot #68, Table S2) is an as-yet undescribed alternatively spliced variant of rat Diablo, whose function may differ from the 23 kDa form.

Signalling proteins with known roles in apoptosis were changed in meiotic cells during *in vivo* testicular androgen manipulation. Two spots were identified as *Pebp1* (Table 1), commonly known as Raf kinase Inhibitory Protein (RKIP). RKIP negatively regulates Raf-MEK-ERK by binding to Raf1, and also regulates the NFκB and G protein-coupled receptor signalling pathways depending on its phosphorylation state. RKIP-mediated modulation of these signalling pathways in turn regulates various cellular processes, most notably apoptosis, reviewed in [44]. RKIP has been implicated in sperm capacitation [45], but a role in other testicular cells has not been described. That RKIP pI isoforms were changed significantly during androgen manipulation was confirmed by 2D Western, with 3 out of the 5 charge isoforms showing significant differences between the low and high androgen groups (Figure 3C). RKIP mRNA is expressed in both pachytene spermatocytes and round spermatids, however the protein is more abundant in spermatids compared to spermatocytes [46], as confirmed by immunohistochemistry in the current study (Figure 3B), suggesting that the protein is subjected to post-translational control [46]. While RKIP mRNA levels were not changed when Leydig cells were depleted from the testis [46], a marked increase in immunostaining was seen during androgen deprivation *in vivo* (Figure 3B). Taken together, the results suggest that RKIP translation and/or post-translational modification in germ cells is changed during testicular androgen manipulation.

Five spots showing significant changes during testicular androgen manipulation *in vivo* were identified as 14-3-3 proteins (*Ywhab*, *Ywhae*, *Ywhag*, *Ywhaz*, see Table 1). These are phospho-serine/threonine proteins that interact with numerous binding partners, in a phosphorylation-dependent manner, to modulate cell growth, survival and differentiation. They play multiple roles in mediating the balance between survival and apoptosis, and can suppress cell cycle progression in response to DNA damage [47]. 14-3-3 proteins are abundant and have been immunolocalised to germ cells including pachytene spermatocytes [48]. 14-3-3-interacting proteins have been recently identified in mouse testis [49], and these included several proteins identified in this study: *Ddx4*, *Fabp9*, *Gstm5*, *Hnrpk*, *Hspa2*, *Ipo5*, *Ltzfl1*, *Ldhb*, *Ldhc*, *Nasp*, *Pebp1*, *Ppt1*, *Pdia3* and *Pdia6*. The current findings suggest that changes in 14-3-3 proteins, probably via post-translational modifications such as phosphorylation, are involved in androgen-mediated regulation of spermatocyte function.

### Proteins with Known Roles in Meiosis

A number of proteins identified in this study have previously been shown to have roles in cell division, and could thus be involved in meiotic division and be responding to androgen-mediated signals from AR-expressing somatic cells. For example, components of the dynactin complex were identified as androgen-responsive in enriched meiotic cell preparations (*Capza1*, *Capza2*, *Actg1* and *Actr1a*, see Table 1). The dynactin complex has an essential role in cell division, including nuclear envelope breakdown and mitotic spindle organisation, by virtue of its interaction with the motor protein dynein [50]. *Mapre1* (Table 1), known as EB1 protein, has various roles in cell division and spindle dynamics, and interacts with dynactin [51]. *Ruvbl1* (Table 1) and a number of tubulin isoforms (*Tuba1c*, *Tuba3a* and *Tubb2c*, see Table 1) were identified as changed in response to testicular androgen manipulation; Ruvbl1 is also involved in cell division and colocalises with tubulins in the mitotic spindle [52]. Interestingly, spots mapping to *Capza1/Capza2 Actr1a*, *Actg1*, *Mapre1*, *Ruvbl1* and tubulins all showed similarity in their fold changes (1.02–1.76 fold upregulated with high androgen, Table 1).

Two proteins known as Hsp 70–2 *(Hspa2*, Table 1) and NASP (*Nasp*, Table 1) were identified in meiotic cells. Hspa70–2 is a molecular chaperone expressed specifically in spermatogenic cells that is essential for the G2-M phase transition in meiosis I [53], whereas NASP is a linker histone chaperone required for normal cell division [54]. Testicular NASP and Hsp70–2 co-localise at synaptonemal complexes, where NASP modulates the ability of Hsp70–2 to bind to CDC2, in turn modulating the formation of the active CDC2/cyclin B1 complex required for the G2-M phase transition [55]. NASP binds to Hsp70–2 and increases its ability to bind to CDC2, thereby preventing formation of the active CDC2/cyclin B1 complex; the authors speculate that over-expression of NASP would inhibit progression of meiosis [55]. Of interest is the fact that we observed a 1.6 fold up-regulation (see spot #83 in Table S1) of NASP during AR blockade (TE+Flutamide treatment), when meiotic division would likely be compromised. Further studies are needed to elucidate whether the NASP-Hsp70-2 interaction is a key mechanism by which androgenic signals from Sertoli cells regulates meiotic division.

### Proteins with Roles in RNA Splicing and Processing

Several proteins that are components of the spliceosome and are involved in RNA splicing were identified as changed in response to testicular androgen manipulation. *Sf3a1* and *Sf3a3* (Table 1) are components of the trimeric SF3A complex which functions during early pre-mRNA splicing [56]. *Prpf19* (known as SNEV), *Sfrs7* (known as 9G8), *Hnrnph3* (known as hnRNP H) and *Hnrnpk* (known as hnRNP K) are also present in the spliceosome [57], [58] and were changed during testicular androgen manipulation (Table 1). Alternative splicing is particularly prevalent in the testis and the expression of ubiquitous, tissue- and cell-type specific nuclear RNA binding proteins is modulated during germ cell development [59]. Pachytene spermatocytes show a peak in mRNA transcription from stage VII onwards [15], [60] and transcribe mRNAs associated with meiotic division [16]. Pachytene spermatocytes in these stages also show a peak in immunostaining of small nuclear ribonucleoproteins, central components of the spliceosome [61]. Taken together, this suggests that pachytene spermatocytes produce mRNAs needed for the completion of meiotic division, and that splicing of these RNAs may be modulated during testicular androgen manipulation.

**Figure 4:**
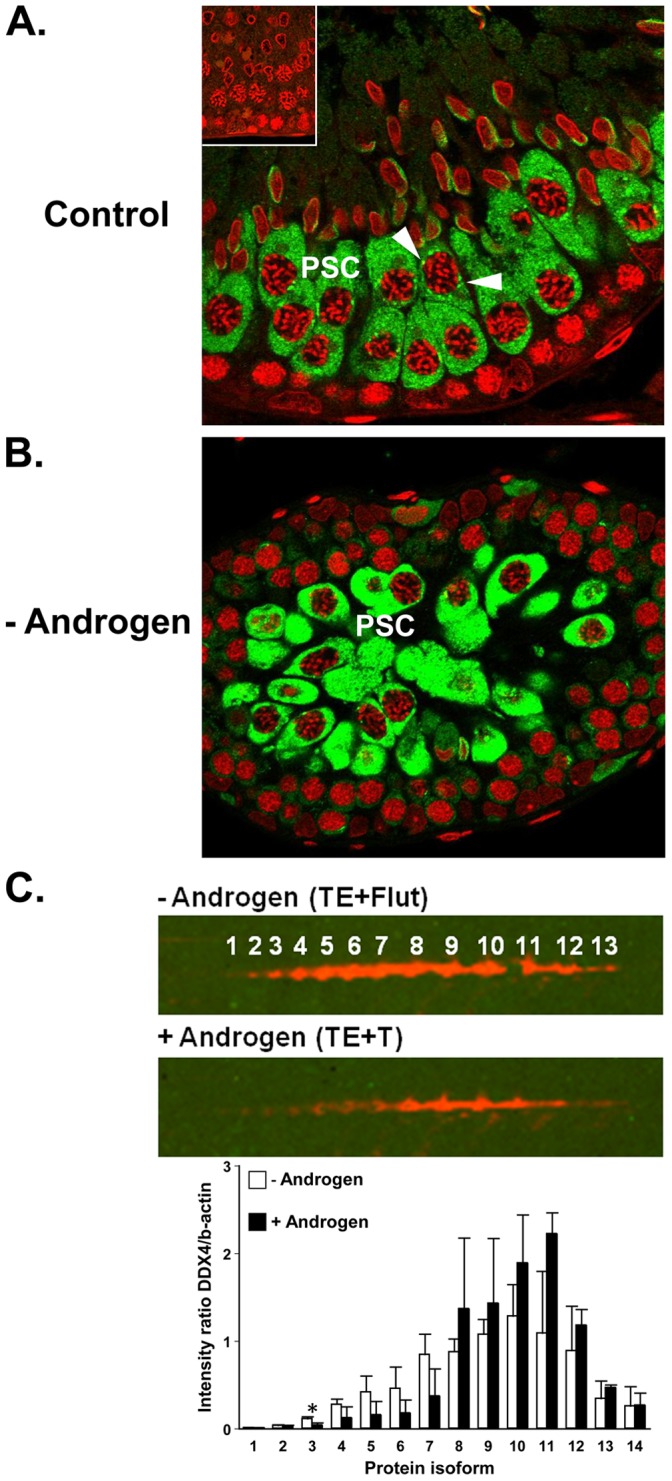
DDX4 during androgen manipulation. **A.** Immunohistochemical localisation of DDX4 (green) in control testis. Staining is observed in late pachytene spermatocyte (PSC) cytoplasm and chromatoid body precursor structures in the perinuclear region (arrowheads). *Inset* shows control for the primary antibody. **B.** During androgen blockade (TE+Flutamide), DDX4 immunostaining intensity increased in late pachytene spermatocyte (PSC) cytoplasm. In panels A and B, cell nuclei were visualized with TOPRO (red). **C.** Evaluation of androgen-responsive pI isoforms of DDX4; the upper panel shows a representative image for the 2D-Western during androgen blockade (-Androgen, TE+Flutamide) compared to androgen replacement with T24 (TE+T24, +Androgen). Fourteen distinct pI isoforms were resolved. Results of the densitometric analysis of pooled samples (lower panel) from –Androgen and +Androgen groups (*for details see* *Figure 3* *legend*) revealed that one isoform showed a significant (p<0.05, t-test) difference between these groups (asterix), however the other isoforms showed trends to increase or decrease with androgen replacement. Data is shown as mean ± SD (n = 3 separate experiments).

*Ddx4* encodes a protein known as DDX4 (Table 1) or mouse vasa homologue (Mvh) and DDX4 protein was identified to be statistically different between the low and high androgen groups (Table 1). 2D Western showed that 14 pI isoforms were present in enriched meiotic cells, and confirmed that one of these was significantly increased during androgen suppression (Figure 4C). This result is supported by a previous proteomic study suggesting that DDX4 is increased during androgen suppression in human testis biopsies [62]. Immunohistochemical analysis confirmed that, during normal spermatogenesis, DDX4 localises to pachytene spermatocyte cytoplasm and chromatoid body precursor structures in the perinuclear region of these cells (Figure 4A), as previously described [63]. Androgen suppression *in vivo* caused a marked increase in the immunostaining of DDX4 in pachytene spermatocyte cytoplasm (Figure 4B). It is well known that DDX4/Mvh is required for early meiotic prophase [64] and that it is a key component of the chromatoid body in pachytene spermatocytes and spermatids [65]. The chromatoid body is thought to co-ordinate different translational regulation pathways in male germ cells, such as miRNA-mediated post-transcriptional regulation, and DDX4/Mvh interacts with Dicer and Argonaute/Piwi proteins involved in miRNA processing, reviewed in [65]. Thus it is possible that Sertoli cells, or perhaps other AR-expressing testicular somatic cells, under the influence of androgens, regulate germ cell expression of DDX4/Mvh isoforms which in turn could influence RNA processing and post-transcriptional regulation.

Consistent with the hypothesis that androgen modulation causes changes in proteins involved in RNA processing, Prmt5 was identified, along with another two proteins, in spot #108 (Table S2). This protein is essential for the dimethylation of spliceosomal components [66] and of Piwi proteins in the chromatoid body [67], and mutation of the Drosophila homologue Dart5 causes arrest of spermatocyte development [66].

### Proteins involved in post-translational processing

SUMO activating enzyme subunit 1 (*Sae1*) was lower during AR blockade (TE+Flut) (Table 1). SUMO1-3 are small proteins that are covalently attached to other proteins; this reversible post-translational modification known as sumoylation modifies protein function and is involved in regulating various cell functions. The Sae1 subunit dimerises with Sae2 to form the E1 activating enzyme, the first in a three-step cascade leading to sumoylation of a protein. Sumoylation pathways in male germ cells have been well described, and sumoylation may play an important role in meiosis [68], however the protein targets of sumoylation in germ cells are not well understood.

**Figure 5:**
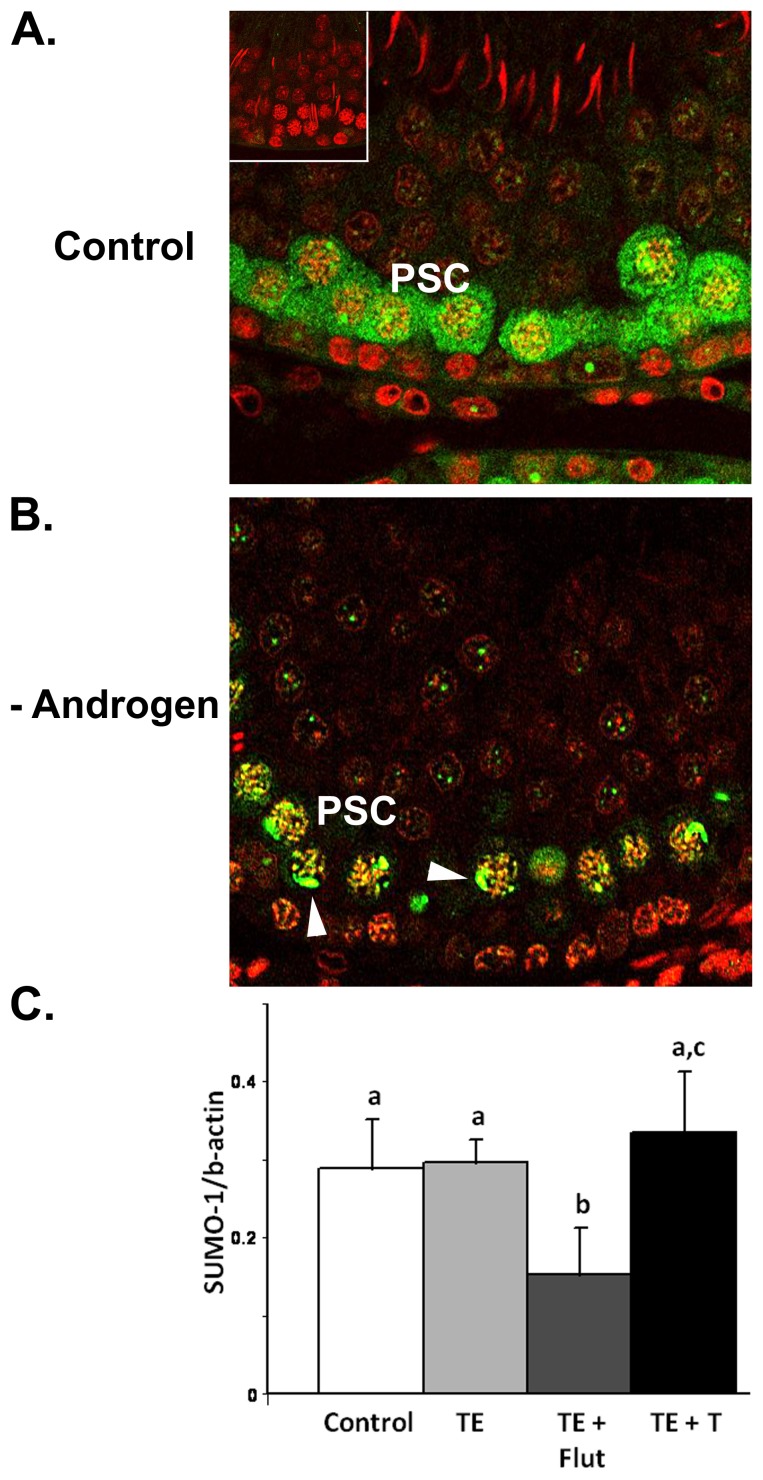
SUMO1 during androgen manipulation. **A.** Immunohistochemical localisation of SUMO1 (green) in control testis. Staining is observed in the cytoplasm of pachytene spermatocytes (PSC) in this stage VII tubule, whereas no staining was observed in the primary antibody control (*inset*). Cell nuclei were visualized with TOPRO (red). **B.** SUMO1 immunostaining in pachytene spermatocyte (PSC) cytoplasm was reduced during androgen suppression, however staining associated with the nuclei and the XY body (arrowheads) was preserved. **C.** Densitometric analysis of 15 kDa SUMO1 (i.e. ‘free’ SUMO1) in 1D-western blots with n = 4 separate animals/group from the four different treatments. Different letters denote statistical differences (p<0.01) between groups. During androgen blockade (TE+Flut), there was a significant decrease in free SUMO1 compared to control. Data is shown as mean ± SD.

In order to investigate whether SUMO proteins may be modulated in meiotic cells during testicular androgen suppression, immunohistochemical analysis of SUMO1 was performed (Figure 5). The results confirmed the previously described localisation of SUMO1 in pachytene spermatocyte nuclei, including a concentration in the XY body [69]. Specific cytoplasmic staining was also observed in these cells (Figure 5A); this was most obvious in stages V-VIII. Consistent with previous studies [68], [69], SUMO1 staining disappeared from spermatocytes during meiotic division (not shown). During androgen suppression, nuclear localisation including XY body immunostaining appeared preserved however there was a marked reduction in cytoplasmic staining (Figure 5B). Since immunohistochemistry does not distinguish between free SUMO1 and sumoylated proteins, Western blot analysis was performed to investigate changes in free SUMO1. In this case, 1D western blots were sufficient as SUMO1 only gave a single spot in the 2D western format (data not shown). SUMO1 was significantly (p = 0.008) decreased between control and TE+Flut groups (Figure 5C), whereas androgen replacement with T24 restored SUMO1 to control levels (TE+flut vs TE+T24, p = 0.005) (Figure 5C). These observations are consistent with the demonstration of a reduction in free SUMO1 protein during androgen suppression in prostate [70].

Taken together, the above results suggest that testicular androgen suppression may cause changes in sumoylation within meiotic cells, both by decreasing the levels of free SUMO and/or decreasing enzymes involved in the sumoylation process. A recent study showed that sumoylation, and the level of free SUMO1, is modulated in meiotic cells in response to oxidative stress induced by hydrogen peroxide or to exposure to tobacco smoke [71]. Thus the data suggest that meiotic cells respond to cellular stress, including stress induced by androgen deprivation, by modulating protein sumoylation.

### Proteins Involved in DNA Repair

Double strand breaks in DNA are initiated and subsequently repaired during homologous chromosome recombination in meiosis, and thus meiotic cells are well equipped with DNA repair enzymes [14], [72]. Given that spermatocytes during androgen suppression showed changes in stress response proteins and evidence of oxidised DNA adducts (Figure 3A), it is possible that other forms of DNA damage occur in these cells during androgen deprivation. Consistent with this proposition, the process network ‘DNA damage checkpoint’ was significantly associated with the differentially-regulated proteins as identified by bioinformatic analyses (Table S4), and proteins with roles in DNA repair were identified (Table 1).

*Prpf19* (SNEV, see Table 1) has a role in RNA splicing (see above), but is also known to have a direct role in DNA repair, and a loss of *Prpf19* expression in lymphoid cells causes an accumulation of double strand breaks and apoptosis [73]. Upon DNA damage, SNEV is ubiquitylated and forms a higher molecular weight oligomeric complex that binds to chromatin [74]. This protein has not been described in the testis, however its mRNA is expressed in Sertoli cells and germ cells (Table S1).

PCNA is involved in DNA replication and as such is well known to be expressed in proliferating cells in the testis (e.g. [75]), however it is also pivotal in the activation of particular DNA repair pathways, reviewed in [76], [77]. PCNA’s ability to modulate DNA repair is controlled by post-translational modifications, including ubiquitylation and sumoylation of a lysine residue at position 164 [78]. Models in yeast suggest that mono-ubiquitylation of lysine 164 of PCNA initiates the translesion synthesis DNA repair pathway, whereas multi-ubiquitylation of this residue directs the initiation of the error free DNA repair pathway [76]–[78]. Previous studies in mice demonstrated PCNA protein in the nuclei of spermatocytes in prophase I, suggesting it may play a role in DNA repair during homologous chromosome recombination [79]. This proposition has been recently supported by studies in yeast, where PCNA plays an essential role in mismatch repair pathways during meiotic recombination [80]. Transgenic male mice expressing PCNA mutated at amino acid 164, and unable to be ubiquitylated, are infertile due to arrest of spermatogenesis at the early pachytene stage [81]. Taken together, there is emerging evidence that PCNA, and its post-translational modifications, participates in essential DNA repair pathways during meiosis.

**Figure 6:**
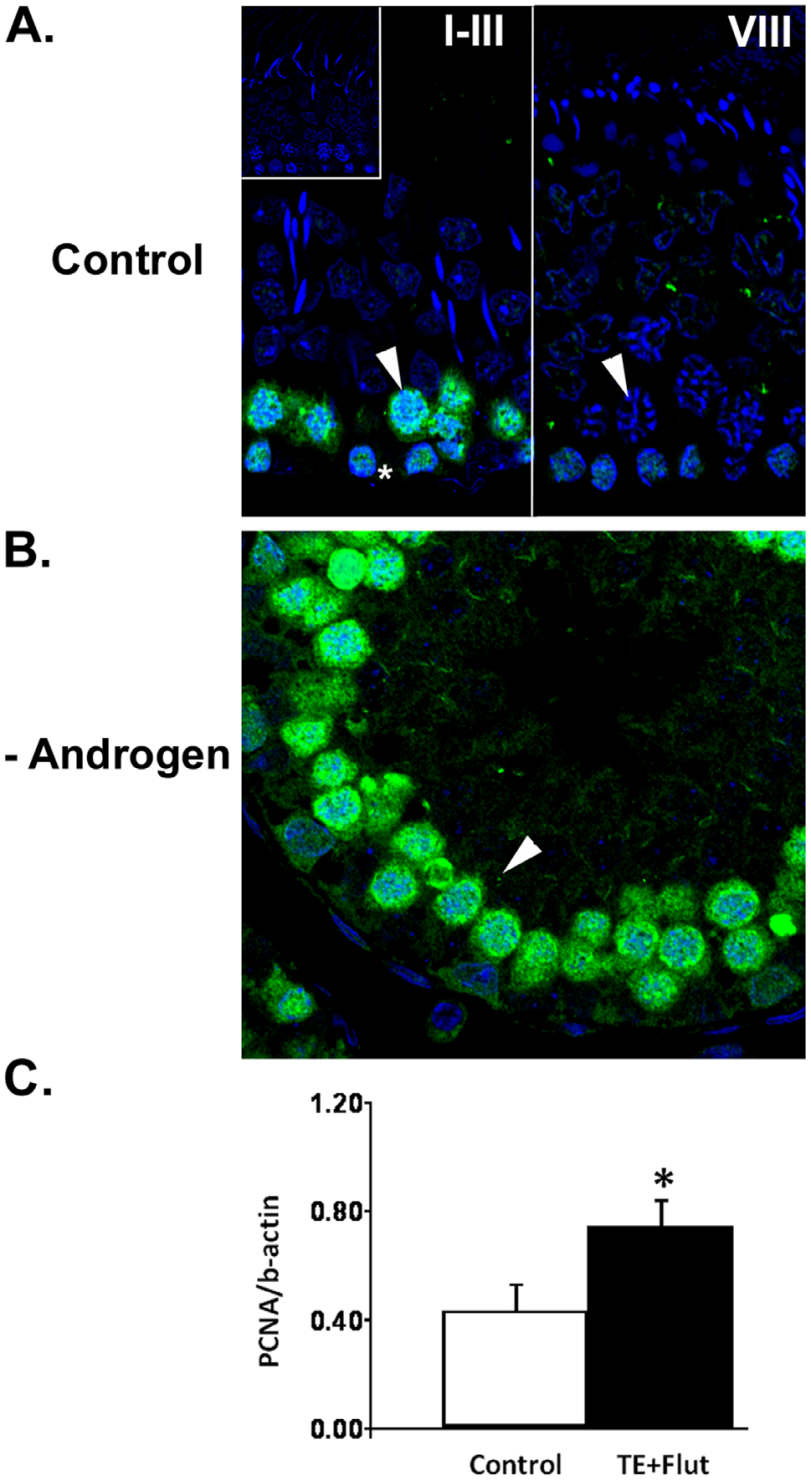
PCNA during androgen manipulation. **A.** PCNA immunostaining (green) in control testis. Representative images from stages I-III and VIII are shown, with visualization of cell nuclei using TOPRO (blue). Pachytene spermatocytes (arrowheads) were immuno-positive in the early stages, but became immuno-negative around stages VII-VIII. PCNA was also observed in proliferating spermatogonia (asterix), whereas no staining was observed in the primary antibody control (inset). **B.** PCNA immunostaining was more intense in pachytene spermatocytes (arrowhead) in stages I-VI when androgen action was suppressed. **C.** Densitometric analysis of PCNA in 1D Western blots revealed a significant (p = 0.001, asterix) increase in PCNA protein during androgen blockade (TE+Flut) compared to control. Data is shown as mean ± SD (n = 5).

We showed that PCNA was changed during testicular androgen suppression (Table 1), with a significant increase in expression during androgen blockade compared to control confirmed by Western blot (Figure 6C). The localisation of PCNA in meiotic spermatocytes previously observed in mice [79] was confirmed here in rats (Figure 6A), with PCNA observed in proliferating spermatogonia and a sub-set of spermatocytes. Nuclear localisation was first observed in leptotene spermatocytes, when meiotic double strand breaks are induced, and became progressively more intense as spermatocytes proceeded through prophase I (data not shown). Staining was most obvious in the nucleus and cytoplasm of pachytene spermatocytes in stages ∼I-V (Figure 6A). Pachytene spermatocytes became abruptly immuno-negative in stages VII-VIII (Figure 6A). During AR blockade, intense immunostaining of these spermatocyte sub-populations was observed (Figure 6B).

*Ube2N* (known as Ubc13 protein, Table 1) is the human homolog of UBC13 in yeast, a ubiquitin-conjugating enzyme involved in the error-free DNA post-replication repair pathway. Specifically, Ubc13 is part of a complex that, upon DNA damage, multi-ubiquitylates the lysine 164 reside of PCNA, resulting in a switch from the translesion synthesis pathway to the error-free DNA repair pathway [78], reviewed in [77]. A role for Ubc13 during meiosis has not been established, however it is immunolocalised in pachytene spermatocyte nuclei in mice [82]. It is tempting to speculate that the increase in Ubc13 observed during androgen suppression may be due to an increased requirement for ubiquitylation of PCNA and thus an enhanced ability to participate in DNA repair; this proposition merits further study.

### Conclusions

We conclude that androgen-dependent signals from Sertoli cells, and/or other AR-expressing somatic cells such as peritubular myoid cells, modulate key proteomic changes in meiotic cells, which in turn impact on their survival and completion of meiosis. Such changes are likely mediated by translational and post-translational mechanisms. The results provide a “snap shot” of the impact of this loss of androgen stimulus on meiotic cell function. The findings suggest that, upon the loss of androgen-dependent stimuli from somatic cells, meiotic cells undergo cellular stresses such as unfolded protein response and oxidative damage, DNA damage and apoptosis. Proteins with roles in cell survival and meiotic division are responsive to this androgen stimulus and are likely key intermediates in the androgen-dependent regulation of the completion of meiosis. The results also provide hypotheses for further testing, including the proposition that DNA repair mechanisms and RNA splicing are modulated in spermatocytes during testicular androgen suppression.

## Materials and Methods

### Animals

The rat model of testicular testosterone suppression used in this study has been extensively validated [27], [28], and employed silicone-tubing implants filled with either testosterone (T) powder (Sigma, St. Louis, MO) (3c m implant length) or estradiol (E) powder (Sigma) (0.4 cm length). Adult male Sprague-Dawley rats (80–100 days old) were obtained from Monash University Central Animal House and housed under a 12L:12D cycle with free access to food and water. Animals (n = 4–5/group) received one T plus one E implant (TE implants) subcutaneously, or no implants (control) for 9 wk [27]. At the end of the 9 wk period, all control and 4 of the TE-treated animals were killed. A further 4 TE-treated animals had their TE implants removed and replaced with 3×8 cm T implants (TE+T24) for 4 days to partially restore testicular testosterone levels [27], while the remaining 5 animals received daily injections of the androgen receptor antagonist, flutamide, (20 mg/kg, s.c., n = 5 animals) (TE+Flut) [29] for 7 days. Animals were then killed and left testes were removed for immediate isolation of spermatocytes while the right testes were processed for immunohistochemistry. Meiotic spermatocytes were isolated and enriched by elutriation centrifugation as described elsewhere [83]. For ploidy analysis, cells were fixed in ethanol overnight at 4°C, washed with DPBS containing 1% FCS, and stained with 250 ug/ml propidium iodide (Sigma) and 5 mg/ml RNase A (Sigma) in 38 mM sodium citrate (Sigma) pH 7.0 at 37°C for 30 min. Flow cytometric analysis indicated that the resultant germ cell preparation consisted mainly of tetraploid spermatocytes (62%), diploid cells, which would also include secondary spermatocytes (20%) and haploid round spermatids (15%). Pachytene spermatocytes comprise the major proportion of meiotic cells in the testis, and thus will comprise the major component of the meiotic cell preparation. This study was approved by the Monash Medical Centre Animal Ethics Committee, ethics permit #MMCB 2006/19.

### Sample Preparation and Expression Analysis by 2D-Difference In-Gel Electrophoresis (2D-DIGE)

Total proteins from enriched meiotic cell preparations were extracted and solubilised in 30 mM Tris/HCl, pH 8.1, 7 M urea, 2 M thiourea,1% (w/v) C7 detergent (C7BzO, Merck, Darmstadt, Germany) as previously described [84], [85]. Reductive alkylation and fluorescent protein labelling with minimal CyDyes and 2D-PAGE were also carried out as described [84]–[86]. Briefly, 40µg protein aliquots from individual animals (n = 4/group) were labelled with either Cy3 (control or TE+T24 groups), Cy5 (TE or TE+Flut groups) or Cy2 (mixed internal standard) and combined to be run on a total of 8 gels as recommended by the manufacturer. Isoelectric focussing was carried out using 24 cm pH 4-7 IPG strips according to the following parameters: constant 60µA/strip, 100V for 1.5 h, 300V for 1.5 h, gradient to 1000V over 4 h, gradient to 8000V over 3 h, constant 8000V until 60,000Vh was reached. Second dimension separation was carried out with 24 cm, 4–20% gradient acrylamide gels cast with a Bind-Silane (GE Biosciences, Little Chalfont, UK)-treated back plate, and run overnight at a constant voltage of 50V in a BioRad (Hercules, CA) Dodeca electrophoresis tank. Gels were scanned using a Fuji FLA5100 laser scanner, and differential expression analysis based on normalized spot volumes was performed using PG240 SameSpots software (Nonlinear Dynamics, Newcastle-upon-Tyne, UK). One gel, containing one sample each from the TE+T24 and TE+Flut groups, failed at the isoelectric focusing stage, and was excluded from subsequent analyses.

### Protein Identification

Protein spots of interest were excised using a ProPicII robotic spot picker (Digilab Genomic Solutions, Ann Arbor, MI) based on the X-Y coordinates exported directly from PG240 SameSpots. Protein identification (see Tables S2, S3) by MALDI-TOF MS and MS/MS was as described [84], or was by LC-MS/MS using a nano HPLC coupled on-line to an LTQ Orbitrap mass spectrometer (Thermo Fisher, Waltham, MA) at the Joint Proteomics Laboratory, http://www.ludwig.edu.au/Ludwig Institute for Cancer Research (Melbourne, Australia) [87].

### Immunohistochemistry

Testes were fixed in Bouin’s-fixative and embedded in paraffin wax. All immunohistochemical procedures were as previously described [28]. Primary antibodies employed were: DDX4 (1∶800; Abcam #13840,Cambridge, UK), RKIP/PEBP1 (1∶1000; Upstate Biotechnology #07-137, Lake Placid, NY), and Sumo-1 (1∶500; Abcam #ab32058). Cell nuclei were visualized with either DAPI (100µM) or TO-PRO-3 iodide (10µM) and sections were then prepared for either light or confocal microscopy as detailed elsewhere [28], [88]. Specificity of primary antibodies was verified by substitution of the primary antibody with an equivalent dilution of non-immune IgG.

### Western Blotting

One dimensional SDS PAGE was used as described [89] to validate expression differences between treatment groups, with the same primary antibodies (PCNA, 1∶500; SUMO-1, 1∶1000) as used for immunohistochemistry. Protein loads were normalised against β-actin (1∶15,000 for PCNA, 1∶2000 for SUMO1; MP Biomedicals #69100, Aurora, OH). Detection employed species-specific secondary antibodies labelled with Alexa Fluor 680 (1∶5,000; Molecular Probes, Eugene, OR) or IRDye 800 (1∶10,000; Rockland, Gilbertsville, PA) and blots were quantified using a fluorescent detection system (Odyssey, Li-Cor Biosciences, Lincoln, NE). To validate expression patterns of RKIP and DDX4 which were observed as isoforms in the 2D-DIGE data, 2D-Westerns were employed. Briefly, 35µg of pooled protein from either the TE+T24 or TE+flutamide groups were concurrently processed onto 11 cm pH 4-7 strips, and focussed using similar conditions as above, prior to second dimension SDS-PAGE using 11 cm 8–16% Tris-HCl precast gels (BioRad). Proteins were transferred onto PVDF membranes (Immobilon-P 0.45um, Millipore, Billerica, MA) (200V, 40 min) in a Criterion Blotter (BioRad), and then incubated with primary antibody for 1 hr at room temperature; (DDX4; 1∶240, PEBP1; 1∶1000, β-actin; 1∶10,000]. Detection was as for 1D SDS-PAGE, and total fluorescent volume for each spot was normalised to the summed spot volume for all β-actin spots. Western blots were repeated 3–4 times for each primary antibody, and similar results were obtained in each case.

### Statistical Analysis

Statistical analysis of proteomic data was performed automatically by the PG240 SameSpots software. Data are given as mean normalised spot volume ± standard deviation. Statistical analysis of immunoblotting results was carried out using SigmaStat v3.5 (Systat Software, Inc.,San Jose, CA) with all data assessed for normal distribution and equal variance, prior to ANOVA and post-hoc Student Newman-Keuls testing. Results with p<0.05 were considered significant, and are presented as mean ± standard deviation (SD) unless otherwise specified.

## Supporting Information

Table S1 Differentially expressed proteins in meiotic cell preparations.
(XLS)

Table S2 Differentially Expressed Proteins Identified by MALDI-TOF MS+MS/MS, PMF/MALDI, LCMS/MS Scaffold and LCMS/MS; detailed mass spectrometry data.
(XLS)

Table S3 Mass spectra for identified proteins.
(PDF)

Table S4 Bioinformatic analysis of androgen-responsive proteins in enriched meiotic cell preparations.
(XLS)
