# Supplementary material for: Proteomic Changes in Rat Spermatogenesis in Response to In Vivo Androgen Manipulation; Impact on Meiotic Cells
Source: PLoS One. 2012 Jul 30;7(7):e41718. doi: 10.1371/journal.pone.0041718 (PMC3408499; doi:10.1371/journal.pone.0041718)
Supplement: Table S3 — Mass spectra for identified proteins. (PDF) [file pone.0041718.s003.pdf]

Stanton, Sluka et al: Proteomic changes in rat spermatogenesis in response to in vivo androgen manipulation; impact on meiotic cells.

Table S3
Mass Spectra for Identified Proteins

Spot 1 (A3 20080606) – Phosphatidylethanolamine-binding protein 1 (Pebp1)

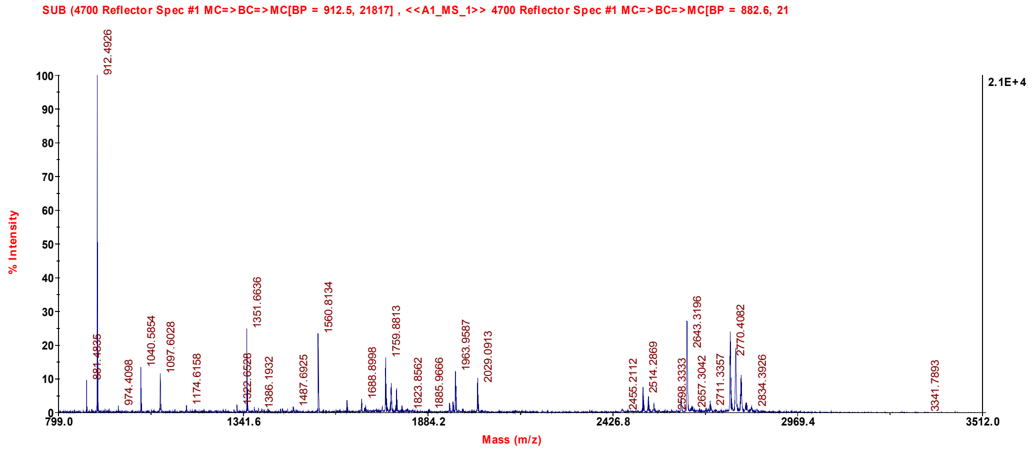

Spot 1 (20080606-A3) Pebp1								
MS+MS/MS								
Calculated	Observed	+/-ppm	start	end	Sequence	Ion Score	C.I.%	Modification
881.4839	881.4835	0	180	187	LHDQLAGK			
912.4937	912.4913	-3	149	155	FKVESFR			
912.4937	912.4913	-3	149	155	FKVESFR	55	100	
1351.674	1351.6621	-9	27	39	VDYGGVTVDELGK			
1351.674	1351.6621	-9	27	39	VDYGGVTVDELGK	50	99.999	
1560.8268	1560.8151	-7	63	76	LYTLVLTDPDAPSR	33	99.949	
1560.8268	1560.8151	-7	63	76	LYTLVLTDPDAPSR			
1688.9218	1688.9044	-10	63	77	LYTLVLTDPDAPSRK			
1758.8896	1758.8826	-4	81	93	FREWHHFLVVNMK			Oxidation (M)
1758.8896	1758.8826	-4	81	93	FREWHHFLVVNMK			Oxidation (M)
1963.9607	1963.9598	0	94	113	GNDISSGTVLSEYVGSGPPK	91	100	
1963.9607	1963.9598	0	94	113	GNDISSGTVLSEYVGSGPPK			
2514.2656	2514.29	10	40	62	VLTPTQVMNRPSSISWDGLDPGK			Oxidation (M)
2643.3008	2643.3284	10	94	119	GNDISSGTVLSEYVGSGPPKDTGLHR			
2643.3008	2643.3284	10	94	119	GNDISSGTVLSEYVGSGPPKDTGLHR	55	100	

Spot 1 (20080606-A3) Pebp1								
PMF/MALDI								
Calculated	Observed	+/-ppm	start	end	Sequence	Ion Score	C.I.%	Modification
1350.6667	1351.6636	-8	27	39	R.VDYGGVTVDELGK.V			
2513.2584	2514.2871	9	40	62	K.VLTPTQVMNRPSSISWDGLDPGK.L			Oxidation (M)
1559.8195	1560.8134	-9	63	76	K.LYTLVLTDPDAPSR.K			
1687.9145	1688.8999	-13	63	77	K.LYTLVLTDPDAPSRK.D			
2028.0891	2029.0914	-2	63	80	K.LYTLVLTDPDAPSRKDPK.F			
1757.8824	1758.8802	-5	81	93	K.FREWHHFLVVNMK.G			Oxidation (M)
1454.7129	1455.6968	-16	83	93	R.EWHHFLVVNMK.G			Oxidation (M)
1962.9535	1963.9589	-1	94	113	K.GNDISSGTVLSEYVGSGPPK.D			
2642.2936	2643.3196	7	94	119	K.GNDISSGTVLSEYVGSGPPKDTGLHR.Y			
1096.6029	1097.6028	-7	147	155	R.GKFKVESFR.K			
911.4865	912.4926	-1	149	155	K.FKVESFR.K			
1039.5815	1040.5854	-3	149	156	K.FKVESFRK.K			
2704.2955	2705.303	0	156	179	R.KKYHLGAPVAGTCFQAEWDDSVPK.L			
880.4767	881.4835	0	180	187	K.LHDQLAGK.-			

Spot 1 (D4 20080815) - Phosphatidylethanolamine-binding protein 1 (Pebp1)

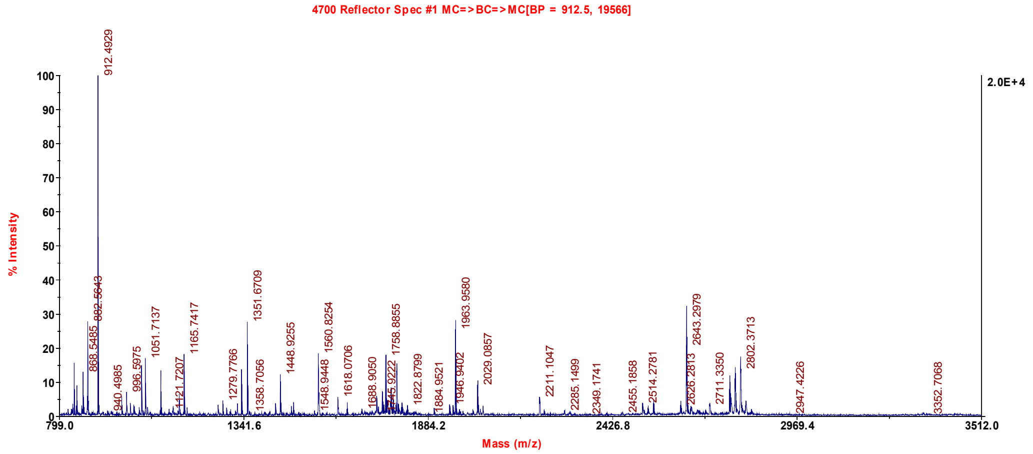

Spot 1 (20080815-D4) Pebp1								
MS+MS/MS								
Calculated	Observed	+/-ppm	start	end	Sequence	Ion Score	C.I.%	Modification
881.4839	881.4843	0	180	187	LHDQLAGK			
912.4937	912.4923	-2	149	155	FKVESFR			
912.4937	912.4923	-2	149	155	FKVESFR	34	99.957	
1351.674	1351.6698	-3	27	39	VDYGGVTVDELGK	54	100	
1351.674	1351.6698	-3	27	39	VDYGGVTVDELGK			
1560.8268	1560.8223	-3	63	76	LYTLVLTDPDAPSR			
1560.8268	1560.8223	-3	63	76	LYTLVLTDPDAPSR	26	99.727	
1758.8896	1758.8821	-4	81	93	FREWHHFLVVNMK			Oxidation (M)
1758.8896	1758.8821	-4	81	93	FREWHHFLVVNMK			Oxidation (M)
1963.9607	1963.9541	-3	94	113	GNDISSGTVLSEYVGSGPPK	66	100	
1963.9607	1963.9541	-3	94	113	GNDISSGTVLSEYVGSGPPK			
2514.2656	2514.2722	3	40	62	VLTPTQVMNRPSSISWDGLDPGK			Oxidation (M)
2643.3008	2643.2937	-3	94	119	GNDISSGTVLSEYVGSGPPKDTGLHR			
2643.3008	2643.2937	-3	94	119	GNDISSGTVLSEYVGSGPPKDTGLHR	57	100	

Spot 1 (D18 20080815) - Phosphatidylethanolamine-binding protein 1 (Pebp1)

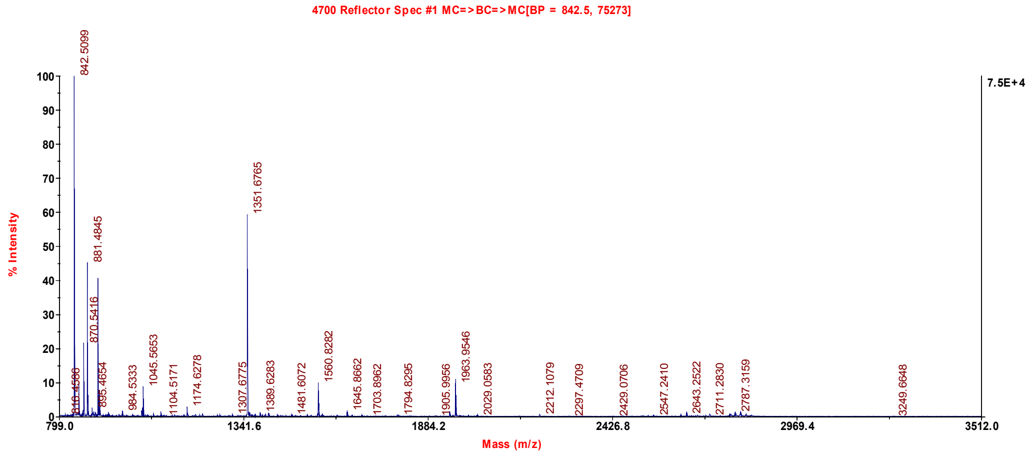

Spot 1 (20080815-D18) Pebp1								
PMF/MALDI								
Calculated	Observed	+/-ppm	start	end	Sequence	Ion Score	C.I.%	Modification
1350.6667	1351.6765	2	27	39	R.VDYGGVTVDELGK.V			
2513.2584	2514.2517	-6	40	62	K.VLTPTQVMNRPSSISWDGLDPGK.L			Oxidation (M)
1559.8195	1560.8282	1	63	76	K.LYTLVLTDPDAPSR.K			
1687.9145	1688.9125	-5	63	77	K.LYTLVLTDPDAPSRK.D			
2028.0891	2029.0584	-19	63	80	K.LYTLVLTDPDAPSRKDPK.F			
1757.8824	1758.83	-34	81	93	K.FREWHHFLVVNMK.G			Oxidation (M)
1438.718	1439.754	20	83	93	R.EWHHFLVVNMK.G			
1962.9535	1963.9546	-3	94	113	K.GNDISSGTVLSEYVGSGPPK.D			
2642.2936	2643.2522	-18	94	119	K.GNDISSGTVLSEYVGSGPPKDTGLHR.Y			
1096.6029	1097.6086	-1	147	155	R.GKFKVESFR.K			
911.4865	912.495	1	149	155	K.FKVESFR.K			
1039.5815	1040.5895	1	149	156	K.FKVESFRK.K			
892.513	893.4838	-41	151	157	K.VESFRKK.Y			
880.4767	881.4845	1	180	187	K.LHDQLAGK.-			

Spot 4 (C20 20080606) - Alpha-enolase (Eno1)

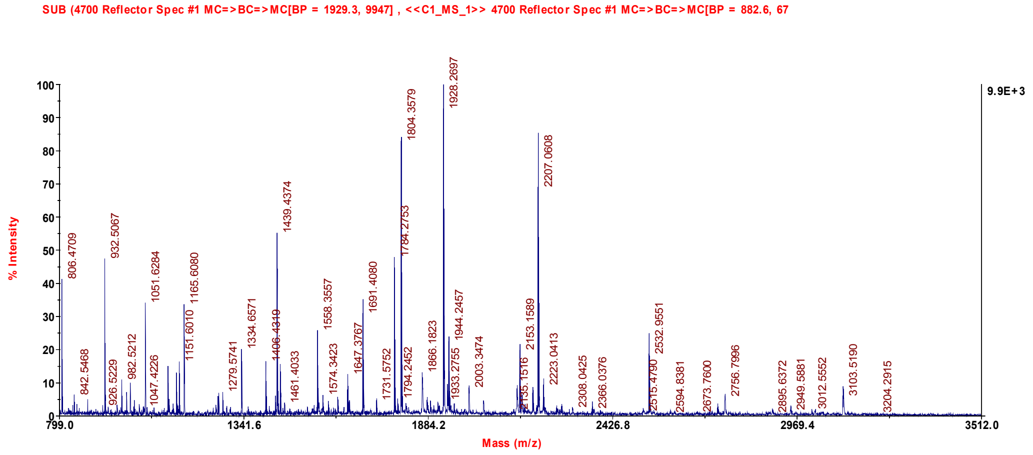

Spot 4 (20080606-C20) Eno1								
MS+MS/MS								
Calculated	Observed	+/-ppm	start	end	Sequence	Ion Score	C.I.%	Modification
806.4518	806.4478	-5	407	412	YNQILR			
806.4518	806.4478	-5	407	412	YNQILR	22	99.626	
932.5311	932.5351	4	427	434	SFRNPLAK			
932.5311	932.5351	4	427	434	SFRNPLAK	3	70.255	
1118.6681	1118.6691	1	404	412	LAKYNQILR			
1143.6156	1143.6167	1	184	193	IGAEVYHNLK			
1259.7106	1259.7148	3	121	132	AGAVEKGVPLYR			
1406.7162	1406.6876	-20	16	28	GNPTVEVDLYTAK			
1439.7417	1439.7106	-22	270	281	YITPDQLADLYK	47	99.999	
1439.7417	1439.7106	-22	270	281	YITPDQLADLYK			
1646.8319	1646.7548	-47	180	193	EAMRIGAEVYHNLK			Oxidation (M)
1691.8962	1691.8134	-49	407	420	YNQILRIEEELGSK			
1691.8962	1691.8134	-49	407	420	YNQILRIEEELGSK			

Spot 4 (D15 20070719) – Alpha-enolase (Eno1)

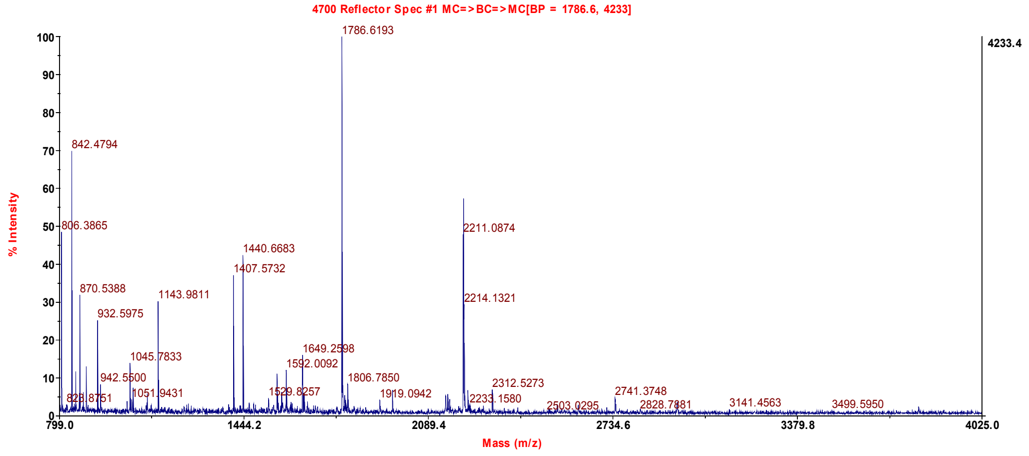

Spot 4 (20070719-D15) Eno1								
MS+MS/MS								
Calculated	Observed	+/-ppm	start	end	Sequence	Ion Score	C.I.%	Modification
806.4518	806.4626	13	406	411	YNQILR			
806.4518	806.4626	13	406	411	YNQILR	3	65.626	
932.5311	932.5218	-10	426	433	SFRNPLAK	12	95.732	
932.5311	932.5218	-10	426	433	SFRNPLAK			
1056.536	1056.531	-5	253	261	AGKYDLDFK			
1143.6156	1143.6307	13	183	192	IGAEVYHNLK	27	99.88	
1143.6156	1143.6307	13	183	192	IGAEVYHNLK			
2192.0327	2192.1135	37	233	252	AGYTDQVVIGMDVAASEFYR			
2208.0278	2208.1079	36	233	252	AGYTDQVVIGMDVAASEFYR			Oxidation (M)

Spot 9 (P1014-16) – mixture of: Aldose reductase (Akr1b1), Palmitoyl-protein thioesterase 1 (Ppt-1) and L-lactate dehydrogenase C chain (Ldhc)

Spot 9 (P1014-16) Akr1b1, Ppt-1 & Ldhc
LCMS/MS (Scaffold)

Sample Name	Acc No.	Proteins(s) inferred	No. Peptides identified	Sequence Coverage	Position in sequence	Peptide Sequence	Precursor m/z	Charge observed	Calculated mass (neutral)	Mascot score	Mascot Expect value
P1014-16	P07943	sp|P07943|Aldose reductase Tax_Id=10116 [Rattus norvegicus]	1	4.43%	156 - 169	(K)AIGVSNFNPLQIER(I)	779.22	2	1556.42	78.3	5.48E-005
	P45479	sp|P45479|Palmitoyl-protein thioesterase 1 Tax_Id=10116 [Rattus norvegicus]	1	4.90%	254 - 268	(K)ETIPLQETTLYTEDR(L)	905.22	2	1808.43	91.4	2.39E-006
	P19629	sp|P19629|L-lactate dehydrogenase C chain Tax_Id=10116 [Rattus norvegicus]	1	5.72%	2 - 20	(M)sTVKEQLIQNLAPDEKQSR(C)	742.56	3	2224.66	58.0	5.87E-003

Spot 9 [P1014-16 (75965 Rattus)] Akr1b1
LCMS/MS

Calculated	Observed	+/-ppm	start	end	Sequence	Ion Score	C.I.%	Modification
1212.7078	606.9384	1	54	64	K.EVGVALQEKLK.E	9		
1567.6799	523.7704	0	79	90	K.LWCTFHDQSMVK.G	7		Oxidation (M)
1556.8311	779.2179	0	156	169	K.AIGVSNFNPLQIER.I	78		
1556.8311	779.2619	0	156	169	K.AIGVSNFNPLQIER.I	50		

Spot 9 [P1014-16 (75965 Rattus)] Ppt-1
LCMS/MS

Calculated	Observed	+/-ppm	start	end	Sequence	Ion Score	C.I.%	Modification
952.4549	476.9599		1	8	-.MASPGYRR.L	11		Oxidation (M)
3121.5624	1041.2379		123	151	R.AVAQRCPTPPMMTLISVGGQHQGVFGLPR.C	10		Oxidation (M)
1037.5505	519.8224		165	174	R.KSLNAGAYSK.V	33		
2279.1281	760.6489		249	268	R.SGQAKETIPLQETTLYTEDR.L	22		
1807.884	905.2199		254	268	K.ETIPLQETTLYTEDR.L	91		
1335.7584	446.9589		274	285	K.MDKAGKLVFLAK.E	15		Oxidation (M)

Spot 9 [P1014-16 (75075 Rodent)] Ldhc
LCMS/MS

Calculated	Observed	+/-ppm	start	end	Sequence	Ion Score	C.I.%	Modification
2225.1651	742.5602		2	20	M.STVKEQLIQNLAPDEKQSR.C	58		Acetyl (Protein N-term)

Spot 16 (D14 20080815) - mCG11326, isoform CRA_b [Mus musculus] (Hnrnph3) and or Heterogeneous nuclear ribonucleoprotein H3 [Mus musculus] (MCG11326, isoform CRA_a) (Hnrnph3)

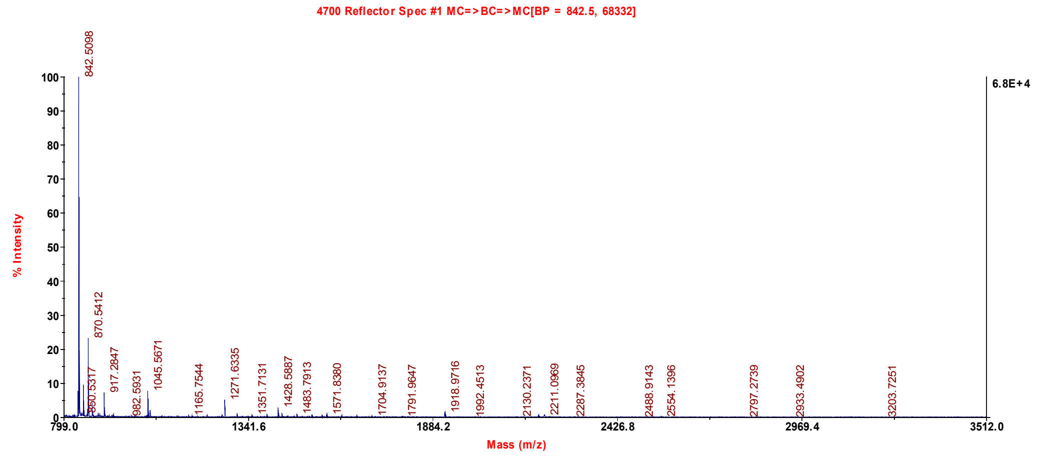

Spot 16 (20080815-D14) Hnrnph3 (mCG11326, isoform CRA_b)								
PMF/MALDI								
Calculated	Observed	+/-ppm	start	end	Sequence	Ion Score	C.I.%	Modification
1338.5913	1339.6135	11	7	19	K.HNGPNDASDGTVR.L			
1270.6194	1271.6335	5	56	67	R.STGEAFVQFASK.E			
2196.1062	2197.0503	-29	56	76	R.STGEAFVQFASKEIAENALGK.H			
943.4974	944.5088	4	68	76	K.EIAENALGK.H			
839.4541	840.4645	4	85	90	R.YIEIFR.S			
805.4294	806.4384	2	91	97	R.SSRSEIK.G			
1307.651	1308.6807	17	94	104	R.SEIKGFYDPPR.R			
850.3974	851.4082	4	98	104	K.GFYDPPR.R			
1006.4985	1007.5095	4	98	105	K.GFYDPPRR.L			
1917.9585	1918.9716	3	191	207	R.ATENDIANFFSPLNPIR.V			
1051.5411	1052.5562	7	208	217	R.VHIDIGADGR.A			
2192.9896	2194.0154	8	218	238	R.ATGEADVEFVTHEDAVAAMSK.D			Oxidation (M)
2553.1265	2554.1396	2	247	272	R.YIELFLNSTPGGGSGMGGSGMGGYGR.D			2 Oxidation (M)
1427.5736	1428.5887	6	273	286	R.DGMDNQGGYGSVGR.M			Oxidation (M)

Spot 16 (20080815-D14) Hnrnph3 (mCG11326, isoform CRA_a)								
PMF/MALDI								
Calculated	Observed	+/-ppm	start	end	Sequence	Ion Score	C.I.%	Modification
1338.5913	1339.6135	11	7	19	K.HNGPNDASDGTVR.L			
1270.6194	1271.6335	5	56	67	R.STGEAFVQFASK.E			
2196.1062	2197.0503	-29	56	76	R.STGEAFVQFASKEIAENALGK.H			
943.4974	944.5088	4	68	76	K.EIAENALGK.H			
839.4541	840.4645	4	85	90	R.YIEIFR.S			
805.4294	806.4384	2	91	97	R.SSRSEIK.G			
1307.651	1308.6807	17	94	104	R.SEIKGFYDPPR.R			
850.3974	851.4082	4	98	104	K.GFYDPPR.R			
1006.4985	1007.5095	4	98	105	K.GFYDPPRR.L			
1917.9585	1918.9716	3	206	222	R.ATENDIANFFSPLNPIR.V			
1051.5411	1052.5562	7	223	232	R.VHIDIGADGR.A			
2192.9896	2194.0154	8	233	253	R.ATGEADVEFVTHEDAVAAMSK.D			Oxidation (M)
2553.1265	2554.1396	2	262	287	R.YIELFLNSTPGGGSGMGGSGMGGYGR.D			2 Oxidation (M)
1427.5736	1428.5887	6	288	301	R.DGMDNQGGYGSVGR.M			Oxidation (M)

Spot 23 (D16 20070719) – Alpha-enolase (Eno1)

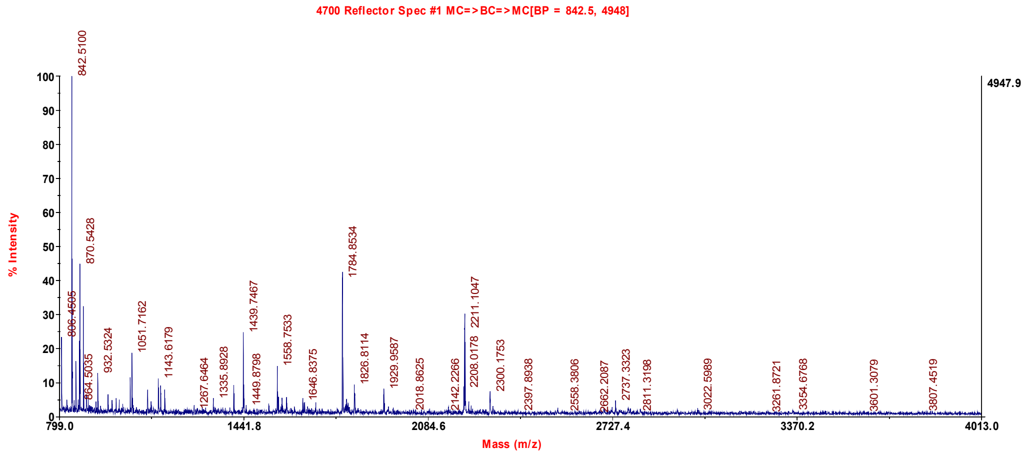

Spot 23 (20070719-D16) Eno1								
MS+MS/MS								
Calculated	Observed	+/-ppm	start	end	Sequence	Ion Score	C.I.%	Modification
806.4518	806.4495	-3	406	411	YNQILR			
806.4518	806.4495	-3	406	411	YNQILR			
899.556	899.5547	-1	71	79	TIAPALVSK			
932.5311	932.5314	0	426	433	SFRNPLAK			
932.5311	932.5314	0	426	433	SFRNPLAK			
1118.6681	1118.678	9	403	411	LAKYNQILR			
1143.6156	1143.6194	3	183	192	IGAEVYHNLK			
1406.7162	1406.7246	6	15	27	GNPTVEVDLYTAK			
1439.7417	1439.75	6	269	280	YITPDQLADLYK			
1439.7417	1439.75	6	269	280	YITPDQLADLYK			
1528.6914	1528.6998	5	256	268	YDLDFKSPDDASR			
1557.759	1557.7505	-5	358	371	LAQSNGWGVMVSHR			Oxidation (M)
1646.8319	1646.8419	6	179	192	EAMRIGAEVYHNLK			Oxidation (M)
1652.7506	1652.8015	31	89	102	IDQLMIEMDGTENK			Oxidation (M) [5]
1784.845	1784.8573	7	253	268	AGKYDLDFKSPDDASR			
1784.845	1784.8573	7	253	268	AGKYDLDFKSPDDASR	13	95.649	
1804.9438	1804.9683	14	32	49	AAVPSGASTGIYEALELR			
1928.9608	1928.9613	0	162	178	LAMQEFMILPVGASSFR			2 Oxidation (M)
1960.9246	1960.9446	10	202	220	DATNVGDEGGFAPNILENK			
2208.0278	2208.0281	0	233	252	AGYTDQVVIGMDVAASEFYR			Oxidation (M)
2208.0278	2208.0281	0	233	252	AGYTDQVVIGMDVAASEFYR			Oxidation (M)
2309.1042	2309.1313	12	199	220	YGKDATNVGDEGGFAPNILENK			
2534.2844	2534.3169	13	32	55	AAVPSGASTGIYEALELRDNDKTR			

Spot 23 (20070719-D16) Eno1								
PMF/MALDI								
Calculated	Observed	+/-ppm	start	end	Sequence	Ion Score	C.I.%	Modification
1405.7089	1406.7225	5	16	28	R.GNPTVEVDLYTAK.G			
1803.9366	1804.9624	10	33	50	R.AAVPSGASTGIYEALELR.D			
898.5488	899.5552	-1	72	80	K.TIAPALVSK.K			
1651.7433	1652.7972	28	90	103	K.IDQLMIEMDGTENK.S			Oxidation (M)
1667.7382	1668.8114	40	90	103	K.IDQLMIEMDGTENK.S			2 Oxidation (M)
1927.9536	1928.9573	-2	163	179	K.LAMQEFMILPVGASSFR.E			2 Oxidation (M)
1645.8246	1646.8374	3	180	193	R.EAMRIGAEVYHNLK.N			Oxidation (M)
1142.6084	1143.6179	2	184	193	R.IGAEVYHNLK.N			
2308.0971	2309.113	4	200	221	K.YGKDATNVGDEGGFAPNILENK.E			
1959.9174	1960.9355	6	203	221	K.DATNVGDEGGFAPNILENK.E			
2661.3108	2662.2087	-41	229	253	K.SAIAKAGYTDQVVIGMDVAASEFYR.A			
2207.0205	2208.0178	-5	234	253	K.AGYTDQVVIGMDVAASEFYR.A			Oxidation (M)
1527.6841	1528.693	1	257	269	K.YDLDFKSPDDASR.Y			
1438.7344	1439.7467	4	270	281	R.YITPDQLADLYK.S			
1556.7518	1557.7461	-8	359	372	K.LAQSNGWGVMVSHR.S			Oxidation (M)
1117.6607	1118.6769	8	404	412	R.LAKYNQILR.I			
805.4446	806.4505	-2	407	412	K.YNQILR.I			
1690.8889	1691.9098	8	407	420	K.YNQILRIEEELGSK.A			
903.4549	904.4266	-39	413	420	R.IEEELGSK.A			
931.5239	932.5324	1	427	434	R.SFRNPLAK.-			

Spot 29 (P1014-09) – 14-3-3 protein epsilon (Ywhae)

Spot 29 (P1014-09) Ywhae												
LCMS/MS (Scaffold)												
Sample Name	Acc No.	Proteins(s) inferred	No. Peptides identified	Sequence Coverage	Position in sequence	Peptide Sequence	Precursor m/z	Charge observed	Calculated mass (neutral)	Mascot score	Mascot Expect value
P1014-09	P62260	sp|P62260|14-3-3 protein epsilon Tax_Id=10116 [Rattus norvegicus]	3	14.10%	143 - 153	(K)EAAENSLVAYK(A)	597.66	2	1193.30	53.2	1.99E-002
					30 - 42	(K)VAGmDVELTVEER(N)	732.10	2	1462.19	49.9	3.88E-002
					1 - 12	(-)mDDREDLVYQAK(L)	762.70	2	1523.39	52.5	2.08E-002

Spot 29 [P1014-9 (75067 Rodent)] Ywhae								
LCMS/MS								
Calculated	Observed	+/-ppm	start	end	Sequence	Ion Score	C.I.%	Modification
1523.6926	762.7021		1	12	-.MDDREDLVYQAK.L	52		Acetyl (Protein N-term)
1539.6875	770.7592		1	12	-.MDDREDLVYQAK.L	41		Acetyl (Protein N-term); Oxidation (M)
1539.6875	771.7377		1	12	-.MDDREDLVYQAK.L	24		Acetyl (Protein N-term); Oxidation (M)
1927.8655	644.1605		13	28	K.LAEQAERYDEMVESMK.K	21		
2071.9554	691.4199		13	29	K.LAEQAERYDEMVESMKK.V	9		Oxidation (M)
1574.7974	526.1412		29	42	K.KVAGMDVELTVEER.N	27		
1462.6974	732.1017		30	42	K.VAGMDVELTVEER.N	50		Oxidation (M)
1193.5928	597.6581		143	153	K.EAAENSLVAYK.A	53		
1288.5419	645.1182		245	255	K.EALQDVEDENQ.-	35		

Spot 30 (E13 20070719) – Ferritin light chain 1 (Ftl1)

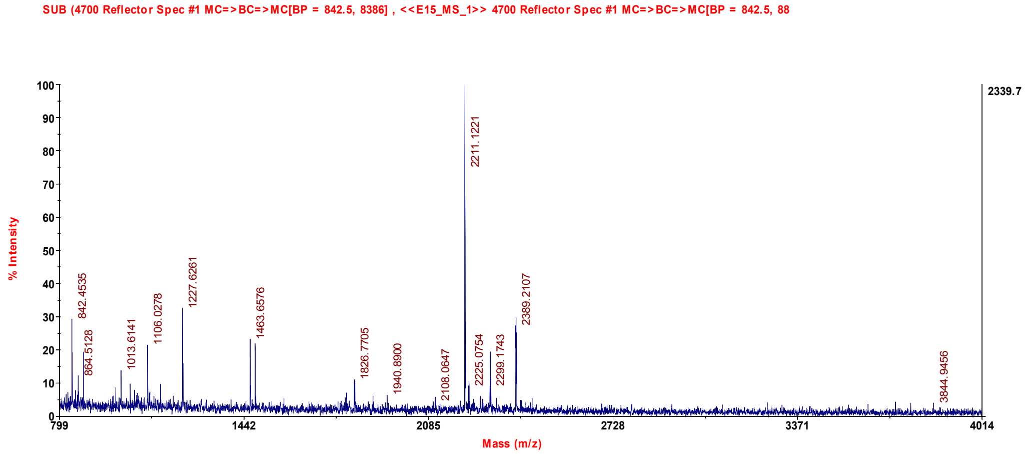

Spot 30 (20070719-D14) Ftl1								
PMF/MALDI								
Calculated	Observed	+/-ppm	start	end	Sequence	Ion Score	C.I.%	Modification
1479.6954	1480.6949	-5	7	19	R.QNYSTEVEAAVNR.L			
863.5341	864.5128	-33	20	26	R.LVNLHLR.A			
1012.6029	1013.6141	4	66	73	R.LLKLQNER.G			
1070.5291	1071.5247	-11	145	153	K.MGNHLTNLR.R			Oxidation (M)
1226.6302	1227.6261	-9	145	154	K.MGNHLTNLRR.V			Oxidation (M)
2388.2074	2389.2107	-2	155	177	R.VAGPQPAQTGVAQASLGEYLFER.L			

Spot 41 (D12 20070719) – Alpha-enolase (Eno1)

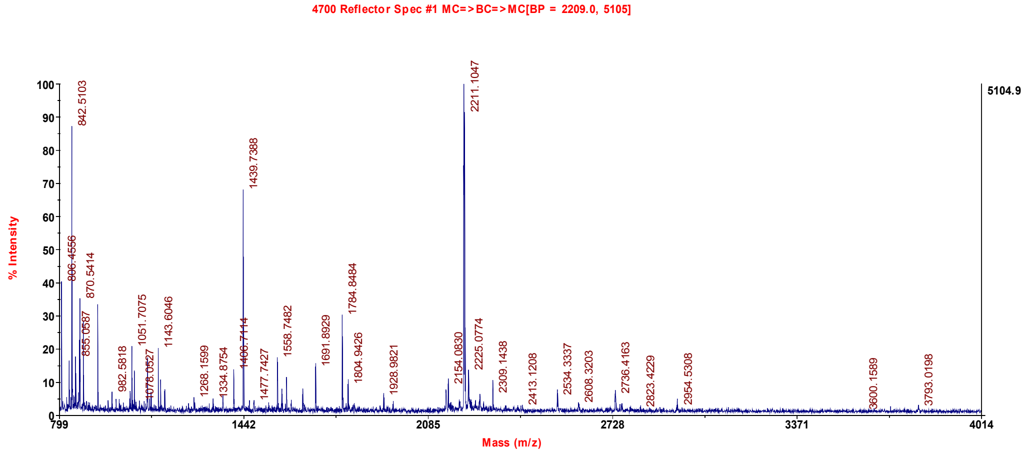

Spot 41 (20070719-D12) Eno1								
**MS+MS/MS**								
Calculated	Observed	+/-ppm	start	end	Sequence	Ion Score	C.I.%	Modification
806.4518	806.4531	2	406	411	YNQILR			
806.4518	806.4531	2	406	411	YNQILR			
932.5311	932.5335	3	426	433	SFRNPLAK			
932.5311	932.5335	3	426	433	SFRNPLAK			
1118.6681	1118.6665	-1	403	411	LAKYNQILR			
1143.6156	1143.6078	-7	183	192	IGAEVYHNLK			
1143.6156	1143.6078	-7	183	192	IGAEVYHNLK	1	15.85	
1406.7162	1406.7178	1	15	27	GNPTVEVDLYTAK			
1439.7417	1439.7451	2	269	280	YITPDQLADLYK			
1439.7417	1439.7451	2	269	280	YITPDQLADLYK	23	99.476	
1646.8319	1646.8364	3	179	192	EAMRIGAEVYHNLK			Oxidation (M)
1691.8962	1691.9052	5	406	419	YNQILRIEEELGSK			
1691.8962	1691.9052	5	406	419	YNQILRIEEELGSK			
1784.845	1784.8613	9	253	268	AGKYDLDFKSPDDASR	13	94.775	
1784.845	1784.8613	9	253	268	AGKYDLDFKSPDDASR			
1804.9438	1804.9519	4	32	49	AAVPSGASTGIYEALELR			
1928.9608	1928.9991	20	162	178	LAMQEFMILPVGASSFR			2 Oxidation (M)
2154.0713	2154.1028	15	9	27	EIFDSRGNPTVEVDLYTAK			
2154.0713	2154.1028	15	9	27	EIFDSRGNPTVEVDLYTAK			
2168.0505	2168.0942	20	262	280	SPDDASRYITPDQLADLYK			
2208.0278	2208.0747	21	233	252	AGYTDQVVIGMDVAASEFYR			Oxidation
2208.0278	2208.0747	21	233	252	AGYTDQVVIGMDVAASEFYR	13	94.691	Oxidation
2309.1042	2309.1675	27	199	220	YGKDATNVGDEGGFAPNILENK			
2534.2844	2534.3625	31	32	55	AAVPSGASTGIYEALELRDNDKTR			
2534.2844	2534.3625	31	32	55	AAVPSGASTGIYEALELRDNDKTR			
2608.248	2608.3098	24	81	102	LNVVEQEKIDQLMIEMDGTENK			2 Oxidation (M)
2736.343	2736.4453	37	80	102	KLNVVEQEKIDQLMIEMDGTENK			2 Oxidation (M)

Spot 41 (20070719-D12) Eno1								
**PMF/MALDI**								
Calculated	Observed	+/-ppm	start	end	Sequence	Ion Score	C.I.%	Modification
2153.0641	2154.083	5	10	28	R.EIFDSRGNPTVEVDLYTAK.G			
1405.7089	1406.7114	-3	16	28	R.GNPTVEVDLYTAK.G			
1803.9366	1804.9427	-1	33	50	R.AAVPSGASTGIYEALELR.D			
2276.1284	2277.1792	19	33	54	R.AAVPSGASTGIYEALELRDNDK.T			
898.5488	899.5755	22	72	80	K.TIAPALVSK.K			
2607.2408	2608.3203	28	82	103	K.LNVVEQEKIDQLMIEMDGTENK.S			2 Oxidation (M)
1651.7433	1652.8271	46	90	103	K.IDQLMIEMDGTENK.S			Oxidation (M)
1927.9536	1928.9822	11	163	179	K.LAMQEFMILPVGASSFR.E			2 Oxidation (M)
1645.8246	1646.8251	-4	180	193	R.EAMRIGAEVYHNLK.N			Oxidation (M)
1142.6084	1143.6046	-10	184	193	R.IGAEVYHNLK.N			
2308.0971	2309.1438	17	200	221	K.YGKDATNVGDEGGFAPNILENK.E			
1959.9174	1960.941	8	203	221	K.DATNVGDEGGFAPNILENK.E			
2207.0205	2208.054	12	234	253	K.AGYTDQVVIGMDVAASEFYR.A			Oxidation (M)
1055.5287	1056.5496	13	254	262	R.AGKYDLDFK.S			
1527.6841	1528.698	4	257	269	K.YDLDFKSPDDASR.Y			
2167.0433	2168.0747	11	263	281	K.SPDDASRYITPDQLADLYK.S			
1438.7344	1439.7388	-2	270	281	R.YITPDQLADLYK.S			
1117.6607	1118.6625	-5	404	412	R.LAKYNQILR.I			
805.4446	806.4556	5	407	412	K.YNQILR.I			
1690.8889	1691.8929	-2	407	420	K.YNQILRIEEELGSK.A			
931.5239	932.5334	2	427	434	R.SFRNPLAK.-			

Spot 46 (A12 20080606) – mCG11326, isoform CRA_b [Mus musculus] (Hnrnph3) and or Heterogeneous nuclear ribonucleoprotein H3 [Mus musculus] (MCG11326, isoform CRA_a) (Hnrnph3)

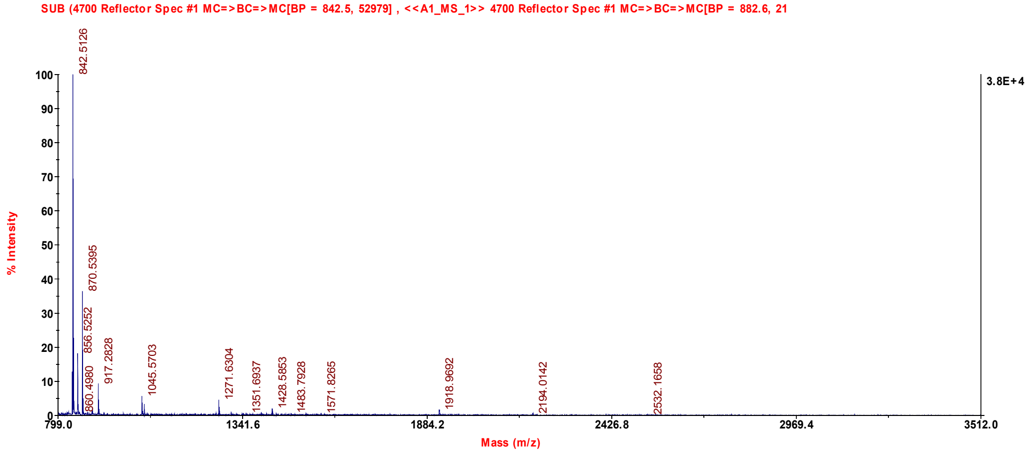

Spot 46 (20080606-A12) Hnrnph3 (mCG11326, isoform CRA_b)								
PMF/MALDI								
Calculated	Observed	+/-ppm	start	end	Sequence	Ion Score	C.I.%	Modification
1270.6194	1271.6304	3	56	67	R.STGEAFVQFASK.E			
839.4541	840.4634	2	85	90	R.YIEIFR.S			
1917.9585	1918.9692	2	191	207	R.ATENDIANFFSPLNPIR.V			
1051.5411	1052.5513	3	208	217	R.VHIDIGADGR.A			
2192.9896	2194.0142	8	218	238	R.ATGEADVEFVTHEDAVAAMSK.D			Oxidation (M)
1427.5736	1428.5853	3	273	286	R.DGMDNQGGYGSVGR.M			Oxidation (M)

Spot 46 (20080606-A12) Hnrph3 (mCG11326, isoform CRA_a)								
PMF/MALDI								
Calculated	Observed	+/-ppm	start	end	Sequence	Ion Score	C.I.%	Modification
1270.6194	1271.6304	3	56	67	R.STGEAFVQFASK.E			
839.4541	840.4634	2	85	90	R.YIEIFR.S			
1917.9585	1918.9692	2	206	222	R.ATENDIANFFSPLNPIR.V			
1051.5411	1052.5513	3	223	232	R.VHIDIGADGR.A			
2192.9896	2194.0142	8	233	253	R.ATGEADVEFVTHEDAVAAMSK.D			Oxidation (M)
1427.5736	1428.5853	3	288	301	R.DGMDNQGGYGSVGR.M			Oxidation (M)

Spot 47 (B14 20080606) – mixture of: Tubulin beta-2C chain (Tubb2c), Tubulin beta-3 chain (Tubb3), Tubulin beta-5 chain (Tubb5), Tubulin beta-2B chain (Tubb2b) and Tubulin beta-2A chain (Tubb2a)

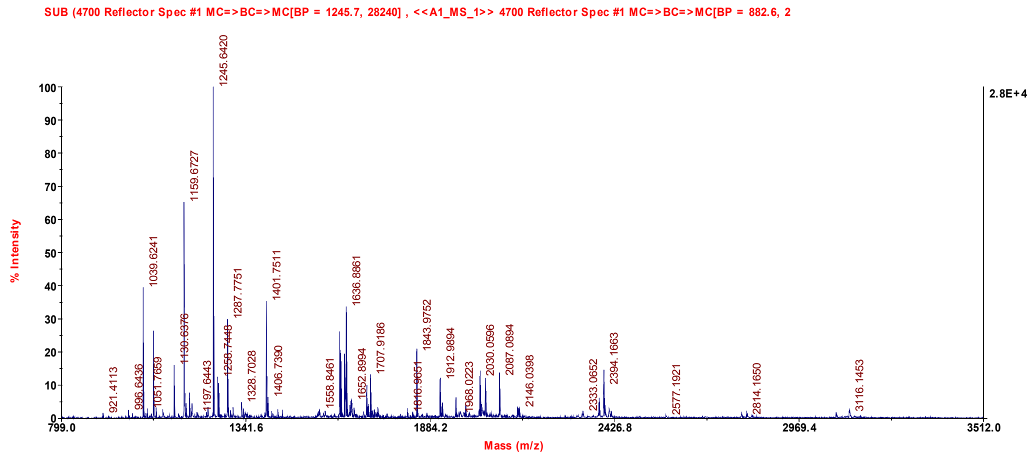

Spot 47 (20080606-B14) Tubb2c
MS+MS/MS

Calculated	Observed	+/-ppm	start	end	Sequence	Ion Score	C.I.%	Modification
1039.5935	1039.6211	27	310	318	YLTVAAVFR	44	99.995	
1039.5935	1039.6211	27	310	318	YLTVAAVFR			
1130.5953	1130.6346	35	242	251	FPGQLNADLR			
1159.6292	1159.6686	34	253	262	LAVNMVPFPR			Oxidation (M)
1159.6292	1159.6686	34	253	262	LAVNMVPFPR	8	78.035	Oxidation (M)
1245.5933	1245.6399	37	381	390	ISEQFTAMFR			Oxidation (M)
1245.5933	1245.6399	37	381	390	ISEQFTAMFR	3	29.736	Oxidation (M)
1258.6902	1258.7437	43	242	252	FPGQLNADLRK			
1287.7241	1287.7717	37	252	262	KLAVNMVPFPR			Oxidation (M)
1287.7241	1287.7717	37	252	262	KLAVNMVPFPR			Oxidation (M)
1328.6481	1328.7032	41	47	58	INVYYNEATGGK			
1401.6943	1401.7499	40	381	391	ISEQFTAMFRR			Oxidation (M)
1401.6943	1401.7499	40	380	390	RISEQFTAMFR			Oxidation (M)
1617.8152	1617.8699	34	63	77	AVLVDLEPGTMDSVR			Oxidation (M)
1617.8152	1617.8699	34	63	77	AVLVDLEPGTMDSVR	17	97.383	Oxidation (M)
1620.8356	1620.8877	32	263	276	LHFFMPGFAPLTSR			
1636.8304	1636.8855	34	263	276	LHFFMPGFAPLTSR			Oxidation (M)
1636.8304	1636.8855	34	263	276	LHFFMPGFAPLTSR			Oxidation (M)
1696.8329	1696.8901	34	337	350	NSSYFVEWIPNNVK			
1707.8622	1707.9181	33	283	297	ALTVPELTQQMFDAK			Oxidation (M)
1707.8622	1707.9181	33	283	297	ALTVPELTQQMFDAK	3	38.378	Oxidation (M)
1843.9337	1843.9734	22	47	62	INVYYNEATGGKYVPR			
1843.9337	1843.9734	22	47	62	INVYYNEATGGKYVPR	32	99.92	
1958.9817	1959.0077	13	104	121	GHYTEGAELVDSVLDVVR			
2030.0375	2030.061	12	363	380	MSATFIGNSTAIQELFKR	23	99.31	Oxidation (M)
2030.0375	2030.061	12	363	380	MSATFIGNSTAIQELFKR			
2087.0767	2087.0896	6	104	122	GHYTEGAELVDSVLDVVRK	20	98.633	
2087.0767	2087.0896	6	104	122	GHYTEGAELVDSVLDVVRK			

Spot 47 (20080606-B14) Tubb2c
PMF/MALDI

Calculated	Observed	+/-ppm	start	end	Sequence	Ion Score	C.I.%	Modification
1327.6408	1328.7028	41	47	58	R.INVYYNEATGGK.Y			
1842.9264	1843.9753	23	47	62	R.INVYYNEATGGKYVPR.A			
1600.8131	1601.8639	27	63	77	R.AVLVDLEPGTMDSVR.S			
1616.808	1617.8704	34	63	77	R.AVLVDLEPGTMDSVR.S			Oxidation (M)
1957.9745	1959.0085	14	104	121	K.GHYTEGAELVDSVLDVVR.K			
2086.0695	2087.0894	6	104	122	K.GHYTEGAELVDSVLDVVRK.E			
1076.525	1077.5687	34	155	162	K.IREEYPDR.I			
1129.588	1130.6376	37	242	251	R.FPGQLNADLR.K			
1257.683	1258.7448	43	242	252	R.FPGQLNADLRK.L			
1286.7169	1287.7751	40	252	262	R.KLAVNMVPFPR.L			Oxidation (M)
1142.627	1143.6765	37	253	262	K.LAVNMVPFPR.L			
1158.6219	1159.6727	38	253	262	K.LAVNMVPFPR.L			Oxidation (M)
1619.8283	1620.8884	33	263	276	R.LHFFMPGFAPLTSR.G			
1635.8232	1636.8861	34	263	276	R.LHFFMPGFAPLTSR.G			Oxidation (M)
1706.8549	1707.9186	33	283	297	R.ALTVPELTQQMFDAK.N			Oxidation (M)
1038.5862	1039.6241	30	310	318	R.YLTVAAVFR.G			
1970.8747	1971.9418	30	321	336	R.MSMKEVDEQMLNVQNK.N			3 Oxidation (M)
1695.8257	1696.8905	34	337	350	K.NSSYFVEWIPNNVK.T			
1872.9291	1873.9821	24	363	379	K.MSATFIGNSTAIQELFK.R			Oxidation (M)
2029.0302	2030.0596	11	363	380	K.MSATFIGNSTAIQELFKR.I			Oxidation (M)
1228.591	1229.647	40	381	390	R.ISEQFTAMFR.R			
1244.586	1245.642	39	381	390	R.ISEQFTAMFR.R			Oxidation (M)
1400.6871	1401.7511	41	381	391	R.ISEQFTAMFRR.K			Oxidation (M)

**Spot 47 (20080606-B14) Tubb3**								
**MS+MS/MS**								
Calculated	Observed	+/-ppm	start	end	Sequence	Ion Score	C.I.%	Modification
1069.604	1069.6357	30	310	318	YLTVATVFR			
1069.604	1069.6357	30	310	318	YLTVATVFR	34	99.945	
1130.5953	1130.6346	35	242	251	FPGQLNADLR			
1159.6292	1159.6686	34	253	262	LAVNMVPFPR			Oxidation (M)
1159.6292	1159.6686	34	253	262	LAVNMVPFPR	8	78.035	Oxidation (M)
1245.5933	1245.6399	37	381	390	ISEQFTAMFR			Oxidation (M)
1245.5933	1245.6399	37	381	390	ISEQFTAMFR	3	29.736	Oxidation (M)
1258.6902	1258.7437	43	242	252	FPGQLNADLRK			
1287.7241	1287.7717	37	252	262	KLAVNMVPFPR			Oxidation (M)
1287.7241	1287.7717	37	252	262	KLAVNMVPFPR			Oxidation (M)
1401.6943	1401.7499	40	381	391	ISEQFTAMFRR			Oxidation (M)
1401.6943	1401.7499	40	380	390	RISEQFTAMFR			Oxidation (M)
1406.6831	1406.7379	39	325	336	EVDEQMLAIQSK			Oxidation (M)
1620.8354	1620.8877	32	263	276	LHFFMPGFAPLTAR			Oxidation (M)
1631.8309	1631.8868	34	63	77	AILVDLEPGTMDSVR			Oxidation (M)
1696.8329	1696.8901	34	337	350	NSSYFVEWIPNNVK			
1707.8622	1707.9181	33	283	297	ALTVPELTQQMFDAK			Oxidation (M)
1707.8622	1707.9181	33	283	297	ALTVPELTQQMFDAK	3	38.378	Oxidation (M)
1912.9552	1912.9902	18	47	62	ISVYYNEASSHKYVPR			
1912.9552	1912.9902	18	47	62	ISVYYNEASSHKYVPR	16	97.023	
1958.9817	1959.0077	13	104	121	GHYTEGAELVDSVLDVVR			
2030.0375	2030.061	12	363	380	MSSTFIGNSTAIQELFKR	23	99.31	
2030.0375	2030.061	12	363	380	MSSTFIGNSTAIQELFKR			
2046.0325	2046.0509	9	363	380	MSSTFIGNSTAIQELFKR	30	99.876	Oxidation (M)
2046.0325	2046.0509	9	363	380	MSSTFIGNSTAIQELFKR			Oxidation (M)
2087.0767	2087.0896	6	104	122	GHYTEGAELVDSVLDVVRK	20	98.633	
2087.0767	2087.0896	6	104	122	GHYTEGAELVDSVLDVVRK			

**Spot 47 (20080606-B14) Tubb3**								
**PMF/MALDI**								
Calculated	Observed	+/-ppm	start	end	Sequence	Ion Score	C.I.%	Modification
1396.6623	1397.7332	46	47	58	R.ISVYYNEASSHK.Y			
1911.9479	1912.9895	18	47	62	R.ISVYYNEASSHKYVPR.A			
1614.8287	1615.882	28	63	77	R.AILVDLEPGTMDSVR.S			
1630.8236	1631.8865	34	63	77	R.AILVDLEPGTMDSVR.S			Oxidation (M)
1957.9745	1959.0085	14	104	121	K.GHYTEGAELVDSVLDVVR.K			
2086.0695	2087.0894	6	104	122	K.GHYTEGAELVDSVLDVVRK.E			
1129.588	1130.6376	37	242	251	R.FPGQLNADLR.K			
1257.683	1258.7448	43	242	252	R.FPGQLNADLRK.L			
1286.7169	1287.7751	40	252	262	R.KLAVNMVPFPR.L			Oxidation (M)
1142.627	1143.6765	37	253	262	K.LAVNMVPFPR.L			
1158.6219	1159.6727	38	253	262	K.LAVNMVPFPR.L			Oxidation (M)
1619.8283	1620.8884	33	263	276	R.LHFFMPGFAPLTAR.G			Oxidation (M)
1706.8549	1707.9186	33	283	297	R.ALTVPELTQQMFDAK.N			Oxidation (M)
1068.5968	1069.6383	32	310	318	R.YLTVATVFR.G			
1405.6759	1406.739	40	325	336	K.EVDEQMLAIQSK.N			Oxidation (M)
1695.8257	1696.8905	34	337	350	K.NSSYFVEWIPNNVK.V			
1872.9291	1873.9821	24	363	379	K.MSSTFIGNSTAIQELFK.R			
2029.0302	2030.0596	11	363	380	K.MSSTFIGNSTAIQELFKR.I			
2045.0252	2046.0491	8	363	380	K.MSSTFIGNSTAIQELFKR.I			Oxidation (M)
1228.591	1229.647	40	381	390	R.ISEQFTAMFR.R			
1244.586	1245.642	39	381	390	R.ISEQFTAMFR.R			Oxidation (M)
1400.6871	1401.7511	41	381	391	R.ISEQFTAMFRR.K			Oxidation (M)

**Spot 47 (20080606-B14) Tubb5**								
**MS+MS/MS**								
Calculated	Observed	+/-ppm	start	end	Sequence	Ion Score	C.I.%	Modification
1039.5935	1039.6211	27	310	310	YLTVAAVFR	44	99.995	
1039.5935	1039.6211	27	310	310	YLTVAAVFR			
1130.5953	1130.6346	35	242	242	FPGQLNADLR			
1159.6292	1159.6686	34	253	253	LAVNMVPFPR			Oxidation (M)
1159.6292	1159.6686	34	253	253	LAVNMVPFPR	8	78.035	Oxidation (M)
1245.5933	1245.6399	37	381	381	ISEQFTAMFR			Oxidation (M)
1245.5933	1245.6399	37	381	390	ISEQFTAMFR	3	29.736	Oxidation (M)
1258.6902	1258.7437	43	242	252	FPGQLNADLRK			
1287.7241	1287.7717	37	252	262	KLAVNMVPFPR			Oxidation (M)
1287.7241	1287.7717	37	252	262	KLAVNMVPFPR			Oxidation (M)
1401.6943	1401.7499	40	381	391	ISEQFTAMFRR			Oxidation (M)
1401.6943	1401.7499	40	380	390	RISEQFTAMFR			Oxidation (M)
1620.8356	1620.8877	32	263	276	LHFFMPGFAPLTSR			
1631.8309	1631.8868	34	63	77	AILVDLEPGTMDSVR			Oxidation (M)
1636.8304	1636.8855	34	263	276	LHFFMPGFAPLTSR			Oxidation (M)
1636.8304	1636.8855	34	263	276	LHFFMPGFAPLTSR			Oxidation (M)
1659.8951	1659.9377	26	283	297	ALTVPELTQQVFDAK			
1696.8329	1696.8901	34	337	350	NSSYFVEWIPNNVK			
1958.9817	1959.0077	13	104	121	GHYTEGAELVDSVLDVVR			
2087.0767	2087.0896	6	104	122	GHYTEGAELVDSVLDVVRK	20	98.633	
2087.0767	2087.0896	6	104	122	GHYTEGAELVDSVLDVVRK			

Spot 47 (20080606-B14) Tubb5
PMF/MALDI

Calculated	Observed	+/-ppm	start	end	Sequence	Ion Score	C.I.%	Modification
1815.9155	1816.9652	23	47	62	R.ISVYYNEATGGKYVPR.A			
1614.8287	1615.882	28	63	77	R.AILVDLEPGTMDSVR.S			
1630.8236	1631.8865	34	63	77	R.AILVDLEPGTMDSVR.S			Oxidation (M)
1957.9745	1959.0085	14	104	121	K.GHYTEGAELVDSVLDVVR.K			
2086.0695	2087.0894	6	104	122	K.GHYTEGAELVDSVLDVVRK.E			
1076.525	1077.5687	34	155	162	K.IREEYPDR.I			
1129.588	1130.6376	37	242	251	R.FPGQLNADLR.K			
1257.683	1258.7448	43	242	252	R.FPGQLNADLRK.L			
1286.7169	1287.7751	40	252	262	R.KLAVNMVPFPR.L			Oxidation (M)
1142.627	1143.6765	37	253	262	K.LAVNMVPFPR.L			
1158.6219	1159.6727	38	253	262	K.LAVNMVPFPR.L			Oxidation (M)
1619.8283	1620.8884	33	263	276	R.LHFFMPGFAPLTSR.G			
1635.8232	1636.8861	34	263	276	R.LHFFMPGFAPLTSR.G			Oxidation (M)
1658.8879	1659.9366	25	283	297	R.ALTVPELTQQVFDAK.N			
1038.5862	1039.6241	30	310	318	R.YLTVAAVFR.G			
1970.8747	1971.9418	30	321	336	R.MSMKEVDEQMLNVQNK.N			3 Oxidation (M)
1695.8257	1696.8905	34	337	350	K.NSSYFVEWIPNNVK.T			
2025.0717	2026.1169	19	363	380	K.MAVTFIGNSTAIQELFKR.I			
2041.0666	2042.0702	-2	363	380	K.MAVTFIGNSTAIQELFKR.I			Oxidation (M)
1228.591	1229.647	40	381	390	R.ISEQFTAMFR.R			
1244.586	1245.642	39	381	390	R.ISEQFTAMFR.R			Oxidation (M)
1400.6871	1401.7511	41	381	391	R.ISEQFTAMFRR.K			Oxidation (M)

Spot 47 (20080606-B14) Tubb2b
MS+MS/MS

Calculated	Observed	+/-ppm	start	end	Sequence	Ion Score	C.I.%	Modification
1130.5953	1130.6346	35	242	251	FPGQLNADLR			
1159.6292	1159.6686	34	253	262	LAVNMVPFPR			Oxidation (M)
1159.6292	1159.6686	34	253	262	LAVNMVPFPR	8	78.035	Oxidation (M)
1245.5933	1245.6399	37	381	390	ISEQFTAMFR			Oxidation (M)
1245.5933	1245.6399	37	381	390	ISEQFTAMFR	3	29.736	Oxidation (M)
1258.6902	1258.7437	43	242	252	FPGQLNADLRK			
1287.7241	1287.7717	37	252	262	KLAVNMVPFPR			Oxidation (M)
1287.7241	1287.7717	37	252	262	KLAVNMVPFPR			Oxidation (M)
1401.6943	1401.7499	40	381	391	ISEQFTAMFRR			Oxidation (M)
1401.6943	1401.7499	40	380	390	RISEQFTAMFR			Oxidation (M)
1620.8356	1620.8877	32	263	276	LHFFMPGFAPLTSR			
1631.8309	1631.8868	34	63	77	AILVDLEPGTMDSVR			Oxidation (M)
1636.8304	1636.8855	34	263	276	LHFFMPGFAPLTSR			Oxidation (M)
1636.8304	1636.8855	34	263	276	LHFFMPGFAPLTSR			Oxidation (M)
1696.8329	1696.8901	34	337	350	NSSYFVEWIPNNVK			
1707.8622	1707.9181	33	283	297	ALTVPELTQQMFDSK			
1707.8622	1707.9181	33	283	297	ALTVPELTQQMFDSK	3	38.378	
1958.9817	1959.0077	13	104	121	GHYTEGAELVDSVLDVVR			
2030.0375	2030.061	12	363	380	MSATFIGNSTAIQELFKR	23	99.31	Oxidation (M)
2030.0375	2030.061	12	363	380	MSATFIGNSTAIQELFKR			Oxidation (M)
2087.0767	2087.0896	6	104	122	GHYTEGAELVDSVLDVVRK	20	98.633	
2087.0767	2087.0896	6	104	122	GHYTEGAELVDSVLDVVRK			

Spot 47 (20080606-B14) Tubb2b
PMF/MALDI

Calculated	Observed	+/-ppm	start	end	Sequence	Ion Score	C.I.%	Modification
1614.8287	1615.882	28	63	77	R.AILVDLEPGTMDSVR.S			
1630.8236	1631.8865	34	63	77	R.AILVDLEPGTMDSVR.S			Oxidation (M)
1957.9745	1959.0085	14	104	121	K.GHYTEGAELVDSVLDVVR.K			
2086.0695	2087.0894	6	104	122	K.GHYTEGAELVDSVLDVVRK.E			
1076.525	1077.5687	34	155	162	K.IREEYPDR.I			
1129.588	1130.6376	37	242	251	R.FPGQLNADLR.K			
1257.683	1258.7448	43	242	252	R.FPGQLNADLRK.L			
1286.7169	1287.7751	40	252	262	R.KLAVNMVPFPR.L			Oxidation (M)
1142.627	1143.6765	37	253	262	K.LAVNMVPFPR.L			
1158.6219	1159.6727	38	253	262	K.LAVNMVPFPR.L			Oxidation (M)
1619.8283	1620.8884	33	263	276	R.LHFFMPGFAPLTSR.G			
1635.8232	1636.8861	34	263	276	R.LHFFMPGFAPLTSR.G			Oxidation (M)
1706.8549	1707.9186	33	283	297	R.ALTVPELTQQMFDSK.N			
1722.8498	1723.9098	31	283	297	R.ALTVPELTQQMFDSK.N			Oxidation (M)
1970.8747	1971.9418	30	321	336	R.MSMKEVDEQMLNVQNK.N			3 Oxidation (M)
1695.8257	1696.8905	34	337	350	K.NSSYFVEWIPNNVK.T			
1872.9291	1873.9821	24	363	379	K.MSATFIGNSTAIQELFK.R			Oxidation (M)
2029.0302	2030.0596	11	363	380	K.MSATFIGNSTAIQELFKR.I			Oxidation (M)
1228.591	1229.647	40	381	390	R.ISEQFTAMFR.R			
1244.586	1245.642	39	381	390	R.ISEQFTAMFR.R			Oxidation (M)
1400.6871	1401.7511	41	381	391	R.ISEQFTAMFRR.K			Oxidation (M)

Spot 47 (20080606-B14) Tubb2a
PMF/MALDI

Calculated	Observed	+/-ppm	start	end	Sequence	Ion Score	C.I.%	Modification
1614.8287	1615.882	28	63	77	R.AILVDLEPGTMDSVR.S			
1630.8236	1631.8865	34	63	77	R.AILVDLEPGTMDSVR.S			Oxidation (M)
1957.9745	1959.0085	14	104	121	K.GHYTEGAELVDSVLDVVR.K			
2086.0695	2087.0894	6	104	122	K.GHYTEGAELVDSVLDVVRK.E			
1076.525	1077.5687	34	155	162	K.IREEYPDR.I			
1129.588	1130.6376	37	242	251	R.FPGQLNADLR.K			
1257.683	1258.7448	43	242	252	R.FPGQLNADLRK.L			
1286.7169	1287.7751	40	252	262	R.KLAVNMVPFPR.L			Oxidation (M)
1142.627	1143.6765	37	253	262	K.LAVNMVPFPR.L			
1158.6219	1159.6727	38	253	262	K.LAVNMVPFPR.L			Oxidation (M)
1619.8283	1620.8884	33	263	276	R.LHFFMPGFAPLTSR.G			
1635.8232	1636.8861	34	263	276	R.LHFFMPGFAPLTSR.G			Oxidation (M)
1706.8549	1707.9186	33	283	297	R.ALTVPELTQQMFDSK.N			
1722.8498	1723.9098	31	283	297	R.ALTVPELTQQMFDSK.N			Oxidation (M)
1970.8747	1971.9418	30	321	336	R.MSMKEVDEQMLNVQNK.N			3 Oxidation (M)
1695.8257	1696.8905	34	337	350	K.NSSYFVEWIPNNVK.T			
1872.9291	1873.9821	24	363	379	K.MSATFIGNSTAIQELFK.R			Oxidation (M)
2029.0302	2030.0596	11	363	380	K.MSATFIGNSTAIQELFKR.I			Oxidation (M)
1228.591	1229.647	40	381	390	R.ISEQFTAMFR.R			
1244.586	1245.642	39	381	390	R.ISEQFTAMFR.R			Oxidation (M)
1400.6871	1401.7511	41	381	391	R.ISEQFTAMFRR.K			Oxidation (M)

Spot 48 (D6 20080606) - Lactate dehydrogenase B (Ldhb)

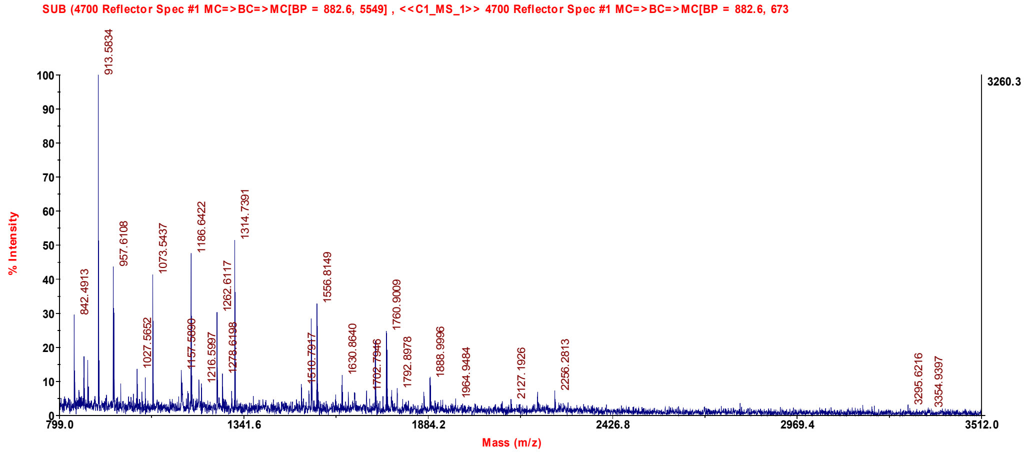

Spot 48 (20080606-D6) Ldhb								
PMF/MALDI								
Calculated	Observed	+/-ppm	start	end	Sequence	Ion Score	C.I.%	Modification
1665.8573	1666.8582	-4	8	23	K.LIAPVADDETAVPNNK.I			
1628.8509	1629.8608	2	44	58	K.SLADELALVDVLEDK.L			
1870.0299	1871.0557	10	44	60	K.SLADELALVDVLEDKLK.G			
2204.097	2205.1138	4	59	77	K.LKGEMMDLQHGSLFLQTPK.I			2 Oxidation (M)
1509.7675	1510.7917	11	78	91	K.IVADKDYSVTANSK.I			
912.5757	913.5834	1	92	100	K.IVVVTAGVR.Q			
1726.9326	1727.9368	-2	92	107	K.IVVVTAGVRQQEGESR.L			
1555.8066	1556.8149	1	101	113	R.QQEGESRLNLVQR.N			
956.6059	957.6108	-2	120	127	K.FIIPQIVK.Y			
1072.5375	1073.5437	-1	171	178	R.FRYLMAEK.L			Oxidation (M)
1761.8971	1762.9047	0	230	244	K.EVHKMVVDSAYEVIK.L			Oxidation (M)
1026.5532	1027.5652	5	271	279	R.IHPVSTMVK.G			Oxidation (M)
958.5447	959.5489	-3	300	308	R.GLTSVINQK.L			
2126.1695	2127.1926	7	300	318	R.GLTSVINQKLKDDEVAQLR.K			
1185.6353	1186.6422	0	309	318	K.LKDDEVAQLR.K			
1313.7303	1314.7391	1	309	319	K.LKDDEVAQLRK.S			
1175.5822	1176.5886	-1	320	329	K.SADTLWDIQK.D			
1531.7882	1532.7867	-6	320	332	K.SADTLWDIQKDLK.D			
1759.8992	1760.9009	-3	320	334	K.SADTLWDIQKDLKDL.-			

Spot 54 (E9 20070719) – Protein DJ-1 (Park 7, Cap1)

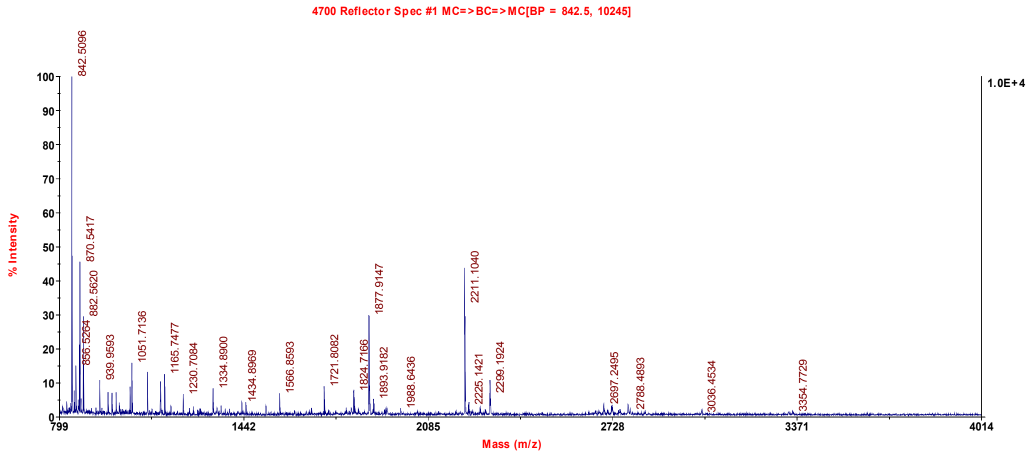

Spot 54 (20070719-E9) Park7/Cap1								
PMF/MALDI								
Calculated	Observed	+/-ppm	start	end	Sequence	Ion Score	C.I.%	Modification
1720.8012	1721.8082	0	13	27	K.GAEEMETVIPVDIMR.R			2 Oxidation (M)
1876.9023	1877.9147	3	13	28	K.GAEEMETVIPVDIMRR.A			2 Oxidation (M)
2725.4287	2726.3196	-43	63	89	K.TQGPYDVVVLPGGNLGAQNLSESALVK.E			
1157.604	1158.6464	30	90	98	K.EILKEQENR.K			
1285.699	1286.7001	-5	90	99	K.EILKEQENRK.G			
851.4865	852.5152	25	123	130	K.VTSHPLAK.D			
1229.6979	1230.7084	3	146	156	R.VEKDGLILTSR.G			
873.492	874.5032	4	149	156	K.DGLILTSR.G			
1892.9884	1893.9182	-41	157	175	R.GPGTSFEFALAIVEALSGK.D			
2695.3527	2696.2808	-29	157	182	R.GPGTSFEFALAIVEALSGKDMANQVK.A			Oxidation (M)

Spot 57 (A22 20080606) - Inner membrane protein, mitochondrial, isoform CRA_a (Immt)

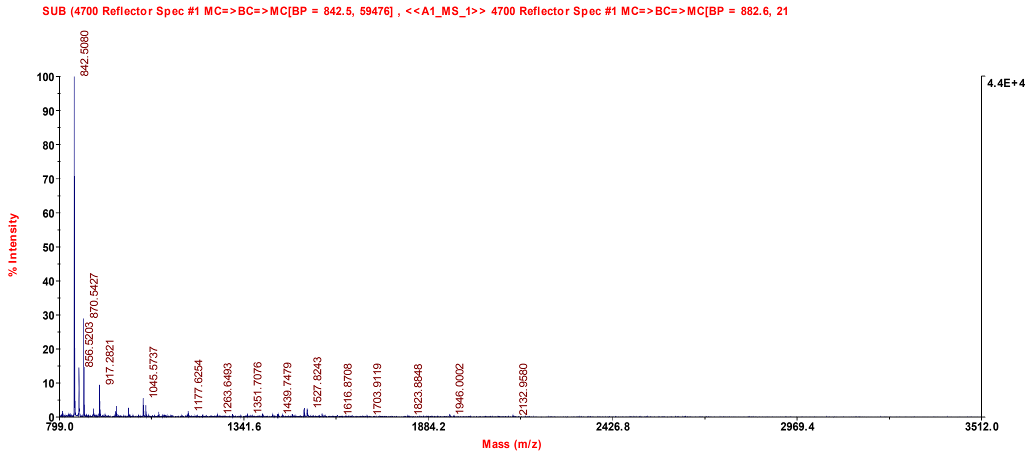

Spot 57 (20080606-A22) Immt (isoform CRA_a)								
PMF/MALDI								
Calculated	Observed	+/-ppm	start	end	Sequence	Ion Score	C.I.%	Modification
1394.6929	1395.7205	15	73	84	R.ESVEKTIPYSDK.L			
966.5862	967.5955	2	102	110	K.KPIQSGPLK.I			
1306.6075	1307.6754	46	196	206	K.QEQVEMESLAK.S			Oxidation (M)
1517.7685	1518.7817	4	207	220	K.SLEDALNQTATVTR.Q			
1001.5505	1002.559	1	260	268	R.TVEGALKER.R			
1526.8205	1527.8243	-2	338	350	K.VVSQYHELVVQAR.D			
1570.7879	1571.833	24	356	369	K.ELDSITPDITPGWK.G			
1454.7841	1455.8057	10	391	402	R.IDQLNRELAQQK.A			
963.5138	964.5222	1	422	430	K.RAFDSAVAK.A			
807.4127	808.4177	-3	423	430	R.AFDSAVAK.A			
1442.7266	1443.8011	47	496	506	K.LSEQELEFHRR.S			
2131.948	2132.958	1	507	524	R.SQEQMDNFTLDINTAYAR.L			Oxidation (M)
1822.8809	1823.8848	-2	527	543	R.GIEQAVQSHAVAEEEAR.K			
1944.9826	1946.0002	5	561	579	K.TSSAEMPTIPLGSAVEAIR.V			Oxidation (M)
1052.5138	1053.5222	1	603	611	R.GVYSEETLR.A			
1176.6251	1177.6254	-6	688	697	K.FVNQLKGESR.R			

Spot 59 (D14 20080606) - Protein phosphatase 2A, regulatory subunit B (PR 53) (Ppp2r4)

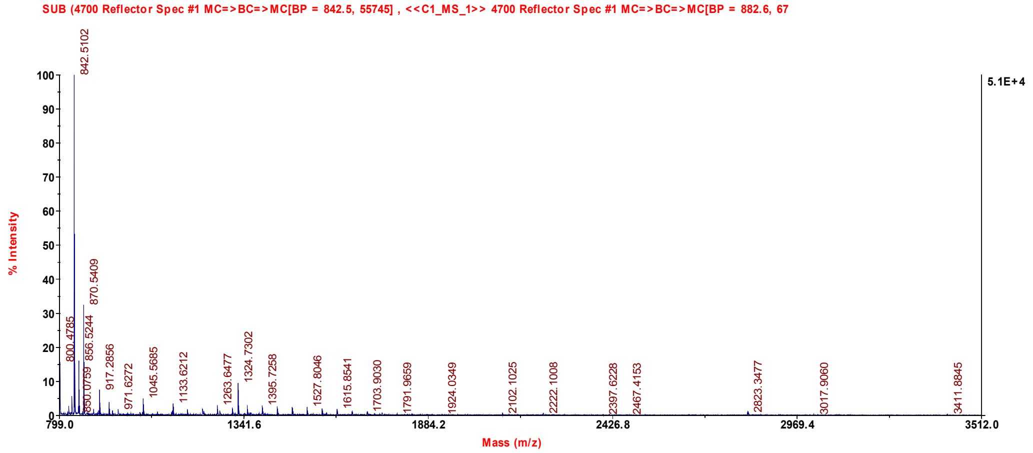

Spot 59 (20080606-D14) Ppp2r4								
PMF/MALDI								
Calculated	Observed	+/-ppm	start	end	Sequence	Ion Score	C.I.%	Modification
2238.1056	2239.0869	-12	7	27	R.QPPPDSSEETPSATPTFIIPK.K			
1269.6387	1270.6415	-4	28	38	K.KEIHTVPDMGK.W			Oxidation (M)
1141.5438	1142.5446	-6	29	38	K.EIHTVPDMGK.W			Oxidation (M)
2101.0732	2102.0969	8	42	60	R.SQAYADYIGFILTLNEGVK.G			
913.4909	914.4963	-2	63	69	K.KLTFDYK.V			
1127.655	1128.6655	3	77	86	K.LVALLDTLDR.W			
854.4399	855.4548	9	100	106	R.FGNKAYR.T			
832.4039	833.4106	-1	141	148	K.EAVGNSTR.I			
1132.6128	1133.6224	2	174	183	R.VDDQVAIVFK.V			
1326.6754	1327.7374	41	184	193	K.VFDRYLEVMR.K			
825.4055	826.4168	5	188	193	R.YLEVMR.K			Oxidation (M)
953.5004	954.5128	5	188	194	R.YLEVMRK.L			Oxidation (M)
1220.6699	1221.6624	-12	287	296	K.VNQGLIRMYK.A			
1014.5651	1015.5662	-6	303	310	K.FPVIQHFK.F			
1323.7187	1324.7302	3	311	323	K.FGSLLPIHPVTSG.-			

Spot 62 (E4 20070719) – Glutathione S-transferase Mu 5 (Gstm5)

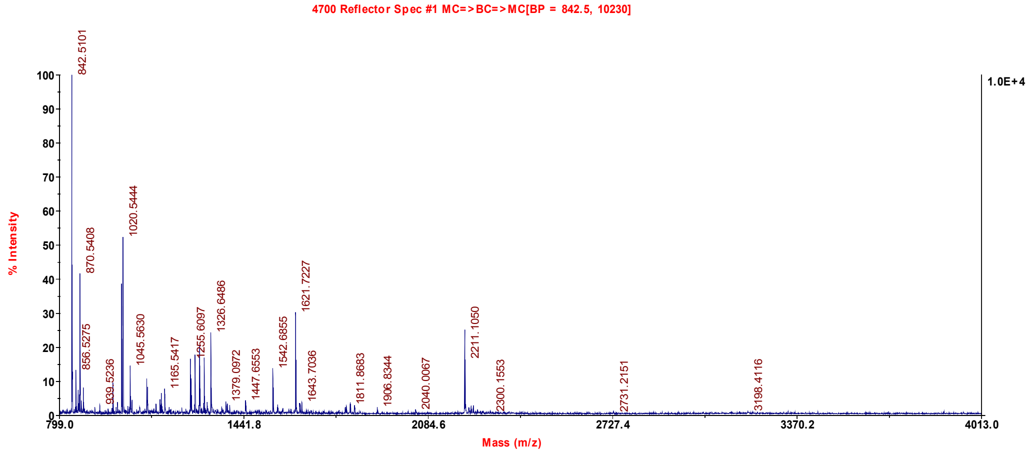

Spot 62 (20070719-E4) Gstm5								
PMF/MALDI								
Calculated	Observed	+/-ppm	start	end	Sequence	Ion Score	C.I.%	Modification
1254.6067	1255.6097	-3	6	15	K.SMVLGYWDIR.G			Oxidation (M)
1604.728	1605.7363	1	23	35	R.MLLEFTDTSYEEK.Q			
1620.7229	1621.7227	-5	23	35	R.MLLEFTDTSYEEK.Q			Oxidation (M)
874.4549	875.4564	-7	48	54	R.SQWLDVK.F			
1256.72	1257.7225	-4	72	82	K.NKITQSNAILR.Y			
1014.5822	1015.5909	1	74	82	K.ITQSNAILR.Y			
1634.6664	1635.7501	47	88	100	K.HNMCGDTEEEKIR.V			Oxidation (M)
1810.8706	1811.8683	-5	99	112	K.IRVDIMENQIMDFR.M			2 Oxidation (M)
1541.6854	1542.6855	-5	101	112	R.VDIMENQIMDFR.M			2 Oxidation (M)
938.5226	939.5236	-7	141	148	K.QFSLFLGK.F			
984.4705	985.4709	-7	149	156	K.FTWFAGEK.L			
1019.54	1020.5444	-3	198	206	K.IAAFLQSDR.C			
1077.5311	1078.5499	11	210	218	K.MPINNKMAK.W			2 Oxidation (M)

Spot 63 (D8 20080815) – Leucine zipper transcription factor-like protein 1 (Lztfl1)

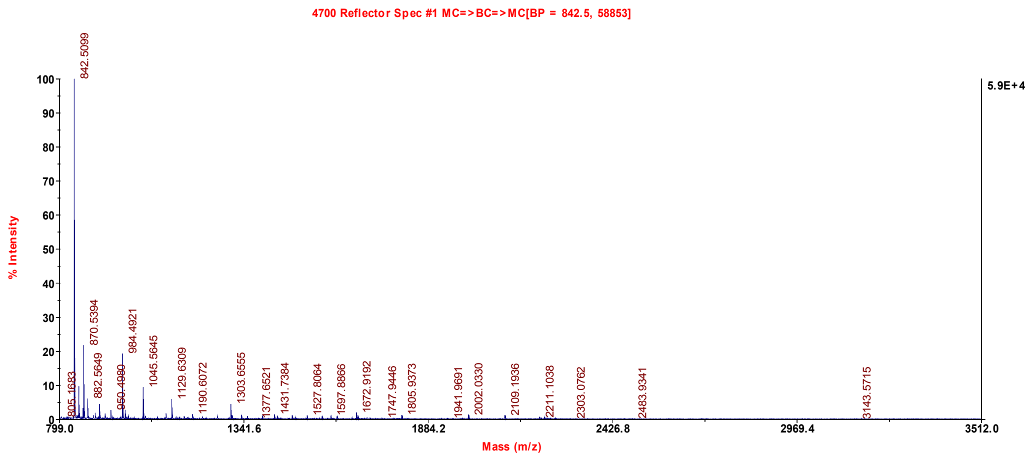

Spot 63 (20080815-D8) Lztfl1
MS+MS/MS

Calculated	Observed	+/-ppm	start	end	Sequence	Ion Score	C.I.%	Modification
950.4941	950.4993	5	86	93	QLFSQAEK			
950.4941	950.4993	5	86	93	QLFSQAEK			
954.4414	954.4524	12	292	299	LAKYESED			
984.4897	984.4936	4	265	272	FQQTAAYR			
984.4897	984.4936	4	265	272	FQQTAAYR	11	89.653	
992.5444	992.5486	4	273	280	NMKEILTK	0	0	Oxidation (M)
992.5444	992.5486	4	273	280	NMKEILTK			
1001.5374	1001.5446	7	282	289	NDQIKDLR			
1045.5524	1045.5641	11	235	244	SLEENLAAAK			
1112.5847	1112.5924	7	264	272	KFQQTAAYR			
1129.6212	1129.6324	10	157	165	LQQENEKLK			
1129.6212	1129.6324	10	157	165	LQQENEKLK	14	94.79	
1190.6012	1190.609	7	225	234	TLNDKTENQK			
1190.6012	1190.609	7	225	234	TLNDKTENQK			
1303.6488	1303.6569	6	98	108	LQTDVSELENR			
1303.6488	1303.6569	6	98	108	LQTDVSELENR	39	99.983	
1334.6838	1334.6913	6	109	119	ELLEQVAEFEK			
1431.7325	1431.7405	6	170	182	TIETQAVNALDEK			
1597.8795	1597.8882	5	138	152	LVPINEGGTTELLNK			
1615.8174	1615.849	20	205	219	AQDLDDLENTVAALK			
1672.9116	1672.9194	5	168	182	LKTIETQAVNALDEK	18	98.015	
1672.9116	1672.9194	5	168	182	LKTIETQAVNALDEK			
1677.8363	1677.8435	4	250	263	VQEQLSMAEKELEK			Oxidation (M)
2002.024	2002.0345	5	188	204	VLQDLQLDQGNQQDFIK			
2002.024	2002.0345	5	188	204	VLQDLQLDQGNQQDFIK	1	0	
2109.1914	2109.1963	2	138	156	LVPINEGGTTELLNKEILR			
2109.1914	2109.1963	2	138	156	LVPINEGGTTELLNKEILR	30	99.87	

Spot 63 (20080815-D8) Lztfl1
PMF/MALDI

Calculated	Observed	+/-ppm	start	end	Sequence	Ion Score	C.I.%	Modification
1825.888	1826.844	-28	29	43	R.LKTVDSCFQDLKESR.L			
949.4869	950.498	4	86	93	R.QLFSQAEK.W			
1302.6415	1303.6555	5	98	108	K.LQTDVSELENR.E			
1333.6765	1334.6891	4	109	119	R.ELLEQVAEFEK.A			
1596.8723	1597.8866	4	138	152	K.LVPINEGGTTELLNK.E			
2108.1841	2109.1936	1	138	156	K.LVPINEGGTTELLNKEILR.L			
1671.9043	1672.9192	5	168	182	R.LKTIETQAVNALDEK.S			
1430.7253	1431.7384	4	170	182	K.TIETQAVNALDEK.S			
2001.0167	2002.033	4	188	204	R.VLQDLQLDQGNQQDFIK.A			
1614.8101	1615.8475	19	205	219	K.AQDLDDLENTVAALK.S			
2234.1066	2235.1006	-6	205	224	K.AQDLDDLENTVAALKSEFQK.T			
1189.5939	1190.6072	5	225	234	K.TLNDKTENQK.S			
1044.5451	1045.5645	12	235	244	K.SLEENLAAAK.H			
1177.5649	1178.595	19	250	259	R.VQEQLSMAEK.E			Oxidation (M)
1676.8291	1677.8413	3	250	263	R.VQEQLSMAEKELEK.K			Oxidation (M)
1804.924	1805.9373	3	250	264	R.VQEQLSMAEKELEKK.F			Oxidation (M)
1111.5774	1112.5927	7	264	272	K.KFQQTAAYR.N			
1484.7558	1485.7847	15	264	275	K.KFQQTAAYRNMK.E			
983.4825	984.4921	2	265	272	K.FQQTAAYR.N			
1941.0142	1941.9691	-27	265	280	K.FQQTAAYRNMKEILTK.K			
1957.0091	1957.9229	-48	265	280	K.FQQTAAYRNMKEILTK.K			Oxidation (M)
991.5372	992.5472	3	273	280	R.NMKEILTK.K			Oxidation (M)
1000.5301	1001.5446	7	282	289	K.NDQIKDLR.K			
1128.6251	1129.6309	-1	282	290	K.NDQIKDLRK.R			
1109.5353	1110.55	7	291	299	K.RLAKYESED.-			
953.4342	954.4532	12	292	299	R.LAKYESED.-			

Spot 63 (A5 20080606) - Actin, cytoplasmic 2 (Actg1)

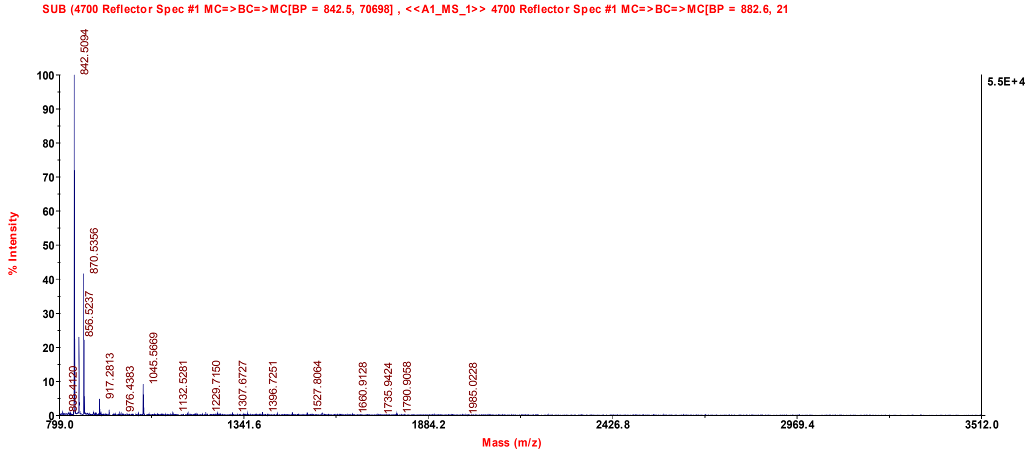

Spot 63 (20080606-A5) Actg1								
MS+MS/MS								
Calculated	Observed	+/-ppm	start	end	Sequence	Ion Score	C.I.%	Modification
923.5672	923.5668	0	329	336	IIAPPERK			
976.4482	976.4392	-9	19	28	AGFAGDDAPR			
976.4482	976.4392	-9	19	28	AGFAGDDAPR	26	99.611	
1132.527	1132.5323	5	197	206	GYSFTTTAER			
1177.6133	1177.6042	-8	316	326	EITALAPSTMK			Oxidation (M)
1203.5609	1203.5585	-2	40	50	HQGVMVGMGQK			2 Oxidation (M)
1790.8918	1790.9001	5	239	254	SYELPDGQVITIGNER			
1790.8918	1790.9001	5	239	254	SYELPDGQVITIGNER			

Spot 63 (A4 20080606) - Galactokinase 1 (Galk1)

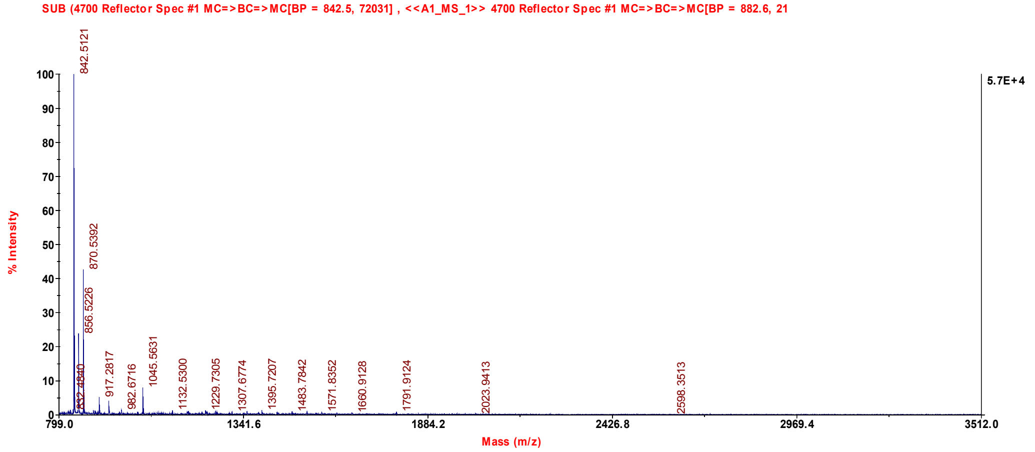

Spot 63 (20080606-A4) Galk1								
PMF/MALDI								
Calculated	Observed	+/-ppm	start	end	Sequence	Ion Score	C.I.%	Modification
1028.5502	1029.556	-1	9	17	R.VEELLAEAR.R			
2022.9357	2023.9412	-1	19	37	R.AFMEEFGAEPELAVSAPGR.V			Oxidation (M)
1233.6816	1234.6881	-1	69	80	R.TDGLVSLLTTSK.D			
1384.7449	1385.7507	-1	205	217	R.SLETSLVPLSDPK.L			
1228.7139	1229.7305	8	218	228	K.LTVLITNSNVR.H			
1258.6306	1259.632	-5	229	239	R.HSLASSEYPIR.R			
944.5039	945.512	1	288	297	R.TAQGAAALSR.G			

Spot 64 (E12 20070719) – Ferritin light chain 1 (Ftl1)

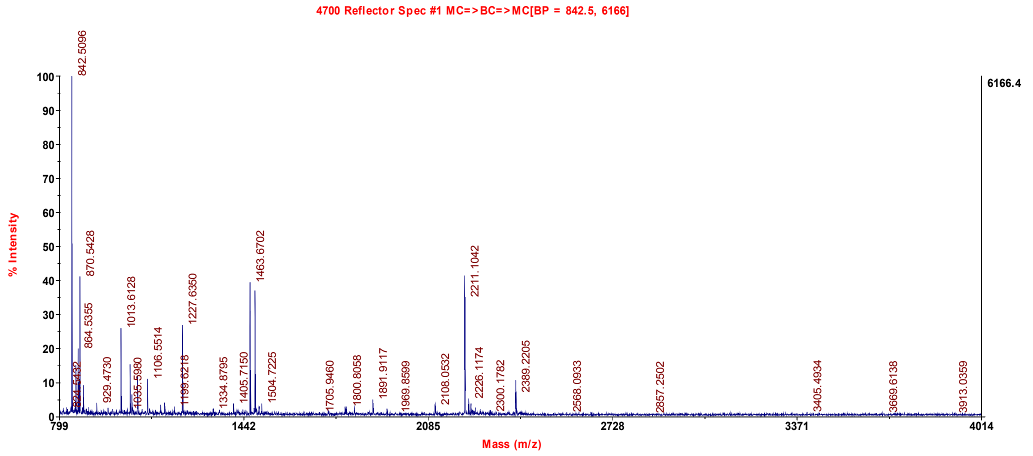

Spot 64 (20070719-E12) Ftl1								
MS+MS/MS								
Calculated	Observed	+/-ppm	start	end	Sequence	Ion Score	C.I.%	Modification
864.5413	864.5333	-9	19	25	LVNLHLR			
864.5413	864.5333	-9	19	25	LVNLHLR			
929.4911	929.4706	-22	68	75	LQNERGGR			
1013.6102	1013.6099	0	65	72	LLKLQNER			
1013.6102	1013.6099	0	65	72	LLKLQNER	19	98.681	
1071.5364	1071.5326	-4	144	152	MGNHLTNLR			Oxidation (M)
1071.5364	1071.5326	-4	144	152	MGNHLTNLR			Oxidation (M)
1199.6313	1199.6202	-9	143	152	KMGNHLTNLR			Oxidation (M)
1227.6375	1227.6312	-5	144	153	MGNHLTNLRR			Oxidation (M)
1227.6375	1227.6312	-5	144	153	MGNHLTNLRR	12	92.9	Oxidation (M)
1405.7242	1405.7131	-8	92	104	TLEAMEAALALEK			Oxidation (M)
1480.7026	1480.6964	-4	6	18	QNYSTEVEAAVNR			
1480.7026	1480.6964	-4	6	18	QNYSTEVEAAVNR	27	99.77	

Spot 64 (20070719-E12) Ftl1								
PMF/MALDI								
Calculated	Observed	+/-ppm	start	end	Sequence	Ion Score	C.I.%	Modification
1479.6954	1480.6982	-3	7	19	R.QNYSTEVEAAVNR.L			
863.5341	864.5355	-7	20	26	R.LVNLHLR.A			
1012.6029	1013.6128	3	66	73	R.LLKLQNER.G			
928.4839	929.473	-20	69	76	K.LQNERGGR.A			
1404.717	1405.715	-7	93	105	K.TLEAMEAALALEK.N			Oxidation (M)
1704.9271	1705.946	7	106	121	K.NLNQALLDLHALGSAR.T			
1198.6241	1199.6218	-8	144	153	K.KMGNHLTNLR.R			Oxidation (M)
1070.5291	1071.5363	0	145	153	K.MGNHLTNLR.R			Oxidation (M)
1226.6302	1227.635	-2	145	154	K.MGNHLTNLRR.V			Oxidation (M)
2388.2074	2389.2207	3	155	177	R.VAGPQPAQTGVAQASLGEYLFER.L			

Spot 68 (P1014-11) – Diablo homolog (Drosophila) (larger C-terminal fragment) (Diablo)

Spot 68 (P1014-11) Diablo											
LCMS/MS (Scaffold)											
Sample Name	Acc No.	Proteins(s) inferred	No. Peptides identified	Sequence Coverage	Position in sequence	Peptide Sequence	Precursor m/z	Charge observed	Calculated mass (neutral)	Mascot score	Mascot Expect value
P1014-11	Q5RK17	tr|Q5RK17|Diablo homolog (Drosophila) Tax_Id=10116 [Rattus norvegicus]	6	19.00%	206 - 215	(K)LAEVQTQELR(Q)	593.70	2	1185.39	89.0	5.48E-006
					61 - 73	(K)SEPQSLSNEALMR(R)	732.14	2	1462.27	52.2	2.29E-002
					218 - 237	(K)TQEASDEAADQEEEAYLRED(-)	767.06	3	2298.16	40.5	3.30E-001
					216 - 237	(R)QKTQEASDEAADQEEEAYLRED(-)	852.24	3	2553.70	70.8	2.88E-004
					218 - 237	(K)TQEASDEAADQEEEAYLRED(-)	1149.80	2	2297.58	112.0	1.62E-008
					216 - 237	(R)QKTQEASDEAADQEEEAYLRED(-)	1277.92	2	2553.83	95.9	5.74E-007

Spot 68 [P1014-11 (75955 Rattus)] Diablo								
LCMS/MS								
Calculated	Observed	+/-ppm	start	end	Sequence	Ion Score	C.I.%	Modification
1237.606	413.9484		1	10	-.MAALRSWMTR.S	8		Oxidation (M)
1460.6929	732.1409		61	73	K.SEPQSLSNEALMR.R	52		
1616.794	809.3199		61	74	K.SEPQSLSNEALMRR.A	12		
850.5025	426.1824		183	189	R.NHIQLVK.S	40		
844.4403	423.1024		190	196	K.SQVQEVR.Q	37		
1857.9908	620.0989		190	205	K.SQVQEVRQLSQKAETK.L	11		
1185.6353	593.6819		206	215	K.LAEVQTQELR.Q	87		
1185.6353	593.7024		206	215	K.LAEVQTQELR.Q	89		
1185.6353	593.7544		206	215	K.LAEVQTQELR.Q	12		
1185.6353	594.6804		206	215	K.LAEVQTQELR.Q	56		
2554.0943	852.1628		216	237	R.QKTQEASDEAADQEEEAYLRED.-	47		
2554.0943	852.2395		216	237	R.QKTQEASDEAADQEEEAYLRED.-	71		
2554.0943	1277.9209		216	237	R.QKTQEASDEAADQEEEAYLRED.-	96		
2297.9407	1149.7985		218	237	K.TQEASDEAADQEEEAYLRED.-	112		
2297.9407	767.0619		218	237	K.TQEASDEAADQEEEAYLRED.-	41		
2297.9407	1150.7819		218	237	K.TQEASDEAADQEEEAYLRED.-	63		

Spot 70 (P1014-14) – mixture of: Ras-related protein Rab-6A [Mus musculus] (Rab6a), Inosine triphosphatase, isoform CRA_a (Itpa) and Ropporin-like protein (AKAP-associated sperm protein) [Mus musculus] (Ropn1l)

**Spot 70 (P1014-14) Rab6a, Itpa & Ropn1l**											
**LCMS/MS (Scaffold)**											
Sample Name	Acc No.	Proteins(s) inferred	No. Peptides identified	Sequence Coverage	Position in sequence	Peptide Sequence	Precursor m/z	Charge observed	Calculated mass (neutral)	Mascot score	Mascot Expect value
P1014-14	P35279	sp|P35279|Ras-related protein Rab-6A Tax_Id=10090 [Mus musculus]	4	18.30%	2 - 12	(M)sAGGDFGNPLR(K)	566.58	2	1131.15	71.9	2.88E-004
					2 - 13	(M)sAGGDFGNPLRK(F)	630.70	2	1259.38	58.5	6.01E-003
					147 - 158	(K)ELNVMFIETSAK(A)	691.66	2	1381.31	80.7	3.30E-005
					170 - 183	(R)VAAALPGMESTQDR(S)	723.18	2	1444.35	73.8	1.58E-004
	ENSRNOP00000028838	ens|ENSRNOP00000028838|ENSRNOG00000021233 transcript:ENSRNOT00000028838 Tax_Id=10116 [Rattus norvegicus]	2	10.60%	2 - 8	(M)aASLVGK(K)	687.26	1	686.25	51.5	2.13E-002
					185 - 198	(K)LQEYFGVTDGAGDH(-)	754.72	2	1507.42	77.7	6.44E-005
	Q9EQ00	sp|Q9EQ00|Ropporin-1-like protein Tax_Id=10090 [Mus musculus]	1	4.78%	70 - 80	(K)TDTGLTQGLLK(V)	573.70	2	1145.38	44.9	1.48E-001

**Spot 70 [P1014-14 (75073 Rodent)] Rab6a**								
**LCMS/MS**								
Calculated	Observed	+/-ppm	start	end	Sequence	Ion Score	C.I.%	Modification
1236.5557	413.1712		1	12	-.MSAGGDFGNPLR.K	11		Oxidation (M)
1131.5309	566.5807		2	12	M.SAGGDFGNPLR.K	72		Acetyl (Protein N-term)
1259.6258	630.6987		2	13	M.SAGGDFGNPLRK.F	59		Acetyl (Protein N-term)
1671.8693	558.4215		61	74	R.TVRLQLWDTAGQER.F	11		
1315.6521	659.6417		64	74	R.LQLWDTAGQER.F	40		
1380.6959	691.6606		147	158	K.ELNVMFIETSAK.A	81		
1444.698	723.1622		170	183	R.VAAALPGMESTQDR.S	65		
1444.698	723.1802		170	183	R.VAAALPGMESTQDR.S	74		
1105.5437	553.6207		184	192	R.SREDMIDIK.L	18		

**Spot 70 [P1014-14 (75073 Rodent)] Itpa**								
**LCMS/MS**								
Calculated	Observed	+/-ppm	start	end	Sequence	Ion Score	C.I.%	Modification
1744.0247	582.5378		2	18	M.AASLVGKKIVFVTGNAK.K	16		Acetyl (Protein N-term)
1075.639	539.1472		9	18	K.KIVFVTGNAK.K	27		
1198.6492	400.5215		134	144	R.GKTPGQIVMPR.G	26		Oxidation (M)
997.5379	499.6796		136	144	K.TPGQIVMPR.G	39		
1507.658	754.7192		185	198	K.LQEYFGVTDGAGDH.-	78		

Spot 72 (D23 20070719) – L-lactate dehydrogenase B chain (Ldhb)

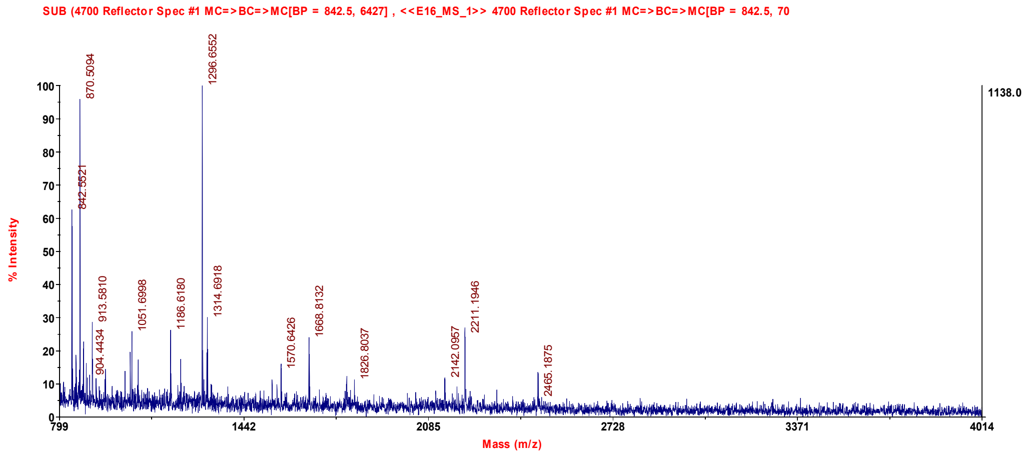

Spot 72 (20070719-D23) Ldhb								
PMF/MALDI								
Calculated	Observed	+/-ppm	start	end	Sequence	Ion Score	C.I.%	Modification
912.5757	913.581	-2	92	100	K.IVVVTAGVR.Q			
1555.8066	1556.8	-9	101	113	R.QQEGESRLNLVQR.N			
1072.5375	1073.5481	3	171	178	R.FRYLMAEK.L			Oxidation (M)
1026.5532	1027.5437	-16	271	279	R.IHPVSTMVK.G			Oxidation (M)
958.5447	959.5363	-16	300	308	R.GLTSVINQK.L			
1185.6353	1186.618	-21	309	318	K.LKDDEVAQLR.K			
1313.7303	1314.6918	-35	309	319	K.LKDDEVAQLRK.S			

Spot 74 (D14 20070719) – Alpha-enolase (Eno1)

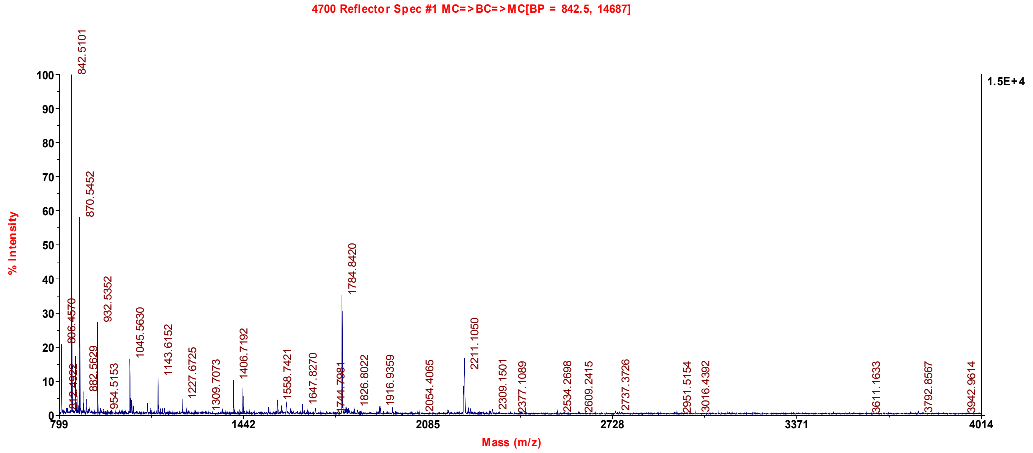

Spot 74 (20070719-D14) Eno1								
MS+MS/MS								
Calculated	Observed	+/-ppm	start	end	Sequence	Ion Score	C.I.%	Modification
806.4518	806.4557	5	406	411	YNQILR			
806.4518	806.4557	5	406	411	YNQILR			
904.4622	904.4578	-5	412	419	IEEELGSK			
932.5311	932.533	2	426	433	SFRNPLAK			
932.5311	932.533	2	426	433	SFRNPLAK			
1056.536	1056.5317	-4	253	261	AGKYDLDFK			
1118.6681	1118.6663	-2	403	411	LAKYNQILR			
1143.6156	1143.6166	1	183	192	IGAEVYHNLK	9	87.218	
1143.6156	1143.6166	1	183	192	IGAEVYHNLK			
1406.7162	1406.723	5	15	27	GNPTVEVDLYTAK	34	99.959	
1406.7162	1406.723	5	15	27	GNPTVEVDLYTAK			
1439.7417	1439.7417	0	269	280	YITPDQLADLYK			
1439.7417	1439.7417	0	269	280	YITPDQLADLYK	1	9.09	
1528.6914	1528.6963	3	256	268	YDLDFKSPDDASR			
1646.8319	1646.8208	-7	179	192	EAMRIGAEVYHNLK			Oxidation (M)
1652.7506	1652.7976	28	89	102	IDQLMIEMDGTENK			Oxidation (M) [5]
1691.8962	1691.9104	8	406	419	YNQILRIEEELGSK			
1784.845	1784.8458	0	253	268	AGKYDLDFKSPDDASR			
1784.845	1784.8458	0	253	268	AGKYDLDFKSPDDASR	20	98.802	
1804.9438	1804.986	23	32	49	AAVPSGASTGIYEALELR			
1960.9246	1960.9298	3	202	220	DATNVGDEGGFAPNILENK			
2154.0713	2154.0872	7	9	27	EIFDSRGNPTVEVDLYTAK			
2208.0278	2208.0452	8	233	252	AGYTDQVVIGMDVAASEFYR			Oxidation (M)
2208.0278	2208.0452	8	233	252	AGYTDQVVIGMDVAASEFYR			Oxidation (M)
2309.1042	2309.1619	25	199	220	YGKDATNVGDEGGFAPNILENK			
2736.343	2736.3987	20	80	102	KLNVVEQEKIDQLMIEMDGTENK			2 Oxidation (M)

Spot 74 (20070719-D14) Eno1								
PMF/MALDI								
Calculated	Observed	+/-ppm	start	end	Sequence	Ion Score	C.I.%	Modification
2153.0641	2154.0771	3	10	28	R.EIFDSRGNPTVEVDLYTAK.G			
1405.7089	1406.7192	2	16	28	R.GNPTVEVDLYTAK.G			
1803.9366	1804.9744	17	33	50	R.AAVPSGASTGIYEALELR.D			
1180.6564	1181.6691	5	61	71	K.GVSKAVEHINK.T			
809.4395	810.4442	-3	65	71	K.AVEHINK.T			
898.5488	899.5757	22	72	80	K.TIAPALVSK.K			
1026.6437	1027.6345	-16	72	81	K.TIAPALVSKK.L			
1651.7433	1652.7947	27	90	103	K.IDQLMIEMDGTENK.S			Oxidation (M)
1645.8246	1646.816	-10	180	193	R.EAMRIGAEVYHNLK.N			Oxidation (M)
1142.6084	1143.6152	0	184	193	R.IGAEVYHNLK.N			
2308.0971	2309.1501	20	200	221	K.YGKDATNVGDEGGFAPNILENK.E			
1959.9174	1960.9214	-2	203	221	K.DATNVGDEGGFAPNILENK.E			
2191.0256	2192.0403	3	234	253	K.AGYTDQVVIGMDVAASEFYR.A			
2207.0205	2208.0347	3	234	253	K.AGYTDQVVIGMDVAASEFYR.A			Oxidation (M)
1055.5287	1056.53	-6	254	262	R.AGKYDLDFK.S			
1527.6841	1528.6925	1	257	269	K.YDLDFKSPDDASR.Y			
1438.7344	1439.7386	-2	270	281	R.YITPDQLADLYK.S			
2624.2495	2625.2419	-6	336	358	K.SCNCLLLKVNQIGSVTESLQACK.L			
1117.6607	1118.6647	-3	404	412	R.LAKYNQILR.I			
805.4446	806.4569	6	407	412	K.YNQILR.I			
1690.8889	1691.9058	6	407	420	K.YNQILRIEEELGSK.A			
903.4549	904.4594	-3	413	420	R.IEEELGSK.A			
931.5239	932.5352	4	427	434	R.SFRNPLAK.-			

Spot 83 (B8 20080606) - Nuclear autoantigenic sperm protein (Nasp)

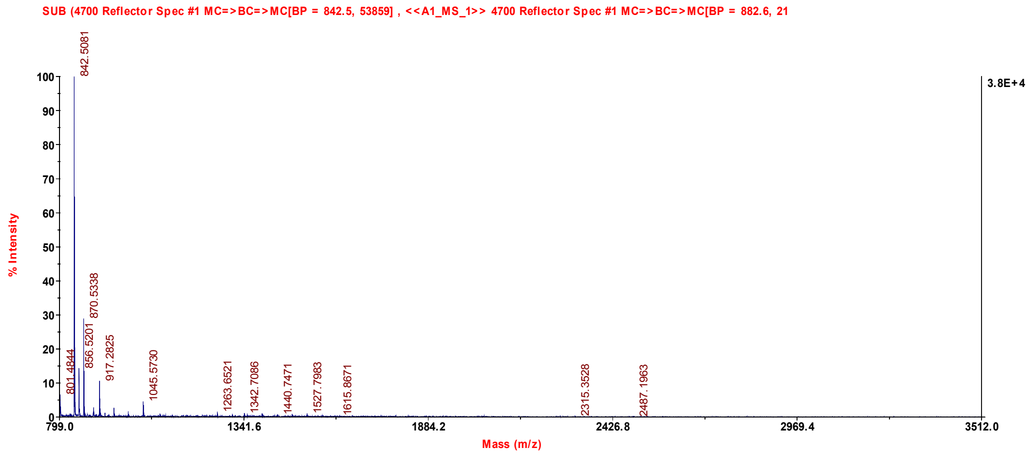

Spot 83 (20080606-B8) Nasp								
MS+MS/MS								
Calculated	Observed	+/-ppm	start	end	Sequence	Ion Score	C.I.%	Modification
801.4828	801.4844	2	94	100	SLLELAR	20	98.009	
801.4828	801.4844	2	94	100	SLLELAR			
856.5614	856.5255	-42	45	52	KLLGLGQK			
856.5614	856.5255	-42	45	52	KLLGLGQK			
959.5519	959.5547	3	598	605	SIDVIEKR	17	95.926	
959.5519	959.5547	3	598	605	SIDVIEKR			
1001.5625	1001.5657	3	650	659	SGNVAELALK			
1001.5625	1001.5657	3	650	659	SGNVAELALK			
1094.584	1094.5774	-6	242	251	SISGVYVQNK			
1094.584	1094.5774	-6	242	251	SISGVYVQNK			
1342.7114	1342.7064	-4	524	535	EAQLYAAQAHLK			
1342.7114	1342.7064	-4	524	535	EAQLYAAQAHLK			
1351.641	1351.688	35	34	45	MESLDVDSEAKK			
1351.641	1351.688	35	34	45	MESLDVDSEAKK			
2487.2031	2487.2202	7	660	685	ATLVESSTSGFTPSGAGASVSMIASR			Oxidation (M)

Spot 83 (20080606-B8) Nasp								
PMF/MALDI								
Calculated	Observed	+/-ppm	start	end	Sequence	Ion Score	C.I.%	Modification
1350.6337	1351.6888	35	34	45	K.MESLDVDSEAKK.L			
855.5542	856.5201	-48	45	52	K.KLLGLGQK.H			
800.4756	801.4844	2	94	100	K.SLLELAR.M			
1262.6506	1263.6521	-5	435	445	R.ATEKAPEDKFK.I			
1341.7041	1342.7086	-2	524	535	K.EAQLYAAQAHLK.L			
958.5447	959.5576	6	598	605	K.SIDVIEKR.M			
1093.5511	1094.5697	10	606	614	R.MAVLLEQMK.E			2 Oxidation (M)
831.4338	832.4793	46	639	645	R.EKIEDAK.E			
1000.5553	1001.5581	-4	650	659	R.SGNVAELALK.A			
2486.1959	2487.1963	-3	660	685	K.ATLVESSTSGFTPSGAGASVSMIASR.K			Oxidation (M)

Spot 86 (P1014-01) – Actin, cytoplasmic 2 (Actg1)

**Spot 86 [P1014-01 (75058 Rodent)] Actgl**								
**LCMS/MS**								
Calculated	Observed	+/-ppm	start	end	Sequence	Ion Score	C.I.%	Modification
2851.264	951.1815		2	28	M.EEEIAALVIDNGSGMCKAGFAGDDAPR.A	60		Acetyl (Protein N-term); Oxidation (M)
975.441	488.1391		19	28	K.AGFAGDDAPR.A	48		
1197.6982	599.6592		29	39	R.AVFPSIVGRPR.H	25		
1789.8846	895.7382		239	254	K.SYELPDGQVITIGNER.F	91		
1789.8846	896.2407		239	254	K.SYELPDGQVITIGNER.F	44		
1547.8051	517.3168		313	326	R.MQKEITALAPSTMK.I	26		

Spot 87 (E10 20070719) – Ferritin heavy chain (Fth1)

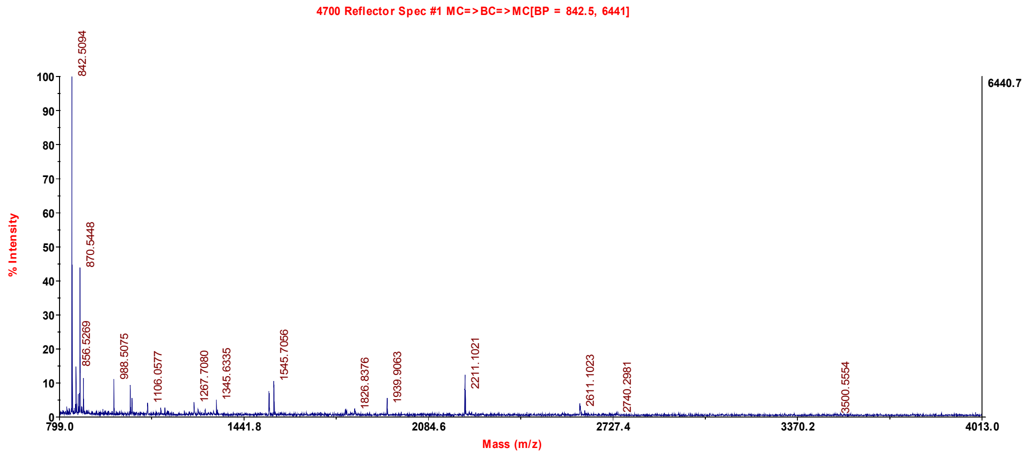

Spot 87 (20070719-E10) Fth1								
PMF/MALDI								
Calculated	Observed	+/-ppm	start	end	Sequence	Ion Score	C.I.%	Modification
1544.6967	1545.7056	1	11	23	R.QNYHQDSEAAINR.Q			
1344.6211	1345.6335	4	55	64	K.YFLHQSHEER.E			
1938.8972	1939.9062	1	55	69	K.YFLHQSHEEREHAEK.L			
875.5116	876.5132	-6	81	87	R.IFLQDIK.K			
1266.6932	1267.708	6	110	120	K.SVNQSLLELHK.L			
1280.6837	1281.6801	-9	148	158	K.ELGDHVTNLRK.M			
2610.1003	2611.1023	-2	159	182	K.MGAPESGMAEYLFDKHTLGHGDES.-			2 Oxidation (M)

Spot 87 (D20 20080815) - Ferritin heavy chain (Fth1)

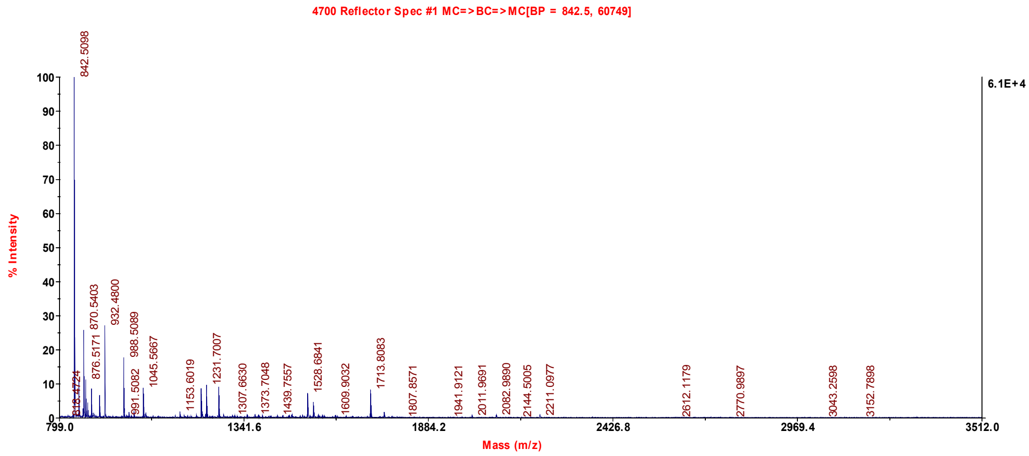

Spot 87 (20080815-D20) Fth1								
PMF/MALDI								
Calculated	Observed	+/-ppm	start	end	Sequence	Ion Score	C.I.%	Modification
1544.6967	1545.7065	2	11	23	R.QNYHQDSEAAINR.Q			
1639.8464	1640.809	-27	65	77	R.EHAEKLMKLQNQR.G			Oxidation (M)
927.4998	928.5146	8	73	80	K.LQNQRGGR.I			
1641.9315	1642.8972	-25	78	91	R.GGRIFLQDIKKPDR.D			
875.5116	876.5171	-2	81	87	R.IFLQDIK.K			
1266.6932	1267.7045	3	110	120	K.SVNQSLLELHK.L			
1480.7998	1481.8113	3	145	157	K.SIKELGDHVTNLR.K			
1608.8947	1609.9032	1	145	158	K.SIKELGDHVTNLRK.M			
1152.5887	1153.6019	5	148	157	K.ELGDHVTNLR.K			
1280.6837	1281.6912	0	148	158	K.ELGDHVTNLRK.M			
1676.7062	1677.7246	7	159	173	K.MGAPESGMAEYLFDK.H			2 Oxidation (M)
2610.1003	2611.0979	-4	159	182	K.MGAPESGMAEYLFDKHTLGHGDES.-			2 Oxidation (M)

Spot 91 (E8 20070719) – Proteasome subunit beta type 4 precursor (Psmb4)

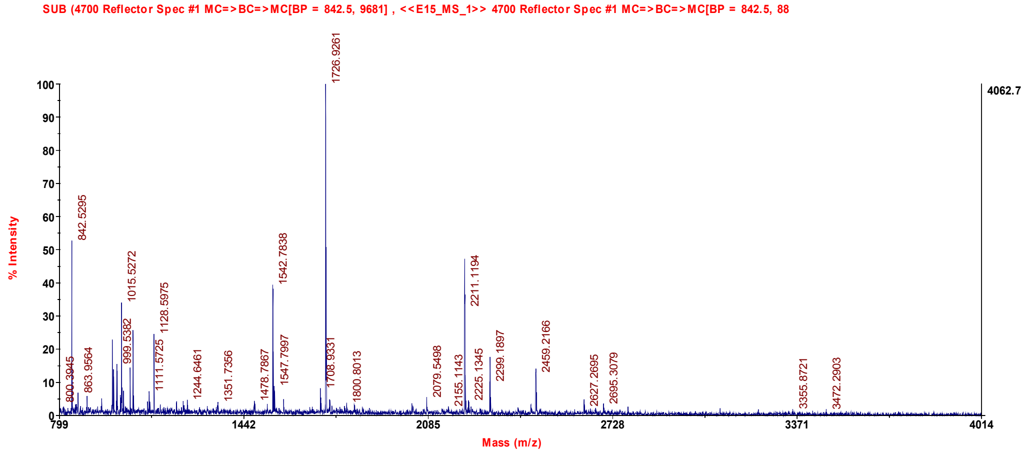

Spot 91 (20070719-E8) Psmb4								
MS+MS/MS								
Calculated	Observed	+/-ppm	start	end	Sequence	Ion Score	C.I.%	Modification
983.5421	983.5433	1	131	138	AIHSWLTR			
983.5421	983.5433	1	131	138	AIHSWLTR			
1022.5516	1022.5544	3	231	239	FQVATVTEK			
1055.5632	1055.5597	-3	219	226	VLYYRDAR			
1055.5632	1055.5597	-3	219	226	VLYYRDAR			
1128.6008	1128.594	-6	201	210	QPVLSQTEAR			
1128.6008	1128.594	-6	201	210	QPVLSQTEAR			
1542.791	1542.7778	-9	227	239	SYNRFQVATVTEK			
1542.791	1542.7778	-9	227	239	SYNRFQVATVTEK			
1547.8097	1547.797	-8	45	59	TQNPMVTGTSVLGVK			Oxidation (M)
1726.9333	1726.9211	-7	196	210	EVLEKQPVLSQTEAR			
1726.9333	1726.9211	-7	196	210	EVLEKQPVLSQTEAR	23	99.414	
2627.2117	2627.2341	9	86	108	IMRVNDSTMLGASGDYADFQYLK			2 Oxidation (M)

Spot 91 (20070719-E8) Psmb4								
PMF/MALDI								
Calculated	Observed	+/-ppm	start	end	Sequence	Ion Score	C.I.%	Modification
1546.8025	1547.7997	-7	46	60	R.TQNPMVTGTSVLGVK.F			Oxidation (M)
2626.2043	2627.2695	22	87	109	R.IMRVNDSTMLGASGDYADFQYLK.Q			2 Oxidation (M)
2458.1798	2459.2166	12	110	131	K.QVLGQMVIDEELLGDGHSYSPR.A			Oxidation (M)
982.5348	983.5345	-8	132	139	R.AIHSWLTR.A			
1725.9261	1726.9261	-4	197	211	R.EVLEKQPVLSQTEAR.E			
1127.5935	1128.5975	-3	202	211	K.QPVLSQTEAR.E			
1054.556	1055.5599	-3	220	227	R.VLYYRDAR.S			
1541.7838	1542.7838	-5	228	240	R.SYNRFQVATVTEK.G			
1021.5444	1022.554	2	232	240	R.FQVATVTEK.G			

Spot 93 (D9 20080606) - mixture of: Tubulin, alpha 1C (Tuba1c), Tubulin alpha-1A chain (Tuba1a), Tubulin alpha-3 chain (Tuba3), Tubulin beta-2C chain (Tubb2c), Tubulin beta-3 chain (Tubb3) and Tubulin beta-2A chain (Tubb2a)

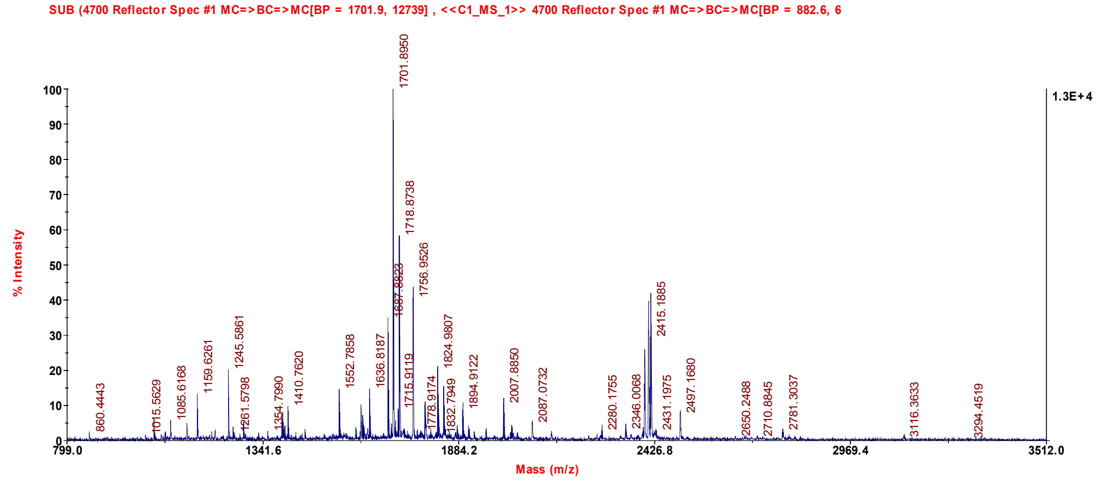

Spot 93 (20080606-D9) Tuba1c								
PMF/MALDI								
Calculated	Observed	+/-ppm	start	end	Sequence	Ion Score	C.I.%	Modification
2006.8858	2007.885	-4	41	60	K.TIGGGDDSFNTFFSETGAGK.H			
2496.167	2497.168	-3	41	64	K.TIGGGDDSFNTFFSETGAGKHVPR.A			
1700.8985	1701.895	-6	65	79	R.AVFVDLEPTVIDEVR.T			
2279.1798	2280.1755	-5	65	84	R.AVFVDLEPTVIDEVRTGTYR.Q			
1409.7667	1410.762	-9	85	96	R.QLFHPEQLITGK.E			
2414.1978	2415.1885	-7	85	105	R.QLFHPEQLITGKEDAANNYAR.G			
1841.0047	1841.9949	-9	106	121	R.GHYTIGKEIIDLVLDR.I			
1084.6128	1085.6168	-3	113	121	K.EIIDLVLDR.I			
1353.798	1354.799	-5	113	123	K.EIIDLVLDRIR.K			
1873.9758	1874.9489	-18	215	229	R.RNLDIERPTYTNLNR.L			
1717.8747	1718.8738	-5	216	229	R.NLDIERPTYTNLNR.L			
2408.2012	2409.199	-4	244	264	R.FDGALNVDLTEFQTNLVPYPR.I			
1755.9559	1756.9526	-6	265	280	R.IHFPLATYAPVISAEK.A			
2780.2785	2781.3037	6	281	304	K.AYHEQLTVAEITNACFEPANQMVK.C			Oxidation (M)
3293.4791	3294.4519	-10	281	308	K.AYHEQLTVAEITNACFEPANQMVKCDPR.H			
1609.9039	1610.8794	-20	321	336	R.GDVVPKDVNAAIATIK.T			
1014.5709	1015.5629	-15	327	336	K.DVNAAIATIK.T			
1754.845	1755.8573	3	339	352	K.RTIQFVDWCPTGFK.V			
1851.9843	1852.9762	-8	353	370	K.VGINYQPPTVVPGGDLAR.V			
1395.6857	1396.6849	-6	391	401	R.LDHKFDLMYAK.R			Oxidation (M)
1058.5219	1059.5314	2	395	402	K.FDLMYAKR.A			Oxidation (M)
2501.107	2502.1455	12	402	422	K.RAFVHWYVGEGMEEGEFSEAR.E			Oxidation (M)
2329.011	2330.0056	-5	403	422	R.AFVHWYVGEGMEEGEFSEAR.E			
2345.0059	2346.0068	-3	403	422	R.AFVHWYVGEGMEEGEFSEAR.E			Oxidation (M)

Spot 93 (20080606-D9) Tuba1a								
PMF/MALDI								
Calculated	Observed	+/-ppm	start	end	Sequence	Ion Score	C.I.%	Modification
2006.8858	2007.885	-4	41	60	K.TIGGGDDSFNTFFSETGAGK.H			
2496.167	2497.168	-3	41	64	K.TIGGGDDSFNTFFSETGAGKHVPR.A			
1700.8985	1701.895	-6	65	79	R.AVFVDLEPTVIDEVR.T			
2279.1798	2280.1755	-5	65	84	R.AVFVDLEPTVIDEVRTGTYR.Q			
1409.7667	1410.762	-9	85	96	R.QLFHPEQLITGK.E			
2414.1978	2415.1885	-7	85	105	R.QLFHPEQLITGKEDAANNYAR.G			
1841.0047	1841.9949	-9	106	121	R.GHYTIGKEIIDLVLDR.I			
1084.6128	1085.6168	-3	113	121	K.EIIDLVLDR.I			
1353.798	1354.799	-5	113	123	K.EIIDLVLDRIR.K			
1873.9758	1874.9489	-18	215	229	R.RNLDIERPTYTNLNR.L			
1717.8747	1718.8738	-5	216	229	R.NLDIERPTYTNLNR.L			
1456.8613	1457.8611	-5	230	243	R.LIGQIVSSITASLR.F			
2408.2012	2409.199	-4	244	264	R.FDGALNVDLTEFQTNLVPYPR.I			
1755.9559	1756.9526	-6	265	280	R.IHFPLATYAPVISAEK.A			
1609.9039	1610.8794	-20	321	336	R.GDVVPKDVNAAIATIK.T			
1014.5709	1015.5629	-15	327	336	K.DVNAAIATIK.T			
1754.845	1755.8573	3	339	352	K.RTIQFVDWCPTGFK.V			
1823.9782	1824.9807	-3	353	370	K.VGINYQPPTVVPGGDLAK.V			
1395.6857	1396.6849	-6	391	401	R.LDHKFDLMYAK.R			Oxidation (M)
1058.5219	1059.5314	2	395	402	K.FDLMYAKR.A			Oxidation (M)
2501.107	2502.1455	12	402	422	K.RAFVHWYVGEGMEEGEFSEAR.E			Oxidation (M)
2329.011	2330.0056	-5	403	422	R.AFVHWYVGEGMEEGEFSEAR.E			
2345.0059	2346.0068	-3	403	422	R.AFVHWYVGEGMEEGEFSEAR.E			Oxidation (M)

Spot 93 (20080606-D9) Tuba3								
PMF/MALDI								
Calculated	Observed	+/-ppm	start	end	Sequence	Ion Score	C.I.%	Modification
2006.8858	2007.885	-4	41	60	K.TIGGGDDSFNTFFSETGAGK.H			
2496.167	2497.168	-3	41	64	K.TIGGGDDSFNTFFSETGAGKHVPR.A			
1686.8829	1687.8823	-5	65	79	R.AVFVDLEPTVVDEVR.T			
1409.7667	1410.762	-9	85	96	R.QLFHPEQLITGK.E			
2414.1978	2415.1885	-7	85	105	R.QLFHPEQLITGKEDAANNYAR.G			
1826.989	1827.9832	-7	106	121	R.GHYTIGKEIVDLVLDR.I			
1070.5972	1071.5952	-9	113	121	K.EIVDLVLDR.I			
1873.9758	1874.9489	-18	215	229	R.RNLDIERPTYTNLNR.L			
1717.8747	1718.8738	-5	216	229	R.NLDIERPTYTNLNR.L			
1456.8613	1457.8611	-5	230	243	R.LIGQIVSSITASLR.F			
2408.2012	2409.199	-4	244	264	R.FDGALNVDLTEFQTNLVPYPR.I			
1755.9559	1756.9526	-6	265	280	R.IHFPLATYAPVISAEK.A			
1609.9039	1610.8794	-20	321	336	R.GDVVPKDVNAAIATIK.T			
1014.5709	1015.5629	-15	327	336	K.DVNAAIATIK.T			
1754.845	1755.8573	3	339	352	K.RTIQFVDWCPTGFK.V			
1823.9782	1824.9807	-3	353	370	K.VGINYQPPTVVPGGDLAK.V			
1395.6857	1396.6849	-6	391	401	R.LDHKFDLMYAK.R			Oxidation (M)
1058.5219	1059.5314	2	395	402	K.FDLMYAKR.A			Oxidation (M)
2501.107	2502.1455	12	402	422	K.RAFVHWYVGEGMEEGEFSEAR.E			Oxidation (M)
2329.011	2330.0056	-5	403	422	R.AFVHWYVGEGMEEGEFSEAR.E			
2345.0059	2346.0068	-3	403	422	R.AFVHWYVGEGMEEGEFSEAR.E			Oxidation (M)

Spot 93 (20080606-D9) Tubb2c								
PMF/MALDI								
Calculated	Observed	+/-ppm	start	end	Sequence	Ion Score	C.I.%	Modification
3115.4159	3116.3633	-19	20	46	K.FWEVISDEHGIDPTGTYHGDSDLQLER.I			
1327.6408	1328.6257	-17	47	58	R.INVYYNEATGGK.Y			
1616.808	1617.7889	-16	63	77	R.AVLVDLEPGTMDSVR.S			Oxidation (M)
2797.3361	2798.3181	-9	78	103	R.SGPFGQIFRPDNFVFGQSGAGNNWAK.G			
1957.9745	1958.9585	-12	104	121	K.GHYTEGAELVDSVLDVVR.K			
2086.0695	2087.0732	-2	104	122	K.GHYTEGAELVDSVLDVVRK.E			
1076.525	1077.5459	13	155	162	K.IREEYPDR.I			
1129.588	1130.5935	-2	242	251	R.FPGQLNADLR.K			
1257.683	1258.6805	-8	242	252	R.FPGQLNADLRK.L			
1286.7169	1287.7161	-6	252	262	R.KLAVNMVPFPR.L			Oxidation (M)
1158.6219	1159.6261	-3	253	262	K.LAVNMVPFPR.L			Oxidation (M)
1619.8283	1620.8158	-12	263	276	R.LHFFMPGFAPLTSR.G			
1635.8232	1636.8187	-7	263	276	R.LHFFMPGFAPLTSR.G			Oxidation (M)
1706.8549	1707.8583	-2	283	297	R.ALTVPELTQQMFDAK.N			Oxidation (M)
1038.5862	1039.5879	-5	310	318	R.YLTVAAVFR.G			
1695.8257	1696.8379	3	337	350	K.NSSYFVEWIPNNVK.T			
1856.9342	1857.9418	0	363	379	K.MSATFIGNSTAIQELFK.R			
1872.9291	1873.9199	-9	363	379	K.MSATFIGNSTAIQELFK.R			Oxidation (M)
2029.0302	2030.0256	-6	363	380	K.MSATFIGNSTAIQELFKR.I			Oxidation (M)
1244.586	1245.5861	-6	381	390	R.ISEQFTAMFR.R			Oxidation (M)
1400.6871	1401.6891	-4	381	391	R.ISEQFTAMFRR.K			Oxidation (M)

Spot 93 (20080606-D9) Tubb3								
PMF/MALDI								
Calculated	Observed	+/-ppm	start	end	Sequence	Ion Score	C.I.%	Modification
1911.9479	1912.9189	-19	47	62	R.ISVYYNEASSHKYVPR.A			
1630.8236	1631.8127	-11	63	77	R.AILVDLEPGTMDSVR.S			Oxidation (M)
2794.3364	2795.3408	-1	78	103	R.SGAFGHLFRPDNFIFGQSGAGNNWAK.G			
1957.9745	1958.9585	-12	104	121	K.GHYTEGAELVDSVLDVVR.K			
2086.0695	2087.0732	-2	104	122	K.GHYTEGAELVDSVLDVVRK.E			
1129.588	1130.5935	-2	242	251	R.FPGQLNADLR.K			
1257.683	1258.6805	-8	242	252	R.FPGQLNADLRK.L			
1286.7169	1287.7161	-6	252	262	R.KLAVNMVPFPR.L			Oxidation (M)
1158.6219	1159.6261	-3	253	262	K.LAVNMVPFPR.L			Oxidation (M)
1619.8283	1620.8158	-12	263	276	R.LHFFMPGFAPLTAR.G			Oxidation (M)
1706.8549	1707.8583	-2	283	297	R.ALTVPELTQQMFDAK.N			Oxidation (M)
1068.5968	1069.5999	-4	310	318	R.YLTVATVFR.G			
1882.8838	1883.9065	8	321	336	R.MSMKEVDEQMLAIQSK.N			Oxidation (M)
1695.8257	1696.8379	3	337	350	K.NSSYFVEWIPNNVK.V			
1872.9291	1873.9199	-9	363	379	K.MSSTFIGNSTAIQELFK.R			
2029.0302	2030.0256	-6	363	380	K.MSSTFIGNSTAIQELFKR.I			
2045.0252	2046.0283	-2	363	380	K.MSSTFIGNSTAIQELFKR.I			Oxidation (M)
1244.586	1245.5861	-6	381	390	R.ISEQFTAMFR.R			Oxidation (M)
1400.6871	1401.6891	-4	381	391	R.ISEQFTAMFRR.K			Oxidation (M)

Spot 93 (20080606-D9) Tubb2a								
PMF/MALDI								
Calculated	Observed	+/-ppm	start	end	Sequence	Ion Score	C.I.%	Modification
1630.8236	1631.8127	-11	63	77	R.AILVDLEPGTMDSVR.S			Oxidation (M)
2797.3361	2798.3181	-9	78	103	R.SGPFGQIFRPDNFVFGQSGAGNNWAK.G			
1957.9745	1958.9585	-12	104	121	K.GHYTEGAELVDSVLDVVR.K			
2086.0695	2087.0732	-2	104	122	K.GHYTEGAELVDSVLDVVRK.E			
1076.525	1077.5459	13	155	162	K.IREEYPDR.I			
1129.588	1130.5935	-2	242	251	R.FPGQLNADLR.K			
1257.683	1258.6805	-8	242	252	R.FPGQLNADLRK.L			
1286.7169	1287.7161	-6	252	262	R.KLAVNMVPFPR.L			Oxidation (M)
1158.6219	1159.6261	-3	253	262	K.LAVNMVPFPR.L			Oxidation (M)
1619.8283	1620.8158	-12	263	276	R.LHFFMPGFAPLTSR.G			
1635.8232	1636.8187	-7	263	276	R.LHFFMPGFAPLTSR.G			Oxidation (M)
1706.8549	1707.8583	-2	283	297	R.ALTVPELTQQMFDSK.N			
1722.8498	1723.875	10	283	297	R.ALTVPELTQQMFDSK.N			Oxidation (M)
1695.8257	1696.8379	3	337	350	K.NSSYFVEWIPNNVK.T			
1856.9342	1857.9418	0	363	379	K.MSATFIGNSTAIQELFK.R			
1872.9291	1873.9199	-9	363	379	K.MSATFIGNSTAIQELFK.R			Oxidation (M)
2029.0302	2030.0256	-6	363	380	K.MSATFIGNSTAIQELFKR.I			Oxidation (M)
1244.586	1245.5861	-6	381	390	R.ISEQFTAMFR.R			Oxidation (M)
1400.6871	1401.6891	-4	381	391	R.ISEQFTAMFRR.K			Oxidation (M)

Spot 95 (P1014-12) – Acrosin-binding protein (Acrbp)

Spot 95 (P1014-12) Acrbp												
LCMS/MS (Scaffold)												
Sample Name	Acc No.	Proteins(s) inferred	No. Peptides identified	Sequence Coverage	Position in sequence	Peptide Sequence	Precursor m/z	Charge observed	Calculated mass (neutral)	Mascot score	Mascot Expect value
P1014-12	Q6AY33	sp|Q6AY33|Acrosin-binding protein Tax_Id=10116 [Rattus norvegicus]	1	3.80%	259 - 270	(K)SLSSNPSFFTPR(V)	670.24	2	1338.47	43.1	1.90E-001

Spot 96 (D4 20070719) – Stress-70 protein, mitochondrial precursor (Hspa9)

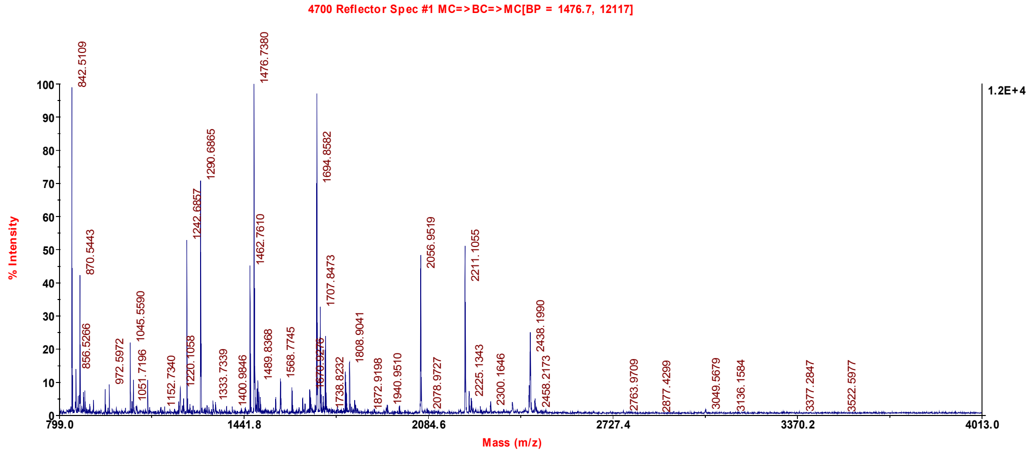

Spot 96 (20070719-D4) Hspa9								
MS+MS/MS								
Calculated	Observed	+/-ppm	start	end	Sequence	Ion Score	C.I.%	Modification
887.5131	887.5158	3	100	107	LVGMPAKR			Oxidation (M)
917.4437	917.4446	1	647	653	LFEMAYK			Oxidation (M)
958.4952	958.5029	8	77	85	VLENAEGAR			
972.5988	972.5967	-2	139	146	NVPFKIVR			
1045.5387	1045.5582	19	647	654	LFEMAYKK			Oxidation (M)
1056.6312	1056.6384	7	285	292	HIVKEFKR			
1231.6641	1231.671	6	635	646	QAASSLQQASLK			
1242.6801	1242.6831	2	207	218	DAGQISGLNVLR			
1242.6801	1242.6831	2	207	218	DAGQISGLNVLR	17	97.309	
1290.6801	1290.6836	3	395	405	VQQTVQDLFGR	28	99.785	
1290.6801	1290.6836	3	395	405	VQQTVQDLFGR			
1380.6501	1380.6456	-3	564	574	NAEKYAEEDRR			
1462.757	1462.7568	0	378	391	SDIGEVILVGGMTR			Oxidation (M)
1462.757	1462.7568	0	378	391	SDIGEVILVGGMTR	19	98.462	Oxidation (M)
1476.7329	1476.7334	0	86	99	TTPSVVAFTPDGER	35	99.957	
1476.7329	1476.7334	0	86	99	TTPSVVAFTPDGER			
1489.8373	1489.8339	-2	349	361	AQFEGIVTDLIKR			
1493.723	1493.7272	3	127	138	RYDDPEVQKDTK			
1568.7704	1568.7692	-1	108	121	QAVTNPNNTFYATK			
1569.8345	1569.7725	-39	160	173	LYSPSQIGAFVLMK			Oxidation (M)
1608.7687	1608.769	0	174	187	MKETAENYLGHTAK			Oxidation (M)
1645.8795	1645.8723	-4	219	234	VINEPTAAALAYGLDK			
1670.9183	1670.9193	1	203	218	QATKDAGQISGLNVLR			
1694.8496	1694.8511	1	188	202	NAVITVPAYFNDSQR			
1694.8496	1694.8511	1	188	202	NAVITVPAYFNDSQR	50	99.999	
1706.8378	1706.8391	1	293	307	ETGVDLTKDNMALQR	12	91.491	Oxidation (M)
1706.8378	1706.8391	1	293	307	ETGVDLTKDNMALQR			
1709.8717	1709.8374	-20	144	159	IVRASNGDAWVEAHGK			
1724.8715	1724.871	0	108	122	QAVTNPNNTFYATKR	18	98.147	
1724.8715	1724.871	0	108	122	QAVTNPNNTFYATKR			
1808.9025	1808.8973	-3	469	485	SQVFSTAADGQTQVEIK	37	99.973	
1808.9025	1808.8973	-3	469	485	SQVFSTAADGQTQVEIK			
2055.9617	2055.9548	-3	266	284	STNGDTFLGGEDFDQALLR			
2233.1055	2233.0835	-10	626	646	DSETGENIRQAASSLQQASLK			
2434.2129	2434.2131	0	542	563	EQQIVIQSSGGLSKDDIENMVK			Oxidation (M)

Spot 96 (20070719-D4) Hspa9								
PMF/MALDI								
Calculated	Observed	+/-ppm	start	end	Sequence	Ion Score	C.I.%	Modification
2078.0024	2078.9724	-18	53	73	K.GAVVGIDLGTTNSCVAVMEGK.Q			
2421.188	2422.1863	-4	53	76	K.GAVVGIDLGTTNSCVAVMEGKQAK.V			Oxidation (M)
957.4879	958.5032	8	77	85	K.VLENAEGAR.T			
1475.7256	1476.7379	3	86	99	R.TTPSVVAFTPDGER.L			
2188.1198	2189.1072	-9	86	106	R.TTPSVVAFTPDGERLVGMPAK.R			Oxidation (M)
886.5058	887.5168	4	100	107	R.LVGMPAKR.Q			Oxidation (M)
1567.7631	1568.7745	3	108	121	R.QAVTNPNNTFYATK.R			
1723.8642	1724.879	4	108	122	R.QAVTNPNNTFYATKR.L			
971.5916	972.5971	-2	139	146	K.NVPFKIVR.A			
1708.8645	1709.8452	-16	144	159	K.IVRASNGDAWVEAHGK.L			
1340.6109	1341.6771	44	147	159	R.ASNGDAWVEAHGK.L			
1568.8272	1569.7787	-36	160	173	K.LYSPSQIGAFVLMK.M			Oxidation (M)
1811.9678	1812.8894	-47	160	175	K.LYSPSQIGAFVLMKMK.E			
1607.7613	1608.7739	3	174	187	K.MKETAENYLGHTAK.N			Oxidation (M)
1693.8424	1694.858	5	188	202	K.NAVITVPAYFNDSQR.Q			
1669.9111	1670.9275	5	203	218	R.QATKDAGQISGLNVLR.V			
1241.6728	1242.6857	5	207	218	K.DAGQISGLNVLR.V			
1644.8723	1645.8791	0	219	234	R.VINEPTAAALAYGLDK.S			
2104.0688	2105.0999	11	219	238	R.VINEPTAAALAYGLDKSEDK.V			
2054.9545	2055.9653	2	266	284	K.STNGDTFLGGEDFDQALLR.H			
899.5229	900.5278	-3	285	291	R.HIVKEFK.R			
1689.8356	1690.8364	-4	293	307	R.ETGVDLTKDNMALQR.V			
1705.8305	1706.8453	4	293	307	R.ETGVDLTKDNMALQR.V			Oxidation (M)
862.3967	863.4466	49	301	307	K.DNMALQR.V			Oxidation (M)
1027.5597	1028.569	2	341	348	K.HLNMKLTR.A			Oxidation (M)
1332.7289	1333.7339	-2	349	360	R.AQFEGIVTDLIK.R			
1488.83	1489.8367	0	349	361	R.AQFEGIVTDLIKR.T			
1445.7548	1446.7496	-9	378	391	K.SDIGEVILVGGMTR.M			
1461.7497	1462.7609	3	378	391	K.SDIGEVILVGGMTR.M			Oxidation (M)
1289.6728	1290.6865	5	395	405	K.VQQTVQDLFGR.A			
1672.8897	1673.9004	2	395	409	K.VQQTVQDLFGRAPSK.A			
1807.8952	1808.9041	1	469	485	K.SQVFSTAADGQTQVEIK.V			
2433.2057	2434.2251	5	542	563	R.EQQIVIQSSGGLSKDDIENMVK.N			Oxidation (M)
1871.8935	1872.9198	10	579	595	R.VEAVNMAEGIIHDTETK.M			Oxidation (M)
903.496	904.5001	-3	618	624	K.MRELLAR.K			Oxidation (M)
2232.0982	2233.0935	-5	626	646	K.DSETGENIRQAASSLQQASLK.L			
1230.6568	1231.6729	7	635	646	R.QAASSLQQASLK.L			
916.4364	917.4445	1	647	653	K.LFEMAYK.K			Oxidation (M)
1044.5314	1045.559	19	647	654	K.LFEMAYKK.M			Oxidation (M)

Spot 103 (A23 20080606) - Inner membrane protein, mitochondrial, isoform CRA_a (Immt)

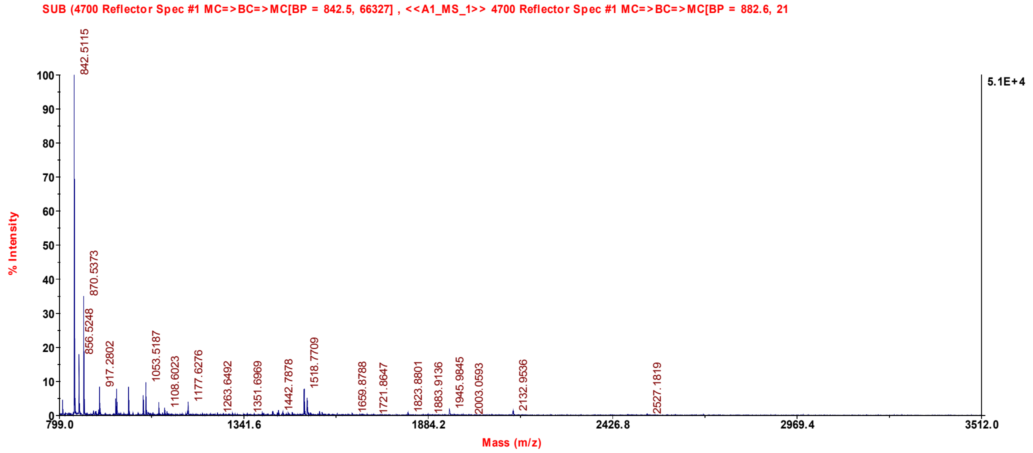

Spot 103 (20080606-A23) Immt (isoform CRA_a)								
PMF/MALDI								
Calculated	Observed	+/-ppm	start	end	Sequence	Ion Score	C.I.%	Modification
1394.6929	1395.7115	8	73	84	R.ESVEKTIPYSDK.L			
966.5862	967.5923	-1	102	110	K.KPIQSGPLK.I			
1306.6075	1307.6732	45	196	206	K.QEQVEMESLAK.S			Oxidation (M)
1517.7685	1518.7709	-3	207	220	K.SLEDALNQTATVTR.Q			
1441.7889	1442.7878	-6	221	234	R.QTITAQNAAVQAVK.A			
1001.5505	1002.5576	0	260	268	R.TVEGALKER.R			
1114.587	1115.587	-6	271	281	K.AVDEAADALLK.A			
905.4528	906.4358	-27	284	290	K.EELEKMK.T			
1562.7828	1563.7844	-4	300	314	R.EIAGATPYITAAEEK.L			
1454.7803	1455.7823	-4	315	327	K.LHSMIVDLDSVVK.K			
1470.7752	1471.776	-4	315	327	K.LHSMIVDLDSVVK.K			Oxidation (M)
1058.572	1059.5907	11	328	337	K.KVQAAQSEAK.V			
1526.8205	1527.8156	-8	338	350	K.VVSQYHELVVQAR.D			
1570.7879	1571.8253	19	356	369	K.ELDSITPDITPGWK.G			
913.5093	914.5098	-7	390	396	R.RIDQLNR.E			
1536.826	1537.8278	-4	403	415	K.ATEKQHIELALER.Q			
1107.6036	1108.6023	-8	407	415	K.QHIELALER.Q			
963.5138	964.5188	-2	422	430	K.RAFDSAVAK.A			
807.4127	808.4213	2	423	430	R.AFDSAVAK.A			
2131.948	2132.9536	-1	507	524	R.SQEQMDNFTLDINTAYAR.L			Oxidation (M)
1822.8809	1823.8801	-4	527	543	R.GIEQAVQSHAVAEEEAR.K			
1944.9826	1945.9845	-3	561	579	K.TSSAEMPTIPLGSAVEAIR.V			Oxidation (M)
1052.5138	1053.5187	-2	603	611	R.GVYSEETLR.A			
2002.0411	2003.0593	5	652	668	K.QLKPPAELYPEDINTFK.L			
1176.6251	1177.6276	-4	688	697	K.FVNQLKGESR.R			

Spot 103 (D2 20070719) – Inner membrane protein, mitochondrial, isoform CRA_a (Immt)

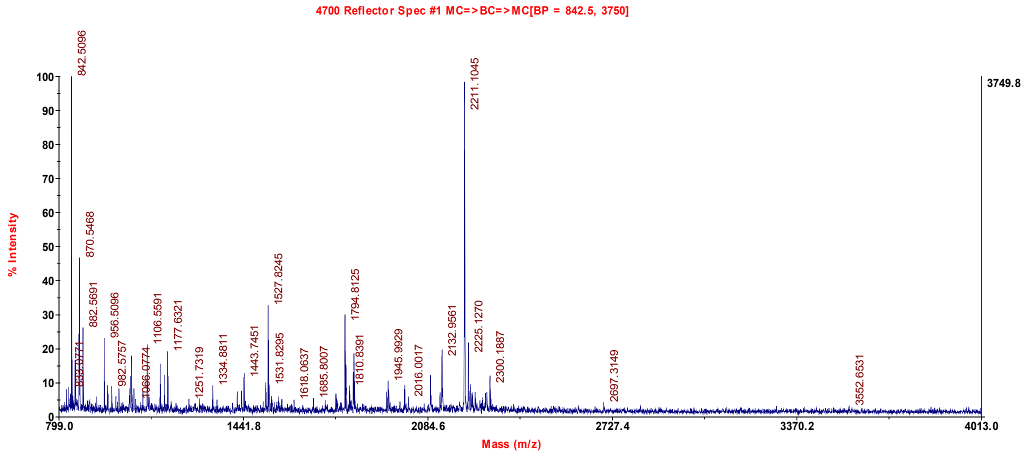

Spot 103 (20070719-D2) Immt (isoform CRA_a)								
PMF/MALDI								
Calculated	Observed	+/-ppm	start	end	Sequence	Ion Score	C.I.%	Modification
846.3773	847.3831	-2	67	72	K.WDSHFR.E			
1418.6579	1419.6589	-4	67	77	K.WDSHFRESVEK.T			
1517.7685	1518.7789	2	207	220	K.SLEDALNQTATVTR.Q			
1441.7889	1442.7845	-8	221	234	R.QTITAQNAAVQAVK.A			
1562.7828	1563.792	1	300	314	R.EIAGATPYITAAEEK.L			
1526.8205	1527.8245	-2	338	350	K.VVSQYHELVVQAR.D			
1536.826	1537.8195	-9	403	415	K.ATEKQHIELALER.Q			
1107.6036	1108.6051	-5	407	415	K.QHIELALER.Q			
1573.8325	1574.8322	-5	465	478	R.QAAAHTDHLRDVLK.V			
2125.0215	2126.03	1	479	495	K.VQEQELKFEFEQDLSEK.L			
1286.6255	1287.6522	15	496	505	K.LSEQELEFHR.R			
1442.7266	1443.7451	8	496	506	K.LSEQELEFHRR.S			
2272.0542	2273.0337	-12	506	524	R.RSQEQMDNFTLDINTAYAR.L			
2131.948	2132.9561	0	507	524	R.SQEQMDNFTLDINTAYAR.L			Oxidation (M)
2092.0661	2093.075	1	525	543	R.LRGIEQAVQSHAVAEEEAR.K			
1822.8809	1823.8853	-2	527	543	R.GIEQAVQSHAVAEEEAR.K			
1944.9826	1945.9929	2	561	579	K.TSSAEMPTIPLGSAVEAIR.V			Oxidation (M)
1052.5138	1053.5045	-16	603	611	R.GVYSEETLR.A			
981.5396	982.5757	29	612	619	R.ARFYAVQK.L			
2002.0411	2003.0355	-6	652	668	K.QLKPPAELYPEDINTFK.L			
1176.6251	1177.6321	0	688	697	K.FVNQLKGESR.R			

Spot 104 (A18 20080606) - Ferritin heavy chain (Fth1)

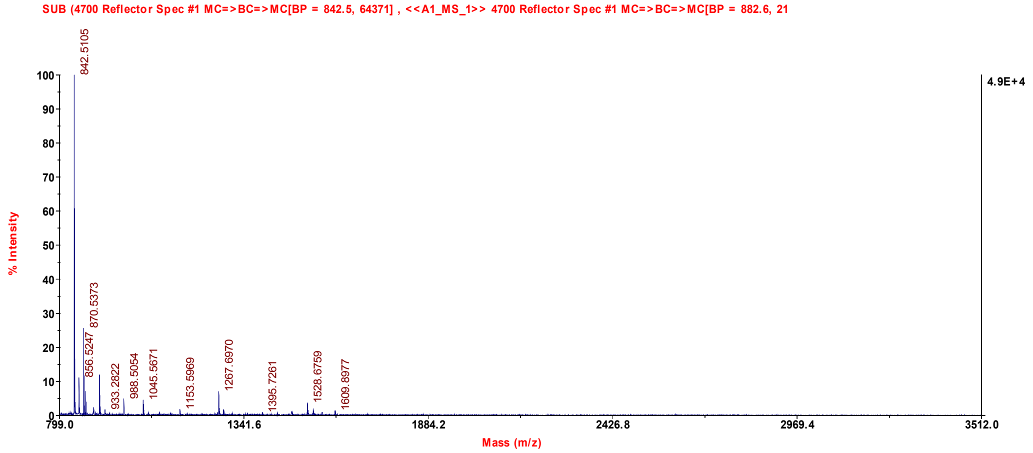

Spot 104 (20080606-A18) Fth1								
MS+MS/MS								
Calculated	Observed	+/-ppm	start	end	Sequence	Ion Score	C.I.%	Modification
876.5189	876.5205	2	81	87	IFLQDIK			
876.5189	876.5205	2	81	87	IFLQDIK	7	79.794	
1153.5959	1153.5964	0	148	157	ELGDHVTNLR			
1153.5959	1153.5964	0	148	157	ELGDHVTNLR			
1267.7004	1267.6987	-1	110	120	SVNQSLLELHK	50	99.999	
1267.7004	1267.6987	-1	110	120	SVNQSLLELHK			
1281.6909	1281.689	-1	148	158	ELGDHVTNLRK			
1345.6284	1345.6732	33	55	64	YFLHQSHEER			
1481.807	1481.8069	0	145	157	SIKELGDHVTNLR			
1545.7041	1545.6989	-3	11	23	QNYHQDSEAAINR	46	99.997	
1545.7041	1545.6989	-3	11	23	QNYHQDSEAAINR			

Spot 104 (20080606-A18) Fth1								
PMF/MALDI								
Calculated	Observed	+/-ppm	start	end	Sequence	Ion Score	C.I.%	Modification
945.488	946.4875	-8	2	10	M.TTASPSQVR.Q			
1544.6967	1545.702	-1	11	23	R.QNYHQDSEAAINR.Q			
1344.6211	1345.6412	10	55	64	K.YFLHQSHEER.E			
875.5116	876.52	1	81	87	R.IFLQDIK.K			
1266.6932	1267.697	-3	110	120	K.SVNQSLLELHK.L			
1480.7998	1481.808	1	145	157	K.SIKELGDHVTNLR.K			
1608.8947	1609.8977	-3	145	158	K.SIKELGDHVTNLRK.M			
1152.5887	1153.5969	1	148	157	K.ELGDHVTNLR.K			
1280.6837	1281.6816	-7	148	158	K.ELGDHVTNLRK.M			

Spot 106 (C2 20080606) - Isopentenyl-diphosphate Delta-isomerase 1 (Idi1)

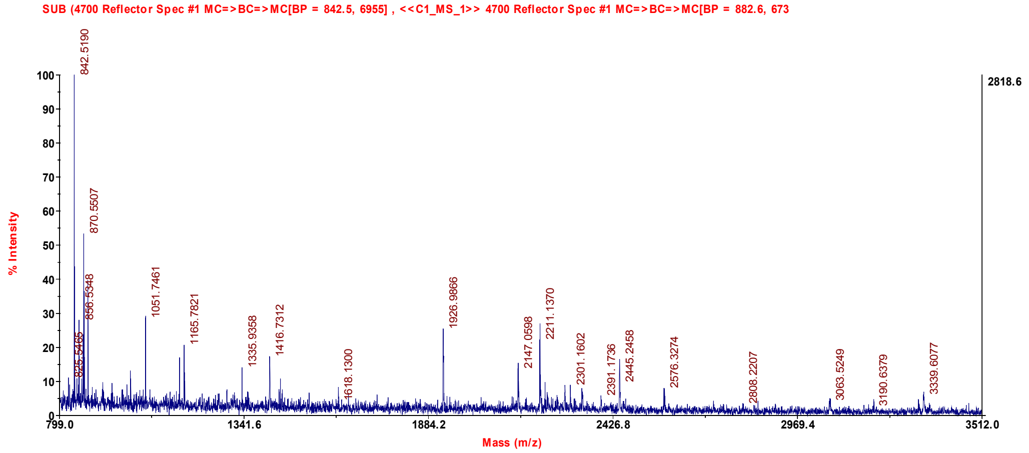

Spot 106 (20080606-C2) Idi1								
PMF/MALDI								
Calculated	Observed	+/-ppm	start	end	Sequence	Ion Score	C.I.%	Modification
2146.0286	2147.0598	11	13	30	K.QVQLLAEMCILIDENDNK.I			
1415.7085	1416.7312	11	53	64	R.AFSVFLFNTENK.L			
2334.1413	2335.1655	7	114	133	K.AELGIPLEEVDLNEMNYLTR.I			Oxidation (M)
2261.1117	2262.1028	-7	138	156	K.AQSDGIWGEHEIDYILFLR.K			

Spot 108 (P1014-18) – mixture of: Protein arginine N-methyltransferase 5 [Mus musculus] (Prmt5), Heat shock-related 70 kDa protein 2 (Hspa2) and Serum albumin (Alb)

**Spot 108 (P1014-18) Prmt5, Hspa2 & Alb**											
**LCMS/MS (Scaffold)**											
Sample Name	Acc No.	Proteins(s) inferred	No. Peptides identified	Sequence Coverage	Position in sequence	Peptide Sequence	Precursor m/z	Charge observed	Calculated mass (neutral)	Mascot score	Mascot Expect value
P1014-18	Q8CIG8	sp|Q8CIG8|Protein arginine N-methyltransferase 5 Tax_Id=10090 [Mus musculus]	3	3.61%	2 - 13	(M)aAmAVGGAGGSR(V)	531.62	2	1061.22	90.3	4.35E-006
					632 - 637	(R)SYTIGL(-)	653.14	1	652.13	40.5	1.66E-001
					2 - 18	(M)aAMAVGGAGGSRVSSGR(D)	766.74	2	1531.46	48.1	5.74E-002
	P14659	sp|P14659|Heat shock-related 70 kDa protein 2 Tax_Id=10116 [Rattus norvegicus]	1	3.32%	613 - 633	(K)LYQGGPGGGGSSGGPTIEEVD(-)	967.14	2	1932.27	93.8	1.29E-006
	ENSRNOP00000003921	ens|ENSRNOP00000003921|ENSRNOG00000002911 transcript:ENSRNOT00000003921 Tax_Id=10116 [Rattus norvegicus]	3	4.61%	550 - 558	(K)QTALAELVK(H)	486.68	2	971.34	62.0	2.94E-003
					549 - 558	(K)KQTALAELVK(H)	550.72	2	1099.43	52.6	2.45E-002
					435 - 452	(R)YTQKAPQVSTPTLVEAAR(N)	653.86	3	1958.56	55.1	1.23E-02

**Spot 108 [P1014-18 (75972 Rattus)] Hspa2**								
**LCMS/MS**								
Calculated	Observed	+/-ppm	start	end	Sequence	Ion Score	C.I.%	Modification
1992.9152	996.9389		79	94	K.FEDATVQSDMKHWPFR.V	6		
1786.9828	597.0084		173	189	R.IINEPTAAAIAYGLDKK.G	10		
1345.6812	449.4429		240	250	R.MVSHLAEEFKR.K	30		
1152.5597	577.3184		521	529	R.MVQEAERYK.S	11		
1932.8701	967.1394		613	633	K.LYQGGPGGGGSSGGPTIEEVD.-	81		
1932.8701	967.1399		613	633	K.LYQGGPGGGGSSGGPTIEEVD.-	94		

**Spot 108 [P1014-18 (75972 Rattus)] Alb**								
**LCMS/MS**								
Calculated	Observed	+/-ppm	start	end	Sequence	Ion Score	C.I.%	Modification
1526.5501	509.6814		76	88	K.TCVADENAENCDK.S	11		
698.4439	699.279		213	219	K.ALVAAVR.Q	15		
1327.5795	443.2209		220	229	R.QRMKCSSMQR.F	10		Oxidation (M)
1507.8048	503.8409		230	242	R.FGERAFKAWAVAR.M	19		
1018.5712	510.9404		234	242	R.AFKAWAVAR.M	31		
530.3064	532.858		282	286	R.AELAK.Y	6		
1959.0425	653.8612		435	452	R.YTQKAPQVSTPTLVEAAR.N	55		
1959.0425	654.8194		435	452	R.YTQKAPQVSTPTLVEAAR.N	24		
1099.6601	550.7199		549	558	K.KQTALAELVK.H	53		
971.5651	486.6784		550	558	K.QTALAELVK.H	62		
1293.7041	432.1389		559	569	K.HKPKATEDQLK.T	10		
1948.8949	650.4379		585	602	K.AADKDNCFATEGPNLVAR.S	44		
1948.8949	975.7209		585	602	K.AADKDNCFATEGPNLVAR.S	34		

Spot 110 (A9 20080606) - Protein DJ-1 (Park7, Cap1)

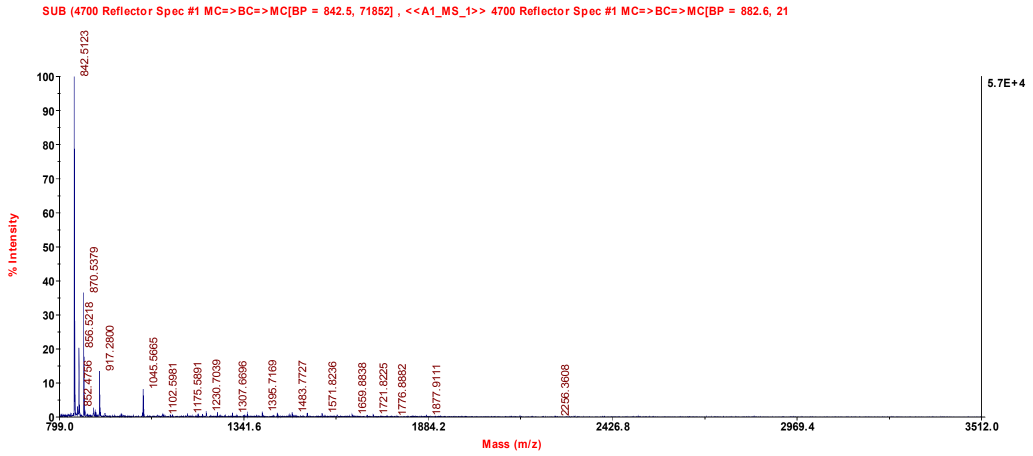

Spot 110 (20080606-A9) Park7, Cap1								
MS+MS/MS								
Calculated	Observed	+/-ppm	start	end	Sequence	Ion Score	C.I.%	Modification
852.4937	852.4921	-2	123	130	VTSHPLAK			
874.4992	874.5054	7	149	156	DGLILTSR	0	0	
874.4992	874.5054	7	149	156	DGLILTSR			
1230.7052	1230.7064	1	146	156	VEKDGLILTSR			
1230.7052	1230.7064	1	146	156	VEKDGLILTSR	18	97.931	
1721.8085	1721.822	8	13	27	GAEEMETVIPVDIMR			2 Oxidation (M)
1877.9095	1877.8989	-6	13	28	GAEEMETVIPVDIMRR			2 Oxidation (M)

Spot 110 (20080606-A9) Park7, Cap1								
PMF/MALDI								
Calculated	Observed	+/-ppm	start	end	Sequence	Ion Score	C.I.%	Modification
1720.8012	1721.8225	8	13	27	K.GAEEMETVIPVDIMR.R			2 Oxidation (M)
1876.9023	1877.9111	1	13	28	K.GAEEMETVIPVDIMRR.A			2 Oxidation (M)
1285.699	1286.7192	10	90	99	K.EILKEQENRK.G			
802.3933	803.4377	46	94	99	K.EQENRK.G			
851.4865	852.4756	-21	123	130	K.VTSHPLAK.D			
1229.6979	1230.7039	-1	146	156	R.VEKDGLILTSR.G			
873.492	874.5	1	149	156	K.DGLILTSR.G			

Spot 113 (C17 20080606) - Aldehyde dehydrogenase, mitochondrial precursor (Aldh2) and/or ATP synthase subunit alpha, mitochondrial precursor (Chain A, Rat Liver F1-Atpase) (Atp5a1)

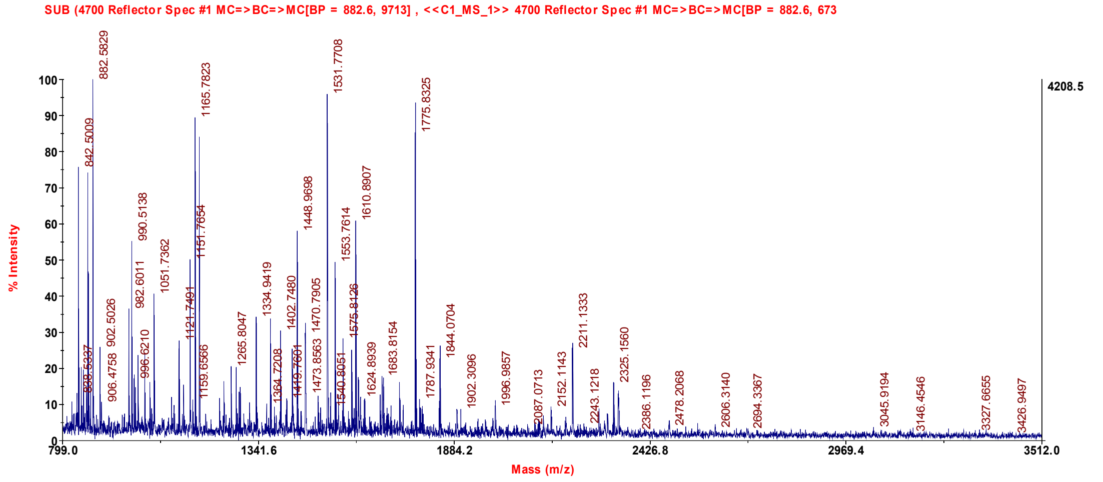

Spot 113 (20080606-C17) Aldh2								
MS+MS/MS								
Calculated	Observed	+/-ppm	start	end	Sequence	Ion Score	C.I.%	Modification
902.4941	902.498	4	431	438	TIEEVVGR			
990.5002	990.5061	6	349	357	VVGNPFDSR			
1177.6575	1177.6609	3	429	438	FKTIEEVVGR			
1177.6575	1177.6609	3	429	438	FKTIEEVVGR			
1402.7227	1402.722	0	84	96	AAQAAFQLGSPWR			
1470.7627	1470.7615	-1	397	409	GYFIQPTVFGDVK			
1531.7428	1531.7408	-1	162	174	TIPIDGDFFSYTR			
1531.7428	1531.7408	-1	162	174	TIPIDGDFFSYTR	25	99.824	
1599.79	1599.7871	-2	495	508	ELGEYGLQAYTEVK			
1775.8123	1775.7914	-12	327	340	TFVQEDVYDEFVER	32	99.963	
1775.8123	1775.7914	-12	327	340	TFVQEDVYDEFVER			
1844.0527	1844.0297	-12	212	228	VAEQTPLTALYVANLIK			
1844.0527	1844.0297	-12	212	228	VAEQTPLTALYVANLIK			
2152.1028	2152.0618	-19	410	428	DGMTIAKEEIFGPVMQILK			2 Oxidation (M)

Spot 113 (20080606-C17) Aldh2								
PMF/MALDI								
Calculated	Observed	+/-ppm	start	end	Sequence	Ion Score	C.I.%	Modification
1699.9158	1700.8511	-42	81	96	K.AVKAAQAAFQLGSPWR.R			
1401.7153	1402.748	18	84	96	K.AAQAAFQLGSPWR.R			
1373.803	1374.7617	-35	106	116	R.LLYRLADLIER.D			
1099.5985	1100.6288	21	110	118	R.LADLIERDR.T			
1457.6728	1458.7109	21	150	161	R.YYAGWADKYHGK.T			
1530.7355	1531.7708	18	162	174	K.TIPIDGDFFSYTR.H			
1368.7799	1369.7545	-24	198	211	K.LGPALATGNVVVMK.V			
1384.7748	1385.8029	15	198	211	K.LGPALATGNVVVMK.V			Oxidation (M)
1843.0455	1844.0704	10	212	228	K.VAEQTPLTALYVANLIK.E			
1774.805	1775.8325	11	327	340	R.TFVQEDVYDEFVER.S			
1232.6262	1233.6631	24	347	357	K.SRVVGNPFDSR.T			
989.4931	990.5138	14	349	357	R.VVGNPFDSR.T			
2477.1823	2478.2068	7	349	370	R.VVGNPFDSRTEQGPQVDETQFK.K			
1505.6998	1506.7349	18	358	370	R.TEQGPQVDETQFK.K			
1633.7948	1634.8286	16	358	371	R.TEQGPQVDETQFKK.I			
1469.7555	1470.7905	19	397	409	R.GYFIQPTVFGDVK.D			
2151.0956	2152.1143	5	410	428	K.DGMTIAKEEIFGPVMQILK.F			2 Oxidation (M)
1418.7479	1419.7601	3	417	428	K.EEIFGPVMQILK.F			Oxidation (M)
1176.6503	1177.6763	16	429	438	K.FKTIEEVVGR.A			
901.4869	902.5026	9	431	438	K.TIEEVVGR.A			
1415.7368	1416.6857	-41	431	443	K.TIEEVVGRANNSK.Y			
1039.5702	1040.6001	22	444	453	K.YGLAAAVFTK.D			
2190.0263	2191.0471	6	489	508	K.MSGSGRELGEYGLQAYTEVK.T			Oxidation (M)
1598.7828	1599.8193	18	495	508	R.ELGEYGLQAYTEVK.T			

Spot 113 (20080606-C17) Atp5a1								
MS+MS/MS								
Calculated	Observed	+/-ppm	start	end	Sequence	Ion Score	C.I.%	Modification
1026.5942	1026.5986	4	195	204	AVDSLVPIGR			
1120.7201	1120.7275	7	172	182	VGLKAPGIIPR			
1374.741	1374.7386	-2	183	194	ISVREPMQTGIK			Oxidation (M)
1548.8679	1548.9358	44	317	329	QAVAYRQMSLLLR			
1553.7383	1553.7302	-5	335	347	EAYPGDVFYLHSR			
1562.9125	1562.9498	24	74	88	VLSIGDGIARVHGLR			
1575.786	1575.7811	-3	59	73	ILGADTSVDLEETGR			
1610.8748	1610.8588	-10	134	149	TGAIVDVPVGDELLGR			
1610.8748	1610.8588	-10	134	149	TGAIVDVPVGDELLGR			
1683.7894	1683.7787	-6	89	103	NVQAEEMVEFSSGLK			Oxidation (M)
2325.1543	2325.0901	-28	473	493	QGQYSPMAIEEQVAVIYAGVR			Oxidation (M)
2338.1672	2338.0771	-39	442	463	EVAAFAQFGSDLDAATQQLLSR			

Spot 113 (20080606-C17) Atp5a1								
PMF/MALDI								
Calculated	Observed	+/-ppm	start	end	Sequence	Ion Score	C.I.%	Modification
1791.9036	1792.8829	-16	43	58	R.LQKTGTAEMSSILEER.I			
1574.7788	1575.8126	17	59	73	R.ILGADTSVDLEETGR.V			
999.5713	1000.5901	12	74	83	R.VLSIGDGIAR.V			
1682.7821	1683.8154	15	89	103	R.NVQAEEMVEFSSGLK.G			Oxidation (M)
1609.8676	1610.8907	10	134	149	R.TGAIVDVPVGDELLGR.V			
1119.7128	1120.7435	21	172	182	R.VGLKAPGIIPR.I			
1373.7337	1374.7617	15	183	194	R.ISVREPMQTGIK.A			Oxidation (M)
1025.5869	1026.6107	16	195	204	K.AVDSLVPIGR.G			
1315.7347	1316.7632	16	219	230	K.TSIAIDTIINQK.R			
1471.8358	1472.8187	-17	219	231	K.TSIAIDTIINQKR.F			
1700.8733	1701.8809	0	302	316	R.DNGKHALIIYDDLSK.Q			
1286.687	1287.7264	25	306	316	K.HALIIYDDLSK.Q			
875.4899	876.4999	3	323	329	R.QMSLLLR.R			Oxidation (M)
1552.731	1553.7614	15	335	347	R.EAYPGDVFYLHSR.L			
1134.5815	1135.6057	15	417	427	R.VGSAAQTRAMK.Q			Oxidation (M)
1622.845	1623.8705	11	428	441	K.QVAGTMKLELAQYR.E			Oxidation (M)
2337.1601	2338.1392	-12	442	463	R.EVAAFAQFGSDLDAATQQLLSR.G			
2324.1471	2325.156	1	473	493	K.QGQYSPMAIEEQVAVIYAGVR.G			Oxidation (M)

Spot 124 (D10 20070719) – Protein disulfide-isomerase A3 (Pdia3)

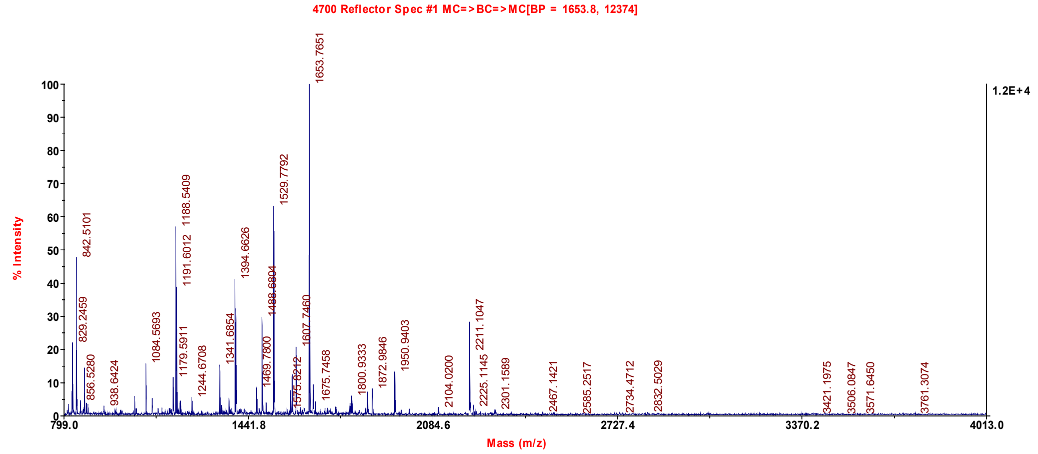

Spot 124 (20070719-D10) Pdia3								
**MS+MS/MS**								
Calculated	Observed	+/-ppm	start	end	Sequence	Ion Score	C.I.%	Modification
866.4617	866.4686	8	416	422	YKELGEK			
877.489	877.4948	7	297	304	LNFAVASR			
938.6396	938.6415	2	74	82	LKGIVPLAK			
995.5632	995.5713	8	131	140	QAGPASVPLR			
1084.5674	1084.5696	2	95	104	YGVSGYPTLK			
1168.6685	1168.6838	13	120	130	TADGIVSHLKK			
1179.5865	1179.5922	5	174	183	AASNLRDNYR			
1188.5354	1188.5417	5	336	344	FVMQEEFSR			Oxidation (M)
1188.5354	1188.5417	5	336	344	FVMQEEFSR	19	98.726	Oxidation (M)
1191.6005	1191.6027	2	63	73	LAPEYEAAATR			
1197.7201	1197.7302	8	423	433	LSKDPNIVIAK			
1244.6633	1244.6726	7	184	194	FAHTNVESLVK			
1341.6837	1341.6868	2	449	460	GFPTIYFSPANK			
1341.6837	1341.6868	2	449	460	GFPTIYFSPANK	61	100	
1347.7015	1347.7002	-1	62	73	RLAPEYEAAATR			
1373.6736	1373.6775	3	352	362	FLQEYFDGNLK			
1394.6587	1394.6625	3	162	173	DLFSDGHSEFLK	56	100	
1394.6587	1394.6625	3	162	173	DLFSDGHSEFLK			
1397.7059	1397.7092	2	472	482	ELNDFISYLQR			
1469.7787	1469.7808	1	449	461	GFPTIYFSPANKK			
1488.6787	1488.683	3	336	347	FVMQEEFSRDGK			Oxidation (M)
1488.6787	1488.683	3	336	347	FVMQEEFSRDGK			Oxidation (M)
1502.6943	1502.7043	7	333	344	GEKFVMQEEFSR			Oxidation (M)
1529.7747	1529.7817	5	352	363	FLQEYFDGNLKR			
1529.7747	1529.7817	5	352	363	FLQEYFDGNLKR	69	100	
1587.8165	1587.826	6	148	161	FISDKDASVVGFFR			
1593.8483	1593.8506	1	483	496	EATNPPIIQEEKPK			
1593.8483	1593.8506	1	483	496	EATNPPIIQEEKPK	21	99.247	
1607.7476	1607.749	1	259	271	DLLTAYYDVDYEK			
1607.7476	1607.749	1	259	271	DLLTAYYDVDYEK	30	99.899	
1652.7662	1652.7681	1	105	119	IFRDGEEAGAYDGPR			
1652.7662	1652.7681	1	105	119	IFRDGEEAGAYDGPR	25	99.722	
1721.9432	1721.9537	6	483	497	EATNPPIIQEEKPKK			
1800.9377	1800.9374	0	364	379	YLKSEPIPETNEGPVK			
1850.0382	1850.0476	5	483	498	EATNPPIIQEEKPKKK			
1872.9813	1872.9875	3	131	147	QAGPASVPLRTEDEFKK			
1950.9331	1950.9434	5	259	274	DLLTAYYDVDYEKNTK			
1950.9331	1950.9434	5	259	274	DLLTAYYDVDYEKNTK	95	100	

Spot 124 (20070719-D10) Pdia3								
PMF/MALDI								
Calculated	Observed	+/-ppm	start	end	Sequence	Ion Score	C.I.%	Modification
1346.6942	1347.6978	-3	62	73	K.RLAPEYEAAATR.L			
1190.5931	1191.6012	1	63	73	R.LAPEYEAAATR.L			
937.6324	938.6424	3	74	82	R.LKGIVPLAK.V			
1083.5601	1084.5693	2	95	104	K.YGVSGYPTLK.I			
1651.759	1652.7646	-1	105	119	K.IFRDGEEAGAYDGPR.T			
1039.5662	1040.5782	5	120	129	R.TADGIVSHLK.K			
1167.6612	1168.6859	15	120	130	R.TADGIVSHLKK.Q			
994.556	995.5738	11	131	140	K.QAGPASVPLR.T			
1743.8791	1744.8855	-1	131	146	K.QAGPASVPLRTEDEFK.K			
1586.8093	1587.8254	6	148	161	K.FISDKDASVVGFFR.D			
1393.6514	1394.6626	3	162	173	R.DLFSDGHSEFLK.A			
1178.5792	1179.5911	4	174	183	K.AASNLRDNYR.F			
1243.6561	1244.6708	6	184	194	R.FAHTNVESLVK.E			
1606.7403	1607.746	-1	259	271	K.DLLTAYYDVDYEK.N			
1949.9258	1950.9403	4	259	274	K.DLLTAYYDVDYEKNTK.G			
1124.5363	1125.5905	42	272	280	K.NTKGSNYWR.N			
979.4943	980.5265	25	281	288	R.NRVMMVAK.T			2 Oxidation (M)
1745.9213	1746.9218	-4	289	304	K.TFLDAGHKLNFAVASR.K			
876.4817	877.4951	7	297	304	K.LNFAVASR.K			
2604.3072	2605.2864	-11	306	329	K.TFSHELSDFGLESTTGEIPVVAIR.T			
1501.6871	1502.7021	5	333	344	K.GEKFVMQEEFSR.D			Oxidation (M)
1171.5332	1172.5309	-8	336	344	K.FVMQEEFSR.D			
1187.5281	1188.5409	5	336	344	K.FVMQEEFSR.D			Oxidation (M)
1471.6766	1472.7472	43	336	347	K.FVMQEEFSRDGK.A			
1487.6715	1488.6804	1	336	347	K.FVMQEEFSRDGK.A			Oxidation (M)
1372.6663	1373.6779	3	352	362	R.FLQEYFDGNLK.R			
1528.7674	1529.7792	3	352	363	R.FLQEYFDGNLKR.Y			
1799.9305	1800.9334	-2	364	379	R.YLKSEPIPETNEGPVK.V			
865.4545	866.4681	7	416	422	K.YKELGEK.L			
1196.7128	1197.7245	4	423	433	K.LSKDPNIVIAK.M			
1340.6765	1341.6854	1	449	460	K.GFPTIYFSPANK.K			
1468.7714	1469.78	1	449	461	K.GFPTIYFSPANKK.L			
1396.6987	1397.7069	1	472	482	R.ELNDFISYLQR.E			
1592.8409	1593.8491	1	483	496	R.EATNPPIIQEEKPK.K			
1720.9359	1721.9529	6	483	497	R.EATNPPIIQEEKPKK.K			

Spot 124 (A21 20080606) - Protein disulfide-isomerase A3 (Pdia3)

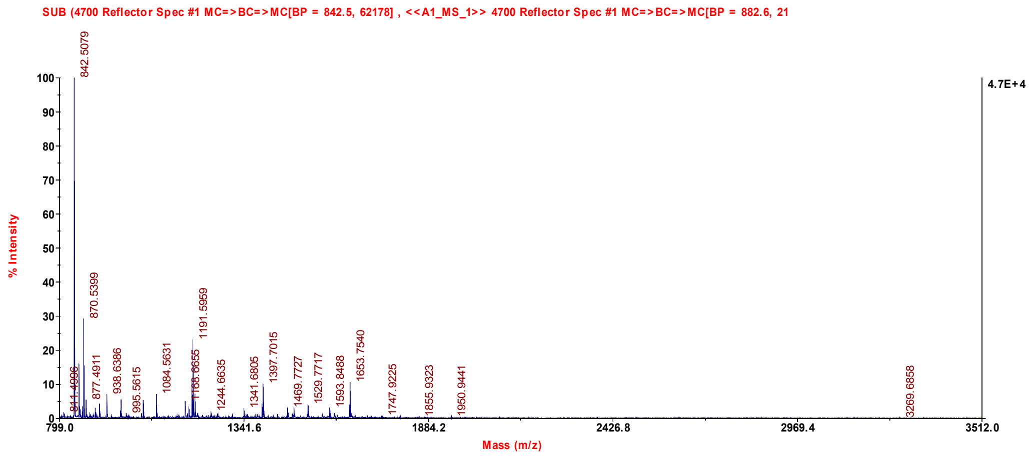

Spot 124 (20080606-A21) Pdia3								
MS+MS/MS								
Calculated	Observed	+/-ppm	start	end	Sequence	Ion Score	C.I.%	Modification
866.4617	866.4694	9	416	422	YKELGEK	2	5.539	
866.4617	866.4694	9	416	422	YKELGEK			
877.489	877.4922	4	297	304	LNFAVASR			
877.489	877.4922	4	297	304	LNFAVASR			
888.4573	888.4609	4	289	296	TFLDAGHK			
938.6396	938.6386	-1	74	82	LKGIVPLAK	18	97.583	
938.6396	938.6386	-1	74	82	LKGIVPLAK			
951.5509	951.5461	-5	219	226	IVAYTEKK			
980.5015	980.5021	1	281	288	NRVMMVAK			2 Oxidation (M)
995.5632	995.5636	0	131	140	QAGPASVPLR			
1040.5735	1040.574	0	120	129	TADGIVSHLK			
1084.5674	1084.5657	-2	95	104	YGVSGYPTLK			
1084.5674	1084.5657	-2	95	104	YGVSGYPTLK	35	99.954	
1168.6685	1168.6705	2	120	130	TADGIVSHLKK	18	97.75	
1168.6685	1168.6705	2	120	130	TADGIVSHLKK			
1179.5865	1179.5856	-1	174	183	AASNLRDNYR			
1179.5865	1179.5856	-1	174	183	AASNLRDNYR			
1188.5354	1188.5331	-2	336	344	FVMQEEFSR			Oxidation (M)
1191.6005	1191.5986	-2	63	73	LAPEYEAAATR			
1191.6005	1191.5986	-2	63	73	LAPEYEAAATR	41	99.989	
1197.7201	1197.7175	-2	423	433	LSKDPNIVIAK			
1197.7201	1197.7175	-2	423	433	LSKDPNIVIAK			
1244.6633	1244.6604	-2	184	194	FAHTNVESLVK			
1341.6837	1341.6836	0	449	460	GFPTIYFSPANK			
1347.7015	1347.6963	-4	62	73	RLAPEYEAAATR			
1394.6587	1394.6566	-2	162	173	DLFSDGHSEFLK			
1397.7059	1397.7043	-1	472	482	ELNDFISYLQR			
1397.7059	1397.7043	-1	472	482	ELNDFISYLQR	22	99.033	
1469.7787	1469.7772	-1	449	461	GFPTIYFSPANKK			
1469.7787	1469.7772	-1	449	461	GFPTIYFSPANKK	28	99.756	
1488.6787	1488.6748	-3	336	347	FVMQEEFSRDGK			Oxidation (M)
1529.7747	1529.7716	-2	352	363	FLQEYFDGNLKR			
1529.7747	1529.7716	-2	352	363	FLQEYFDGNLKR	38	99.979	
1587.8165	1587.8143	-1	148	161	FISDKDASVVGFFR			
1593.8483	1593.8505	1	483	496	EATNPPIIQEEKPK			
1593.8483	1593.8505	1	483	496	EATNPPIIQEEKPK	6	64.169	
1607.7476	1607.7432	-3	259	271	DLLTAYYDVDYEK			
1652.7472	1652.755	5	434	448	MDATANDVPSPYEVK			Oxidation (M)
1652.7662	1652.755	-7	105	119	IFRDGEEAGAYDGPR	11	89.948	

Spot 124 (20080606-A21) Pdia3								
PMF/MALDI								
Calculated	Observed	+/-ppm	start	end	Sequence	Ion Score	C.I.%	Modification
1346.6942	1347.6969	-3	62	73	K.RLAPEYEAAATR.L			
1190.5931	1191.5959	-4	63	73	R.LAPEYEAAATR.L			
937.6324	938.6387	-1	74	82	R.LKGIVPLAK.V			
1083.5601	1084.5631	-4	95	104	K.YGVSGYPTLK.I			
1235.5054	1236.5073	-4	108	119	R.DGEEAGAYDGPR.T			
1039.5662	1040.5709	-2	120	129	R.TADGIVSHLK.K			
1167.6612	1168.6655	-2	120	130	R.TADGIVSHLKK.Q			
994.556	995.5616	-2	131	140	K.QAGPASVPLR.T			
1586.8093	1587.8108	-4	148	161	K.FISDKDASVVGFFR.D			
1393.6514	1394.6555	-2	162	173	R.DLFSDGHSEFLK.A			
1178.5792	1179.5848	-1	174	183	K.AASNLRDNYR.F			
1243.6561	1244.6635	0	184	194	R.FAHTNVESLVK.E			
950.5437	951.5482	-3	219	226	K.IVAYTEKK.M			
1606.7403	1607.7469	0	259	271	K.DLLTAYYDVDYEK.N			
1949.9258	1950.9441	6	259	274	K.DLLTAYYDVDYEKNTK.G			
979.4943	980.5002	-1	281	288	R.NRVMMVAK.T			2 Oxidation (M)
887.4501	888.4617	5	289	296	K.TFLDAGHK.L			
876.4817	877.4911	2	297	304	K.LNFAVASR.K			
1485.6922	1486.7593	40	333	344	K.GEKFVMQEEFSR.D			
1187.5281	1188.5309	-4	336	344	K.FVMQEEFSR.D			Oxidation (M)
1487.6715	1488.6713	-5	336	347	K.FVMQEEFSRDGK.A			Oxidation (M)
1528.7674	1529.7716	-2	352	363	R.FLQEYFDGNLKR.Y			
1799.9305	1800.9391	1	364	379	R.YLKSEPIPETNEGPVK.V			
1395.6882	1396.6897	-4	367	379	K.SEPIPETNEGPVK.V			
865.4545	866.4642	3	416	422	K.YKELGEK.L			
1196.7128	1197.7148	-4	423	433	K.LSKDPNIVIAK.M			
1651.7399	1652.7528	3	434	448	K.MDATANDVPSPYEVK.G			Oxidation (M)
1340.6765	1341.6805	-2	449	460	K.GFPTIYFSPANK.K			
1468.7714	1469.7727	-4	449	461	K.GFPTIYFSPANKK.L			
1396.6987	1397.7015	-3	472	482	R.ELNDFISYLQR.E			
1592.8409	1593.8488	0	483	496	R.EATNPPIIQEEKPK.K			

Spot 130 (P1014-06) – 14-3-3 protein epsilon (Ywhae)

Spot 130 (P1014-06) Ywhae											
LCMS/MS (Scaffold)											
Sample Name	Acc No.	Proteins(s) inferred	No. Peptides identified	Sequence Coverage	Position in sequence	Peptide Sequence	Precursor m/z	Charge observed	Calculated mass (neutral)	Mascot score	Mascot Expect value
P1014-06	P62260	sp|P62260|14-3-3 protein epsilon Tax_Id=10116 [Rattus norvegicus]	1	4.71%	1 - 12	(-)mDDREDLVYQAK(L)	762.70	2	1523.38	48.6	5.12E-002

Spot 132 (B12 20080606) - Peroxiredoxin-6 (Prdx6)

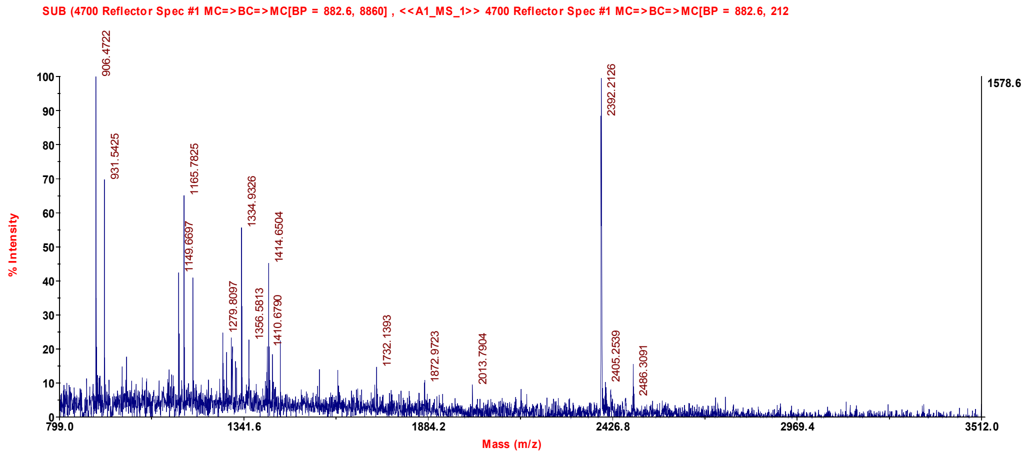

Spot 132 (20080606-B12) Prdx6								
MS+MS/MS								
Calculated	Observed	+/-ppm	start	end	Sequence	Ion Score	C.I.%	Modification
906.4679	906.4673	-1	156	162	NFDEILR			
906.4679	906.4673	-1	156	162	NFDEILR	2	62.253	
931.5359	931.5396	4	57	64	LAPEFAKR			
1045.604	1045.5707	-32	54	63	AAKLAPEFAK			
1149.6666	1149.6783	10	133	142	VVFIFGPDKK			
1191.6732	1191.6832	8	145	155	LSILYPATTGR			
2392.2256	2392.2173	-3	2	24	PGGLLLGDEAPNFEANTTIGHIR	55	100	
2392.2256	2392.2173	-3	2	24	PGGLLLGDEAPNFEANTTIGHIR			

Spot 132 (20080606-B12) Prdx6								
PMF/MALDI								
Calculated	Observed	+/-ppm	start	end	Sequence	Ion Score	C.I.%	Modification
2391.2183	2392.2126	-5	2	24	M.PGGLLLGDEAPNFEANTTIGHIR.F			
930.5287	931.5425	7	57	64	K.LAPEFAKR.N			
1148.6594	1149.6697	3	133	142	R.VVFIFGPDKK.L			
1190.6659	1191.6793	5	145	155	K.LSILYPATTGR.N			
905.4607	906.4722	5	156	162	R.NFDEILR.V			
1871.955	1872.9723	5	183	199	K.KGESVMVLPTLPEEEAK.Q			Oxidation (M)
2485.3138	2486.3091	-5	183	204	K.KGESVMVLPTLPEEEAKQLFPK.G			Oxidation (M)

Spot 137 (D2 20080606) – mixture of: 14-3-3 protein beta/alpha (Ywhab) and 14-3-3 protein zeta/delta (Ywhaz)

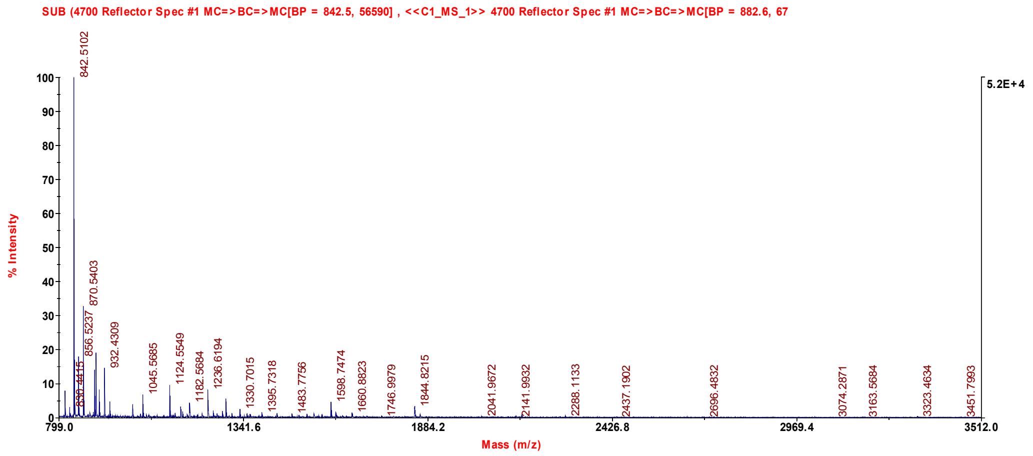

Spot 137 (20080606-D2) Ywhab								
PMF/MALDI								
Calculated	Observed	+/-ppm	start	end	Sequence	Ion Score	C.I.%	Modification
1014.5458	1015.5552	2	12	20	K.AKLAEQAER.Y			
815.4137	816.4211	0	14	20	K.LAEQAER.Y			
1843.808	1844.8215	3	14	29	K.LAEQAERYDDMAAAMK.A			2 Oxidation (M)
1597.7332	1598.7474	4	30	43	K.AVTEQGHELSNEER.N			
906.5174	907.5248	0	44	51	R.NLLSVAYK.N			
1502.8569	1503.8656	1	44	57	R.NLLSVAYKNVVGAR.R			
1658.958	1659.8936	-43	44	58	R.NLLSVAYKNVVGARR.S			
902.5073	903.5159	1	63	70	R.VISSIEQK.T			
1288.6987	1289.7085	2	63	73	R.VISSIEQKTER.N			
1659.8791	1660.8823	-2	63	76	R.VISSIEQKTERNEK.K			
1182.5815	1183.5779	-9	77	85	K.KQQMGKEYR.E			Oxidation (M)
1054.4866	1055.4932	-1	78	85	K.QQMGKEYR.E			Oxidation (M)
1358.7194	1359.7356	7	106	117	K.YLILNATHAESK.V			
931.4222	932.4309	2	123	129	K.MKGDYFR.Y			Oxidation (M)
1181.5564	1182.5684	4	130	140	R.YLSEVASGDNK.Q			
2158.0178	2159.0479	11	141	159	K.QTTVSNSQQAYQEAFEISK.K			
2286.1128	2287.1699	22	141	160	K.QTTVSNSQQAYQEAFEISKK.E			
1235.6445	1236.6194	-26	160	169	K.KEMQPTHPIR.L			
1251.6394	1252.6422	-4	160	169	K.KEMQPTHPIR.L			Oxidation (M)
1123.5444	1124.5549	3	161	169	K.EMQPTHPIR.L			Oxidation (M)
1204.6485	1205.6566	1	215	224	K.DSTLIMQLLR.D			Oxidation (M)

Spot 137 (20080606-D2) Ywhaz								
PMF/MALDI								
Calculated	Observed	+/-ppm	start	end	Sequence	Ion Score	C.I.%	Modification
1014.5458	1015.5552	2	10	18	K.AKLAEQAER.Y			
815.4137	816.4211	0	12	18	K.LAEQAER.Y			
1547.7063	1548.7156	1	28	41	K.SVTEQGAELSNEER.N			
2436.2132	2437.1902	-12	28	49	K.SVTEQGAELSNEERNLLSVAYK.N			
906.5174	907.5248	0	42	49	R.NLLSVAYK.N			
1502.8569	1503.8656	1	42	55	R.NLLSVAYKNVVGAR.R			
1658.958	1659.8936	-43	42	56	R.NLLSVAYKNVVGARR.S			
888.4916	889.496	-3	61	68	R.VVSSIEQK.T			
1503.778	1504.8334	32	61	74	R.VVSSIEQKTEGAEK.K			
1631.873	1632.8765	-2	61	75	R.VVSSIEQKTEGAEKK.Q			
1329.6928	1330.7015	1	104	115	K.FLIPNASQPESK.V			
1597.7963	1598.7474	-35	116	127	K.VFYLKMKGDYYR.Y			Oxidation (M)
931.4222	932.4309	2	121	127	K.MKGDYYR.Y			
947.4171	948.4271	3	121	127	K.MKGDYYR.Y			Oxidation (M)
1278.6456	1279.6515	-1	128	139	R.YLAEVAAGDDKK.G			
2039.98	2040.9906	2	140	157	K.GIVDQSQQAYQEAFEISK.K			
1235.6445	1236.6194	-26	158	167	K.KEMQPTHPIR.L			
1251.6394	1252.6422	-4	158	167	K.KEMQPTHPIR.L			Oxidation (M)
1123.5444	1124.5549	3	159	167	K.EMQPTHPIR.L			Oxidation (M)
1204.6485	1205.6566	1	213	222	K.DSTLIMQLLR.D			Oxidation (M)

Spot 141 (D11 20080606) - Ubiquitin-conjugating enzyme E2N (Ube2n)

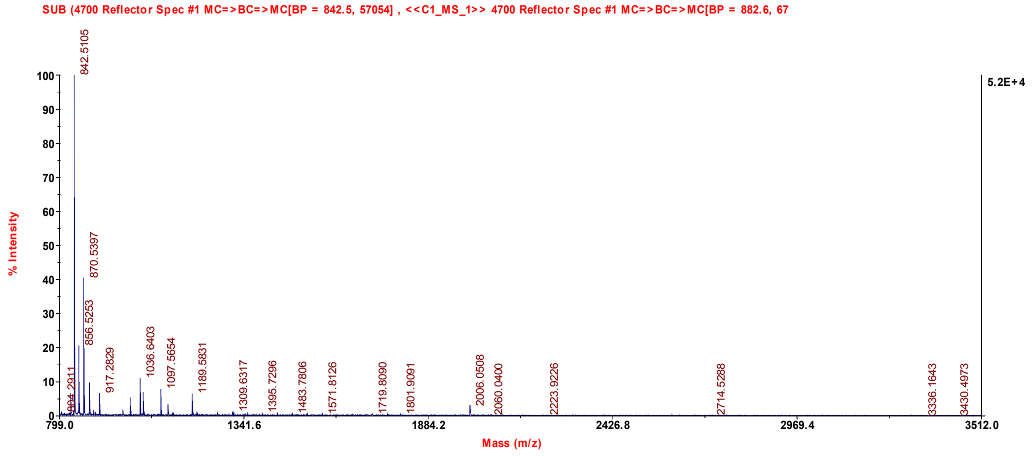

Spot 141 (20080606-D11) Ube2n								
PMF/MALDI								
Calculated	Observed	+/-ppm	start	end	Sequence	Ion Score	C.I.%	Modification
886.5236	887.5328	2	8	14	R.IIKETQR.L			
1035.6328	1036.6403	0	15	24	R.LLAEPVPGIK.A			
2005.048	2006.0508	-2	15	33	R.LLAEPVPGIKAEPDESNAR.Y			
1746.8902	1747.9059	5	54	68	K.LELFLPEEYPMAAPK.V			
1762.8851	1763.8965	2	54	68	K.LELFLPEEYPMAAPK.V			Oxidation (M)
984.5029	985.5074	-3	75	82	K.IYHPNVDK.L			
1310.7095	1311.6727	-34	75	85	K.IYHPNVDKLGR.I			
1188.5734	1189.5831	2	131	141	K.SNEAQAIETAR.A			

Spot 142 (A17 20080606) - mixture of: Protein disulfide-isomerase A6 (Pdia6), Tubulin alpha-1A chain (Tuba1a), Tubulin alpha-1B (Tuba1b), Tubulin alpha-1C (Tuba1c), and Tubulin alpha-3 (Tuba3)

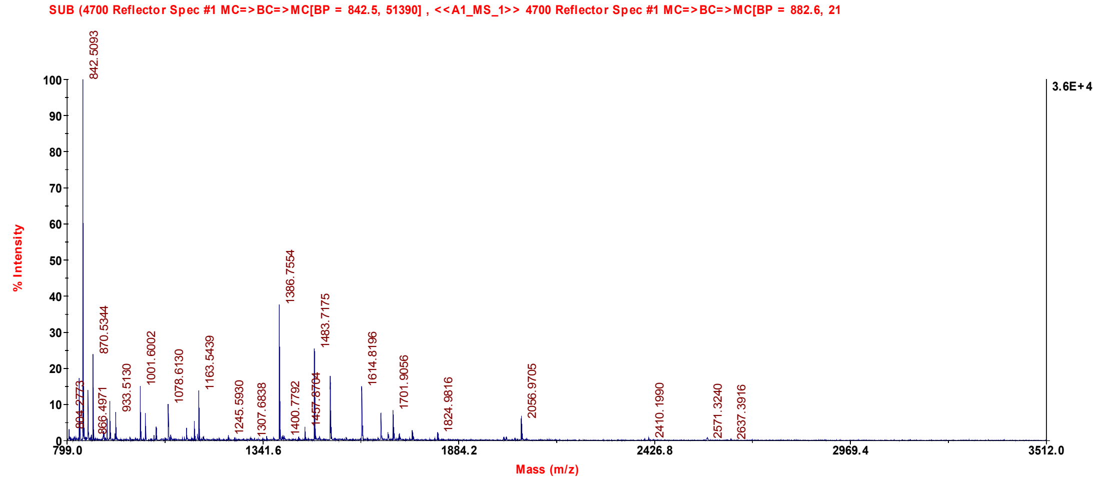

Spot 142 (20080606-A17) Pdia6								
MS+MS/MS								
Calculated	Observed	+/-ppm	start	end	Sequence	Ion Score	C.I.%	Modification
850.5219	850.5365	17	367	373	MKFALLK			
866.5168	866.516	-1	367	373	MKFALLK			Oxidation (M)
901.5141	901.5116	-3	61	67	LTPEWKK			
933.5112	933.5127	2	257	264	TRSDIVSR			
1001.5989	1001.6018	3	68	77	AASALKDVVK	42	99.992	
1001.5989	1001.6018	3	68	77	AASALKDVVK			
1078.6143	1078.6139	0	314	322	NSYLEVLLK	31	99.907	
1078.6143	1078.6139	0	314	322	NSYLEVLLK			
1123.651	1123.6462	-4	232	241	YGIKGFPTIK			
1151.4963	1151.4963	0	246	256	GESPVDYDGGR			
1163.5439	1163.5427	-1	109	118	NKPEDYQGGR			
1163.5439	1163.5427	-1	109	118	NKPEDYQGGR	64	100	
1386.7587	1386.756	-2	119	132	TGEAIVDAALSALR	76	100	
1386.7587	1386.756	-2	119	132	TGEAIVDAALSALR			
1483.7175	1483.7181	0	374	386	GSFSEQGINEFLR	49	99.998	
1483.7175	1483.7181	0	374	386	GSFSEQGINEFLR			
1527.8489	1527.8445	-3	217	231	LAAVDATVNQVLASR	43	99.994	
1527.8489	1527.8445	-3	217	231	LAAVDATVNQVLASR			
1614.8234	1614.8203	-2	393	409	GSTAPVGGGSFPNITPR			
1614.8234	1614.8203	-2	393	409	GSTAPVGGGSFPNITPR	34	99.943	
1667.8024	1667.8011	-1	242	256	IFQKGESPVDYDGGR	38	99.979	
1667.8024	1667.8011	-1	242	256	IFQKGESPVDYDGGR			
1755.0123	1755.0106	-1	215	231	VKLAAVDATVNQVLASR			
2015.0079	2014.9926	-8	195	212	NLEPEWAAAATEVKEQTK			
2571.3313	2571.3171	-6	78	102	VGAVNADKHQSLGGQYGVQGFPTIK			
2637.3657	2637.4011	13	265	288	ALDLFSDNAPPPELLEIINEDIAK			

Spot 142 (20080606-A17) Pdia6								
PMF/MALDI								
Calculated	Observed	+/-ppm	start	end	Sequence	Ion Score	C.I.%	Modification
900.5069	901.5107	-4	61	67	R.LTPEWKK.A			
1000.5917	1001.6002	1	68	77	K.AASALKDVVK.V			
2570.3242	2571.324	-3	78	102	K.VGAVNADKHQSLGGQYGVQGFPTIK.I			
1815.9268	1816.9316	-1	86	102	K.HQSLGGQYGVQGFPTIK.I			
1162.5367	1163.5439	0	109	118	K.NKPEDYQGGR.T			
1385.7514	1386.7554	-2	119	132	R.TGEAIVDAALSALR.Q			
2014.0007	2014.9895	-9	195	212	K.NLEPEWAAAATEVKEQTK.G			
1754.005	1755.009	-2	215	231	K.VKLAAVDATVNQVLASR.Y			
1526.8416	1527.8455	-2	217	231	K.LAAVDATVNQVLASR.Y			
1666.7951	1667.8004	-1	242	256	K.IFQKGESPVDYDGGR.T			
1150.4891	1151.496	0	246	256	K.GESPVDYDGGR.T			
932.5039	933.513	2	257	264	R.TRSDIVSR.A			
2636.3585	2637.3916	10	265	288	R.ALDLFSDNAPPPELLEIINEDIAK.K			
1077.607	1078.613	-1	314	322	R.NSYLEVLLK.L			
865.5095	866.4971	-23	367	373	K.MKFALLK.G			Oxidation (M)
1482.7103	1483.7175	0	374	386	K.GSFSEQGINEFLR.E			
1613.8162	1614.8196	-2	393	409	R.GSTAPVGGGSFPNITPR.E			

Spot 142 (20070719-D9) Pdia6								
MS+MS/MS								
Calculated	Observed	+/-ppm	start	end	Sequence	Ion Score	C.I.%	Modification
901.5141	901.512	-2	61	67	LTPEWKK			
933.5112	933.508	-3	257	264	TRSDIVSR			
1001.5989	1001.588	-11	68	77	AASALKDVVK			
1078.6143	1078.6113	-3	314	322	NSYLEVLLK			
1123.651	1123.6239	-24	232	241	YGIKGFPTIK			
1151.4963	1151.4943	-2	246	256	GESPVDYDGGR			
1163.5439	1163.5404	-3	109	118	NKPEDYQGGR	29	99.856	
1163.5439	1163.5404	-3	109	118	NKPEDYQGGR			
1386.7587	1386.7555	-2	119	132	TGEAIVDAALSALR			
1386.7587	1386.7555	-2	119	132	TGEAIVDAALSALR	19	98.545	
1483.7175	1483.7114	-4	374	386	GSFSEQGINEFLR			
1483.7175	1483.7114	-4	374	386	GSFSEQGINEFLR	37	99.978	
1527.8489	1527.8403	-6	217	231	LAAVDATVNQVLASR	2	31.314	
1527.8489	1527.8403	-6	217	231	LAAVDATVNQVLASR			
1528.7642	1528.7972	22	195	208	NLEPEWAAAATEVK			
1614.8234	1614.8156	-5	393	409	GSTAPVGGGSFPNITPR			
1614.8234	1614.8156	-5	393	409	GSTAPVGGGSFPNITPR	46	99.997	
1667.8024	1667.7954	-4	242	256	IFQKGESPVDYDGGR			
1667.8024	1667.7954	-4	242	256	IFQKGESPVDYDGGR	77	100	
1793.8928	1793.8906	-1	103	118	IFGANKNKPEDYQGGR			
1816.9341	1816.9204	-8	86	102	HQSLGGQYGVQGFPTIK			
2015.0079	2015.0028	-3	195	212	NLEPEWAAAATEVKEQTK			
2015.0079	2015.0028	-3	195	212	NLEPEWAAAATEVKEQTK	88	100	
2200.1243	2200.1133	-5	195	214	NLEPEWAAAATEVKEQTKGK			
2571.3313	2571.3267	-2	78	102	VGAVNADKHQSLGGQYGVQGFPTIK			
2571.3313	2571.3267	-2	78	102	VGAVNADKHQSLGGQYGVQGFPTIK	2	35.157	

Spot 142 (20070719-D9) Pdia6								
PMF/MALDI								
Calculated	Observed	+/-ppm	start	end	Sequence	Ion Score	C.I.%	Modification
900.5069	901.5105	-4	61	67	R.LTPEWKK.A			
1000.5917	1001.5868	-12	68	77	K.AASALKDVVK.V			
2570.3242	2571.335	1	78	102	K.VGAVNADKHQSLGGQYGVQGFPTIK.I			
1815.9268	1816.9236	-6	86	102	K.HQSLGGQYGVQGFPTIK.I			
1792.8856	1793.8934	0	103	118	K.IFGANKNKPEDYQGGR.T			
1162.5367	1163.542	-2	109	118	K.NKPEDYQGGR.T			
1385.7514	1386.7562	-2	119	132	R.TGEAIVDAALSALR.Q			
1527.7569	1528.8004	24	195	208	K.NLEPEWAAAATEVK.E			
2014.0007	2015.0076	0	195	212	K.NLEPEWAAAATEVKEQTK.G			
1526.8416	1527.842	-5	217	231	K.LAAVDATVNQVLASR.Y			
1122.6437	1123.6227	-25	232	241	R.YGIKGFPTIK.I			
1666.7951	1667.8	-1	242	256	K.IFQKGESPVDYDGGR.T			
1150.4891	1151.4943	-2	246	256	K.GESPVDYDGGR.T			
932.5039	933.5059	-6	257	264	R.TRSDIVSR.A			
1077.607	1078.6105	-4	314	322	R.NSYLEVLLK.L			
849.5146	850.5498	33	367	373	K.MKFALLK.G			
1482.7103	1483.7151	-2	374	386	K.GSFSEQGINEFLR.E			
1613.8162	1614.8201	-2	393	409	R.GSTAPVGGGSFPNITPR.E			
2326.1342	2327.1636	9	393	415	R.GSTAPVGGGSFPNITPREPWDGK.D			

Spot 142 (20080606-A17) Tuba1a								
MS+MS/MS								
Calculated	Observed	+/-ppm	start	end	Sequence	Ion Score	C.I.%	Modification
909.504	909.5071	3	157	164	LSVDYGKK			
909.504	909.5071	3	157	164	LSVDYGKK	14	94.592	
1015.5782	1015.5756	-3	327	336	DVNAAIATIK	27	99.751	
1015.5782	1015.5756	-3	327	336	DVNAAIATIK			
1085.6201	1085.6171	-3	113	121	EIIDLVLDR			
1396.693	1396.7179	18	391	401	LDHKFDLMYAK			Oxidation (M)
1457.8685	1457.8682	0	230	243	LIGQIVSSITASLR			
1701.9058	1701.9041	-1	65	79	AVFVDLEPTVIDEVR	43	99.994	
1701.9058	1701.9041	-1	65	79	AVFVDLEPTVIDEVR			
1718.8821	1718.8796	-1	216	229	NLDIERPTYTNLNR			
1756.9633	1756.9862	13	265	280	IHFPLATYAPVISAEK			
1824.9854	1824.9821	-2	353	370	VGINYQPPTVVPGGDLAK			
2007.8929	2007.9025	5	41	60	TIGGGDDSFNTFFSETGAGK			

Spot 142 (20080606-A17) Tuba1a								
PMF/MALDI								
Calculated	Observed	+/-ppm	start	end	Sequence	Ion Score	C.I.%	Modification
2006.8858	2007.9072	7	41	60	K.TIGGGDDSFNTFFSETGAGK.H			
1700.8985	1701.9056	0	65	79	R.AVFVDLEPTVIDEVR.T			
1084.6128	1085.6172	-3	113	121	K.EIIDLVLDR.I			
908.4967	909.5056	2	157	164	R.LSVDYGKK.S			
1717.8747	1718.8787	-2	216	229	R.NLDIERPTYTNLNR.L			
1456.8613	1457.8704	1	230	243	R.LIGQIVSSITASLR.F			
2408.2012	2409.1812	-11	244	264	R.FDGALNVDLTEFQTNLVPYPR.I			
1755.9559	1756.9847	12	265	280	R.IHFPLATYAPVISAEK.A			
1014.5709	1015.5756	-3	327	336	K.DVNAAIATIK.T			
1823.9782	1824.9816	-2	353	370	K.VGINYQPPTVVPGGDLAK.V			

Spot 142 (20080606-A17) Tuba1b								
MS+MS/MS								
Calculated	Observed	+/-ppm	start	end	Sequence	Ion Score	C.I.%	Modification
909.504	909.5071	3	157	164	LSVDYGKK			
909.504	909.5071	3	157	164	LSVDYGKK	14	94.592	
1015.5782	1015.5756	-3	327	336	DVNAAIATIK	27	99.751	
1015.5782	1015.5756	-3	327	336	DVNAAIATIK			
1085.6201	1085.6171	-3	113	121	EIIDLVLDR			
1396.693	1396.7179	18	391	401	LDHKFDLMYAK			Oxidation (M)
1701.9058	1701.9041	-1	65	79	AVFVDLEPTVIDEVR	43	99.994	
1701.9058	1701.9041	-1	65	79	AVFVDLEPTVIDEVR			
1718.8821	1718.8796	-1	216	229	NLDIERPTYTNLNR			
1756.9633	1756.9862	13	265	280	IHFPLATYAPVISAEK			
1824.9854	1824.9821	-2	353	370	VGINYQPPTVVPGGDLAK			
2007.8929	2007.9025	5	41	60	TIGGGDDSFNTFFSETGAGK			

Spot 142 (20080606-A17) Tuba1b								
PMF/MALDI								
Calculated	Observed	+/-ppm	start	end	Sequence	Ion Score	C.I.%	Modification
2006.8858	2007.9072	7	41	60	K.TIGGGDDSFNTFFSETGAGK.H			
1700.8985	1701.9056	0	65	79	R.AVFVDLEPTVIDEVR.T			
1084.6128	1085.6172	-3	113	121	K.EIIDLVLDR.I			
908.4967	909.5056	2	157	164	R.LSVDYGKK.S			
1717.8747	1718.8787	-2	216	229	R.NLDIERPTYTNLNR.L			
1486.8719	1487.8765	-2	230	243	R.LISQIVSSITASLR.F			
2408.2012	2409.1812	-11	244	264	R.FDGALNVDLTEFQTNLVPYPR.I			
1755.9559	1756.9847	12	265	280	R.IHFPLATYAPVISAEK.A			
1014.5709	1015.5756	-3	327	336	K.DVNAAIATIK.T			
1823.9782	1824.9816	-2	353	370	K.VGINYQPPTVVPGGDLAK.V			

Spot 142 (20080606-A17) Tuba1c								
MS+MS/MS								
Calculated	Observed	+/-ppm	start	end	Sequence	Ion Score	C.I.%	Modification
909.504	909.5071	3	157	164	LSVDYGKK			
909.504	909.5071	3	157	164	LSVDYGKK	14	94.592	
1015.5782	1015.5756	-3	327	336	DVNAAIATIK	27	99.751	
1015.5782	1015.5756	-3	327	336	DVNAAIATIK			
1085.6201	1085.6171	-3	113	121	EIIDLVLDR			
1396.693	1396.7179	18	391	401	LDHKFDLMYAK			Oxidation (M)
1701.9058	1701.9041	-1	65	79	AVFVDLEPTVIDEVR	43	99.994	
1701.9058	1701.9041	-1	65	79	AVFVDLEPTVIDEVR			
1718.8821	1718.8796	-1	216	229	NLDIERPTYTNLNR			
1756.9633	1756.9862	13	265	280	IHFPLATYAPVISAEK			
2007.8929	2007.9025	5	41	60	TIGGGDDSFNTFFSETGAGK			

Spot 142 (20080606-A17) Tuba1c								
PMF/MALDI								
Calculated	Observed	+/-ppm	start	end	Sequence	Ion Score	C.I.%	Modification
2006.8858	2007.9072	7	41	60	K.TIGGGDDSFNTFFSETGAGK.H			
1700.8985	1701.9056	0	65	79	R.AVFVDLEPTVIDEVR.T			
1084.6128	1085.6172	-3	113	121	K.EIIDLVLDR.I			
908.4967	909.5056	2	157	164	R.LSVDYGKK.S			
1717.8747	1718.8787	-2	216	229	R.NLDIERPTYTNLNR.L			
1486.8719	1487.8765	-2	230	243	R.LISQIVSSITASLR.F			
2408.2012	2409.1812	-11	244	264	R.FDGALNVDLTEFQTNLVPYPR.I			
1755.9559	1756.9847	12	265	280	R.IHFPLATYAPVISAEK.A			
1014.5709	1015.5756	-3	327	336	K.DVNAAIATIK.T			

Spot 142 (20080606-A17) Tuba3								
MS+MS/MS								
Calculated	Observed	+/-ppm	start	end	Sequence	Ion Score	C.I.%	Modification
909.504	909.5071	3	157	164	LSVDYGKK			
909.504	909.5071	3	157	164	LSVDYGKK	14	94.592	
1015.5782	1015.5756	-3	327	336	DVNAAIATIK	27	99.751	
1015.5782	1015.5756	-3	327	336	DVNAAIATIK			
1071.6045	1071.6034	-1	113	121	EIVDLVLDR			
1396.693	1396.7179	18	391	401	LDHKFDLMYAK			Oxidation (M)
1457.8685	1457.8682	0	230	243	LIGQIVSSITASLR			
1687.8901	1687.8824	-5	65	79	AVFVDLEPTVVDEVR			
1718.8821	1718.8796	-1	216	229	NLDIERPTYTNLNR			
1756.9633	1756.9862	13	265	280	IHFPLATYAPVISAEK			
1824.9854	1824.9821	-2	353	370	VGINYQPPTVVPGGDLAK			
2007.8929	2007.9025	5	41	60	TIGGGDDSFNTFFSETGAGK			

Spot 142 (20080606-A17) Tuba3								
PMF/MALDI								
Calculated	Observed	+/-ppm	start	end	Sequence	Ion Score	C.I.%	Modification
2006.8858	2007.9072	7	41	60	K.TIGGGDDSFNTFFSETGAGK.H			
1686.8829	1687.8845	-3	65	79	R.AVFVDLEPTVVDEVR.T			
908.4967	909.5056	2	157	164	R.LSVDYGKK.S			
1717.8747	1718.8787	-2	216	229	R.NLDIERPTYTNLNR.L			
1456.8613	1457.8704	1	230	243	R.LIGQIVSSITASLR.F			
2408.2012	2409.1812	-11	244	264	R.FDGALNVDLTEFQTNLVPYPR.I			
1755.9559	1756.9847	12	265	280	R.IHFPLATYAPVISAEK.A			
1014.5709	1015.5756	-3	327	336	K.DVNAAIATIK.T			
1823.9782	1824.9816	-2	353	370	K.VGINYQPPTVVPGGDLAK.V			

Spot 142 (D9 20070719) – Protein disulfide-isomerase A6 (Pdi6)

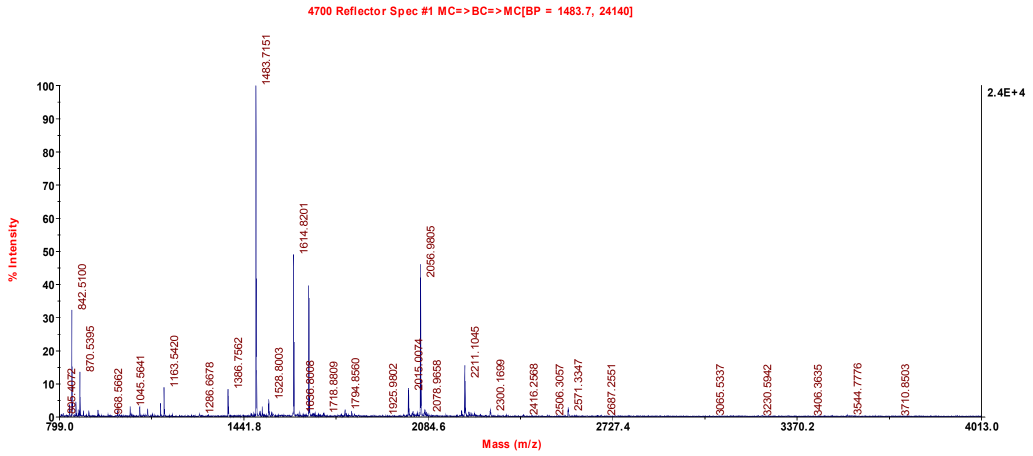

Spot 142 (20070719-D9) Pdia6								
MS+MS/MS								
Calculated	Observed	+/-ppm	start	end	Sequence	Ion Score	C.I.%	Modification
901.5141	901.512	-2	61	67	LTPEWKK			
933.5112	933.508	-3	257	264	TRSDIVSR			
1001.5989	1001.588	-11	68	77	AASALKDVVK			
1078.6143	1078.6113	-3	314	322	NSYLEVLLK			
1123.651	1123.6239	-24	232	241	YGIKGFPTIK			
1151.4963	1151.4943	-2	246	256	GESPVDYDGGR			
1163.5439	1163.5404	-3	109	118	NKPEDYQGGR	29	99.856	
1163.5439	1163.5404	-3	109	118	NKPEDYQGGR			
1386.7587	1386.7555	-2	119	132	TGEAIVDAALSALR			
1386.7587	1386.7555	-2	119	132	TGEAIVDAALSALR	19	98.545	
1483.7175	1483.7114	-4	374	386	GSFSEQGINEFLR			
1483.7175	1483.7114	-4	374	386	GSFSEQGINEFLR	37	99.978	
1527.8489	1527.8403	-6	217	231	LAAVDATVNQVLASR	2	31.314	
1527.8489	1527.8403	-6	217	231	LAAVDATVNQVLASR			
1528.7642	1528.7972	22	195	208	NLEPEWAAAATEVK			
1614.8234	1614.8156	-5	393	409	GSTAPVGGGSFPNITPR			
1614.8234	1614.8156	-5	393	409	GSTAPVGGGSFPNITPR	46	99.997	
1667.8024	1667.7954	-4	242	256	IFQKGESPVDYDGGR			
1667.8024	1667.7954	-4	242	256	IFQKGESPVDYDGGR	77	100	
1793.8928	1793.8906	-1	103	118	IFGANKNKPEDYQGGR			
1816.9341	1816.9204	-8	86	102	HQSLGGQYGVQGFPTIK			
2015.0079	2015.0028	-3	195	212	NLEPEWAAAATEVKEQTK			
2015.0079	2015.0028	-3	195	212	NLEPEWAAAATEVKEQTK	88	100	
2200.1243	2200.1133	-5	195	214	NLEPEWAAAATEVKEQTKGK			
2571.3313	2571.3267	-2	78	102	VGAVNADKHQSLGGQYGVQGFPTIK			
2571.3313	2571.3267	-2	78	102	VGAVNADKHQSLGGQYGVQGFPTIK	2	35.157	

Spot 142 (20070719-D9) Pdia6								
PMF/MALDI								
Calculated	Observed	+/-ppm	start	end	Sequence	Ion Score	C.I.%	Modification
900.5069	901.5105	-4	61	67	R.LTPEWKK.A			
1000.5917	1001.5868	-12	68	77	K.AASALKDVVK.V			
2570.3242	2571.335	1	78	102	K.VGAVNADKHQSLGGQYGVQGFPTIK.I			
1815.9268	1816.9236	-6	86	102	K.HQSLGGQYGVQGFPTIK.I			
1792.8856	1793.8934	0	103	118	K.IFGANKNKPEDYQGGR.T			
1162.5367	1163.542	-2	109	118	K.NKPEDYQGGR.T			
1385.7514	1386.7562	-2	119	132	R.TGEAIVDAALSALR.Q			
1527.7569	1528.8004	24	195	208	K.NLEPEWAAAATEVK.E			
2014.0007	2015.0076	0	195	212	K.NLEPEWAAAATEVKEQTK.G			
1526.8416	1527.842	-5	217	231	K.LAAVDATVNQVLASR.Y			
1122.6437	1123.6227	-25	232	241	R.YGIKGFPTIK.I			
1666.7951	1667.8	-1	242	256	K.IFQKGESPVDYDGGR.T			
1150.4891	1151.4943	-2	246	256	K.GESPVDYDGGR.T			
932.5039	933.5059	-6	257	264	R.TRSDIVSR.A			
1077.607	1078.6105	-4	314	322	R.NSYLEVLLK.L			
849.5146	850.5498	33	367	373	K.MKFALLK.G			
1482.7103	1483.7151	-2	374	386	K.GSFSEQGINEFLR.E			
1613.8162	1614.8201	-2	393	409	R.GSTAPVGGGSFPNITPR.E			
2326.1342	2327.1636	9	393	415	R.GSTAPVGGGSFPNITPREPWDGK.D			

Spot 145 (P1014-15) – Aldose reductase (Akr1b1)

Spot 145 (P1014-15) Akr1b1												
**LCMS/MS (Scaffold)**												
Sample Name	Acc No.	Proteins(s) inferred	No. Peptides identified	Sequence Coverage	Position in sequence	Peptide Sequence	Precursor m/z	Charge observed	Calculated mass (neutral)	Mascot score	Mascot Expect value
P1014-15	P07943	sp|P07943|Aldose reductase Tax_Id=10116 [Rattus norvegicus]	1	4.43%	156 - 169	(K)AIGVSNFNPLQIER(I)	779.20	2	1556.38	56.5	8.30E-003

Spot 148 (E14 20070719) – Ferritin light chain 1 (Ftl1)

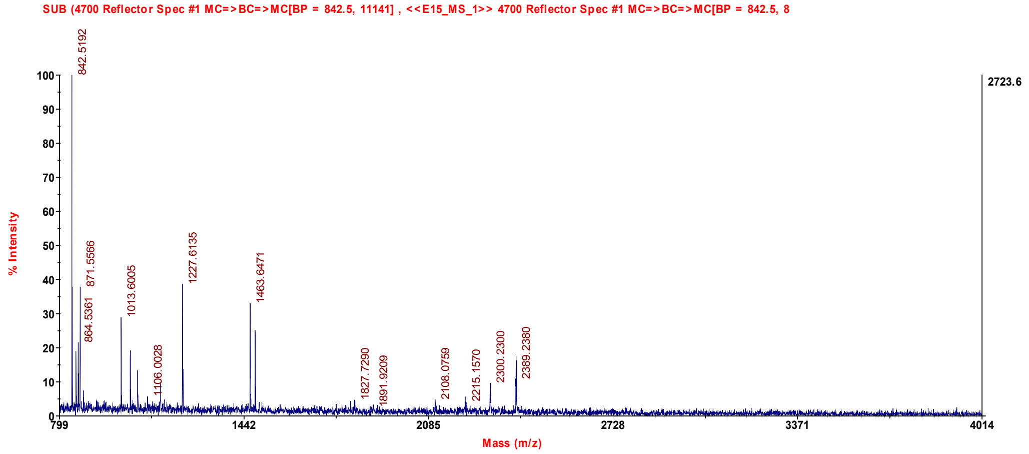

Spot 148 (20070719-E14) Ftl1								
PMF/MALDI								
Calculated	Observed	+/-ppm	start	end	Sequence	Ion Score	C.I.%	Modification
1479.6954	1480.6689	-23	7	19	R.QNYSTEVEAAVNR.L			
863.5341	864.5361	-6	20	26	R.LVNLHLR.A			
1012.6029	1013.6005	-10	66	73	R.LLKLQNER.G			
1070.5291	1071.5176	-18	145	153	K.MGNHLTNLR.R			Oxidation (M)
1226.6302	1227.6135	-20	145	154	K.MGNHLTNLRR.V			Oxidation (M)
2388.2074	2389.238	10	155	177	R.VAGPQPAQTGVAQASLGEYLFER.L			

Spot 150 (C6 20080606) – mixture of: rCG61762, isoform CRA_f (similar to: Splicing factor, arginine/serine-rich 7) (rCG61762) and Splicing factor, arginine/serine-rich 7 (Sfrs7)

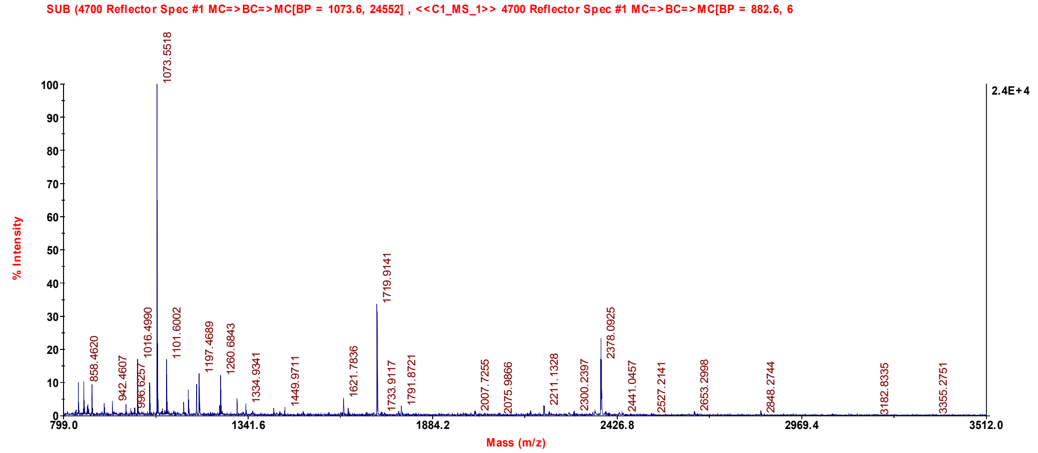

Spot 150 (20080606-C6) rCG61762								
PMF/MALDI								
Calculated	Observed	+/-ppm	start	end	Sequence	Ion Score	C.I.%	Modification
2354.1866	2355.1855	-4	7	29	R.YGGETKVYVGNLGTGAGKGELER.A			
1718.8951	1719.9141	7	13	29	K.VYVGNLGTGAGKGELER.A			
1072.5342	1073.5516	10	30	38	R.AFSYYGPLR.T			
1620.7573	1621.7834	12	45	58	R.NPPGFAFVEFEDPR.D			
2377.0975	2378.0925	-5	45	65	R.NPPGFAFVEFEDPRDAEDAVR.G			
2847.3464	2848.2744	-28	45	70	R.NPPGFAFVEFEDPRDAEDAVRGLDGK.V			
1259.6656	1260.6842	9	77	87	R.VRVELSTGMPR.R			Oxidation (M)
1415.7667	1416.7821	6	77	88	R.VRVELSTGMPRR.S			Oxidation (M)
1256.685	1257.7036	9	88	97	R.RSRFDRPPAR.R			
1100.5839	1101.6002	8	89	97	R.SRFDRPPAR.R			
857.4508	858.462	5	91	97	R.FDRPPAR.R			
1015.4835	1016.499	8	98	105	R.RPFDPNDR.C			
941.4903	942.4607	-39	134	141	R.SHSRSRGR.R			

Spot 150 (20080606-C6) Sfrs7								
PMF/MALDI								
Calculated	Observed	+/-ppm	start	end	Sequence	Ion Score	C.I.%	Modification
2354.1866	2355.1855	-4	7	29	R.YGGETKVYVGNLGTGAGKGELER.A			
1718.8951	1719.9141	7	13	29	K.VYVGNLGTGAGKGELER.A			
1072.5342	1073.5516	10	30	38	R.AFSYYGPLR.T			
1620.7573	1621.7834	12	45	58	R.NPPGFAFVEFEDPR.D			
2377.0975	2378.0925	-5	45	65	R.NPPGFAFVEFEDPRDAEDAVR.G			
2847.3464	2848.2744	-28	45	70	R.NPPGFAFVEFEDPRDAEDAVRGLDGK.V			
1259.6656	1260.6842	9	77	87	R.VRVELSTGMPR.R			Oxidation (M)
1415.7667	1416.7821	6	77	88	R.VRVELSTGMPRR.S			Oxidation (M)
1256.685	1257.7036	9	88	97	R.RSRFDRPPAR.R			
1100.5839	1101.6002	8	89	97	R.SRFDRPPAR.R			
857.4508	858.462	5	91	97	R.FDRPPAR.R			
1015.4835	1016.499	8	98	105	R.RPFDPNDR.C			

Spot 154 (D12 20080606) - RuvB-like 1 (Ruvbl1)

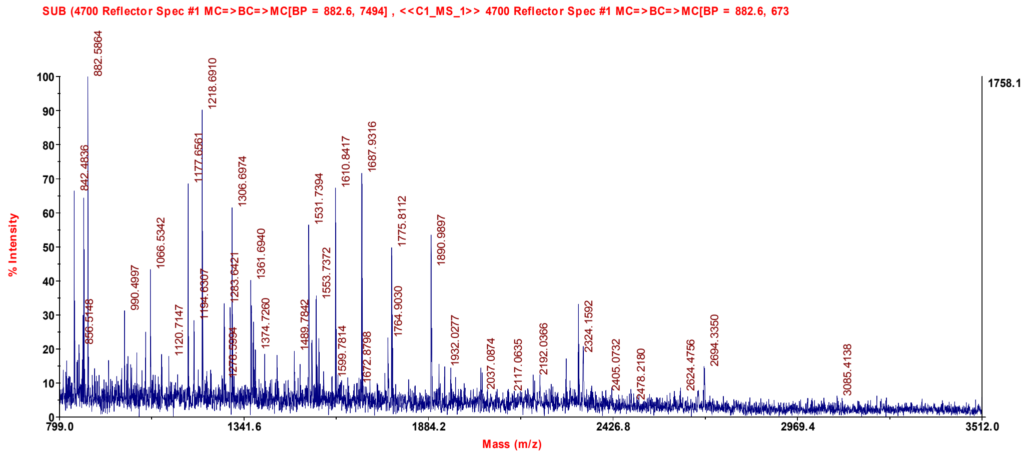

Spot 154 (20080606-D12) Ruvbl1								
PMF/MALDI								
Calculated	Observed	+/-ppm	start	end	Sequence	Ion Score	C.I.%	Modification
1299.6531	1300.667	5	34	46	K.QAASGLVGQENAR.E			
1437.7398	1438.7488	1	108	118	K.KTEVLMENFRR.A			Oxidation (M)
1931.0728	1932.0277	-27	166	182	K.GTKQLKLDPSIFESLQK.E			
1930.0523	1931.032	-14	169	184	K.QLKLDPSIFESLQKER.V			
1560.8147	1561.8115	-7	172	184	K.LDPSIFESLQKER.V			
1733.8836	1734.913	13	185	201	R.VEAGDVIYIEANSGAVK.R			
1889.9847	1890.9897	-1	185	202	R.VEAGDVIYIEANSGAVKR.Q			
1686.9304	1687.9316	-4	318	333	R.ALESSIAPIVIFASNR.G			
1947.9902	1948.9852	-6	340	357	R.GTEDITSPHGIPLDLLDR.V			
1754.8947	1755.8873	-8	363	376	R.TMLYTPQEMKQIIK.I			2 Oxidation (M)
2323.1655	2324.1592	-6	379	400	R.AQTEGINISEEALNHLGEIGTK.T			
1367.6755	1368.6757	-5	446	456	K.ILADQQDKYMK.-			Oxidation (M)

Spot 160 (C7 20080606) – Thimet oligopeptidase (Thop1) and/or Probable ATP-dependent RNA helicase DDX4 (Ddx4)

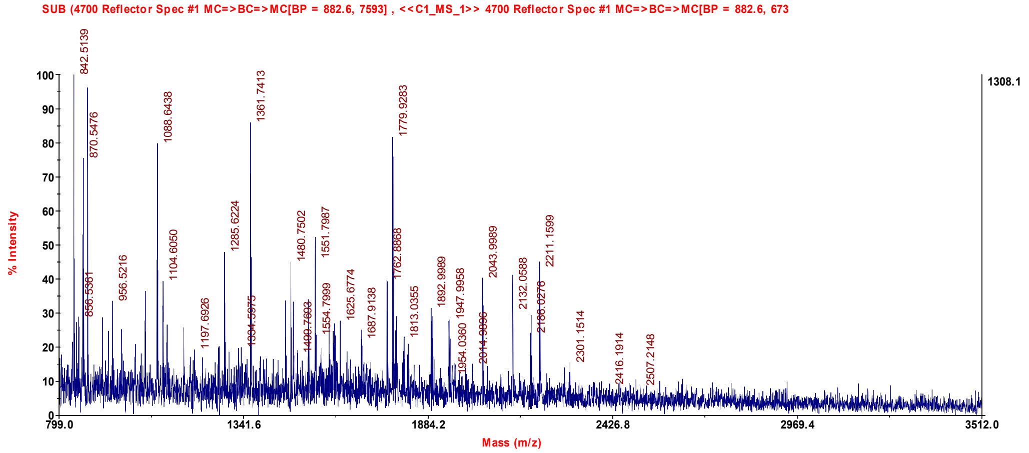

Spot 160 (20080606-C7) Thop1								
PMF/MALDI								
Calculated	Observed	+/-ppm	start	end	Sequence	Ion Score	C.I.%	Modification
1115.5723	1116.5919	11	26	34	R.WDLSAQQIR.A			
1786.8261	1787.8387	3	51	66	R.VGAQDFEDVSYESTLK.A			
1463.762	1464.7814	8	67	79	K.ALADVEVTYTVQR.N			
1891.9904	1892.9989	1	80	95	R.NILDFPQHVSPNKDIR.A			
1486.6796	1487.7109	16	104	115	K.KLSEFDVEMSMR.Q			Oxidation (M)
1550.794	1551.7987	-2	287	300	K.TSQTVATFLDELAR.K			
1678.889	1679.8962	0	287	301	K.TSQTVATFLDELARK.L			
1196.6877	1197.6926	-2	301	310	R.KLKPLGEQER.A			
1284.6173	1285.6224	-2	358	367	K.EYFPMQVVTR.G			Oxidation (M)
2042.9949	2043.9989	-2	401	418	R.DAASGEEIGKFYLDLYPR.E			
1085.5546	1086.5762	13	411	418	K.FYLDLYPR.E			
1345.7215	1346.7361	5	542	553	K.SRQANAGLFNLR.Q			
1102.5883	1103.5961	0	544	553	R.QANAGLFNLR.Q			
2185.0287	2186.0276	-4	560	578	K.VDQVLHTQTDVDPAEEYAR.L			
1761.8931	1762.8868	-8	643	659	R.TSILRPGGSEDASTMLK.Q			
1777.888	1778.9191	13	643	659	R.TSILRPGGSEDASTMLK.Q			Oxidation (M)

Spot 160 (20080606-C7) Ddx4								
PMF/MALDI								
Calculated	Observed	+/-ppm	start	end	Sequence	Ion Score	C.I.%	Modification
1960.8955	1961.9379	18	97	113	R.GFLNNKFEEGDSSGFWK.E			
1624.6754	1625.6774	-3	133	148	R.GGYPDGNDSEASGPFR.R			
1607.8155	1608.7781	-28	194	209	R.GAYKGLNEEVVTGSGK.N			
1087.639	1088.6438	-2	302	311	K.YSIPIVLAGR.D			
2300.2133	2301.1514	-30	324	344	K.TAAFLLPILAHMMRDGITASR.F			Oxidation (M)
1360.735	1361.7413	-1	361	371	R.ELINQIYLEAR.K			
1603.8471	1604.8655	7	381	395	R.AVVIYGGTQFGHSIR.Q			
1778.9203	1779.9283	0	461	475	R.QTLLFSATFPEEIQR.L			
869.5698	870.5476	-34	515	521	R.KLVEILR.N			
1761.9083	1762.8868	-16	522	536	R.NIGDERPMVFVETKK.K			
1777.9033	1778.9191	5	522	536	R.NIGDERPMVFVETKK.K			Oxidation (M)
1085.5465	1086.5762	21	550	559	K.ISTTSIHGDR.E			
1498.7488	1499.7693	9	550	562	K.ISTTSIHGDREQR.E			
2131.0586	2132.0588	-3	625	643	R.AISFFDTESDNHLAQPLVK.V			

Spot 162 (B6 20080606) – mixture of: ATP synthase subunit beta, mitochondrial precursor (Atp5b), ATP synthase beta subunit (Atp5b), Tubulin beta-5 chain (Tubb5), Tubulin beta-2C chain (Tubb2c), Tubulin beta-3 chain (Tubb3), Tubulin beta-2A chain (Tubb2a) and Tubulin beta-2B chain (Tubb2b)

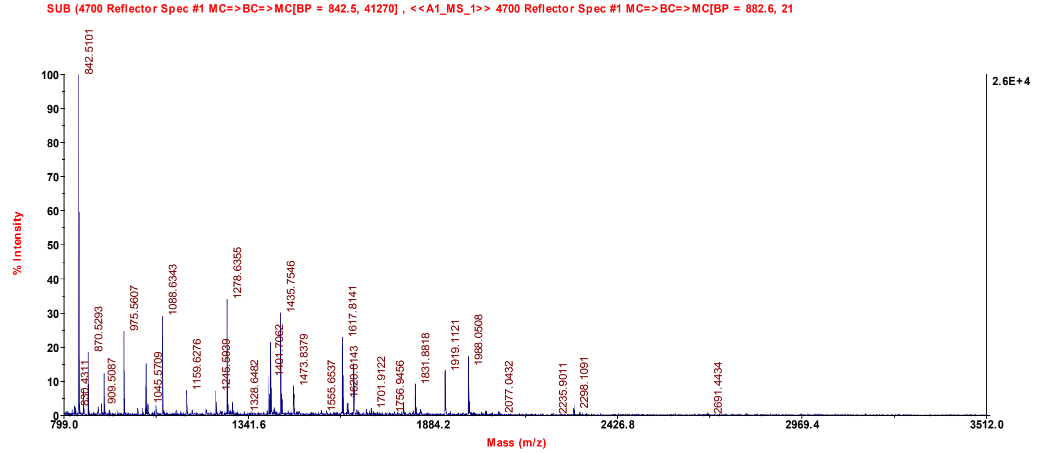

Spot 162 (20080606-B6) Atp5b (precursor)
MS+MS/MS

Calculated	Observed	+/-ppm	start	end	Sequence	Ion Score	C.I.%	Modification
975.5621	975.562	0	202	212	IGLFGGAGVGK			
975.5621	975.562	0	202	212	IGLFGGAGVGK	73	100	
1038.5942	1038.5979	4	134	143	IPVGPETLGR			
1040.6714	1040.667	-4	481	489	LVPLKETIK			
1040.6714	1040.667	-4	481	489	LVPLKETIK	18	98.277	
1088.635	1088.6351	0	189	198	VVDLLAPYAK	45	99.997	
1088.635	1088.6351	0	189	198	VVDLLAPYAK			
1217.7	1217.6989	-1	199	212	GGKIGLFGGAGVGK			
1278.6359	1278.6357	0	110	121	TIAMDGTEGLVR			Oxidation (M)
1278.6359	1278.6357	0	110	121	TIAMDGTEGLVR	16	97.06	Oxidation (M)
1401.7042	1401.7065	2	144	155	IMNVIGEPIDER			Oxidation (M)
1406.681	1406.6829	1	226	239	AHGGYSVFAGVGER			
1406.681	1406.6829	1	226	239	AHGGYSVFAGVGER	28	99.829	
1435.7539	1435.7545	0	311	324	FTQAGSEVSALLGR			
1435.7539	1435.7545	0	311	324	FTQAGSEVSALLGR	62	100	
1439.7893	1439.7858	-2	282	294	VALTGLTVAEYFR			
1473.8345	1473.8369	2	213	225	TVLIMELINNVAK			Oxidation (M)
1473.8345	1473.8369	2	213	225	TVLIMELINNVAK	13	94.797	Oxidation (M)
1617.8053	1617.8105	3	265	279	VALVYGQMNEPPGAR			Oxidation (M)
1617.8053	1617.8105	3	265	279	VALVYGQMNEPPGAR	3	39.566	Oxidation (M)
1650.9174	1650.9211	2	95	109	LVLEVAQHLGESTVR	24	99.592	
1650.9174	1650.9211	2	95	109	LVLEVAQHLGESTVR			
1796.9574	1796.9711	8	144	159	IMNVIGEPIDERGPIK			Oxidation (M)
1796.9574	1796.9711	8	144	159	IMNVIGEPIDERGPIK	4	53.625	Oxidation (M)
1831.8643	1831.8785	8	407	422	IMDPNIVGSEHYDVAR			Oxidation (M)
1831.8643	1831.8785	8	407	422	IMDPNIVGSEHYDVAR	12	93.524	Oxidation (M)
1919.0959	1919.1094	7	125	143	VLDSGAPIKIPVGPETLGR	57	100	
1919.0959	1919.1094	7	125	143	VLDSGAPIKIPVGPETLGR			
1988.0334	1988.0475	7	388	406	AIAELGIYPAVDPLDSTSR	45	99.996	
1988.0334	1988.0475	7	388	406	AIAELGIYPAVDPLDSTSR			
2039.0055	2039.0231	9	463	480	FLSQPFQVAEVFTGHMGK			Oxidation (M)
2298.074	2298.1057	14	325	345	IPSAVGYQPTLATDMGTMQER			2 Oxidation (M)

Spot 162 (20080606-B6) Atp5b (precursor)
PMF/MALDI

Calculated	Observed	+/-ppm	start	end	Sequence	Ion Score	C.I.%	Modification
1649.9101	1650.9224	3	95	109	R.LVLEVAQHLGESTVR.T			
1261.6336	1262.6232	-14	110	121	R.TIAMDGTEGLVR.G			
1277.6286	1278.6355	0	110	121	R.TIAMDGTEGLVR.G			Oxidation (M)
1037.5869	1038.5923	-2	134	143	K.IPVGPETLGR.I			
1400.6969	1401.7062	1	144	155	R.IMNVIGEPIDER.G			Oxidation (M)
1087.6277	1088.6343	-1	189	198	K.VVDLLAPYAK.G			
974.5549	975.5607	-2	202	212	K.IGLFGGAGVGK.T			
1472.8272	1473.8379	2	213	225	K.TVLIMELINNVAK.A			Oxidation (M)
1405.6739	1406.6832	1	226	239	K.AHGGYSVFAGVGER.T			
2075.9833	2077.0432	25	242	259	R.EGNDLYHEMIESGVINLK.D			Oxidation (M)
1616.7981	1617.8141	5	265	279	K.VALVYGQMNEPPGAR.A			Oxidation (M)
1438.782	1439.7874	-1	282	294	R.VALTGLTVAEYFR.D			
1434.7467	1435.7546	0	311	324	R.FTQAGSEVSALLGR.I			
2297.0668	2298.1091	15	325	345	R.IPSAVGYQPTLATDMGTMQER.I			2 Oxidation (M)
1987.0262	1988.0508	9	388	406	R.AIAELGIYPAVDPLDSTSR.I			
1830.857	1831.8818	10	407	422	R.IMDPNIVGSEHYDVAR.G			Oxidation (M)
2037.9983	2039.0341	14	463	480	R.FLSQPFQVAEVFTGHMGK.L			Oxidation (M)

Spot 162 (20080606-B6) Atp5b								
PMF/MALDI								
Calculated	Observed	+/-ppm	start	end	Sequence	Ion Score	C.I.%	Modification
1649.9101	1650.9224	3	41	55	R.LVLEVAQHLGESTVR.T			
1261.6336	1262.6232	-14	56	67	R.TIAMDGTEGLVR.G			
1277.6286	1278.6355	0	56	67	R.TIAMDGTEGLVR.G			Oxidation (M)
1918.0888	1919.1121	8	71	89	K.VLDSGAPIKIPVGPETLGR.I			
1037.5869	1038.5923	-2	80	89	K.IPVGPETLGR.I			
1400.6969	1401.7062	1	90	101	R.IMNVIGEPIDER.G			Oxidation (M)
1795.9502	1796.9758	10	90	105	R.IMNVIGEPIDERGPIK.T			Oxidation (M)
1087.6277	1088.6343	-1	135	144	K.VVDLLAPYAK.G			
1216.6928	1217.7041	3	145	158	K.GGKIGLFGGAGVGK.T			
974.5549	975.5607	-2	148	158	K.IGLFGGAGVGK.T			
1472.8272	1473.8379	2	159	171	K.TVLIMELINNVAK.A			Oxidation (M)
1405.6739	1406.6832	1	172	185	K.AHGGYSVFAGVGER.T			
2075.9833	2077.0432	25	188	205	R.EGNDLYHEMIESGVINLK.D			Oxidation (M)
1616.7981	1617.8141	5	211	225	K.VALVYGQMNEPPGAR.A			Oxidation (M)
1438.782	1439.7874	-1	228	240	R.VALTGLTVAEYFR.D			
1434.7467	1435.7546	0	257	270	R.FTQAGSEVSALLGR.I			
2297.0668	2298.1091	15	271	291	R.IPSAVGYQPTLATDMGTMQER.I			2 Oxidation (M)
1987.0262	1988.0508	9	334	352	R.AIAELGIYPAVDPLDSTSR.I			
1830.857	1831.8818	10	353	368	R.IMDPNIVGSEHYDVAR.G			Oxidation (M)
2690.3684	2691.4434	25	379	402	K.SLQDIIAILGMDELSEEDKLTVSR.A			Oxidation (M)
2037.9983	2039.0341	14	409	426	R.FLSQPFQVAEVFTGHMGK.L			Oxidation (M)
1039.6641	1040.665	-6	427	435	K.LVPLKETIK.G			

Spot 162 (20080606-B6) Tubb5								
PMF/MALDI								
Calculated	Observed	+/-ppm	start	end	Sequence	Ion Score	C.I.%	Modification
1300.6299	1301.6589	17	47	58	R.ISVYYNEATGGK.Y			
1630.8236	1631.8336	2	63	77	R.AILVDLEPGTMDSVR.S			Oxidation (M)
1129.588	1130.6001	4	242	251	R.FPGQLNADLR.K			
1158.6219	1159.6276	-1	253	262	K.LAVNMVPFPR.L			Oxidation (M)
1619.8283	1620.8143	-13	263	276	R.LHFFMPGFAPLTSR.G			
1658.8879	1659.8839	-7	283	297	R.ALTVPELTQQVFDAK.N			
1038.5862	1039.5927	-1	310	318	R.YLTVAAVFR.G			
1695.8257	1696.8177	-9	337	350	K.NSSYFVEWIPNNVK.T			
1244.586	1245.5939	1	381	390	R.ISEQFTAMFR.R			Oxidation (M)

Spot 162 (20080606-B6) Tubb2c								
PMF/MALDI								
Calculated	Observed	+/-ppm	start	end	Sequence	Ion Score	C.I.%	Modification
1327.6408	1328.6482	0	47	58	R.INVYYNEATGGK.Y			
1616.808	1617.8141	-1	63	77	R.AVLVDLEPGTMDSVR.S			Oxidation (M)
1129.588	1130.6001	4	242	251	R.FPGQLNADLR.K			
1158.6219	1159.6276	-1	253	262	K.LAVNMVPFPR.L			Oxidation (M)
1619.8283	1620.8143	-13	263	276	R.LHFFMPGFAPLTSR.G			
1706.8549	1707.8745	7	283	297	R.ALTVPELTQQMFDAK.N			Oxidation (M)
1038.5862	1039.5927	-1	310	318	R.YLTVAAVFR.G			
1695.8257	1696.8177	-9	337	350	K.NSSYFVEWIPNNVK.T			
1244.586	1245.5939	1	381	390	R.ISEQFTAMFR.R			Oxidation (M)

Spot 162 (20080606-B6) Tubb3								
PMF/MALDI								
Calculated	Observed	+/-ppm	start	end	Sequence	Ion Score	C.I.%	Modification
1630.8236	1631.8336	2	63	77	R.AILVDLEPGTMDSVR.S			Oxidation (M)
1129.588	1130.6001	4	242	251	R.FPGQLNADLR.K			
1158.6219	1159.6276	-1	253	262	K.LAVNMVPFPR.L			Oxidation (M)
1619.8283	1620.8143	-13	263	276	R.LHFFMPGFAPLTAR.G			Oxidation (M)
1706.8549	1707.8745	7	283	297	R.ALTVPELTQQMFDAK.N			Oxidation (M)
1068.5968	1069.6086	4	310	318	R.YLTVATVFR.G			
1405.6759	1406.6832	0	325	336	K.EVDEQMLAIQSK.N			Oxidation (M)
1695.8257	1696.8177	-9	337	350	K.NSSYFVEWIPNNVK.V			
1244.586	1245.5939	1	381	390	R.ISEQFTAMFR.R			Oxidation (M)

Spot 162 (20080606-B6) Tubb2a								
PMF/MALDI								
Calculated	Observed	+/-ppm	start	end	Sequence	Ion Score	C.I.%	Modification
1630.8236	1631.8336	2	63	77	R.AILVDLEPGTMDSVR.S			Oxidation (M)
1350.6676	1351.6962	16	163	174	R.IMNTFSVMPSPK.V			
1129.588	1130.6001	4	242	251	R.FPGQLNADLR.K			
1158.6219	1159.6276	-1	253	262	K.LAVNMVPFPR.L			Oxidation (M)
1619.8283	1620.8143	-13	263	276	R.LHFFMPGFAPLTSR.G			
1706.8549	1707.8745	7	283	297	R.ALTVPELTQQMFDSK.N			
1695.8257	1696.8177	-9	337	350	K.NSSYFVEWIPNNVK.T			
1244.586	1245.5939	1	381	390	R.ISEQFTAMFR.R			Oxidation (M)

Spot 162 (20080606-B6) Tubb2a								
PMF/MALDI								
Calculated	Observed	+/-ppm	start	end	Sequence	Ion Score	C.I.%	Modification
1630.8236	1631.8336	2	63	77	R.AILVDLEPGTMDSVR.S			Oxidation (M)
1350.6676	1351.6962	16	163	174	R.IMNTFSVMPSPK.V			
1129.588	1130.6001	4	242	251	R.FPGQLNADLR.K			
1158.6219	1159.6276	-1	253	262	K.LAVNMVPFPR.L			Oxidation (M)
1619.8283	1620.8143	-13	263	276	R.LHFFMPGFAPLTSR.G			
1706.8549	1707.8745	7	283	297	R.ALTVPELTQQMFDSK.N			
1695.8257	1696.8177	-9	337	350	K.NSSYFVEWIPNNVK.T			
1244.586	1245.5939	1	381	390	R.ISEQFTAMFR.R			Oxidation (M)

Spot 167 (D1 20070719) – predicted: Importin 5 (Ipo5)

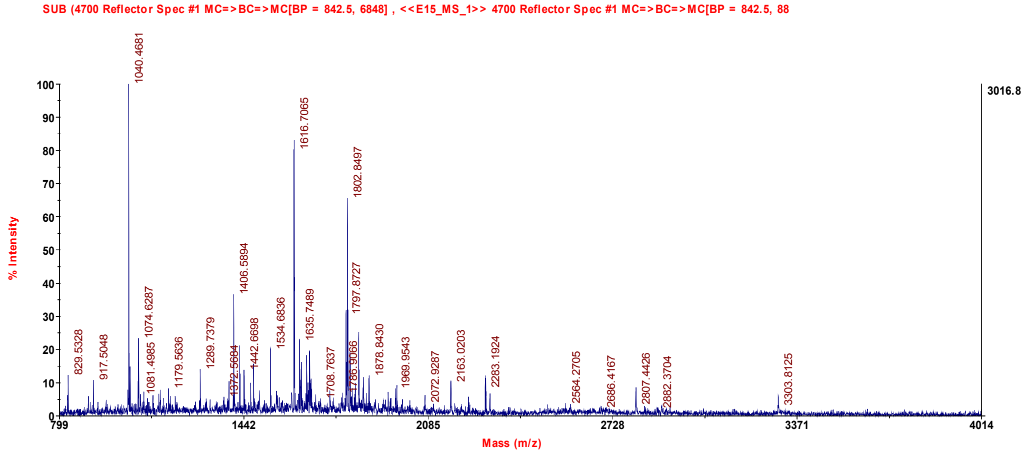

Spot 167 (20070719-D1) Ipo5 (predicted)								
PMF/MALDI								
Calculated	Observed	+/-ppm	start	end	Sequence	Ion Score	C.I.%	Modification
1533.7423	1534.6836	-43	68	80	R.KQAEETYENIPGR.S			
1405.6473	1406.5894	-46	69	80	K.QAEETYENIPGR.S			
1288.7867	1289.7379	-43	81	91	R.SKITFLLQAIR.N			
1073.6597	1074.6287	-36	83	91	K.ITFLLQAIR.N			
961.4465	962.4514	-2	92	100	R.NTTAAEEAR.Q			
916.5164	917.5048	-21	101	108	R.QMAAVLLR.R			Oxidation (M)
1072.6175	1073.5883	-34	101	109	R.QMAAVLLRR.L			Oxidation (M)
1943.9002	1944.8784	-15	132	147	K.SELLMIIQMETQSSMR.K			3 Oxidation (M)
2071.9952	2072.9287	-36	132	148	K.SELLMIIQMETQSSMRK.K			3 Oxidation (M)
1840.8591	1841.8136	-29	160	175	R.NLIDEDGNNQWPEGLK.F			
1615.7664	1616.7065	-42	176	189	K.FLFDSVSSQNMGLR.E			Oxidation (M)
1828.9835	1829.928	-34	237	253	R.ATAAFILANEHNVALFK.H			
1674.7494	1675.6906	-39	402	414	K.EHIMQMLQNADWK.Y			2 Oxidation (M)
1993.9138	1994.9261	3	402	416	K.EHIMQMLQNADWKYR.H			2 Oxidation (M)
1785.9043	1786.9066	-3	483	498	K.VIAALLQTMEDQGNQR.V			
1801.8992	1802.8497	-32	483	498	K.VIAALLQTMEDQGNQR.V			Oxidation (M)
1092.6114	1093.5751	-40	529	537	K.HLHSIMVLK.L			Oxidation (M)
1634.8055	1635.7489	-39	568	580	K.FVPYYDLFMPSLK.H			Oxidation (M)
1036.5665	1037.5446	-28	581	589	K.HIVENAVQK.E			
1926.9431	1927.8802	-36	612	627	K.EKFMQDASDVMQLLLK.T			2 Oxidation (M)
1637.8157	1638.7701	-32	614	627	K.FMQDASDVMQLLLK.T			
1669.8055	1670.7588	-32	614	627	K.FMQDASDVMQLLLK.T			2 Oxidation (M)
1810.8998	1811.8496	-32	659	673	K.EFQQYLPVVMGPLMK.T			2 Oxidation (M)
1818.8202	1819.8508	13	720	734	K.STACQMLVCYAKELK.E			Oxidation (M)
1796.9196	1797.8727	-30	732	746	K.ELKEGFVEYTEQVVK.L			
1426.698	1427.6392	-46	735	746	K.EGFVEYTEQVVK.L			
828.5143	829.5328	14	747	753	K.LMVPLLK.F			Oxidation (M)
1039.4876	1040.4681	-26	754	761	K.FYFHDGVR.V			
2162.0201	2163.0203	-3	797	816	K.AIGTEPDSDVLSEIMHSFAK.C			Oxidation (M)
1441.7313	1442.6698	-48	842	852	K.LEEHFKNQELR.Q			
1494.7718	1495.708	-48	883	895	K.VSDILHSIFSSYK.E			
1751.9094	1752.8333	-48	883	897	K.VSDILHSIFSSYKEK.V			
826.5137	827.5179	-4	1101	1107	K.RLANVVR.Q			

Spot 167 (P1036 sample O090618-01) lpo5										
**LCMS/MS (Scaffold)**										
Sample Name	Acc No.	Proteins(s) inferred	No. Peptides identified	Sequence Coverage	Position in sequence	Peptide Sequence	Precursor m/z	Charge observed	Calculated mass (neutral)	Mascot score	Mascot Expect value
P1036-O090618-01	ENSRNOP00000014859	ens|ENSRNOP00000014	32	39.40%	40 - 50	(R)SKITFLLQAIR(N)	430.6	3	1288.79	41.7	3.15E-03
		859|ENSRNOG00000010989			499 - 505	(K)LQELIQK(G)	436.27	2	870.52	41	2.04E-02
		transcript:ENSRNOT00000014859			60 - 67	(R)QMAAVLLR(R)	451.27	2	900.52	46.5	8.49E-03
		Tax_Id=10116 [Rattus norvegicus]			843 - 855	(K)VSDILHSIFSSYK(E)	499.27	3	1494.77	35.1	9.98E-02
					42 - 50	(K)ITFLLQAIR(N)	537.84	2	1073.66	84.9	2.88E-07
					42 - 50	(K)ITFLLQAIR(N)	537.84	2	1073.66	58.3	1.23E-04
					529 - 541	(K)FVPYYDLFMPSLK(H)	540.61	3	1618.81	42.9	1.82E-02
					575 - 588	(K)FMQDASDVMQLLLK(T)	546.95	3	1637.81	50.7	3.01E-03
					843 - 857	(K)VSDILHSIFSSYKEK(V)	584.98	3	1751.91	60.6	2.75E-04
					239 - 249	(K)SLVEIADTVPK(Y)	586.33	2	1170.65	39	3.71E-02
					196 - 212	(R)ATAAFILANEHNVALFK(H)	610.67	3	1828.98	43.6	1.12E-02
					196 - 212	(R)ATAAFILANEHNVALFK(H)	611	3	1829.98	32.2	1.82E-01
					963 - 979	(K)TKENVNATENcISAVGK(I)	612.63	3	1834.87	43	1.41E-02
					176 - 190	(R)MLVQcMQDQEHPSIR(T)	624.95	3	1871.83	29.9	1.51E-01
					573 - 588	(K)EKFMQDASDVMQLLLK(T)	632.66	3	1894.95	40.9	2.75E-02
					91 - 106	(K)SELLMIIQMETQSSMR(K)	632.98	3	1895.92	46.5	7.06E-03
					40 - 50	(R)SKITFLLQAIR(N)	645.4	2	1288.79	72.4	2.50E-06
					279 - 297	(R)QLALEVIVTLSETAAAMLR(K)	677.05	3	2028.13	57.6	2.39E-04
					478 - 489	(K)SLLIPYLDNLVK(H)	694.41	2	1386.81	39.7	1.35E-02
					509 - 528	(K)LVLEQVVTSIASVADTAEEK(F)	701.38	3	2101.12	70.5	2.29E-05
					509 - 528	(K)LVLEQVVTSIASVADTAEEK(F)	701.38	3	2101.12	103	1.26E-08
					28 - 39	(K)QAEETYENIPGR(S)	703.83	2	1405.65	77.1	3.88E-06
					696 - 707	(K)EGFVEYTEQVVK(L)	714.36	2	1426.7	54.1	1.07E-03
					757 - 776	(K)AIGTEPDSDVLSEIMHSFAK(C)	716.35	3	2146.03	34.5	9.53E-02
					843 - 855	(K)VSDILHSIFSSYK(E)	748.39	2	1494.77	58.5	4.56E-04
					27 - 39	(R)KQAEETYENIPGR(S)	767.88	2	1533.74	84.1	1.23E-06
					69 - 90	(R)LLSSAFDEVYPALPSDVQTAIK(S)	788.75	3	2363.23	43.2	1.32E-02
					135 - 148	(K)FLFDSVSSQNMGLR(E)	800.89	2	1599.77	88.4	3.79E-07
					529 - 541	(K)FVPYYDLFMPSLK(H)	810.41	2	1618.81	41.1	2.75E-02
					363 - 375	(K)EHIMQMLQNADWK(Y)	822.39	2	1642.76	72.1	1.17E-05
					575 - 588	(K)FmQDASDVMQLLLK(T)	827.91	2	1653.81	90.6	2.68E-07
					68 - 90	(R)RLLSSAFDEVYPALPSDVQTAIK(S)	840.78	3	2519.33	49.5	2.23E-03
					499 - 505	(K)LQELIQK(G)	871.53	1	870.52	56.6	5.61E-04
					903 - 923	(K)YAEYFLRPMLQYVcDNSPEVR(Q)	884.41	3	2650.22	39	2.45E-02
					444 - 459	(K)VIAALLQTMEDQGNQR(V)	893.96	2	1785.91	74.2	1.41E-05
					444 - 459	(K)VIAALLQTmEDQGNQR(V)	901.96	2	1801.9	67.9	5.12E-05
					2 - 26	(M)aAAAAEQQQFYLLLGNLLSPDNVVR (K)	915.15	3	2742.44	50.5	1.86E-03
					196 - 212	(R)ATAAFILANEHNVALFK(H)	915.5	2	1828.98	93.6	1.15E-07
					119 - 134	(R)NLIDEDGNNQWPEGLK(F)	921.44	2	1840.86	71.5	1.66E-05
					573 - 588	(K)EKFMQDASDVMQLLLK(T)	948.49	2	1894.95	68.9	4.35E-05
					2 - 27	(M)aAAAAEQQQFYLLLGNLLSPDNVVR K(Q)	957.85	3	2870.53	58.2	2.39E-04
					509 - 528	(K)LVLEQVVTSIASVADTAEEK(F)	1051.56	2	2101.11	112	1.66E-09
					757 - 776	(K)AIGTEPDSDVLSEIMHSFAK(C)	1074.02	2	2146.02	85.9	6.74E-07
					635 - 673	(K)TASIKPEVALLDTQDMENMSDDDG WEFVNLGDQQSFGIK(T)	1086.76	4	4343.01	82.2	6.29E-07
					69 - 90	(R)LLSSAFDEVYPALPSDVQTAIK(S)	1182.62	2	2363.23	68.7	3.71E-05
					589 - 612	(K)TQTDFNDMEDDDPQISYMISAWAR(M)	1425.11	2	2848.2	74.9	1.69E-06
					635 - 673	(K)TASIKPEVALLDTQDMENMSDDDG WEFVNLGDQQSFGIK(T)	1448.67	3	4343	78.5	1.38E-06

Spot 170 (D3 20080606) Protein phosphatase 2A activator, regulatory subunit 4 (Protein phosphatase 2A, regulatory subunit B (PR 53)) (Ppp2r4)

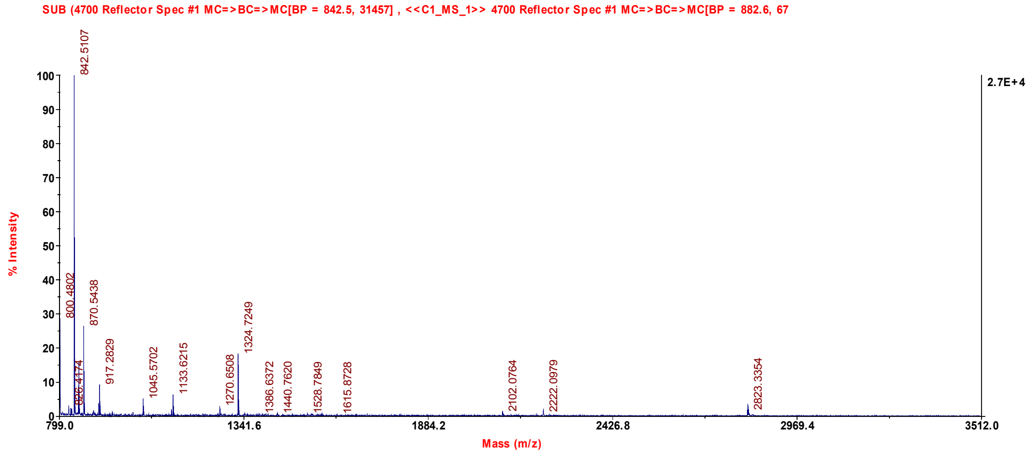

Spot 170 (20080606-D3) Ppp2r4								
PMF/MALDI								
Calculated	Observed	+/-ppm	start	end	Sequence	Ion Score	C.I.%	Modification
1269.6387	1270.6508	4	28	38	K.KEIHTVPDMGK.W			Oxidation (M)
1439.7231	1440.762	22	29	40	K.EIHTVPDMGKWK.R			
2101.0732	2102.0764	-2	42	60	R.SQAYADYIGFILTLNEGVK.G			
913.4909	914.4992	1	63	69	K.KLTFDYK.V			
1127.655	1128.6689	6	77	86	K.LVALLDTLDR.W			
854.4399	855.4559	10	100	106	R.FGNKAYR.T			
1132.6128	1133.6215	1	174	183	R.VDDQVAIVFK.V			
1342.6703	1343.6556	-16	184	193	K.VFDRYLEVMR.K			Oxidation (M)
825.4055	826.4174	6	188	193	R.YLEVMR.K			Oxidation (M)
953.5004	954.5139	6	188	194	R.YLEVMRK.L			Oxidation (M)
807.4603	808.4556	-15	195	200	K.LQKTYR.M			
1323.7187	1324.7249	-1	311	323	K.FGSLLPIHPVTSG.-			

Spot 170 (D11 20080815) - Microtubule-associated protein RP/EB family member 1 (Mapre1)

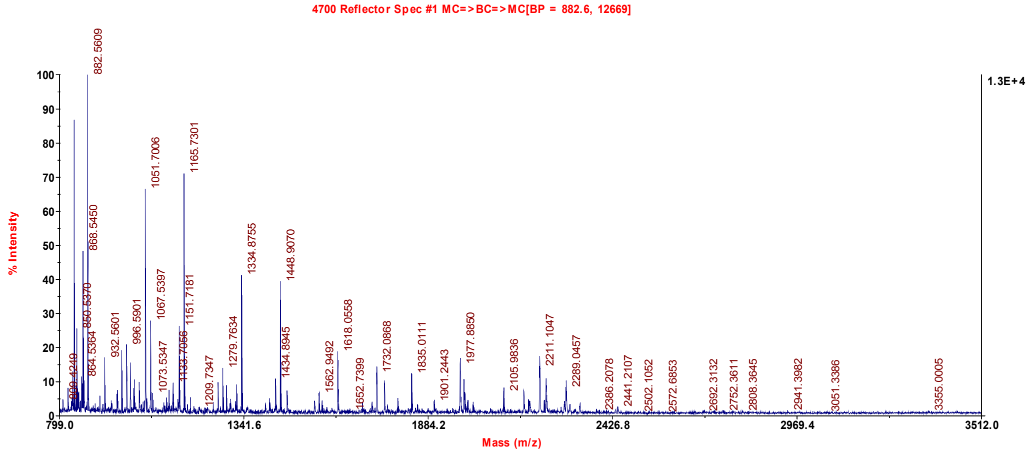

Spot 170 (20080815-D11) Ppp2r4								
MS+MS/MS								
Calculated	Observed	+/-ppm	start	end	Sequence	Ion Score	C.I.%	Modification
932.5675	932.5603	-8	77	84	ILQAGFKR			
1320.6583	1320.6429	-12	67	76	LEHEYIQNFK			
1794.9174	1794.8583	-33	63	76	FQAKLEHEYIQNFK			
1835.0245	1835.0121	-7	151	168	KPLGSGSAAPQRPIATQR			
1835.0245	1835.0121	-7	151	168	KPLGSGSAAPQRPIATQR			
1977.8977	1977.8853	-6	114	130	FFDANYDGKEYDPVAAR	10	94.941	
1977.8977	1977.8853	-6	114	130	FFDANYDGKEYDPVAAR			
1992.1124	1992.0796	-16	131	150	QGQETAVAPSLVAPALSKPK			
2163.9644	2163.9788	7	182	201	KNPGMGNGDDEAAELMQQVK			2 Oxidation (M)

Spot 174 (B13 20080606) - Septin-2 (Sept2)

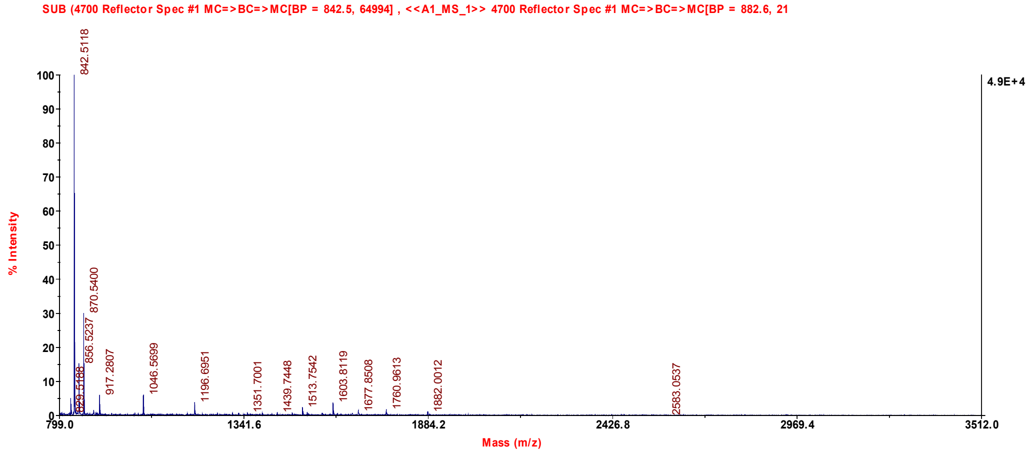

Spot 174 (20080606-B13) Sept2								
MS+MS/MS								
Calculated	Observed	+/-ppm	start	end	Sequence	Ion Score	C.I.%	Modification
858.493	858.5201	32	319	325	DQILLEK			
952.6189	952.611	-8	175	183	VNIVPVIAK			
1046.5841	1046.5691	-14	184	192	ADTLTLKER	0	0	
1046.5841	1046.5691	-14	184	192	ADTLTLKER			
1196.6997	1196.698	-1	67	77	IIPGAAEKIER			
1196.6997	1196.698	-1	67	77	IIPGAAEKIER	28	99.81	
1325.6947	1325.6918	-2	199	209	ILDEIEEHSIK			
1513.7533	1513.7513	-1	117	128	TIISYIDEQFER			
1513.7533	1513.7513	-1	117	128	TIISYIDEQFER	19	98.337	
1603.8174	1603.8112	-4	78	91	TVQIEASTVEIEER			
1603.8174	1603.8112	-4	78	91	TVQIEASTVEIEER	29	99.828	
1677.8442	1677.8506	4	97	112	LTVVDTPGYGDAINSR	26	99.704	
1677.8442	1677.8506	4	97	112	LTVVDTPGYGDAINSR			
1759.9589	1759.9509	-5	233	249	ASIPFSVVGSNQLIEAK			
1759.9589	1759.9509	-5	233	249	ASIPFSVVGSNQLIEAK			
1881.9956	1882.0024	4	51	66	STLINSLFLTDLYPER			
1881.9956	1882.0024	4	51	66	STLINSLFLTDLYPER			

Spot 174 (20080606-B13) Sept2								
PMF/MALDI								
Calculated	Observed	+/-ppm	start	end	Sequence	Ion Score	C.I.%	Modification
1880.9884	1882.0012	3	51	66	K.STLINSLFLTDLYPER.I			
1195.6924	1196.6951	-4	67	77	R.IIPGAAEKIER.T			
1602.8101	1603.8119	-3	78	91	R.TVQIEASTVEIEER.G			
1886.9949	1887.9987	-2	78	94	R.TVQIEASTVEIEERGVK.L			
1676.837	1677.8508	4	97	112	R.LTVVDTPGYGDAINSR.D			
1512.746	1513.7541	1	117	128	K.TIISYIDEQFER.Y			
1045.5768	1046.5699	-13	184	192	K.ADTLTLKER.E			
1324.6874	1325.6821	-9	199	209	R.ILDEIEEHSIK.I			
1758.9516	1759.9485	-6	233	249	K.ASIPFSVVGSNQLIEAK.G			
909.4048	910.4039	-9	332	338	R.MQEMIAR.M			2 Oxidation (M)

Spot 177 (C11 20080606) Protein phosphatase 2A activator, regulatory subunit 4 (Protein phosphatase 2A, regulatory subunit B (PR 53)) (Ppp2r4)

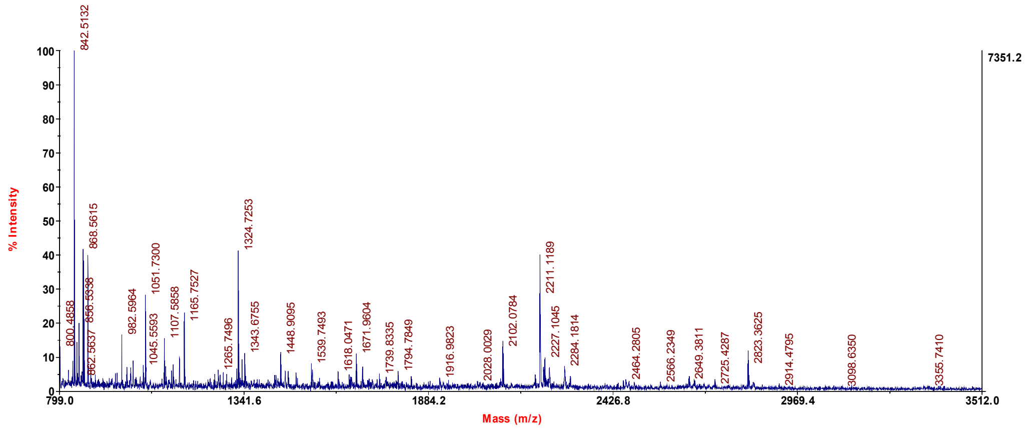

Spot 177 (20080606-C11) Ppp2r4								
PMF/MALDI								
Calculated	Observed	+/-ppm	start	end	Sequence	Ion Score	C.I.%	Modification
2238.1056	2239.1313	8	7	27	R.QPPPDSSEETPSATPTFIIPK.K			
2101.0732	2102.0784	-1	42	60	R.SQAYADYIGFILTLNEGVK.G			
2286.1896	2287.2009	2	42	62	R.SQAYADYIGFILTLNEGVKGK.K			
1669.8927	1670.8972	-2	63	76	K.KLTFDYKVSEAIEK.L			
1541.7977	1542.7842	-13	64	76	K.LTFDYKVSEAIEK.L			
1127.655	1128.6617	0	77	86	K.LVALLDTLDR.W			
2648.381	2649.3811	-3	77	99	K.LVALLDTLDRWIDETPPVDQPSR.F			
1538.7365	1539.7493	4	87	99	R.WIDETPPVDQPSR.F			
1670.972	1671.9604	-11	169	183	K.IGVLRVDDQVAIVFK.V			
1132.6128	1133.6317	10	174	183	R.VDDQVAIVFK.V			
1649.8777	1650.8804	-3	174	187	R.VDDQVAIVFKVFDR.Y			
2441.2777	2442.1736	-46	174	193	R.VDDQVAIVFKVFDRYLEVMR.K			
1342.6703	1343.6755	-2	184	193	K.VFDRYLEVMR.K			Oxidation (M)
1470.7653	1471.7703	-2	184	194	K.VFDRYLEVMRK.L			Oxidation (M)
1014.5651	1015.5699	-2	303	310	K.FPVIQHFK.F			
1323.7187	1324.7253	0	311	323	K.FGSLLPIHPVTSG.-			

Spot 180 (D21 20070719) – Protein disulfide-isomerase (P4hb, Pdia1)

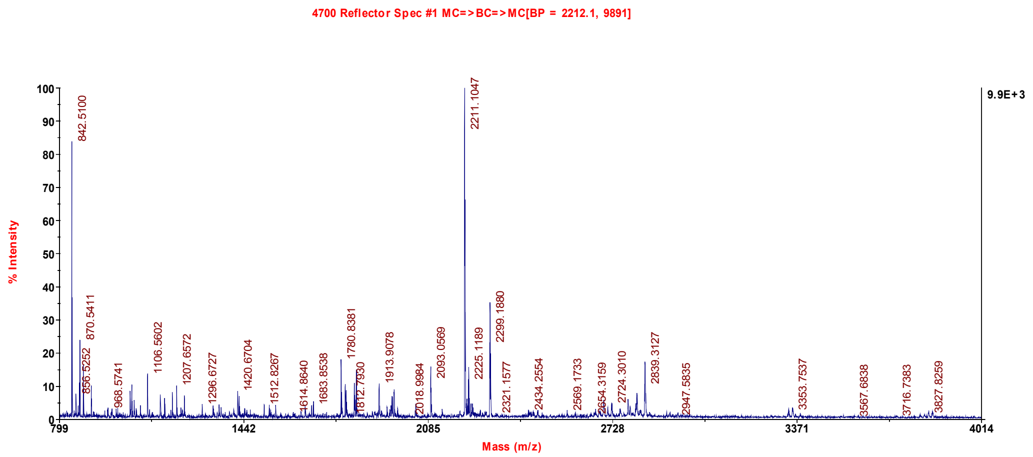

Spot 180 (20070719-D21) P4hb, Pdia1								
**MS+MS/MS**								
Calculated	Observed	+/-ppm	start	end	Sequence	Ion Score	C.I.%	Modification
910.4417	910.442	0	447	454	FFPASADR			
1424.7783	1424.7697	-6	198	209	YQLDKDGVVLFK			
1552.8733	1552.8718	-1	198	210	YQLDKDGVVLFKK			
1655.766	1655.7738	5	211	224	FDEGRNNFEGEITK			
1780.8348	1780.834	0	84	99	VDATEESDLAQQYGVR			
1780.8348	1780.834	0	84	99	VDATEESDLAQQYGVR			
1833.913	1833.9158	2	288	302	ILFIFIDSDHTDNQR	6	77.98	
1833.913	1833.9158	2	288	302	ILFIFIDSDHTDNQR			
1912.9034	1912.9113	4	211	226	FDEGRNNFEGEITKEK			
1957.9402	1957.9503	5	447	463	FFPASADRTVIDYNGER			
1965.0439	1965.045	1	233	249	HNQLPLVIEFTEQTAPK			
1965.0439	1965.045	1	233	249	HNQLPLVIEFTEQTAPK			
2093.0508	2093.0537	1	81	99	LAKVDATEESDLAQQYGVR			
2093.0508	2093.0537	1	81	99	LAKVDATEESDLAQQYGVR			
2219.1013	2219.1096	4	427	446	MDSTANEVEAVKVHSFPTLK			Oxidation (M)
2709.4609	2709.3352	-46	233	256	HNQLPLVIEFTEQTAPKIFGGEIK			
2752.415	2752.3369	-28	81	105	LAKVDATEESDLAQQYGVRGYPTIK			

Spot 180 (20070719-D21) P4hb, Pdia1								
**PMF/MALDI**								
Calculated	Observed	+/-ppm	start	end	Sequence	Ion Score	C.I.%	Modification
1001.5505	1002.5637	6	72	80	K.LKAEGSEIR.L			
2092.0436	2093.0566	3	81	99	R.LAKVDATEESDLAQQYGVR.G			
1779.8275	1780.838	2	84	99	K.VDATEESDLAQQYGVR.G			
2439.1918	2440.156	-18	84	105	K.VDATEESDLAQQYGVRGYPTIK.F			
3715.7952	3716.738	-17	135	172	R.TGPAATTLSDTAAAESLVDSSEVTVIGFFKDAGSDSAK.Q			
2683.3381	2684.3135	-12	173	197	K.QFLLAAEAVDDIPFGITSNSDVFSK.Y			
1423.7711	1424.7665	-8	198	209	K.YQLDKDGVVLFK.K			
1654.7587	1655.774	5	211	224	K.FDEGRNNFEGEITK.E			
1307.6357	1308.6401	-2	216	226	R.NNFEGEITKEK.L			
1964.0367	1965.0511	4	233	249	K.HNQLPLVIEFTEQTAPK.I			
2708.4537	2709.3306	-48	233	256	K.HNQLPLVIEFTEQTAPKIFGGEIK.T			
1080.6695	1081.6775	1	257	265	K.THILLFLPK.S			
2018.0221	2018.9983	-15	286	302	K.GKILFIFIDSDHTDNQR.I			
1832.9057	1833.9185	3	288	302	K.ILFIFIDSDHTDNQR.I			
965.5586	966.5642	-2	303	310	R.ILEFFGLK.K			
1221.6162	1222.6284	4	319	328	R.LITLEEEMTK.Y			Oxidation (M)
1408.6722	1409.6929	10	329	340	K.YKPESDELTAEK.I			
2433.2362	2434.2554	5	353	372	K.IKPHLMSQELPEDWDKQPVK.V			Oxidation (M)
1226.5455	1227.5577	4	378	387	K.NFEEVAFDEK.K			
1354.6405	1355.6542	5	378	388	K.NFEEVAFDEKK.N			
1728.9046	1729.9224	6	412	426	K.LGETYKDHENIVIAK.M			
2218.094	2219.1128	5	427	446	K.MDSTANEVEAVKVHSFPTLK.F			Oxidation (M)
927.5178	928.5508	28	439	446	K.VHSFPTLK.F			
909.4345	910.442	0	447	454	K.FFPASADR.T			
1956.933	1957.9537	7	447	463	K.FFPASADRTVIDYNGER.T			
1065.5091	1066.528	11	455	463	R.TVIDYNGER.T			

Spot 187 (D17 20070719) – Alpha-centractin (Actr1a)

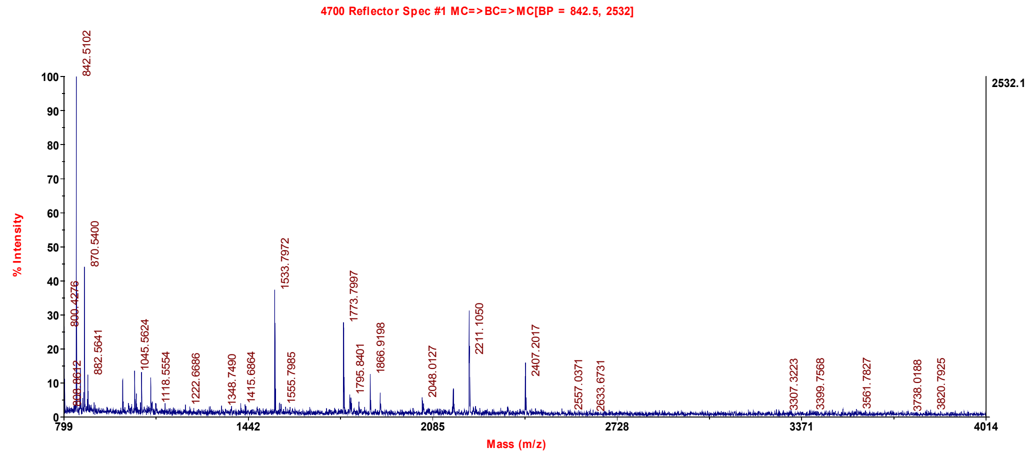

Spot 187 (20070719-D17) Actr1a								
PMF/MALDI								
Calculated	Observed	+/-ppm	start	end	Sequence	Ion Score	C.I.%	Modification
1002.5134	1003.5367	16	23	32	K.AGFAGDQIPK.Y			
1532.7909	1533.7972	-1	47	61	R.VMAGALEGDIFIGPK.A			Oxidation (M)
2155.0732	2156.1138	15	47	66	R.VMAGALEGDIFIGPKAEEHR.G			Oxidation (M)
1088.5325	1089.5431	3	73	81	R.YPMEHGIVK.D			Oxidation (M)
2050.8877	2051.8726	-11	73	88	R.YPMEHGIVKDWNDMER.I			2 Oxidation (M)
1100.5938	1101.5892	-11	183	192	R.IDIAGRDVSR.F			
1900.8843	1901.8918	0	200	215	R.KEGYDFHSSSEFEIVK.A			
1772.7893	1773.7997	2	201	215	K.EGYDFHSSSEFEIVK.A			
1865.9159	1866.9198	-2	239	255	K.AQYYLPDGSTIEIGPSR.F			
1068.604	1069.6195	8	328	336	K.IRISAPQER.L			
799.4188	800.4276	2	330	336	R.ISAPQER.L			

Spot 188 (E1 20070719) – Proliferating cell nuclear antigen (Pcna)

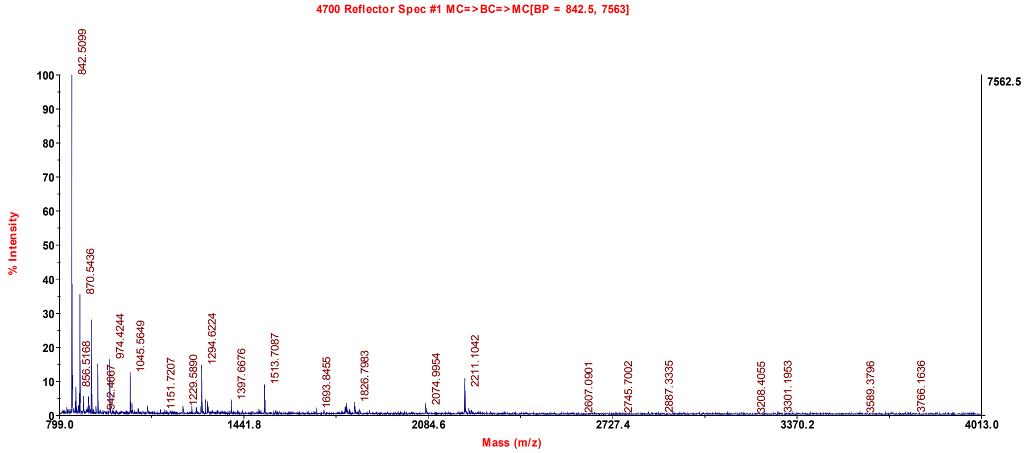

Spot 188 (20070719-E1) Pcna								
MS+MS/MS								
Calculated	Observed	+/-ppm	start	end	Sequence	Ion Score	C.I.%	Modification
900.4607	900.4645	4	241	248	IADMGHLK			Oxidation (M)
910.4087	910.4077	-1	139	146	MPSGEFAR			Oxidation (M)
910.4087	910.4077	-1	139	146	MPSGEFAR			Oxidation (M)
932.4875	932.4891	2	211	217	YLNFFTK			
932.4875	932.4891	2	211	217	YLNFFTK	27	99.792	
974.4213	974.4222	1	54	61	SEGFDTYR	5	63.494	
974.4213	974.4222	1	54	61	SEGFDTYR			
1293.6433	1293.6249	-14	169	181	FSASGELGNGNIK			
1397.6763	1397.6659	-7	65	77	NLAMGVNLTSMSK			2 Oxidation (M)
1513.7057	1513.7073	1	249	261	YYLAPKIEDEEGS			
1513.7057	1513.7073	1	249	261	YYLAPKIEDEEGS	0	0	
1719.9496	1719.9065	-25	65	80	NLAMGVNLTSMSKILK			
2074.9927	2074.991	-1	92	110	AEDNADTLALVFEAPNQEK	14	95.988	
2074.9927	2074.991	-1	92	110	AEDNADTLALVFEAPNQEK			

Spot 188 (20070719-E1) Pcna								
PMF/MALDI								
Calculated	Observed	+/-ppm	start	end	Sequence	Ion Score	C.I.%	Modification
973.4141	974.4244	3	54	61	R.SEGFDTYR.C			
1796.8219	1797.8129	-9	62	77	R.CDRNLAMGVNLTSMSK.I			
1828.8117	1829.8138	-3	62	77	R.CDRNLAMGVNLTSMSK.I			2 Oxidation (M)
1364.6792	1365.6415	-33	65	77	R.NLAMGVNLTSMSK.I			
1396.669	1397.6676	-6	65	77	R.NLAMGVNLTSMSK.I			2 Oxidation (M)
1718.9423	1719.9095	-23	65	80	R.NLAMGVNLTSMSKILK.C			
2073.9854	2074.9954	1	92	110	R.AEDNADTLALVFEAPNQEK.V			
893.4065	894.4213	8	139	146	K.MPSGEFAR.I			
909.4014	910.4088	0	139	146	K.MPSGEFAR.I			Oxidation (M)
1292.6361	1293.6304	-10	169	181	K.FSASGELGNGNIK.L			
931.4803	932.4893	2	211	217	R.YLNFFTK.A			
899.4535	900.4644	4	241	248	K.IADMGHLK.Y			Oxidation (M)
1512.6984	1513.7087	2	249	261	K.YYLAPKIEDEEGS.-			

Spot 192 (C10 20080606) - Mu-chrystallin homolog (Crym)

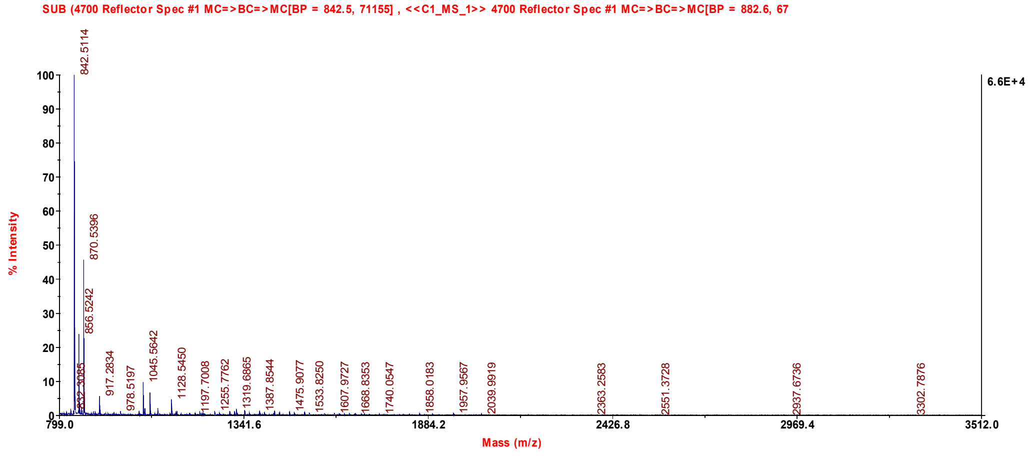

Spot 192 (20080606-C10) Crym								
**MS+MS/MS**								
Calculated	Observed	+/-ppm	start	end	Sequence	Ion Score	C.I.%	Modification
1050.5579	1050.5555	-2	242	250	QAVLYVDSR	9	87.734	
1050.5579	1050.5555	-2	242	250	QAVLYVDSR			
1065.5323	1065.5336	1	176	185	FASSVQGDVR			
1065.5323	1065.5336	1	176	185	FASSVQGDVR	9	87.096	
1088.6422	1088.6429	1	118	128	RTAAVSAIATK			
1088.6422	1088.6429	1	118	128	RTAAVSAIATK			
1128.5466	1128.5472	1	37	47	GPDGGVMQPVR			Oxidation (M)
1128.5466	1128.5472	1	37	47	GPDGGVMQPVR	11	92.062	Oxidation (M)
1141.5524	1141.5577	5	304	313	LVYDSWSSGK			
1319.6875	1319.6869	0	291	303	SLGMAVEDLVAAK			Oxidation (M)
1319.6875	1319.6869	0	291	303	SLGMAVEDLVAAK			Oxidation (M)
1636.7925	1636.793	0	171	185	ENAEKFASSVQGDVR			
1668.834	1668.8275	-4	4	18	APAFLSADEVQDHLR			
1858.032	1858.0294	-1	19	36	SSSLLIPPLEAALANFSK			
1896.0146	1895.9785	-19	286	303	TTVFKSLGMAVEDLVAAK			Oxidation (M)
1957.9575	1957.9514	-3	57	75	GFLGVMPAYSAAEDALTTK			Oxidation (M)
2039.9954	2039.9926	-1	234	250	ELDDELMKQAVLYVDSR			Oxidation (M)

Spot 192 (20080606-C10) Crym								
**PMF/MALDI**								
Calculated	Observed	+/-ppm	start	end	Sequence	Ion Score	C.I.%	Modification
1667.8267	1668.8353	1	4	18	R.APAFLSADEVQDHLR.S			
1857.0247	1858.0183	-7	19	36	R.SSSLLIPPLEAALANFSK.G			
1127.5394	1128.545	-1	37	47	K.GPDGGVMQPVR.T			Oxidation (M)
1956.9503	1957.9567	0	57	75	R.GFLGVMPAYSAAEDALTTK.L			Oxidation (M)
1087.6349	1088.6405	-2	118	128	K.RTAAVSAIATK.F			
931.5338	932.5331	-9	119	128	R.TAAVSAIATK.F			
1635.7852	1636.8014	5	171	185	R.ENAEKFASSVQGDVR.V			
1064.5251	1065.5341	2	176	185	K.FASSVQGDVR.V			
2038.9881	2039.9919	-2	234	250	R.ELDDELMKQAVLYVDSR.E			Oxidation (M)
1049.5506	1050.5554	-2	242	250	K.QAVLYVDSR.E			
1895.0074	1895.9846	-16	286	303	K.TTVFKSLGMAVEDLVAAK.L			Oxidation (M)
1318.6802	1319.6865	-1	291	303	K.SLGMAVEDLVAAK.L			Oxidation (M)
1140.5451	1141.5593	6	304	313	K.LVYDSWSSGK.-			

Spot 201 (C15 20080606) – Splicing factor 3a, subunit 3 [Mus musculus] (Sf3a3)

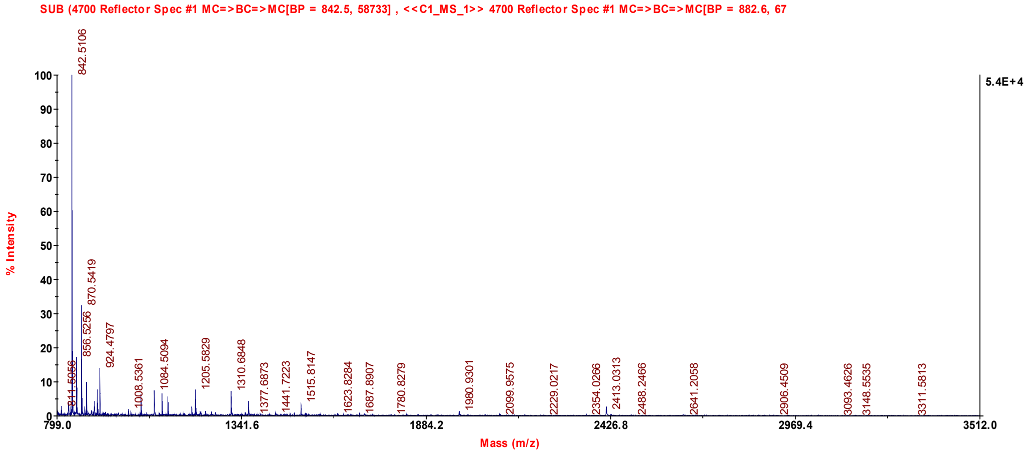

Spot 201 (20080606-C15) Sf3a3							
PMF/MALDI							
Calculated	Observed	+/-ppm	start	end	Sequence	Ion Score	C.I.%	Modification
1171.6309	1172.6293	-8	2	10	M.ETILEQQRR.Y			
860.414	861.4413	23	10	15	R.RYHEEK.E			
838.3929	839.3997	0	18	24	R.LMDVMAK.E			2 Oxidation (M)
1440.7069	1441.7223	6	31	42	K.STLRDQINSDHR.T			
1083.5019	1084.5094	0	50	58	R.YMEVSGNLR.D			Oxidation (M)
1208.5673	1209.5884	11	59	68	R.DLYDDKDGLR.K			
2098.9595	2099.9575	-4	70	87	K.EELNAISGPNEFAEFYNR.L			
956.5192	957.53	4	90	96	K.QIKEFHR.K			
2412.0353	2413.0312	-5	118	138	R.ENPSEEAQNLVEFTDEEGYGR.Y			
1979.9186	1980.9301	2	185	199	R.YLEMLLEYLQDYTDR.V			Oxidation (M)
1514.8093	1515.8147	-1	200	212	R.VKPLQDQNELFGK.I			
879.4338	880.4443	4	213	219	K.IQTDFEK.K			
1007.5287	1008.5361	0	213	220	K.IQTDFEKK.W			
884.5695	885.5754	-1	265	273	K.SALLALGLK.C			
1494.793	1495.7976	-2	290	303	K.GKSLESLDTSLFAK.N			
1309.6765	1310.6848	1	292	303	K.SLESLDTSLFAK.N			
1123.5734	1124.5802	0	339	347	R.QLTHENVQR.K			
1251.6684	1252.6731	-2	339	348	R.QLTHENVQRK.Q			
802.445	803.4521	0	418	424	R.GPKAFQR.H			
844.398	845.4119	8	425	430	R.HFAEWR.H			
2353.0346	2354.0266	-6	471	490	R.WQPDTEEEYEDSSGNVVNKK.T			
923.4712	924.4797	1	491	497	K.TYEDLKR.Q			

Spot 201 (D7 20070719) – Heterogeneous nuclear ribonucleoprotein K (Hnrnpk)

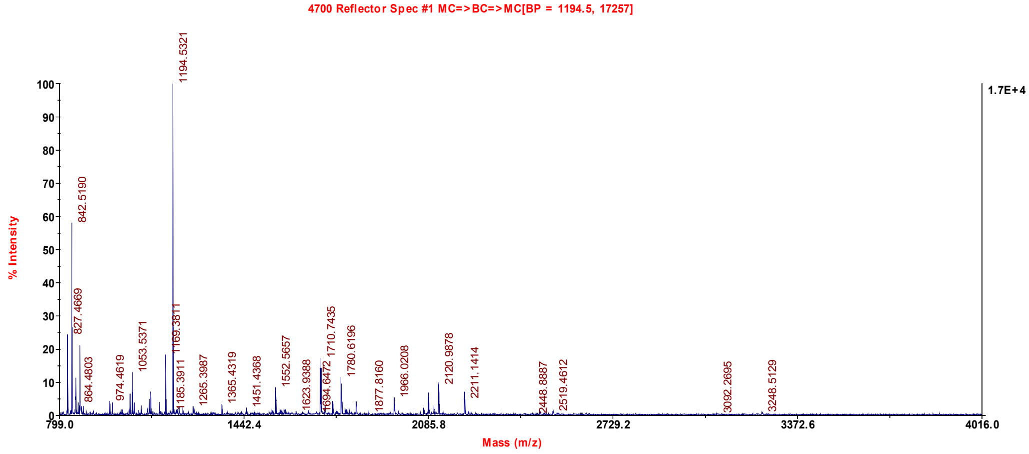

Spot 201 (20070719-D7) Hnrnpk

MS+MS/MS

Calculated	Observed	+/-ppm	start	end	Sequence	Ion Score	C.I.%	Modification
876.4249	876.4186	-7	457	463	QYSGKFF			
1013.4356	1013.4648	29	317	325	GGDLMAYDR			Oxidation (M)
1014.3832	1014.4172	34	279	286	DYDDMSPR			Oxidation (M)
1053.6415	1053.652	10	192	201	VVLIGGKPDR			
1053.6415	1053.652	10	192	201	VVLIGGKPDR			
1105.5208	1105.5552	31	259	268	GGFDRMPPGR			Oxidation (M)
1169.5367	1169.5696	28	317	326	GGDLMAYDRR	11	96.09	Oxidation (M)
1169.5367	1169.5696	28	317	326	GGDLMAYDRR			
1194.6993	1194.735	30	306	316	NLPLPPPPPPR			
1194.6993	1194.735	30	306	316	NLPLPPPPPPR	49	99.999	
1229.6683	1229.6609	-6	248	258	GRPVGFPMRGR			
2121.9553	2121.8572	-46	269	286	GGRPMPPSRRDYDDMSPR			2 Oxidation (M)
2121.9553	2121.8572	-46	269	286	GGRPMPPSRRDYDDMSPR			2 Oxidation (M)

Spot 213 (D6 20070719) – Succinate dehydrogenase [ubiquinone] flavoprotein subunit, mitochondrial precursor (Sdha)

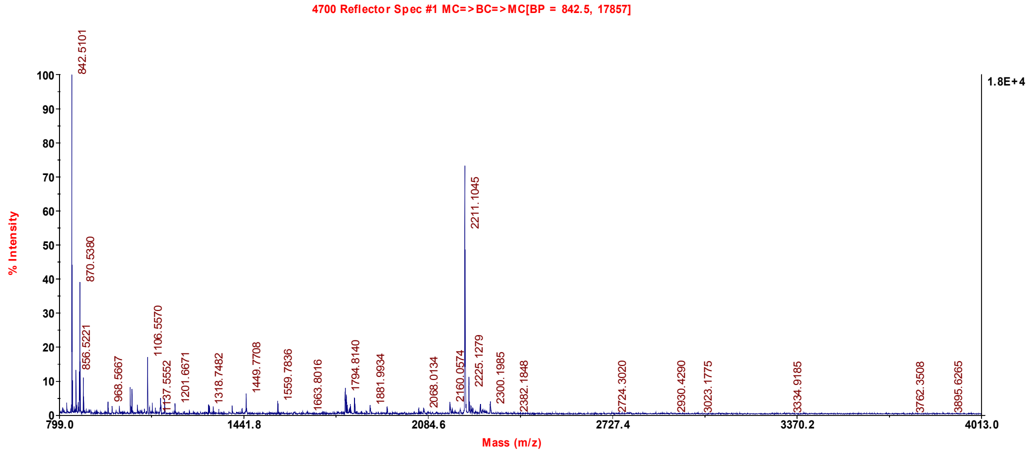

Spot 213 (20070719-D6) Sdha								
PMF/MALDI								
Calculated	Observed	+/-ppm	start	end	Sequence	Ion Score	C.I.%	Modification
1094.5185	1095.5428	16	113	120	R.WHFYDTVK.G			
1136.5574	1137.5552	-8	155	163	R.TEDGRIYQR.A			
2277.9929	2279.0176	8	181	199	R.CCCVADRTGHSLLHTLYGR.S			
1353.7153	1354.7296	5	188	199	R.TGHSLLHTLYGR.S			
1320.715	1321.7206	-1	241	253	R.AKNTIIATGGYGR.T			
1121.5829	1122.583	-6	243	253	K.NTIIATGGYGR.T			
2250.9522	2252.063	46	254	274	R.TYFSCTSAHTSTGDGTAMVTR.A			
1645.8093	1646.8055	-7	478	492	K.ANAGEESVMNLDKLR.F			
1661.8042	1662.8225	7	478	492	K.ANAGEESVMNLDKLR.F			Oxidation (M)
1751.8447	1752.8514	0	505	519	R.LSMQKSMQSHAAVFR.V			2 Oxidation (M)
1148.5397	1149.5619	13	510	519	K.SMQSHAAVFR.V			Oxidation (M)
1399.746	1400.7614	6	531	542	K.VSQLYGDLQHLK.T			
2067.0273	2068.0134	-10	591	607	K.VRIDEYDYSKPIEGQQK.K			
1811.8577	1812.8661	1	593	607	R.IDEYDYSKPIEGQQK.K			
1069.5457	1070.5603	7	608	615	K.KPFAEHWR.K			
1197.6407	1198.6654	15	608	616	K.KPFAEHWRK.H			
1317.7292	1318.7482	9	629	639	K.VTLDYRPVIDK.T			

Spot 218 (D1 20080606) – mixture of: 14-3-3 zeta/delta (Ywhaz) and 14-3-3 protein gamma (Ywhag)

SUB (4700 Reflector Spec #1 MC=>BC=>MC[BP = 1548.7, 10727] , <<C1_MS_1>> 4700 Reflector Spec #1 MC=>BC=>MC[BP = 882.6, 6

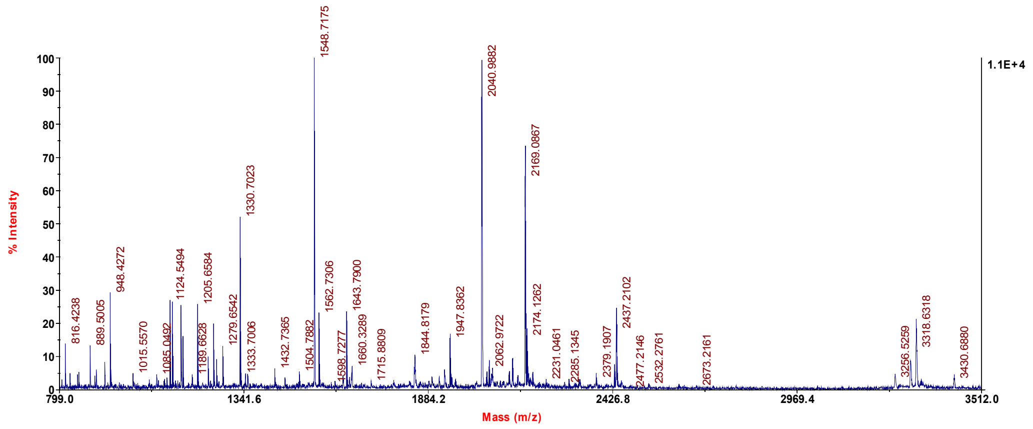

Spot 218 (20080606-D1) Ywhaz								
PMF/MALDI								
Calculated	Observed	+/-ppm	start	end	Sequence	Ion Score	C.I.%	Modification
1014.5458	1015.557	4	10	18	K.AKLAEQAER.Y			
815.4137	816.4238	4	12	18	K.LAEQAER.Y			
1933.7856	1934.8495	29	12	27	K.LAEQAERYDDMAACMK.S			2 Oxidation (M)
1547.7063	1548.7175	3	28	41	K.SVTEQGAELSNEER.N			
2436.2132	2437.2102	-4	28	49	K.SVTEQGAELSNEERNLLSVAYK.N			
906.5174	907.5287	4	42	49	R.NLLSVAYK.N			
888.4916	889.5005	2	61	68	R.VVSSIEQK.T			
1503.778	1504.7882	2	61	74	R.VVSSIEQKTEGAEK.K			
1631.873	1632.8796	0	61	75	R.VVSSIEQKTEGAEKK.Q			
1329.6928	1330.7023	2	104	115	K.FLIPNASQPESK.V			
1597.7963	1598.7277	-47	116	127	K.VFYLKMKGDYYR.Y			Oxidation (M)
931.4222	932.4315	2	121	127	K.MKGDYYR.Y			
947.4171	948.4272	3	121	127	K.MKGDYYR.Y			Oxidation (M)
1278.6456	1279.6542	1	128	139	R.YLAEVAAGDDKK.G			
3300.615	3301.5815	-12	128	157	R.YLAEVAAGDDKKGIVDQSQQAYQEAFEISK.K			
2296.1699	2297.1646	-5	139	158	K.KGIVDQSQQAYQEAFEISKK.E			
2039.98	2040.9882	0	140	157	K.GIVDQSQQAYQEAFEISK.K			
2168.0749	2169.0867	2	140	158	K.GIVDQSQQAYQEAFEISKK.E			
1235.6445	1236.6598	7	158	167	K.KEMQPTHPIR.L			
1251.6394	1252.6476	1	158	167	K.KEMQPTHPIR.L			Oxidation (M)
1107.5495	1108.5632	6	159	167	K.EMQPTHPIR.L			
1123.5444	1124.5494	-2	159	167	K.EMQPTHPIR.L			Oxidation (M)
2316.2041	2317.189	-10	168	187	R.LGLALNFSVFYYEILNSPEK.A			
2130.9844	2131.9812	-5	194	212	K.TAFDEAIAELDTLSEESYK.D			
3317.6224	3318.6318	1	194	222	K.TAFDEAIAELDTLSEESYKDSTLIMQLLR.D			Oxidation (M)
1188.6536	1189.6628	2	213	222	K.DSTLIMQLLR.D			
1204.6485	1205.6584	2	213	222	K.DSTLIMQLLR.D			Oxidation (M)

Spot 218 (20080606-D1) Ywhag								
PMF/MALDI								
Calculated	Observed	+/-ppm	start	end	Sequence	Ion Score	C.I.%	Modification
815.4137	816.4238	4	13	19	R.LAEQAER.Y			
1843.808	1844.8179	1	13	28	R.LAEQAERYDDMAAAMK.N			2 Oxidation (M)
1642.7798	1643.79	2	29	42	K.NVTELNEPLSNEER.N			
2531.2867	2532.2761	-7	29	50	K.NVTELNEPLSNEERNLLSVAYK.N			
906.5174	907.5287	4	43	50	R.NLLSVAYK.N			
902.5073	903.5107	-4	62	69	R.VISSIEQK.T			
1597.7963	1598.7277	-47	121	132	K.VFYLKMKGDYYR.Y			Oxidation (M)
931.4222	932.4315	2	126	132	K.MKGDYYR.Y			
947.4171	948.4272	3	126	132	K.MKGDYYR.Y			Oxidation (M)
1235.651	1236.6598	1	133	143	R.YLAEVATGEKR.A			
2376.128	2377.187	22	153	172	K.AYSEAHEISKEHMQPTHPIR.L			Oxidation (M)
1260.6033	1261.6112	0	163	172	K.EHMQPTHPIR.L			Oxidation (M)
2129.9641	2130.9656	-3	199	217	K.TAFDDAIAELDTLNEDSYK.D			
3300.6071	3301.5815	-10	199	227	K.TAFDDAIAELDTLNEDSYKDSTLIMQLLR.D			
3316.602	3317.5806	-9	199	227	K.TAFDDAIAELDTLNEDSYKDSTLIMQLLR.D			Oxidation (M)
1188.6536	1189.6628	2	218	227	K.DSTLIMQLLR.D			
1204.6485	1205.6584	2	218	227	K.DSTLIMQLLR.D			Oxidation (M)

Spot 240 (C23 20080606) - 14-3-3 protein epsilon (Ywhae)

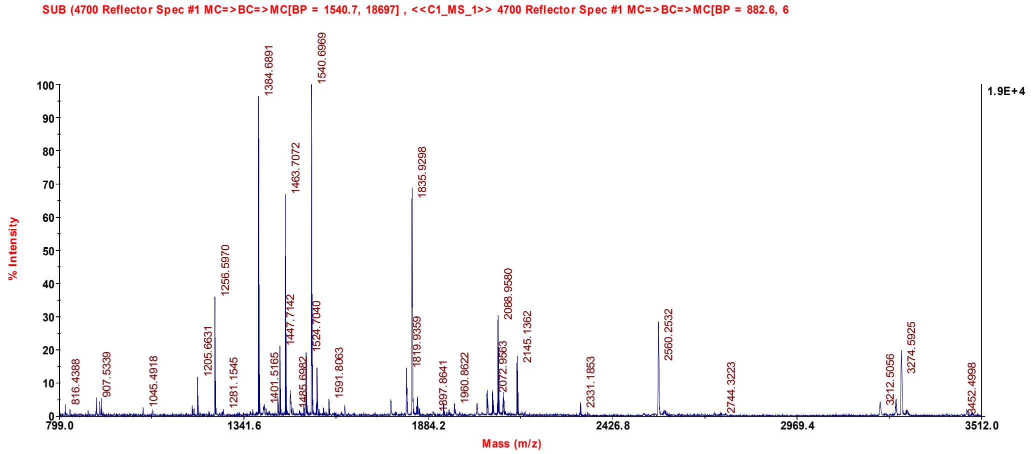

Spot 240 (20080606-C23) Ywhae								
**PMF/MALDI**								
Calculated	Observed	+/-ppm	start	end	Sequence	Ion Score	C.I.%	Modification
815.4137	816.4388	22	13	19	K.LAEQAER.Y			
1927.8655	1928.8673	-3	13	28	K.LAEQAERYDEMVESMK.K			
1943.8604	1944.8766	5	13	28	K.LAEQAERYDEMVESMK.K			Oxidation (M)
1959.8553	1960.8622	0	13	28	K.LAEQAERYDEMVESMK.K			2 Oxidation (M)
1574.7974	1575.8132	5	29	42	K.KVAGMDVELTVEER.N			
1590.7923	1591.8063	4	29	42	K.KVAGMDVELTVEER.N			Oxidation (M)
1446.7024	1447.7142	3	30	42	K.VAGMDVELTVEER.N			
1462.6974	1463.7072	2	30	42	K.VAGMDVELTVEER.N			Oxidation (M)
906.5174	907.5339	10	43	50	R.NLLSVAYK.N			
1516.8725	1517.8828	2	43	56	R.NLLSVAYKNVIGAR.R			
916.5229	917.5478	19	62	69	R.IISSIEQK.E			
882.4381	883.4578	14	81	86	K.MIREYR.Q			Oxidation (M)
1440.6918	1441.7024	2	84	94	R.EYRQMVETELK.L			Oxidation (M)
1937.0047	1938.0088	-2	107	123	K.HLIPAANTGESKVFYYK.M			
905.4178	906.4367	13	124	130	K.MKGDYHR.Y			
921.4127	922.4326	14	124	130	K.MKGDYHR.Y			Oxidation (M)
1255.5833	1256.597	5	131	141	R.YLAEFATGNDR.K			
1383.6783	1384.6891	3	131	142	R.YLAEFATGNDRK.E			
1193.5928	1194.6115	10	143	153	K.EAAENSLVAYK.A			
1818.9298	1819.9359	-1	154	170	K.AASDIAMTELPPTHPIR.L			
1834.9247	1835.9298	-1	154	170	K.AASDIAMTELPPTHPIR.L			Oxidation (M)
2330.1947	2331.1853	-7	171	190	R.LGLALNFSVFYYEILNSPDR.A			
2086.9582	2087.9653	0	197	215	K.AAFDDAIAELDTLSEESYK.D			
3257.6013	3258.5818	-8	197	225	K.AAFDDAIAELDTLSEESYKDSTLIMQLLR.D			
3273.5962	3274.5925	-3	197	225	K.AAFDDAIAELDTLSEESYKDSTLIMQLLR.D			Oxidation (M)
1188.6536	1189.665	3	216	225	K.DSTLIMQLLR.D			
1204.6485	1205.6631	6	216	225	K.DSTLIMQLLR.D			Oxidation (M)
3466.459	3467.4968	9	226	255	R.DNLTLWTSDMQGDGEEQNKEALQDVEDENQ.-			Oxidation (M)

Spot 240 (P1014-05) Ywhae											
**LCMS/MS (Scaffold)**											
Sample Name	Acc No.	Proteins(s) inferred	No. Peptides identified	Sequence Coverage	Position in sequence	Peptide Sequence	Precursor m/z	Charge observed	Calculated mass (neutral)	Mascot score	Mascot Expect value
P1014-05	P62260	sp|P62260|14-3-3 protein epsilon Tax_Id=10116 [Rattus norvegicus]	9	32.50%	87 - 94	(R)QMVETELK(L)	489.16	2	976.30	54.9	1.55E-002
					143 - 153	(K)EAAENSLVAYK(A)	597.68	2	1193.35	60.6	3.62E-003
					131 - 141	(R)YLAEFATGNDR(K)	629.08	2	1256.15	51.9	2.62E-002
					245 - 255	(K)EALQDVEDENQ(-)	645.08	2	1288.15	72.4	2.39E-004
					13 - 28	(K)LAEQAERYDEmVESMK(K)	648.86	3	1943.56	44.6	1.35E-001
					13 - 29	(K)LAEQAERYDEMVESMKK(V)	686.82	3	2057.43	58.0	6.29E-003
					30 - 42	(K)VAGmDVELTVEER(N)	732.20	2	1462.39	70.6	3.30E-004
					1 - 12	(-)mDDREDLVYQAK(L)	762.70	2	1523.39	52.0	2.34E-002
					13 - 28	(K)LAEQAERYDEMVESMK(K)	964.76	2	1927.50	56.8	6.59E-003

Spot 240 [P1014-05 (75062 Rodent)] Ywhae								
LCMS/MS								
Calculated	Observed	+/-ppm	start	end	Sequence	Ion Score	C.I.%	Modification
1523.6926	762.6622		1	12	-.MDDREDLVYQAK.L	39		Acetyl (Protein N-term)
1523.6926	762.7012		1	12	-.MDDREDLVYQAK.L	52		Acetyl (Protein N-term)
1523.6926	762.7227		1	12	-.MDDREDLVYQAK.L	49		Acetyl (Protein N-term)
1523.6926	762.7391		1	12	-.MDDREDLVYQAK.L	47		Acetyl (Protein N-term)
1523.6926	763.1816		1	12	-.MDDREDLVYQAK.L	37		Acetyl (Protein N-term)
1539.6875	770.1812		1	12	-.MDDREDLVYQAK.L	19		Acetyl (Protein N-term); Oxidation (M)
1539.6875	513.9822		1	12	-.MDDREDLVYQAK.L	29		Acetyl (Protein N-term); Oxidation (M)
1539.6875	770.6812		1	12	-.MDDREDLVYQAK.L	26		Acetyl (Protein N-term); Oxidation (M)
1539.6875	771.1996		1	12	-.MDDREDLVYQAK.L	25		Acetyl (Protein N-term); Oxidation (M)
1927.8655	964.7587		13	28	K.LAEQAERYDEMVESMK.K	57		
1927.8655	964.8182		13	28	K.LAEQAERYDEMVESMK.K	49		
1927.8655	643.8385		13	28	K.LAEQAERYDEMVESMK.K	35		
1943.8604	648.8605		13	28	K.LAEQAERYDEMVESMK.K	45		Oxidation (M)
1959.8553	654.1422		13	28	K.LAEQAERYDEMVESMK.K	8		2 Oxidation (M)
2055.9605	514.9177		13	29	K.LAEQAERYDEMVESMKK.V	35		
2055.9605	686.5392		13	29	K.LAEQAERYDEMVESMKK.V	47		
2055.9605	686.8182		13	29	K.LAEQAERYDEMVESMKK.V	58		
2071.9554	691.8405		13	29	K.LAEQAERYDEMVESMKK.V	32		Oxidation (M)
1462.6974	732.1996		30	42	K.VAGMDVELTVEER.N	71		Oxidation (M)
906.5174	454.1381		43	50	R.NLLSVAYK.N	35		
1516.8725	506.8195		43	56	R.NLLSVAYKNVIGAR.R	16		
2144.1324	715.6212		62	80	R.IISSIEQKEENKGGEDKLK.M	54		
976.4899	489.1581		87	94	R.QMVETELK.L	55		
992.4848	497.1021		87	94	R.QMVETELK.L	36		Oxidation (M)
992.4848	497.6016		87	94	R.QMVETELK.L	25		Oxidation (M)
718.369	719.22		119	123	K.VFYYK.M	17		
1255.5833	629.0812		131	141	R.YLAEFATGNDR.K	52		
2559.2605	854.2411		131	153	R.YLAEFATGNDRKEAAENSLVAYK.A	31		
1193.5928	597.6797		143	153	K.EAAENSLVAYK.A	61		
1193.5928	598.1376		143	153	K.EAAENSLVAYK.A	36		
1193.5928	598.1581		143	153	K.EAAENSLVAYK.A	30		
1834.9247	612.5002		154	170	K.AASDIAMTELPPTHPIR.L	19		Oxidation (M)
1288.5419	645.0402		245	255	K.EALQDVEDENQ.-	13		
1288.5419	645.0812		245	255	K.EALQDVEDENQ.-	72		

Spot 242 (C5 20080606) – mixture of: F-actin-capping protein subunit alpha-2 (Capza2) and F-actin capping protein alpha-1 subunit (Capza1)

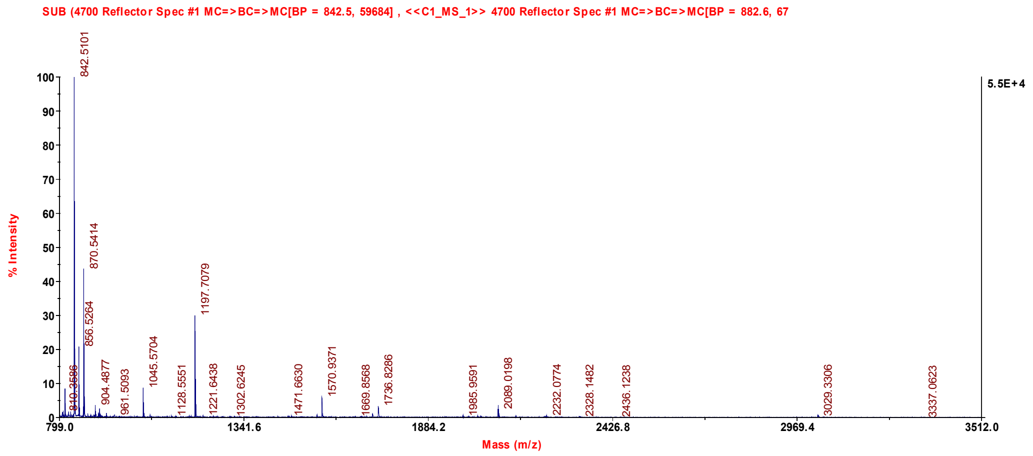

Spot 242 (20080606-C5) Capza2								
MS+MS/MS								
Calculated	Observed	+/-ppm	start	end	Sequence	Ion Score	C.I.%	Modification
815.4522	815.4528	1	98	103	FDHLRK	15	97.303	
815.4522	815.4528	1	98	103	FDHLRK			
869.5315	869.5419	12	260	266	RQLPVTR			
904.4886	904.488	-1	267	273	TKIDWNK	12	94.884	
904.4886	904.488	-1	267	273	TKIDWNK			
906.4349	906.4688	37	279	286	IGKEMQNA			Oxidation (M)
1197.6949	1197.7072	10	38	47	LLLNNDNLLR	73	100	
1197.6949	1197.7072	10	38	47	LLLNNDNLLR			
2029.0138	2029.0148	0	194	210	IQVHYYEDGNVQLVSHK			

Spot 242 (20080606-C5) Capza2								
PMF/MALDI								
Calculated	Observed	+/-ppm	start	end	Sequence	Ion Score	C.I.%	Modification
1196.6877	1197.7079	11	38	47	R.LLLNNDNLLR.E			
814.445	815.4535	2	98	103	K.FDHLRK.E			
1984.9353	1985.9591	8	130	146	R.AYVKEHYPNGVCTVYGK.K			
890.4399	891.4515	5	173	178	R.WRSEWK.F			
2028.0065	2029.0139	0	194	210	K.IQVHYYEDGNVQLVSHK.D			
868.5243	869.5349	4	260	266	R.RQLPVTR.T			
903.4814	904.4877	-1	267	273	R.TKIDWNK.I			
905.4276	906.4713	40	279	286	K.IGKEMQNA.-			Oxidation (M)

Spot 242 (20080606-C5) Capza1								
PMF/MALDI								
Calculated	Observed	+/-ppm	start	end	Sequence	Ion Score	C.I.%	Modification
960.4876	961.5093	15	8	15	R.VSDEEKVR.I			
2088.0065	2089.0198	3	20	37	K.FITHAPPGEFNEVFNDVR.L			
1196.6877	1197.7079	11	38	47	R.LLLNNDNLLR.E			
2139.9684	2140.9941	9	48	66	R.EGAAHAFAQYNMDQFTPVK.I			Oxidation (M)
2230.0502	2231.0654	4	67	86	K.IEGYDDQVLITEHGDLGNSR.F			
814.445	815.4535	2	98	103	K.FDHLRK.E			
1555.7002	1556.7272	13	104	118	K.EASDPQPEDVDGGLK.S			
1984.9126	1985.9591	20	104	121	K.EASDPQPEDVDGGLKSWR.D			
890.4399	891.4515	5	173	178	R.WRSEWK.F			
1569.9131	1570.9371	11	179	193	K.FTITPPTAQVVGVLK.I			
2028.0065	2029.0139	0	194	210	K.IQVHYYEDGNVQLVSHK.D			
1718.8323	1719.8534	8	211	226	K.DVQDSVTVSNEVQTAK.E			
3028.3495	3029.3352	-7	231	256	K.IIESAENEYQTAISENYQTMSDTTFK.A			Oxidation (M)
868.5243	869.5349	4	260	266	R.RQLPVTR.T			
903.4814	904.4877	-1	267	273	R.TKIDWNK.I			
905.4276	906.4713	40	279	286	K.IGKEMQNA.-			Oxidation (M)

Spot 269 (D15 20080815) - Phosphatidylethanolamine-binding protein 1 (Pebp1)

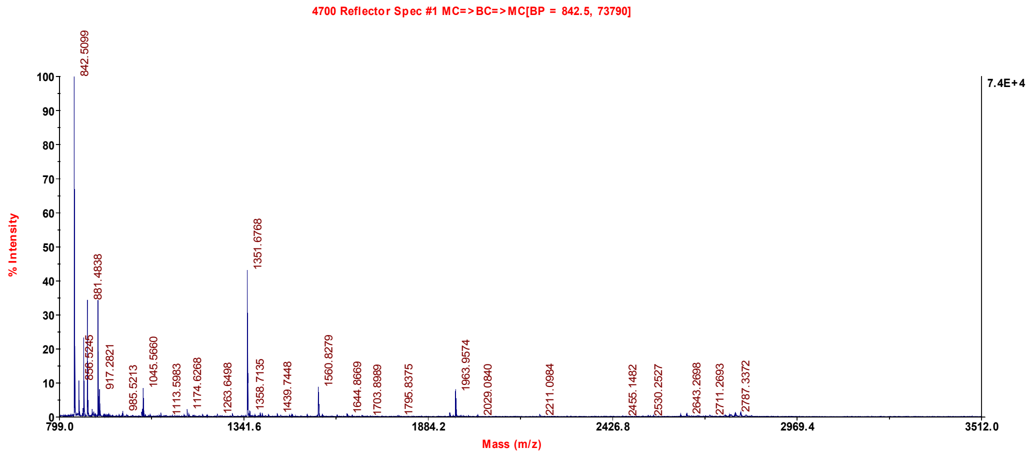

Spot 269 (20080815-D15) Pebp1								
**PMF/MALDI**								
Calculated	Observed	+/-ppm	start	end	Sequence	Ion Score	C.I.%	Modification
1350.6667	1351.6768	2	27	39	R.VDYGGVTVDELGK.V			
2513.2584	2514.2336	-13	40	62	K.VLTPTQVMNRPSSISWDGLDPGK.L			Oxidation (M)
1559.8195	1560.8279	1	63	76	K.LYTLVLTDPDAPSR.K			
1687.9145	1688.9252	2	63	77	K.LYTLVLTDPDAPSRK.D			
2028.0891	2029.084	-6	63	80	K.LYTLVLTDPDAPSRKDPK.F			
1438.718	1439.7448	14	83	93	R.EWHHFLVVNMK.G			
1962.9535	1963.9574	-2	94	113	K.GNDISSGTVLSEYVGSGPPK.D			
2642.2936	2643.2698	-12	94	119	K.GNDISSGTVLSEYVGSGPPKDTGLHR.Y			
1096.6029	1097.6111	1	147	155	R.GKFKVESFR.K			
911.4865	912.4941	0	149	155	K.FKVESFR.K			
1039.5815	1040.5903	2	149	156	K.FKVESFRK.K			
892.513	893.4798	-45	151	157	K.VESFRKK.Y			
880.4767	881.4838	0	180	187	K.LHDQLAGK.-			

Spot 269 (P1014-13) Rab6a & Itpa												
**LCMS/MS (Scaffold)**												
Sample Name	Acc No.	Proteins(s) inferred	No. Peptides identified	Sequence Coverage	Position in sequence	Peptide Sequence	Precursor m/z	Charge observed	Calculated mass (neutral)	Mascot score	Mascot Expect value
P1014-13	P35279	sp|P35279|Ras-related protein Rab-6A Tax_Id=10090 [Mus musculus]	2	12.00%	64 - 74	(R)LQLWDTAGQER(F)	658.70	2	1315.38	53.2	1.99E-002
					170 - 183	(R)VAAALPGMESTQDR(S)	723.14	2	1444.26	65.7	1.04E-003
	ENSRNOP00000028838	ens|ENSRNOP00000028838|ENSRNOG00000021233 transcript:ENSRNOT00000028838 Tax_Id=10116 [Rattus norvegicus]	1	3.54%	2 - 8	(M)aASLVGK(K)	687.26	1	686.25	41.7	2.04E-001

Spot 285 (C18 20080606) - Tissue specific transplantation antigen P35B (Tsta3)

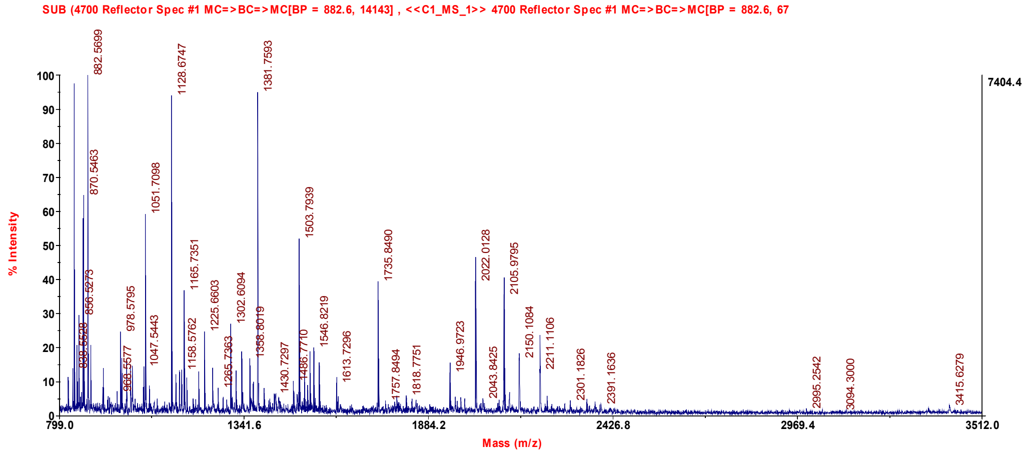

Spot 285 (20080606-C18) Tsta3								
PMF/MALDI								
Calculated	Observed	+/-ppm	start	end	Sequence	Ion Score	C.I.%	Modification
1127.6663	1128.6747	1	10	21	R.ILVTGGSGLVGR.A			
2386.2533	2387.2095	-21	22	44	R.AIQKVVADGAGLPGEEWVFVSSK.D			
1945.9786	1946.9723	-7	26	44	K.VVADGAGLPGEEWVFVSSK.D			
1734.8424	1735.849	0	45	60	K.DADLTDAAQTQALFQK.V			
1367.6986	1368.7051	-1	79	88	R.NIKYNLDFWR.K			
1012.4767	1013.4888	5	82	88	K.YNLDFWR.K			
1140.5716	1141.5787	0	82	89	K.YNLDFWRK.N			
2021.0079	2022.0128	-1	90	107	K.NVHINDNVLHSAFEVGTR.K			
2149.1028	2150.1084	-1	90	108	K.NVHINDNVLHSAFEVGTRK.V			
1046.5291	1047.5443	8	148	155	K.RMIDVQNR.A			Oxidation (M)
890.428	891.4374	2	149	155	R.MIDVQNR.A			Oxidation (M)
1502.7841	1503.7939	2	200	214	K.SSGSALTVWGTGKPR.R			
1380.7514	1381.7593	0	215	225	R.RQFIYSLDLAR.L			
1224.6503	1225.6603	2	216	225	R.QFIYSLDLAR.L			
945.58	946.5831	-4	226	232	R.LFIWVLR.E			
2390.174	2391.1636	-7	233	253	R.EYNEVEPIILSVGEEDEVSIK.E			
2348.0366	2349.0815	16	254	275	K.EAAEAVVEAMDFSGEVTFDSTK.S			Oxidation (M)

Spot 286 (D8 20080606) - Tubulin cofactor A (Tbca)

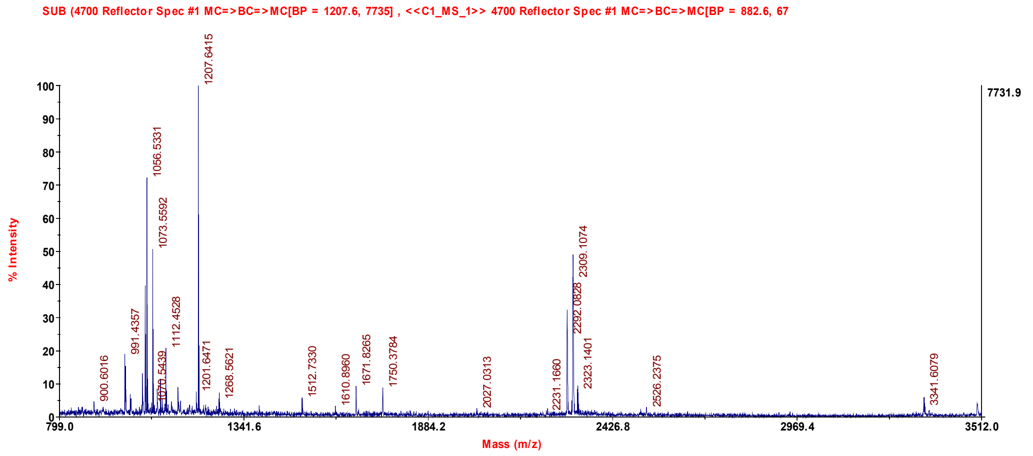

Spot 286 (20080606-D8) Tbca								
PMF/MALDI								
Calculated	Observed	+/-ppm	start	end	Sequence	Ion Score	C.I.%	Modification
1228.671	1229.6184	-49	1	10	-.MADPRVRQIK.I			Oxidation (M)
899.5916	900.6016	3	11	18	K.IKTGVVKR.L			
1269.6274	1270.5747	-47	22	31	K.EKVMYEKEAK.Q			Oxidation (M)
1511.7289	1512.733	-2	40	52	K.MKAEDGENYAIKK.Q			Oxidation (M)
2291.1393	2292.0828	-28	42	61	K.AEDGENYAIKKQAEILQESR.M			
1200.6462	1201.6471	-5	52	61	K.KQAEILQESR.M			
1072.5513	1073.5592	1	53	61	K.QAEILQESR.M			
1206.6357	1207.6415	-1	70	79	R.RLEAAYTDLR.Q			
1050.5345	1051.5414	0	71	79	R.LEAAYTDLR.Q			
2308.107	2309.1074	-3	80	98	R.QILESEKDLEEAEEYKEAR.I			
1085.6332	1086.6387	-2	99	108	R.IVLDSVKLEA.-			

Spot 290 (D19 20080815) - Tropomyosin alpha-3 chain (Tpm3)

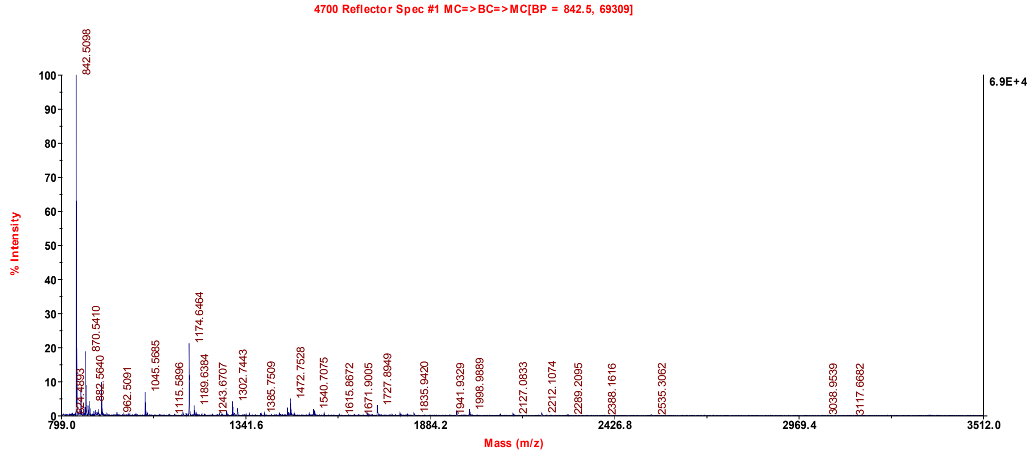

Spot 290 (20080815-D19) Tpm3								
PMF/MALDI								
Calculated	Observed	+/-ppm	start	end	Sequence	Ion Score	C.I.%	Modification
1262.6653	1263.6456	-21	1	12	-.MAGSTTIEAVKR.K			
1769.8908	1770.9226	14	13	27	R.KIQVLQQQADDAEER.A			
2126.0716	2127.0833	2	13	30	R.KIQVLQQQADDAEERAER.L			
1997.9766	1998.9889	3	14	30	K.IQVLQQQADDAEERAER.L			
1114.5731	1115.5896	8	31	39	R.LQREVEGER.R			
1270.6742	1271.6923	9	31	40	R.LQREVEGERR.A			
1542.775	1543.7888	4	41	54	R.AREQAEAEVASLNR.R			
1698.8761	1699.8883	3	41	55	R.AREQAEAEVASLNRR.I			
1315.6368	1316.6532	7	43	54	R.EQAEAEVASLNR.R			
1471.7379	1472.7528	5	43	55	R.EQAEAEVASLNRR.I			
1242.6456	1243.6707	14	56	65	R.IQLVEEELDR.A			
1726.8849	1727.8949	2	56	69	R.IQLVEEELDRAQER.L			
1442.798	1443.809	3	70	82	R.LATALQKLEEAEK.A			
961.5015	962.5091	0	90	97	R.GMKVIENR.A			Oxidation (M)
1960.0186	1961.0372	6	98	113	R.ALKDEEKMELQEIQLK.E			Oxidation (M)
1814.8911	1815.9022	2	117	131	K.HIAEEADRKYEEVAR.K			
893.4606	894.4749	8	126	132	K.YEEVARK.L			
1283.7449	1284.7581	5	132	142	R.KLVIIEGDLER.T			
1155.6499	1156.6622	4	133	142	K.LVIIEGDLER.T			
1670.8839	1671.9005	6	133	146	K.LVIIEGDLERTEER.A			
1793.8288	1794.8336	-1	156	169	R.EMDEQIRLMDQNLK.C			2 Oxidation (M)
876.4375	877.447	3	163	169	R.LMDQNLK.C			Oxidation (M)
1687.794	1688.8079	4	178	190	K.YSQKEDKYEEEIK.I			
829.5273	830.5179	-20	191	197	K.ILTDKLK.E			
1415.7984	1416.7644	-29	191	202	K.ILTDKLKEAETR.A			
845.4606	846.4998	38	196	202	K.LKEAETR.A			
1307.6106	1308.6512	26	198	208	K.EAETRAEFAER.S			
1188.6238	1189.6384	6	216	225	K.TIDDLEDKLK.C			
1466.6633	1467.6875	12	237	248	R.MLDQTLLDLNEM.-			2 Oxidation (M)

Spot 297 (D2 20080815) - Fatty acid binding protein 9, testis (Fabp9)

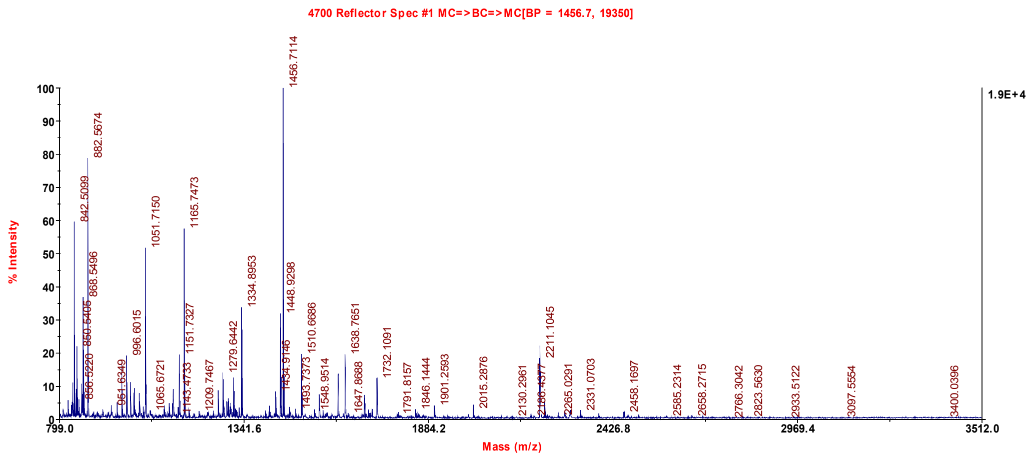

Spot 297 (20080815-D2) Fabp9								
MS+MS/MS								
Calculated	Observed	+/-ppm	start	end	Sequence	Ion Score	C.I.%	Modification
1456.7067	1456.7101	2	11	22	LVSSENFENYVR	37	99.991	
1456.7067	1456.7101	2	11	22	LVSSENFENYVR			
1510.6655	1510.6696	3	67	79	LGEEFEETTADNR			
1510.6655	1510.6696	3	67	79	LGEEFEETTADNR	10	95.552	
1638.7605	1638.7638	2	67	80	LGEEFEETTADNRK	21	99.659	
1638.7605	1638.7638	2	67	80	LGEEFEETTADNRK			
1695.8734	1695.8802	4	83	97	SLITFEGGSMIQIQR			Oxidation (M)

Spot 297 (C12 20080606) - Fatty acid binding protein 9, testis (Fabp9)

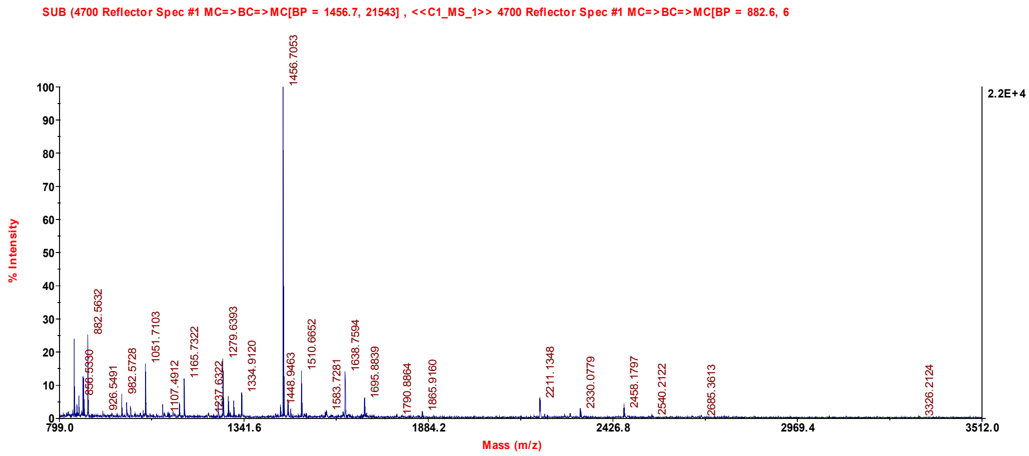

Spot 297 (20080606-C12) Fabp9								
PMF/MALDI								
Calculated	Observed	+/-ppm	start	end	Sequence	Ion Score	C.I.%	Modification
1236.6213	1237.6322	3	1	10	-.MIEPFLGTWK.L			Oxidation (M)
1455.6994	1456.7053	-1	11	22	K.LVSSENFENYVR.E			
2329.071	2330.0779	0	60	79	R.NTEISFKLGEEFEETTADNR.K			
2457.1659	2458.1797	3	60	80	R.NTEISFKLGEEFEETTADNRK.V			
1509.6583	1510.6652	0	67	79	K.LGEEFEETTADNR.K			
1637.7533	1638.7594	-1	67	80	K.LGEEFEETTADNRK.V			
1864.9166	1865.916	-4	67	82	K.LGEEFEETTADNRKVK.S			
1694.8662	1695.8839	6	83	97	K.SLITFEGGSMIQIQR.W			Oxidation (M)

Spot 302 (E5 20070719) – Microtubule-associated protein RP/EB family member 1 (Mapre1)

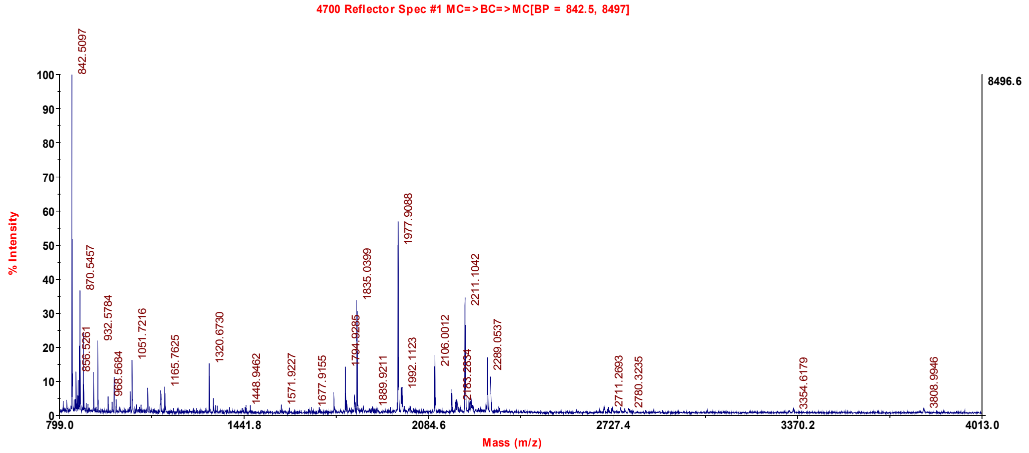

Spot 302 (20070719-E5) Mapre1								
MS+MS/MS								
Calculated	Observed	+/-ppm	start	end	Sequence	Ion Score	C.I.%	Modification
932.5675	932.5753	8	76	83	ILQAGFKR			
932.5675	932.5753	8	76	83	ILQAGFKR			
1045.5465	1045.5662	19	214	221	DFYFGKLR			
1061.505	1061.5457	38	212	219	ERDFYFGK			
1320.6583	1320.668	7	66	75	LEHEYIQNFK	8	88.061	
1320.6583	1320.668	7	66	75	LEHEYIQNFK			
1794.9174	1794.9248	4	62	75	FQAKLEHEYIQNFK			
1794.9174	1794.9248	4	62	75	FQAKLEHEYIQNFK	12	94.454	
1835.0245	1835.0361	6	150	167	KPLGSGSAAPQRPIATQR			
1835.0245	1835.0361	6	150	167	KPLGSGSAAPQRPIATQR			
1977.8977	1977.9028	3	113	129	FFDANYDGKEYDPVAAR			
1977.8977	1977.9028	3	113	129	FFDANYDGKEYDPVAAR	5	74.882	
1988.9963	1989.0118	8	204	219	LTVEDLEKERDFYFGK			
1988.9963	1989.0118	8	204	219	LTVEDLEKERDFYFGK			
1992.1124	1992.1089	-2	130	149	QGQETAVAPSLVAPALSKPK			
2105.9927	2105.998	3	112	129	KFFDANYDGKEYDPVAAR	5	73.21	
2105.9927	2105.998	3	112	129	KFFDANYDGKEYDPVAAR			
3808.1191	3808.0178	-27	130	167	QGQETAVAPSLVAPALSKPKKPLGSGSAAPQRPIATQR			

Spot 302 (20070719-E5) Mapre1								
PMF/MALDI								
Calculated	Observed	+/-ppm	start	end	Sequence	Ion Score	C.I.%	Modification
2021.0734	2022.0638	-8	61	76	K.VKFQAKLEHEYIQNFK.I			
1793.91	1794.9285	6	63	76	K.FQAKLEHEYIQNFK.I			
1319.651	1320.673	11	67	76	K.LEHEYIQNFK.I			
931.5603	932.5784	12	77	84	K.ILQAGFKR.M			
2104.9854	2106.0012	4	113	130	K.KFFDANYDGKEYDPVAAR.Q			
1976.8904	1977.9088	6	114	130	K.FFDANYDGKEYDPVAAR.Q			
1991.1051	1992.1123	0	131	150	R.QGQETAVAPSLVAPALSKPK.K			
1834.0173	1835.0399	8	151	168	K.KPLGSGSAAPQRPIATQR.T			
2018.8673	2019.9552	40	183	201	K.NPGMGNGDDEAAELMQQVK.V			Oxidation (M)
1570.893	1571.9227	14	202	214	K.VLKLTVEDLEKER.D			
1987.9891	1989.0173	11	205	220	K.LTVEDLEKERDFYFGK.L			
1060.4978	1061.5466	39	213	220	K.ERDFYFGK.L			
1044.5393	1045.5695	22	215	222	R.DFYFGKLR.N			

Spot 307 (D20 20070719) – Protein disulfide-isomerase precursor (P4hb, Pdia1)

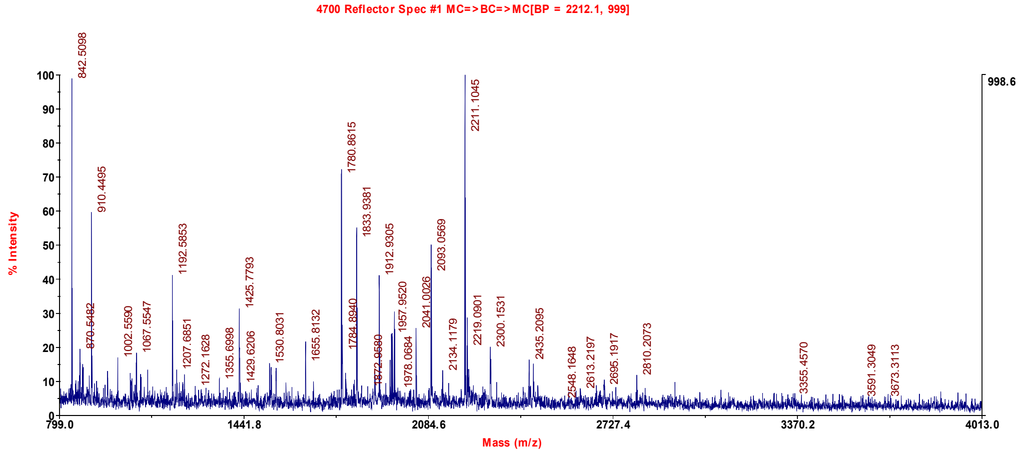

Spot 307 (20070719-D20) P4hb, Pdia1								
MS+MS/MS								
Calculated	Observed	+/-ppm	start	end	Sequence	Ion Score	C.I.%	Modification
910.4417	910.446	5	447	454	FFPASADR			
910.4417	910.446	5	447	454	FFPASADR			
966.5658	966.5798	14	303	310	ILEFFGLK			
1002.5577	1002.555	-3	72	80	LKAEGSEIR			
1066.5164	1066.5223	6	455	463	TVIDYNGER			
1081.6769	1081.6942	16	257	265	THILLFLPK			
1355.6477	1355.6957	35	378	388	NFEEVAFDEKK			
1424.7783	1424.804	18	198	209	YQLDKDGVVLFK			
1552.8733	1552.8961	15	198	210	YQLDKDGVVLFKK			
1587.8013	1587.8472	29	266	279	SVSDYDGKLSNFKK			
1655.766	1655.8142	29	211	224	FDEGRNNFEGEITK			
1780.8348	1780.8633	16	84	99	VDATEESDLAQQYGVR			
1780.8348	1780.8633	16	84	99	VDATEESDLAQQYGVR	4	40.068	
1833.913	1833.9404	15	288	302	ILFIFIDSDHTDNQR	15	95.567	
1833.913	1833.9404	15	288	302	ILFIFIDSDHTDNQR			
1912.9034	1912.9344	16	211	226	FDEGRNNFEGEITKEK			
1912.9034	1912.9344	16	211	226	FDEGRNNFEGEITKEK			
1957.9402	1957.9553	8	447	463	FFPASADRTVIDYNGER			
1965.0439	1965.0548	6	233	249	HNQLPLVIEFTEQTAPK			
2093.0508	2093.0603	5	81	99	LAKVDATEESDLAQQYGVR			
2093.0508	2093.0603	5	81	99	LAKVDATEESDLAQQYGVR	19	98.368	
2219.1013	2219.0928	-4	427	446	MDSTANEVEAVKVHSFPTLK			Oxidation (M)
2219.1013	2219.0928	-4	427	446	MDSTANEVEAVKVHSFPTLK			Oxidation (M)
2434.2434	2434.2012	-17	353	372	IKPHLMSQELPEDWDKQPVK			Oxidation (M)
2613.2852	2613.2244	-23	319	340	LITLEEEMTKYKPESDELTAEK			Oxidation (M)

Spot 307 (20070719-D20) P4hb, Pdia1								
PMF/MALDI								
Calculated	Observed	+/-ppm	start	end	Sequence	Ion Score	C.I.%	Modification
1001.5505	1002.559	1	72	80	K.LKAEGSEIR.L			
2092.0436	2093.0569	3	81	99	R.LAKVDATEESDLAQQYGVR.G			
1779.8275	1780.8615	15	84	99	K.VDATEESDLAQQYGVR.G			
1201.5979	1202.6328	23	123	132	R.EADDIVNWLK.K			
1423.7711	1424.8047	18	198	209	K.YQLDKDGVVLFK.K			
1654.7587	1655.8132	29	211	224	K.FDEGRNNFEGEITK.E			
1964.0367	1965.0522	4	233	249	K.HNQLPLVIEFTEQTAPK.I			
1080.6695	1081.698	20	257	265	K.THILLFLPK.S			
1832.9057	1833.9381	14	288	302	K.ILFIFIDSDHTDNQR.I			
965.5586	966.5825	17	303	310	R.ILEFFGLK.K			
1093.6535	1094.6735	12	303	311	R.ILEFFGLKK.E			
2612.2778	2613.2197	-25	319	340	R.LITLEEEMTKYKPESDELTAEK.I			Oxidation (M)
1408.6722	1409.7102	22	329	340	K.YKPESDELTAEK.I			
1964.9666	1966.0592	43	353	368	K.IKPHLMSQELPEDWDK.Q			
2433.2362	2434.1958	-20	353	372	K.IKPHLMSQELPEDWDKQPVK.V			Oxidation (M)
1354.6405	1355.6998	38	378	388	K.NFEEVAFDEKK.N			
1728.9046	1729.9167	3	412	426	K.LGETYKDHENIVIAK.M			
2218.094	2219.0901	-5	427	446	K.MDSTANEVEAVKVHSFPTLK.F			Oxidation (M)
927.5178	928.5173	-8	439	446	K.VHSFPTLK.F			
909.4345	910.4495	9	447	454	K.FFPASADR.T			
1956.933	1957.952	6	447	463	K.FFPASADRTVIDYNGER.T			
1065.5091	1066.5245	8	455	463	R.TVIDYNGER.T			

Spot 311 (D5 20070719) – Probable ATP-dependent RNA helicase DDX4 (Ddx4)

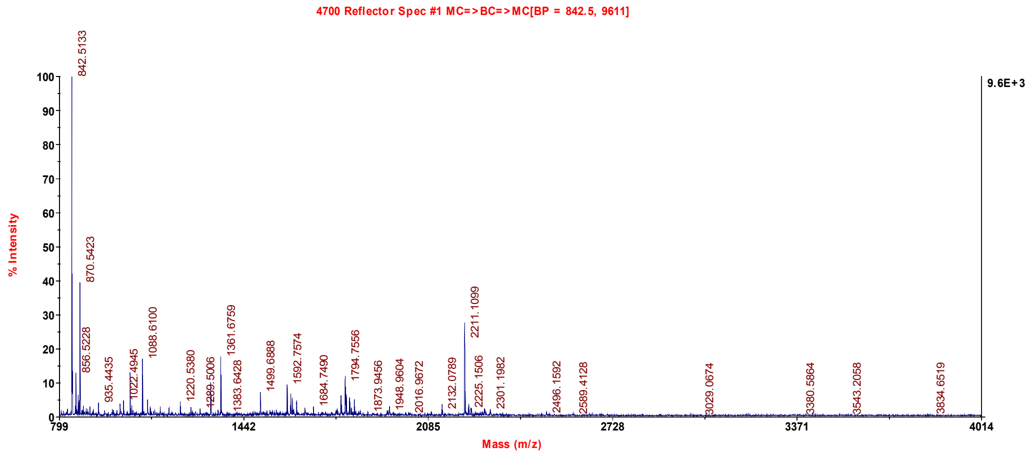

Spot 311 (20070719-D5) Ddx4								
MS+MS/MS								
Calculated	Observed	+/-ppm	start	end	Sequence	Ion Score	C.I.%	Modification
870.577	870.5404	-42	515	521	KLVEILR			
935.4581	935.4437	-15	565	572	EQALGDFR			
1088.6462	1088.615	-29	302	311	YSIPIVLAGR			
1088.6462	1088.615	-29	302	311	YSIPIVLAGR			
1220.6018	1220.5472	-45	563	572	EREQALGDFR			
1361.7423	1361.6884	-40	361	371	ELINQIYLEAR			
1361.7423	1361.6884	-40	361	371	ELINQIYLEAR	20	99.081	
1383.647	1383.6567	7	436	447	MLDMGFGPEMKK			
1499.7561	1499.7087	-32	550	562	ISTTSIHGDREQR			
1499.7561	1499.7087	-32	550	562	ISTTSIHGDREQR			
1610.7405	1610.7026	-24	173	188	KPAASDSGSGDTFQSR			
1625.6826	1625.6464	-22	133	148	GGYPDGNDSEASGPFR			
1762.9156	1762.8679	-27	522	536	NIGDERPMVFVETKK			
1779.9276	1779.8959	-18	461	475	QTLLFSATFPEEIQR			
1779.9276	1779.8959	-18	461	475	QTLLFSATFPEEIQR			
1781.7837	1781.7552	-16	133	149	GGYPDGNDSEASGPFRR			
2132.0659	2132.1204	26	625	643	AISFFDTESDNHLAQPLVK	34	99.962	
2132.0659	2132.1204	26	625	643	AISFFDTESDNHLAQPLVK			

Spot 311 (20070719-D5) Ddx4								
PMF/MALDI								
Calculated	Observed	+/-ppm	start	end	Sequence	Ion Score	C.I.%	Modification
1496.6049	1497.6653	35	39	53	R.TSASSSEMEDGPSGR.D			
1624.6754	1625.6217	-38	133	148	R.GGYPDGNDSEASGPFR.R			
1609.7332	1610.6796	-38	188	203	R.KPAASDSGSGDTFQSR.S			
1188.5986	1189.5704	-30	213	224	K.GLNEEVVTGSGK.N			
1087.639	1088.61	-33	317	326	K.YSIPIVLAGR.D			
1360.735	1361.6759	-49	376	386	R.ELINQIYLEAR.K			
1603.8471	1604.7882	-41	396	410	R.AVVIYGGTQFGHSIR.Q			
804.4415	805.4499	1	426	432	R.LMDIIGK.E			Oxidation (M)
1092.5451	1093.4994	-49	442	450	K.YLVLDEADR.M			
1105.5148	1106.504	-16	463	472	K.LISCPGMPSK.E			Oxidation (M)
1778.9203	1779.8661	-35	476	490	R.QTLLFSATFPEEIQR.L			
1591.8206	1592.7576	-44	514	527	R.DVQQSILQVGQYSK.R			
934.4508	935.4435	-16	580	587	R.EQALGDFR.C			
2131.0586	2132.0789	6	640	658	R.AISFFDTESDNHLAQPLVK.V			
1021.5193	1022.4946	-31	690	699	R.GAVFASVDTR.K			

Spot 313 (D3 20070719) – Stress-70 protein, mitochondrial precursor (Hspa9)

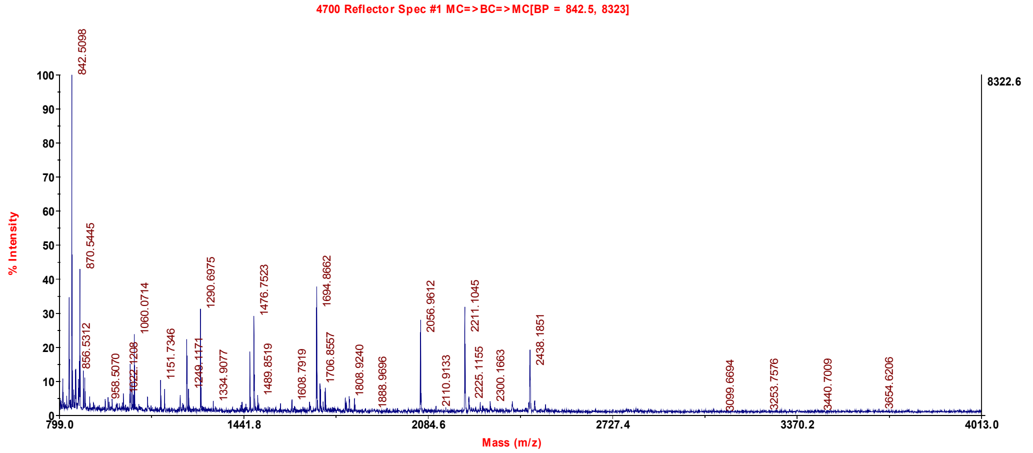

Spot 313 (20070719-D3) Hspa9								
MS+MS/MS								
Calculated	Observed	+/-ppm	start	end	Sequence	Ion Score	C.I.%	Modification
887.5131	887.5109	-2	100	107	LVGMPAKR			Oxidation (M)
904.5032	904.502	-1	618	624	MRELLAR			Oxidation (M)
958.4952	958.504	9	77	85	VLENAEGAR			
1045.5387	1045.5636	24	647	654	LFEMAYKK			Oxidation (M)
1056.6312	1056.6444	12	285	292	HIVKEFKR			
1242.6801	1242.6956	12	207	218	DAGQISGLNVLR			
1242.6801	1242.6956	12	207	218	DAGQISGLNVLR	0	0	
1290.6801	1290.6971	13	395	405	VQQTVQDLFGR			
1290.6801	1290.6971	13	395	405	VQQTVQDLFGR	7	81.02	
1462.757	1462.7739	12	378	391	SDIGEVILVGGMTR			Oxidation (M)
1462.757	1462.7739	12	378	391	SDIGEVILVGGMTR			Oxidation (M)
1476.7329	1476.752	13	86	99	TTPSVVAFTPDGER			
1476.7329	1476.752	13	86	99	TTPSVVAFTPDGER	0	3.104	
1489.8373	1489.8536	11	349	361	AQFEGIVTDLIKR			
1490.7996	1490.8464	31	613	624	EEISKMRELLAR			Oxidation (M)
1608.7687	1608.7921	15	174	187	MKETAENYLGHTAK			Oxidation (M)
1670.9183	1670.9171	-1	203	218	QATKDAGQISGLNVLR			
1694.8496	1694.8652	9	188	202	NAVITVPAYFNDSQR			
1694.8496	1694.8652	9	188	202	NAVITVPAYFNDSQR	4	58.284	
1706.8378	1706.8563	11	293	307	ETGVDLTKDNMALQR			Oxidation (M)
1709.8717	1709.8639	-5	144	159	IVRASNGDAWVEAHGK			
1724.8715	1724.8921	12	108	122	QAVTNPNNTFYATKR			
1808.9025	1808.9252	13	469	485	SQVFSTAADGQTQVEIK			
2110.9783	2110.9368	-20	596	612	MEEFKDQLPADECNKLK			Carbamidomethyl (C); Oxidation (M)
2434.2129	2434.2026	-4	542	563	EQQIVIQSSGGLSKDDIENMVK			Oxidation (M)

Spot 313 (20070719-D3) Hspa9								
PMF/MALDI								
Calculated	Observed	+/-ppm	start	end	Sequence	Ion Score	C.I.%	Modification
2421.188	2422.1882	-3	53	76	K.GAVVGIDLGTTNSCVAVMEGKQAK.V			Oxidation (M)
957.4879	958.507	12	77	85	K.VLENAEGAR.T			
1475.7256	1476.7522	13	86	99	R.TTPSVVAFTPDGER.L			
886.5058	887.5123	-1	100	107	R.LVGMPAKR.Q			Oxidation (M)
1567.7631	1568.7812	7	108	121	R.QAVTNPNNTFYATK.R			
1723.8642	1724.8933	13	108	122	R.QAVTNPNNTFYATKR.L			
971.5916	972.615	17	139	146	K.NVPFKIVR.A			
1708.8645	1709.8644	-4	144	159	K.IVRASNGDAWVEAHGK.L			
1568.8272	1569.7996	-22	160	173	K.LYSPSQIGAFVLMK.M			Oxidation (M)
1607.7613	1608.7917	14	174	187	K.MKETAENYLGHTAK.N			Oxidation (M)
1693.8424	1694.8662	10	188	202	K.NAVITVPAYFNDSQR.Q			
1669.9111	1670.9181	0	203	218	R.QATKDAGQISGLNVLR.V			
1241.6728	1242.6979	14	207	218	K.DAGQISGLNVLR.V			
1705.8305	1706.8556	10	293	307	R.ETGVDLTKDNMALQR.V			Oxidation (M)
1488.83	1489.8518	10	349	361	R.AQFEGIVTDLIKR.T			
1461.7497	1462.7737	11	378	391	K.SDIGEVILVGGMTR.M			Oxidation (M)
1289.6728	1290.6974	13	395	405	K.VQQTVQDLFGR.A			
1807.8952	1808.924	12	469	485	K.SQVFSTAADGQTQVEIK.V			
2433.2057	2434.199	-6	542	563	R.EQQIVIQSSGGLSKDDIENMVK.N			Oxidation (M)
903.496	904.5026	-1	618	624	K.MRELLAR.K			Oxidation (M)
1044.5314	1045.5656	26	647	654	K.LFEMAYKK.M			Oxidation (M)

Spot 320 (C22 20080606) - Heat shock protein 1 (chaperonin) (Hspd1)

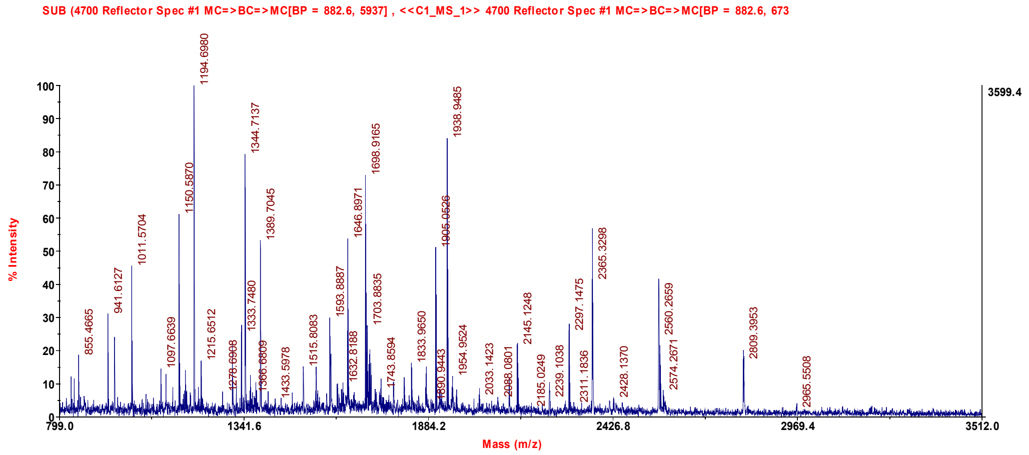

Spot 320 (20080606-C22) Hspd1								
PMF/MALDI								
Calculated	Observed	+/-ppm	start	end	Sequence	Ion Score	C.I.%	Modification
2144.1221	2145.1248	-2	38	58	R.ALMLQGVDLLADAVAVTMGPK.G			2 Oxidation (M)
1556.8311	1557.827	-7	59	72	K.GRTVIIEQSWGSPK.V			
1343.7085	1344.7137	-2	61	72	R.TVIIEQSWGSPK.V			
2559.2413	2560.2659	7	97	121	K.LVQDVANNTNEEAGDGTTTATVLAR.S			
1182.672	1183.6521	-23	131	141	K.ISKGANPVEIR.R			
854.461	855.4665	-2	134	141	K.GANPVEIR.R			
1010.5621	1011.5704	1	134	142	K.GANPVEIRR.G			
2238.0838	2239.1038	6	203	221	K.DGKTLNDELEIIEGMKFDR.G			Oxidation (M)
1921.9455	1922.9319	-11	206	221	K.TLNDELEIIEGMKFDR.G			
1937.9404	1938.9485	0	206	221	K.TLNDELEIIEGMKFDR.G			Oxidation (M)
1806.8941	1807.8937	-4	219	233	K.FDRGYISPYFINTSK.G			
2120.0691	2121.0276	-23	219	236	K.FDRGYISPYFINTSKGQK.C			
1388.6976	1389.7045	0	222	233	R.GYISPYFINTSK.G			
1729.8233	1730.8927	36	237	250	K.CEFQDAYVLLSEKK.I			
2032.1429	2033.1423	-4	250	268	K.KISSVQSIVPALEIANAHR.K			
1904.0479	1905.0526	-1	251	268	K.ISSVQSIVPALEIANAHR.K			
2364.3264	2365.3298	-2	269	290	R.KPLVIIAEDVDGEALSTLVLNR.L			
1725.9526	1726.9257	-20	293	309	K.VGLQVVAVKAPGFGDNR.K			
1854.0476	1855.0613	3	293	310	K.VGLQVVAVKAPGFGDNRK.N			
832.3828	833.3945	5	302	309	K.APGFGDNR.K			
960.4777	961.494	9	302	310	K.APGFGDNRK.N			
1645.8961	1646.8971	-4	345	359	K.VGEVIVTKDDAMLLK.G			Oxidation (M)
2452.2333	2453.2002	-16	370	389	K.RIQEITEQLDITTSEYEKEK.L			
2038.9946	2040.0173	8	371	387	R.IQEITEQLDITTSEYEK.E			
2296.1322	2297.1475	3	371	389	R.IQEITEQLDITTSEYEKEK.L			
2808.4028	2809.3953	-5	371	393	R.IQEITEQLDITTSEYEKEKLNER.L			
1631.8115	1632.8188	0	406	420	K.VGGTSDVEVNEKKDR.V			
959.5036	960.5117	1	421	429	R.VTDALNATR.A			
940.6069	941.6127	-2	463	470	K.IGIEIIKR.A			
1214.6507	1215.6512	-6	482	493	K.NAGVEGSLIVEK.I			
2563.1822	2564.2178	11	494	516	K.ILQSSSEVGYDAMLGDFVNMVEK.G			2 Oxidation (M)
1096.6604	1097.6639	-3	517	526	K.GIIDPTKVVR.T			

Spot 330 (D22 20070719) – SUMO-activating enzyme subunit 1 (Sae1)

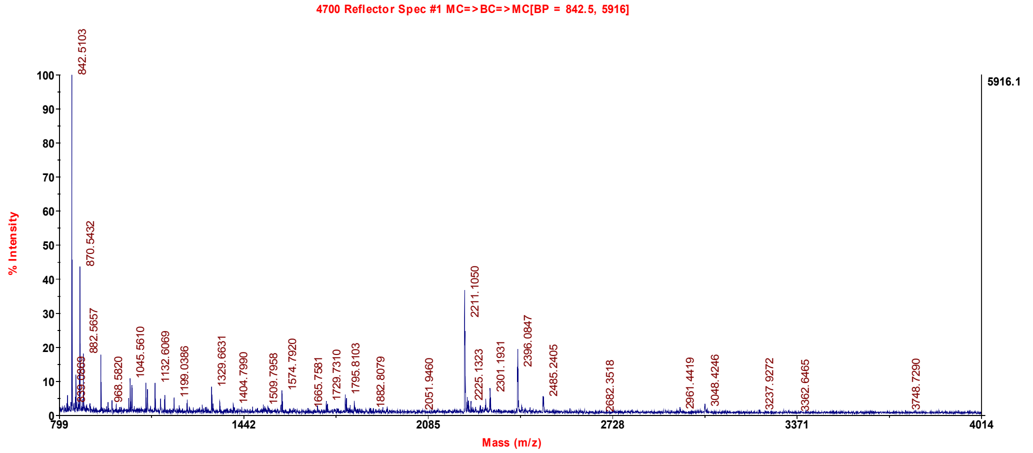

Spot 330 (20070719-D22) Sae1								
PMF/MALDI								
Calculated	Observed	+/-ppm	start	end	Sequence	Ion Score	C.I.%	Modification
2395.0775	2396.0845	0	2	24	M.VEKEEVSGGGGGISEEEAAQYDR.Q			
1099.6138	1100.6176	-3	28	36	R.LWGLEAQKR.L			
1513.8902	1514.8726	-16	42	56	R.VLIVGMKGLGAEIAK.N			Oxidation (M)
826.5276	827.5223	-15	57	64	K.NLILAGVK.G			
2484.2319	2485.2402	0	65	86	K.GLTMLDHEQVSPEDLGAQFLIR.T			Oxidation (M)
1573.7808	1574.7919	2	87	101	R.TGSVGQNRAEASLER.A			
1243.6231	1244.6166	-11	102	112	R.AQNLNPMVDVK.V			Oxidation (M)
1356.6634	1357.6595	-8	187	199	K.VSQGVEDGPDAKR.A			
2264.092	2265.0693	-13	257	276	K.GRDPTSDSYSEDAELLLQIR.N			
2050.9695	2051.9458	-15	259	276	R.DPTSDSYSEDAELLLQIR.N			
2219.0747	2220.0918	4	277	296	R.NDVFDSLGVSPDLLPDDFVR.Y			
2283.0531	2284.1475	38	320	338	K.ALSQRDPPHNNFFFFDGMK.G			Oxidation (M)
1727.7403	1728.7565	5	325	338	R.DPPHNNFFFFDGMK.G			Oxidation (M)

Spot 338 (C21 20080606) – NADH-ubiquinone oxidoreductase 75 kDa subunit, mitochondrial (Ndufs1)

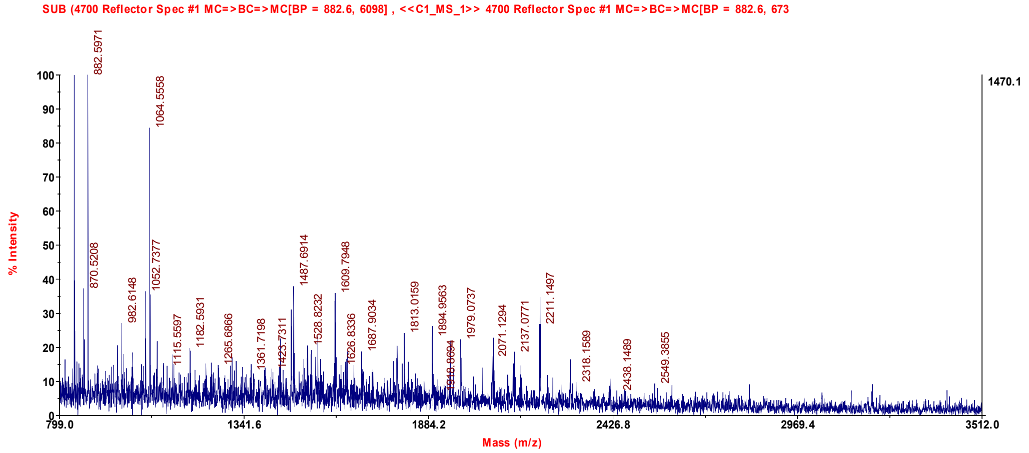

Spot 338 (20080606-C21) Ndufs1								
PMF/MALDI								
Calculated	Observed	+/-ppm	start	end	Sequence	Ion Score	C.I.%	Modification
1692.7865	1693.7677	-15	56	68	K.VGMQIPRFCYHER.L			
1607.7791	1608.7827	-2	185	200	R.FASEIAGVDDLGTTGR.G			
2136.0733	2137.0771	-2	247	266	R.KTESIDVMDAVGSNIVVSTR.T			Oxidation (M)
1917.8527	1918.8694	5	277	291	R.MHEDINEEWISDKTR.F			Oxidation (M)
968.508	969.5104	-5	292	299	R.FAYDGLKR.Q			
1610.794	1611.8	-1	312	325	K.GLLTYTSWEDALSR.V			
1181.5751	1182.5931	9	326	336	R.VAGMLQSFEGK.A			Oxidation (M)
1063.5451	1064.5558	3	409	417	R.FEAPLFNAR.I			
1557.7787	1558.7979	8	451	464	K.ILQDIASGNHEFSK.V			
1400.781	1401.7819	-5	471	483	K.KPMVVLGSSALQR.D			Oxidation (M)
2070.1473	2071.1294	-12	519	538	R.IASQVAALDLGYKPGVEAIR.K			
1537.8253	1538.8057	-17	608	621	K.VAVTPPGLAREDWK.I			
2074.0946	2075.1047	1	625	643	R.ALSEIAGITLPYDTLDQVR.N			

Spot 339 (D4 20080606) - 6-phosphogluconolactonase (Pgls)

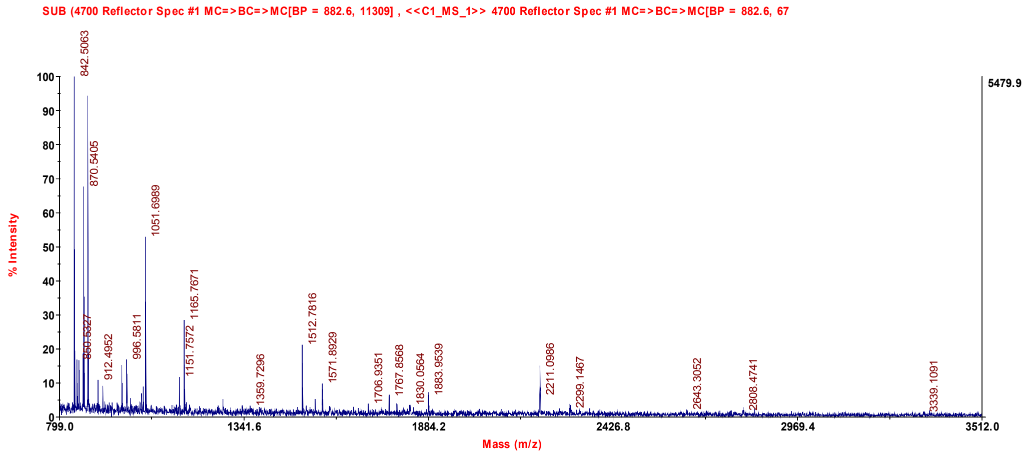

Spot 339 (20080606-D4) Pgls								
PMF/MALDI								
Calculated	Observed	+/-ppm	start	end	Sequence	Ion Score	C.I.%	Modification
1511.7732	1512.7817	1	91	106	R.DLPAATAPAGPASFAR.W			
1766.8628	1767.8569	-7	116	130	R.LVPFDHAESTYGLYR.T			
2807.4229	2808.4744	16	137	162	K.LPIPDSQVLTIDPALPVEDAAEDYAR.K			
1570.8831	1571.8931	2	205	219	K.IVAPIGDSPKPPPQR.V			
1882.9458	1883.9539	0	249	265	R.ILEDQESALPAAMVQPR.T			Oxidation (M)

Spot 342 (A24 20080606) - Inner membrane protein, mitochondrial, isoform CRA_a (Immt)

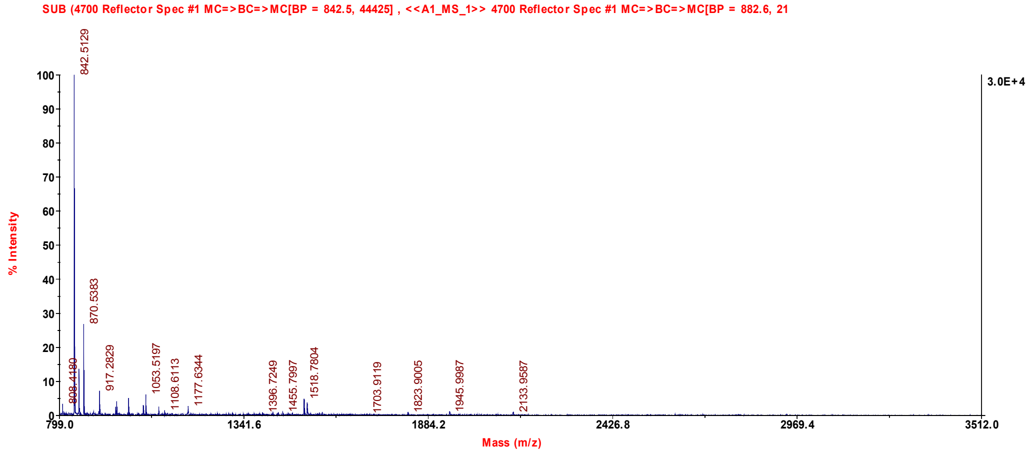

Spot 342 (20080606-A24) Immt								
PMF/MALDI								
Calculated	Observed	+/-ppm	start	end	Sequence	Ion Score	C.I.%	Modification
966.5862	967.5931	0	102	110	K.KPIQSGPLK.I			
1517.7685	1518.7804	3	175	188	K.SLEDALNQTATVTR.Q			
1441.7889	1442.8073	8	189	202	R.QTITAQNAAVQAVK.A			
1562.7828	1563.7919	1	268	282	R.EIAGATPYITAAEEK.L			
1454.7803	1455.7997	8	283	295	K.LHSMIVDLDSVVK.K			
1526.8205	1527.8331	3	306	318	K.VVSQYHELVVQAR.D			
1570.7879	1571.8281	21	324	337	K.ELDSITPDITPGWK.G			
1107.6036	1108.6113	0	380	388	K.QHIELALER.Q			
807.4127	808.418	-2	396	403	R.AFDSAVAK.A			
2131.948	2132.9565	1	480	497	R.SQEQMDNFTLDINTAYAR.L			Oxidation (M)
1822.8809	1823.9005	7	500	516	R.GIEQAVQSHAVAEEEAR.K			
1944.9826	1945.9987	5	534	552	K.TSSAEMPTIPLGSAVEAIR.V			Oxidation (M)
1052.5138	1053.5197	-1	576	584	R.GVYSEETLR.A			

Spot 344 (D8 20070719) – mixture of: Tubulin alpha-3 chain (Tuba3), Tubulin alpha-8 chain (Tuba8), Tubulin alpha-1A chain (Tuba1a), Tubulin alpha-1B chain (Tuba1b), Tubulin alpha-1C chain (Tuba1c) and Tubulin alpha-8 chain (Tuba8)

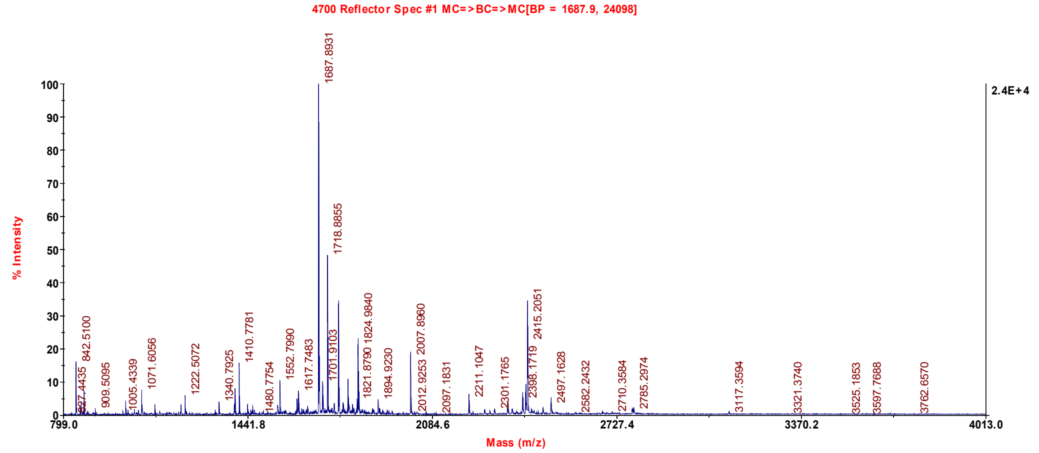

Spot 344 (20070719-D8) Tuba3								
MS+MS/MS								
Calculated	Observed	+/-ppm	start	end	Sequence	Ion Score	C.I.%	Modification
1015.5782	1015.5765	-2	327	336	DVNAAIATIK			
1071.6045	1071.6045	0	113	121	EIVDLVLDR			
1340.7897	1340.7898	0	113	123	EIVDLVLDRIR			
1396.693	1396.6952	2	391	401	LDHKFDLMYAK			Oxidation (M)
1410.7739	1410.7773	2	85	96	QLFHPEQLITGK			
1410.7739	1410.7773	2	85	96	QLFHPEQLITGK	62	100	
1457.8685	1457.8756	5	230	243	LIGQIVSSITASLR			
1552.7941	1552.7986	3	391	402	LDHKFDLMYAKR			Oxidation (M)
1552.7941	1552.7986	3	391	402	LDHKFDLMYAKR	4	47.118	Oxidation (M)
1687.8901	1687.8923	1	65	79	AVFVDLEPTVVDEVR			
1687.8901	1687.8923	1	65	79	AVFVDLEPTVVDEVR	103	100	
1718.8821	1718.8857	2	216	229	NLDIERPTYTNLNR			
1718.8821	1718.8857	2	216	229	NLDIERPTYTNLNR	24	99.486	
1756.9633	1756.964	0	265	280	IHFPLATYAPVISAEK			
1756.9633	1756.964	0	265	280	IHFPLATYAPVISAEK	133	100	
1824.9854	1824.9844	-1	353	370	VGINYQPPTVVPGGDLAK	47	99.998	
1824.9854	1824.9844	-1	353	370	VGINYQPPTVVPGGDLAK			
1827.9963	1828.0005	2	106	121	GHYTIGKEIVDLVLDR			
1894.8318	1894.9236	48	312	326	YMACCMLYRGDVVPK			2 Carbamidomethyl (C); 2 Oxidation (M)
2007.8929	2007.8955	1	41	60	TIGGGDDSFNTFFSETGAGK	130	100	
2007.8929	2007.8955	1	41	60	TIGGGDDSFNTFFSETGAGK			
2266.1714	2266.1824	5	65	84	AVFVDLEPTVVDEVRTGTYR			
2346.0132	2346.0085	-2	403	422	AFVHWYVGEGMEEGEFSEAR			Oxidation (M)
2409.2085	2409.209	0	244	264	FDGALNVDLTEFQTNLVPYPR			
2415.2051	2415.207	1	85	105	QLFHPEQLITGKEDAANNYAR	63	100	
2415.2051	2415.207	1	85	105	QLFHPEQLITGKEDAANNYAR			
2497.1741	2497.1648	-4	41	64	TIGGGDDSFNTFFSETGAGKHVPR			
3117.3428	3117.3591	5	423	450	EDLAALEKDYEEVGVDSVEAEAEEGEEY			

Spot 344 (20070719-D8) Tuba3								
PMF/MALDI								
Calculated	Observed	+/-ppm	start	end	Sequence	Ion Score	C.I.%	Modification
2006.8858	2007.896	1	41	60	K.TIGGGDDSFNTFFSETGAGK.H			
2496.167	2497.1628	-5	41	64	K.TIGGGDDSFNTFFSETGAGKHVPR.A			
1686.8829	1687.8931	2	65	79	R.AVFVDLEPTVVDEVR.T			
2265.1641	2266.1775	3	65	84	R.AVFVDLEPTVVDEVRTGTYR.Q			
1409.7667	1410.7781	3	85	96	R.QLFHPEQLITGK.E			
2414.1978	2415.2051	0	85	105	R.QLFHPEQLITGKEDAANNYAR.G			
1022.4417	1023.4598	11	97	105	K.EDAANNYAR.G			
1826.989	1828.0007	2	106	121	R.GHYTIGKEIVDLVLDR.I			
1070.5972	1071.6056	1	113	121	K.EIVDLVLDR.I			
1339.7823	1340.7925	2	113	123	K.EIVDLVLDRIR.K			
908.4967	909.5095	6	157	164	R.LSVDYGKK.S			
1873.9758	1874.9896	3	215	229	R.RNLDIERPTYTNLNR.L			
1717.8747	1718.8855	2	216	229	R.NLDIERPTYTNLNR.L			
1456.8613	1457.876	5	230	243	R.LIGQIVSSITASLR.F			
2408.2012	2409.2073	-1	244	264	R.FDGALNVDLTEFQTNLVPYPR.I			
1755.9559	1756.9663	2	265	280	R.IHFPLATYAPVISAEK.A			
1609.9039	1610.897	-9	321	336	R.GDVVPKDVNAAIATIK.T			
1014.5709	1015.5777	-1	327	336	K.DVNAAIATIK.T			
1754.845	1755.89	21	339	352	K.RTIQFVDWCPTGFK.V			
1823.9782	1824.984	-1	353	370	K.VGINYQPPTVVPGGDLAK.V			
1395.6857	1396.6965	3	391	401	R.LDHKFDLMYAK.R			Oxidation (M)
886.4259	887.4356	3	395	401	K.FDLMYAK.R			
902.4208	903.4473	21	395	401	K.FDLMYAK.R			Oxidation (M)
1058.5219	1059.5331	4	395	402	K.FDLMYAKR.A			Oxidation (M)
2501.107	2502.1792	26	402	422	K.RAFVHWYVGEGMEEGEFSEAR.E			Oxidation (M)
2345.0059	2346.0068	-3	403	422	R.AFVHWYVGEGMEEGEFSEAR.E			Oxidation (M)
3116.3357	3117.3594	5	423	450	R.EDLAALEKDYEEVGVDSVEAEAEEGEEY.-			

Spot 344 (20070719-D8) Tuba1a
MS+MS/MS

Calculated	Observed	+/-ppm	start	end	Sequence	Ion Score	C.I.%	Modification
1015.5782	1015.5765	-2	327	336	DVNAAIATIK			
1396.693	1396.6952	2	391	401	LDHKFDLMYAK			Oxidation (M)
1410.7739	1410.7773	2	85	96	QLFHPEQLITGK			
1410.7739	1410.7773	2	85	96	QLFHPEQLITGK	62	100	
1457.8685	1457.8756	5	230	243	LIGQIVSSITASLR			
1552.7941	1552.7986	3	391	402	LDHKFDLMYAKR			Oxidation (M)
1552.7941	1552.7986	3	391	402	LDHKFDLMYAKR	4	47.118	Oxidation (M)
1701.9058	1701.9078	1	65	79	AVFVDLEPTVIDEVR			
1701.9058	1701.9078	1	65	79	AVFVDLEPTVIDEVR	45	99.996	
1718.8821	1718.8857	2	216	229	NLDIERPTYTNLNR			
1718.8821	1718.8857	2	216	229	NLDIERPTYTNLNR	24	99.486	
1756.9633	1756.964	0	265	280	IHFPLATYAPVISAEK			
1756.9633	1756.964	0	265	280	IHFPLATYAPVISAEK	133	100	
1824.9854	1824.9844	-1	353	370	VGINYQPPTVVPGGDLAK	47	99.998	
1824.9854	1824.9844	-1	353	370	VGINYQPPTVVPGGDLAK			
2007.8929	2007.8955	1	41	60	TIGGGDDSFNTFFSETGAGK	130	100	
2007.8929	2007.8955	1	41	60	TIGGGDDSFNTFFSETGAGK			
2346.0132	2346.0085	-2	403	422	AFVHWYVGEGMEEGEFSEAR			Oxidation (M)
2409.2085	2409.209	0	244	264	FDGALNVDLTEFQTNLVPYPR			
2415.2051	2415.207	1	85	105	QLFHPEQLITGKEDAANNYAR	63	100	
2415.2051	2415.207	1	85	105	QLFHPEQLITGKEDAANNYAR			
2497.1741	2497.1648	-4	41	64	TIGGGDDSFNTFFSETGAGKHVPR			

Spot 344 (20070719-D8) Tuba1a
PMF/MALDI

Calculated	Observed	+/-ppm	start	end	Sequence	Ion Score	C.I.%	Modification
2006.8858	2007.896	1	41	60	K.TIGGGDDSFNTFFSETGAGK.H			
2496.167	2497.1628	-5	41	64	K.TIGGGDDSFNTFFSETGAGKHVPR.A			
1700.8985	1701.9103	3	65	79	R.AVFVDLEPTVIDEVR.T			
1409.7667	1410.7781	3	85	96	R.QLFHPEQLITGK.E			
2414.1978	2415.2051	0	85	105	R.QLFHPEQLITGKEDAANNYAR.G			
1022.4417	1023.4598	11	97	105	K.EDAANNYAR.G			
908.4967	909.5095	6	157	164	R.LSVDYGKK.S			
1873.9758	1874.9896	3	215	229	R.RNLDIERPTYTNLNR.L			
1717.8747	1718.8855	2	216	229	R.NLDIERPTYTNLNR.L			
1456.8613	1457.876	5	230	243	R.LIGQIVSSITASLR.F			
2408.2012	2409.2073	-1	244	264	R.FDGALNVDLTEFQTNLVPYPR.I			
1755.9559	1756.9663	2	265	280	R.IHFPLATYAPVISAEK.A			
1609.9039	1610.897	-9	321	336	R.GDVVPKDVNAAIATIK.T			
1014.5709	1015.5777	-1	327	336	K.DVNAAIATIK.T			
1754.845	1755.89	21	339	352	K.RTIQFVDWCPTGFK.V			
1823.9782	1824.984	-1	353	370	K.VGINYQPPTVVPGGDLAK.V			
1395.6857	1396.6965	3	391	401	R.LDHKFDLMYAK.R			Oxidation (M)
886.4259	887.4356	3	395	401	K.FDLMYAK.R			
902.4208	903.4473	21	395	401	K.FDLMYAK.R			Oxidation (M)
1058.5219	1059.5331	4	395	402	K.FDLMYAKR.A			Oxidation (M)
2501.107	2502.1792	26	402	422	K.RAFVHWYVGEGMEEGEFSEAR.E			Oxidation (M)
2345.0059	2346.0068	-3	403	422	R.AFVHWYVGEGMEEGEFSEAR.E			Oxidation (M)

Spot 344 (20070719-D8) Tuba1b
MS+MS/MS

Calculated	Observed	+/-ppm	start	end	Sequence	Ion Score	C.I.%	Modification
1015.5782	1015.5765	-2	327	336	DVNAAIATIK			
1396.693	1396.6952	2	391	401	LDHKFDLMYAK			Oxidation (M)
1410.7739	1410.7773	2	85	96	QLFHPEQLITGK			
1410.7739	1410.7773	2	85	96	QLFHPEQLITGK	62	100	
1552.7941	1552.7986	3	391	402	LDHKFDLMYAKR			Oxidation (M)
1552.7941	1552.7986	3	391	402	LDHKFDLMYAKR	4	47.118	Oxidation (M)
1701.9058	1701.9078	1	65	79	AVFVDLEPTVIDEVR			
1701.9058	1701.9078	1	65	79	AVFVDLEPTVIDEVR	45	99.996	
1718.8821	1718.8857	2	216	229	NLDIERPTYTNLNR			
1718.8821	1718.8857	2	216	229	NLDIERPTYTNLNR	24	99.486	
1740.8527	1740.8761	13	339	352	RSIQFVDWCPTGFK			Carbamidomethyl (C)
1756.9633	1756.964	0	265	280	IHFPLATYAPVISAEK			
1756.9633	1756.964	0	265	280	IHFPLATYAPVISAEK	133	100	
1824.9854	1824.9844	-1	353	370	VGINYQPPTVVPGGDLAK	47	99.998	
1824.9854	1824.9844	-1	353	370	VGINYQPPTVVPGGDLAK			
2007.8929	2007.8955	1	41	60	TIGGGDDSFNTFFSETGAGK	130	100	
2007.8929	2007.8955	1	41	60	TIGGGDDSFNTFFSETGAGK			
2346.0132	2346.0085	-2	403	422	AFVHWYVGEGMEEGEFSEAR			Oxidation (M)
2409.2085	2409.209	0	244	264	FDGALNVDLTEFQTNLVPYPR			
2415.2051	2415.207	1	85	105	QLFHPEQLITGKEDAANNYAR	63	100	
2415.2051	2415.207	1	85	105	QLFHPEQLITGKEDAANNYAR			
2497.1741	2497.1648	-4	41	64	TIGGGDDSFNTFFSETGAGKHVPR			

Spot 344 (20070719-D8) Tuba1b
PMF/MALDI

Calculated	Observed	+/-ppm	start	end	Sequence	Ion Score	C.I.%	Modification
2006.8858	2007.896	1	41	60	K.TIGGGDDSFNTFFSETGAGK.H			
2496.167	2497.1628	-5	41	64	K.TIGGGDDSFNTFFSETGAGKHVPR.A			
1700.8985	1701.9103	3	65	79	R.AVFVDLEPTVIDEVR.T			
1409.7667	1410.7781	3	85	96	R.QLFHPEQLITGK.E			
2414.1978	2415.2051	0	85	105	R.QLFHPEQLITGKEDAANNYAR.G			
1022.4417	1023.4598	11	97	105	K.EDAANNYAR.G			
908.4967	909.5095	6	157	164	R.LSVDYGKK.S			
1873.9758	1874.9896	3	215	229	R.RNLDIERPTYTNLNR.L			
1717.8747	1718.8855	2	216	229	R.NLDIERPTYTNLNR.L			
2408.2012	2409.2073	-1	244	264	R.FDGALNVDLTEFQTNLVPYPR.I			
1755.9559	1756.9663	2	265	280	R.IHFPLATYAPVISAEK.A			
1609.9039	1610.897	-9	321	336	R.GDVVPKDVNAAIATIK.T			
1014.5709	1015.5777	-1	327	336	K.DVNAAIATIK.T			
1740.8294	1741.8292	-4	339	352	K.RSIQFVDWCPTGFK.V			
1823.9782	1824.984	-1	353	370	K.VGINYQPPTVVPGGDLAK.V			
1395.6857	1396.6965	3	391	401	R.LDHKFDLMYAK.R			Oxidation (M)
886.4259	887.4356	3	395	401	K.FDLMYAK.R			
902.4208	903.4473	21	395	401	K.FDLMYAK.R			Oxidation (M)
1058.5219	1059.5331	4	395	402	K.FDLMYAKR.A			Oxidation (M)
2501.107	2502.1792	26	402	422	K.RAFVHWYVGEGMEEGEFSEAR.E			Oxidation (M)
2345.0059	2346.0068	-3	403	422	R.AFVHWYVGEGMEEGEFSEAR.E			Oxidation (M)

Spot 344 (20070719-D8) Tuba1c
MS+MS/MS

Calculated	Observed	+/-ppm	start	end	Sequence	Ion Score	C.I.%	Modification
1015.5782	1015.5765	-2	327	336	DVNAAIATIK			
1396.693	1396.6952	2	391	401	LDHKFDLMYAK			Oxidation (M)
1410.7739	1410.7773	2	85	96	QLFHPEQLITGK			
1410.7739	1410.7773	2	85	96	QLFHPEQLITGK	62	100	
1552.7941	1552.7986	3	391	402	LDHKFDLMYAKR			Oxidation (M)
1552.7941	1552.7986	3	391	402	LDHKFDLMYAKR	4	47.118	Oxidation (M)
1701.9058	1701.9078	1	65	79	AVFVDLEPTVIDEVR			
1701.9058	1701.9078	1	65	79	AVFVDLEPTVIDEVR	45	99.996	
1718.8821	1718.8857	2	216	229	NLDIERPTYTNLNR			
1718.8821	1718.8857	2	216	229	NLDIERPTYTNLNR	24	99.486	
1756.9633	1756.964	0	265	280	IHFPLATYAPVISAEK			
1756.9633	1756.964	0	265	280	IHFPLATYAPVISAEK	133	100	
2007.8929	2007.8955	1	41	60	TIGGGDDSFNTFFSETGAGK	130	100	
2007.8929	2007.8955	1	41	60	TIGGGDDSFNTFFSETGAGK			
2346.0132	2346.0085	-2	403	422	AFVHWYVGEGMEEGEFSEAR			Oxidation (M)
2409.2085	2409.209	0	244	264	FDGALNVDLTEFQTNLVPYPR			
2415.2051	2415.207	1	85	105	QLFHPEQLITGKEDAANNYAR	63	100	
2415.2051	2415.207	1	85	105	QLFHPEQLITGKEDAANNYAR			
2497.1741	2497.1648	-4	41	64	TIGGGDDSFNTFFSETGAGKHVPR			
2780.3018	2780.312	4	281	304	AYHEQLTVAEITNACFEPANQMVK			Carbamidomethyl (C); Oxidation (M)

Spot 344 (20070719-D8) Tuba1c
PMF/MALDI

Calculated	Observed	+/-ppm	start	end	Sequence	Ion Score	C.I.%	Modification
2006.8858	2007.896	1	41	60	K.TIGGGDDSFNTFFSETGAGK.H			
2496.167	2497.1628	-5	41	64	K.TIGGGDDSFNTFFSETGAGKHVPR.A			
1700.8985	1701.9103	3	65	79	R.AVFVDLEPTVIDEVR.T			
1409.7667	1410.7781	3	85	96	R.QLFHPEQLITGK.E			
2414.1978	2415.2051	0	85	105	R.QLFHPEQLITGKEDAANNYAR.G			
1022.4417	1023.4598	11	97	105	K.EDAANNYAR.G			
908.4967	909.5095	6	157	164	R.LSVDYGKK.S			
1873.9758	1874.9896	3	215	229	R.RNLDIERPTYTNLNR.L			
1717.8747	1718.8855	2	216	229	R.NLDIERPTYTNLNR.L			
2408.2012	2409.2073	-1	244	264	R.FDGALNVDLTEFQTNLVPYPR.I			
1755.9559	1756.9663	2	265	280	R.IHFPLATYAPVISAEK.A			
1609.9039	1610.897	-9	321	336	R.GDVVPKDVNAAIATIK.T			
1014.5709	1015.5777	-1	327	336	K.DVNAAIATIK.T			
1754.845	1755.89	21	339	352	K.RTIQFVDWCPTGFK.V			
1851.9843	1852.9657	-14	353	370	K.VGINYQPPTVVPGGDLAR.V			
1395.6857	1396.6965	3	391	401	R.LDHKFDLMYAK.R			Oxidation (M)
886.4259	887.4356	3	395	401	K.FDLMYAK.R			
902.4208	903.4473	21	395	401	K.FDLMYAK.R			Oxidation (M)
1058.5219	1059.5331	4	395	402	K.FDLMYAKR.A			Oxidation (M)
2501.107	2502.1792	26	402	422	K.RAFVHWYVGEGMEEGEFSEAR.E			Oxidation (M)
2345.0059	2346.0068	-3	403	422	R.AFVHWYVGEGMEEGEFSEAR.E			Oxidation (M)

Spot 344 (20070719-D8) Tuba8
MS+MS/MS

Calculated	Observed	+/-ppm	start	end	Sequence	Ion Score	C.I.%	Modification
1396.693	1396.6952	2	391	401	LDHKFDLMYAK			Oxidation (M)
1410.7739	1410.7773	2	85	96	QLFHPEQLITGK			
1410.7739	1410.7773	2	85	96	QLFHPEQLITGK	62	100	
1552.7941	1552.7986	3	391	402	LDHKFDLMYAKR			Oxidation (M)
1552.7941	1552.7986	3	391	402	LDHKFDLMYAKR	4	51.931	Oxidation (M)
1687.8571	1687.8923	21	65	79	AVMVDLEPTVVDEVR			Oxidation (M)
1687.8571	1687.8923	21	65	79	AVMVDLEPTVVDEVR	103	100	Oxidation (M)
1718.8821	1718.8857	2	216	229	NLDIERPTYTNLNR			
1718.8821	1718.8857	2	216	229	NLDIERPTYTNLNR	24	99.532	
1824.9854	1824.9844	-1	353	370	VGINYQPPTVVPGGDLAK	47	99.998	
1824.9854	1824.9844	-1	353	370	VGINYQPPTVVPGGDLAK			
1894.8318	1894.9236	48	312	326	YMACCMLYRGDVVPK			2 Carbamidomethyl (C); 2 Oxidation (M)
2346.0132	2346.0085	-2	403	422	AFVHWYVGEGMEEGEFSEAR			Oxidation (M)
2409.2085	2409.209	0	244	264	FDGALNVDLTEFQTNLVPYPR			
2415.2051	2415.207	1	85	105	QLFHPEQLITGKEDAANNYAR	63	100	
2415.2051	2415.207	1	85	105	QLFHPEQLITGKEDAANNYAR			

Spot 350 (D7 20080606) – mixture of: Cytosol aminopeptidase (Leucine aminopeptidase 3) (Lap3) and Pre-mRNA-processing factor 19 (Nuclear matrix protein SNEV [Mus musculus]) (Prpf19)

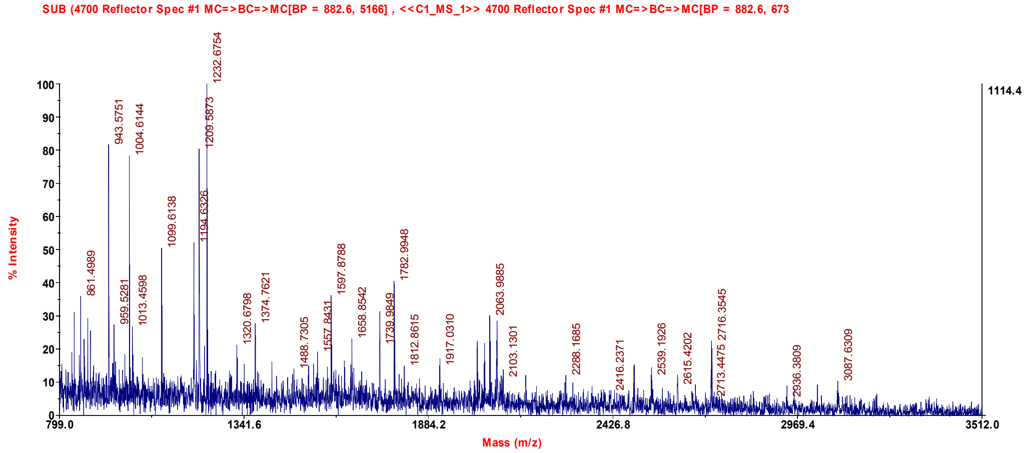

Spot 350 (20080606-D7) Lap3								
PMF/MALDI								
Calculated	Observed	+/-ppm	start	end	Sequence	Ion Score	C.I.%	Modification
2025.8916	2026.9033	2	44	61	K.DKDDDVPQFTSAGENFNK.L			
2082.9355	2083.95	3	105	122	R.SAGVDDQENWHEGKENIR.A			
1231.6673	1232.6754	1	189	200	K.GVLFASGQNLAR.Q			
1635.7232	1636.7472	10	201	214	R.QLMESPANEMTPTR.F			2 Oxidation (M)
2085.0088	2085.97	-22	236	253	R.TKSWIEEQEMGSFLSVAK.G			Oxidation (M)
3084.5444	3085.5066	-15	254	282	K.GSEEPPVFLEIHYTGSPNATEAPLVFVGK.G			
1193.6292	1194.6326	-3	283	294	K.GITFDSGGISIK.A			
2715.2336	2716.3545	42	295	321	K.ASANMDLMRADMGGAATICSAIVSAAK.L			2 Oxidation (M)
1663.8318	1664.8372	-1	418	431	K.LFEASVETGDRVWR.M			
1208.5648	1209.5873	13	432	440	R.MPLFEHYTR.Q			Oxidation (M)
1003.6066	1004.6144	1	506	513	R.TLIEFLLR.F			

Spot 350 (20080606-D7) Prpf19								
PMF/MALDI								
Calculated	Observed	+/-ppm	start	end	Sequence	Ion Score	C.I.%	Modification
2046.9833	2047.9907	0	77	93	K.ALQDEWDAVMLHSFTLR.Q			Oxidation (M)
1373.7514	1374.7621	2	176	187	K.LQDKATVLTTER.K			
889.4869	890.4869	-8	180	187	K.ATVLTTER.K			
1781.9774	1782.9948	6	191	206	R.GKTVPEELVKPEELSK.Y			
1596.861	1597.8788	7	193	206	K.TVPEELVKPEELSK.Y			
1916.0255	1917.031	-1	193	208	K.TVPEELVKPEELSKYR.Q			
2614.3755	2615.4202	14	266	289	K.KVTSVVFHPSQELVFSASPDATIR.I			
2486.2806	2487.282	-2	267	289	K.VTSVVFHPSQELVFSASPDATIR.I			
958.5236	959.5281	-3	376	382	K.IWDLKER.T			
1319.6721	1320.6798	0	429	439	K.TLQLDNNFEVK.S			
1012.4648	1013.4598	-12	489	497	K.FIASTGMDR.S			Oxidation (M)

Spot 351 (D10 20080606) - Protein disulfide isomerase (P4hb, Pdia1)

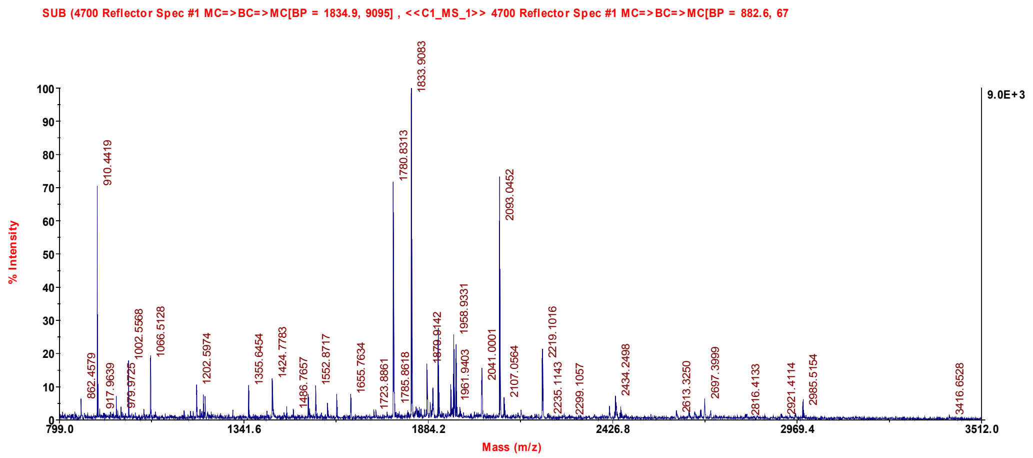

Spot 351 (20080606-D10) P4hb, Pdia1								
PMF/MALDI								
Calculated	Observed	+/-ppm	start	end	Sequence	Ion Score	C.I.%	Modification
861.4596	862.4579	-10	60	67	K.ALAPEYAK.A			
1001.5505	1002.5568	-1	72	80	K.LKAEGSEIR.L			
2092.0436	2093.0452	-3	81	99	R.LAKVDATEESDLAQQYGVR.G			
1779.8275	1780.8313	-2	84	99	K.VDATEESDLAQQYGVR.G			
1465.6797	1466.6812	-4	109	122	K.NGDTASPKEYTAGR.E			
2649.267	2650.292	7	109	132	K.NGDTASPKEYTAGREADDIVNWLK.K			
1878.9112	1879.9142	-2	117	132	K.EYTAGREADDIVNWLK.K			
1201.5979	1202.5974	-6	123	132	R.EADDIVNWLK.K			
1485.7939	1486.7657	-24	123	134	R.EADDIVNWLKKR.T			
2984.4866	2985.5154	7	135	164	R.TGPAATTLSDTAAAESLVDSSEVTVIGFFK.D			
1423.7711	1424.7783	0	198	209	K.YQLDKDGVVLFK.K			
1551.8661	1552.8717	-1	198	210	K.YQLDKDGVVLFKK.F			
1654.7587	1655.7634	-2	211	224	K.FDEGRNNFEGEITK.E			
1911.8962	1912.9025	-1	211	226	K.FDEGRNNFEGEITKEK.L			
1964.0367	1965.0376	-3	233	249	K.HNQLPLVIEFTEQTAPK.I			
1458.6991	1459.6934	-9	266	278	K.SVSDYDGKLSNFK.K			
1586.794	1587.8044	2	266	279	K.SVSDYDGKLSNFKK.A			
1832.9057	1833.9083	-3	288	302	K.ILFIFIDSDHTDNQR.I			
965.5586	966.5621	-4	303	310	R.ILEFFGLK.K			
1221.6162	1222.6202	-3	319	328	R.LITLEEEMTK.Y			Oxidation (M)
2612.2778	2613.325	15	319	340	R.LITLEEEMTKYKPESDELTAEK.I			Oxidation (M)
1408.6722	1409.6628	-12	329	340	K.YKPESDELTAEK.I			
2433.2362	2434.2498	3	353	372	K.IKPHLMSQELPEDWDKQPVK.V			Oxidation (M)
1722.8828	1723.8861	-2	373	387	K.VLVGKNFEEVAFDEK.K			
1850.9778	1852.0033	10	373	388	K.VLVGKNFEEVAFDEKK.N			
1226.5455	1227.5547	2	378	387	K.NFEEVAFDEK.K			
1354.6405	1355.6454	-2	378	388	K.NFEEVAFDEKK.N			
1728.9046	1729.9036	-5	412	426	K.LGETYKDHENIVIAK.M			
2202.0991	2203.1057	0	427	446	K.MDSTANEVEAVKVHSFPTLK.F			
2218.094	2219.1016	0	427	446	K.MDSTANEVEAVKVHSFPTLK.F			Oxidation (M)
909.4345	910.4419	0	447	454	K.FFPASADR.T			
1956.933	1957.9414	1	447	463	K.FFPASADRTVIDYNGER.T			
1065.5091	1066.5128	-3	455	463	R.TVIDYNGER.T			

Spot 352 (B5 20080606) - COP9 signalosome complex subunit 4 (Cops4)

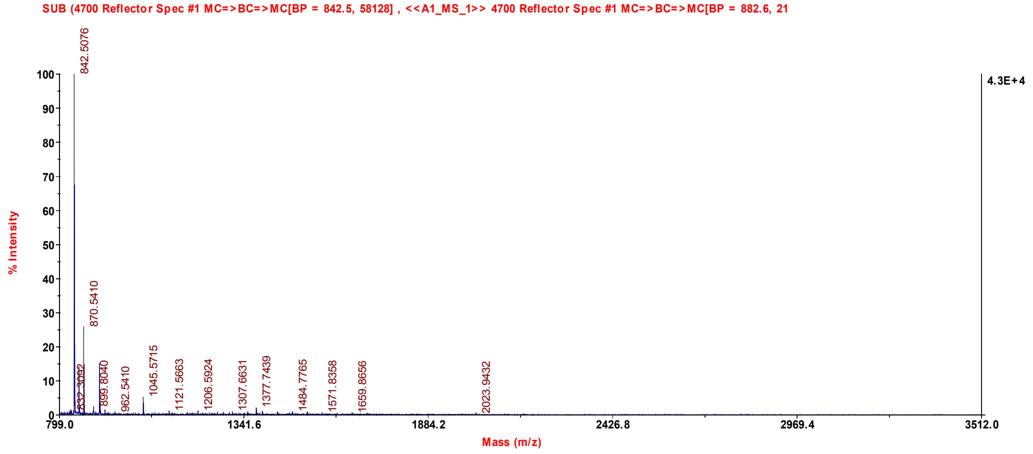

Spot 352 (20080606-B5) Cops4								
MS+MS/MS								
Calculated	Observed	+/-ppm	start	end	Sequence	Ion Score	C.I.%	Modification
834.4468	834.4577	13	201	207	FIEAAQR			
962.5417	962.5405	-1	200	207	KFIEAAQR			
1121.5619	1121.5663	4	338	347	IASQMITEGR			Oxidation (M)
1206.5961	1206.5956	0	291	302	ATTADGSSILDR			
1239.6401	1239.6414	1	245	254	MLATLFKDER			Oxidation (M)
1281.7161	1281.6975	-15	303	314	AVIEHNLLSASK			
1377.7372	1377.7427	4	96	107	VISFEEQVASIR			
1377.7372	1377.7427	4	96	107	VISFEEQVASIR	12	93.582	
2023.9607	2023.9435	-8	154	170	LYLEDDDPVQAEAYINR			
2023.9607	2023.9435	-8	154	170	LYLEDDDPVQAEAYINR	21	99.185	
3030.4771	3030.4241	-17	275	302	GNQLQEFAAMLMPHQKATTADGSSILDR			

Spot 352 (20080606-B5) Cops4								
PMF/MALDI								
Calculated	Observed	+/-ppm	start	end	Sequence	Ion Score	C.I.%	Modification
1376.73	1377.7421	4	96	107	R.VISFEEQVASIR.Q			
2022.9534	2023.9425	-9	154	170	R.LYLEDDDPVQAEAYINR.A			
820.4555	821.459	-5	194	199	R.VLDYRR.K			
961.5345	962.5381	-4	200	207	R.KFIEAAQR.Y			
833.4395	834.4591	15	201	207	K.FIEAAQR.Y			
1238.6329	1239.6398	0	245	254	R.MLATLFKDER.C			Oxidation (M)
2224.1609	2225.1296	-17	272	290	R.IIRGNQLQEFAAMLMPHQK.A			
3029.4699	3030.4172	-20	275	302	R.GNQLQEFAAMLMPHQKATTADGSSILDR.A			
1205.5888	1206.5944	-1	291	302	K.ATTADGSSILDR.A			
1280.7088	1281.7002	-12	303	314	R.AVIEHNLLSASK.L			
1120.5546	1121.5657	3	338	347	K.IASQMITEGR.M			Oxidation (M)
1990.9571	1991.9879	12	348	364	R.MNGFIDQIDGIVHFETR.E			

Spot 356 (B15 20080606) - Splicing factor 3a, subunit 1 (Sf3a1)

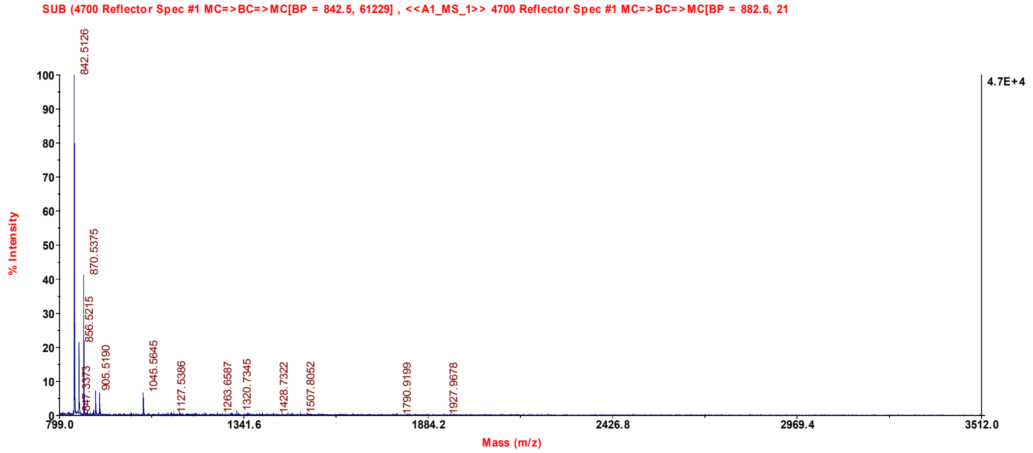

Spot 356 (20080606-B15) Sf3a1								
PMF/MALDI								
Calculated	Observed	+/-ppm	start	end	Sequence	Ion Score	C.I.%	Modification
1319.7197	1320.7345	6	51	62	R.NIVDKTASFVAR.N			
918.4195	919.4335	7	63	70	R.NGPEFEAR.I			
1324.6986	1325.6404	-49	103	115	K.EGKAQEPSAAIPK.V			
1926.9582	1927.9678	1	116	131	K.VMQQQQQATQQQLPQK.V			Oxidation (M)
904.513	905.519	-1	169	176	K.LTAQFVAR.N			
879.4702	880.4664	-13	211	217	K.LVEQYTK.I			
846.3476	847.3373	-21	423	428	K.MQEHMR.I			Oxidation (M)
1506.793	1507.8052	3	472	485	R.TDIFGVEETAIGKK.I			
1427.7143	1428.7322	7	486	497	K.IGEEEIQKPEEK.V			
841.5021	842.5126	4	699	706	R.NKGPVSIK.V			
1126.5441	1127.5386	-11	742	752	K.IHEATGMPAGK.Q			

Spot 360 (E3 20070719) – Proteasome subunit alpha type 6 (Psma6)

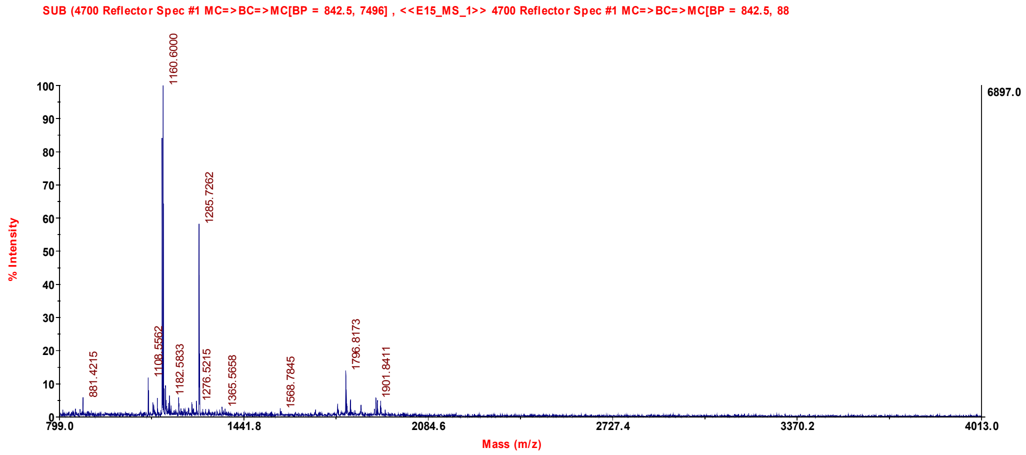

Spot 360 (20070719-E3) Psma6								
**MS+MS/MS**								
Calculated	Observed	+/-ppm	start	end	Sequence	Ion Score	C.I.%	Modification
881.4151	881.4256	12	96	102	YEAANWK			
1108.5535	1108.5562	2	94	102	ARYEAANWK			
1108.5535	1108.5562	2	94	102	ARYEAANWK			
1156.611	1156.6102	-1	12	21	HITIFSPEGR			
1160.5986	1160.5981	0	22	30	LYQVEYAFK			
1160.5986	1160.5981	0	22	30	LYQVEYAFK	44	99.996	
1285.7223	1285.726	3	31	43	AINQGGLTSVAVR	10	90.066	
1285.7223	1285.726	3	31	43	AINQGGLTSVAVR			
1767.9122	1767.9266	8	165	181	ATAAGVKQTESTSFLEK			
1794.8441	1794.8049	-22	103	116	YKYGYEIPVDMLCK			Carbamidomethyl (C); Oxidation (M)
1933.9514	1933.9553	2	4	21	GSSAGFDRHITIFSPEGR			

Spot 360 (20070719-E3) Psma6								
**PMF/MALDI**								
Calculated	Observed	+/-ppm	start	end	Sequence	Ion Score	C.I.%	Modification
1932.9442	1933.9255	-13	4	21	R.GSSAGFDRHITIFSPEGR.L			
1155.6037	1156.6105	0	12	21	R.HITIFSPEGR.L			
1159.5913	1160.6	1	22	30	R.LYQVEYAFK.A			
1284.715	1285.7262	3	31	43	K.AINQGGLTSVAVR.G			
1107.5461	1108.5562	3	94	102	R.ARYEAANWK.Y			
880.4079	881.4215	7	96	102	R.YEAANWK.Y			
1766.905	1767.9244	7	165	181	K.ATAAGVKQTESTSFLEK.K			
1168.5612	1169.5929	21	172	181	K.QTESTSFLEK.K			

Spot 375 (A8 20080606) – mixture of: Ras-related protein Rab-2A (Rab2a) and Protein DJ-1 (Park7, Cap1)

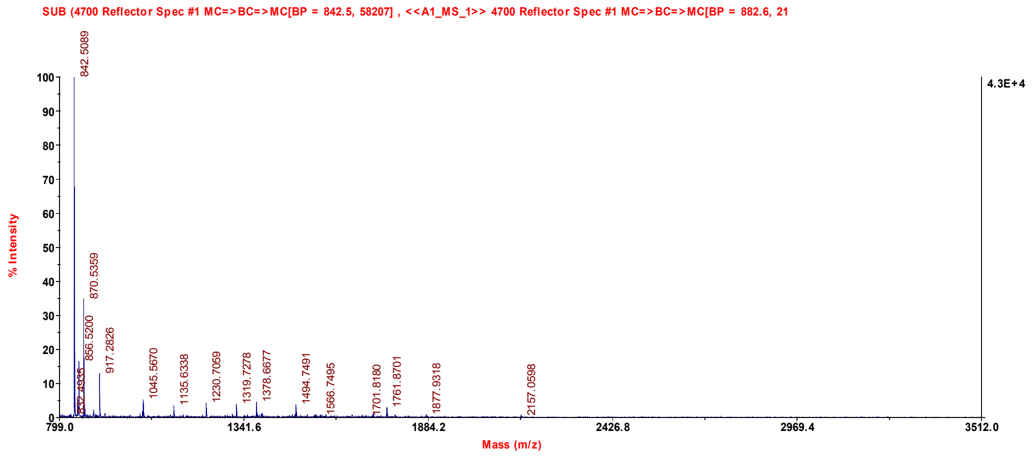

Spot 375 (20080606-A8) Rab2a								
**MS+MS/MS**								
Calculated	Observed	+/-ppm	start	end	Sequence	Ion Score	C.I.%	Modification
1036.5057	1036.5148	9	131	139	KEEGEAFAR			
1036.5057	1036.5148	9	131	139	KEEGEAFAR			
1045.5425	1045.5641	21	70	77	SITRSYYR			
1135.6357	1135.6366	1	9	19	YIIIGDTGVGK	51	99.999	
1135.6357	1135.6366	1	9	19	YIIIGDTGVGK			
1319.7317	1319.7283	-3	78	90	GAAGALLVYDITR	13	94.86	
1319.7317	1319.7283	-3	78	90	GAAGALLVYDITR			
1378.6671	1378.6672	0	140	151	EHGLIFMETSAK			Oxidation (M)
1378.6671	1378.6672	0	140	151	EHGLIFMETSAK			Oxidation (M)
1494.7434	1494.7462	2	152	165	TASNVEEAFINTAK	29	99.874	
1494.7434	1494.7462	2	152	165	TASNVEEAFINTAK			
1550.7598	1550.7543	-4	57	69	LQIWDTAGQESFR			
1760.8813	1760.8767	-3	171	186	IQEGVFDINNEANGIK			
2157.071	2157.0581	-6	152	170	TASNVEEAFINTAKEIYEK			

Spot 375 (20080606-A8) Rab2a								
**PMF/MALDI**								
Calculated	Observed	+/-ppm	start	end	Sequence	Ion Score	C.I.%	Modification
1134.6285	1135.6338	-2	9	19	K.YIIIGDTGVGK.S			
1380.7071	1381.6903	-17	20	30	K.SCLLLQFTDKR.F			
1549.7525	1550.7616	1	57	69	K.LQIWDTAGQESFR.S			
1044.5352	1045.567	23	70	77	R.SITRSYYR.G			
1318.7245	1319.7278	-3	78	90	R.GAAGALLVYDITR.R			
1717.806	1718.8014	-7	92	105	R.DTFNHLTTWLEDAR.Q			
1700.8338	1701.818	-14	106	120	R.QHSNSNMVIMLIGNK.S			Oxidation (M)
1391.7045	1392.7074	-3	128	139	R.EVKKEEGEAFAR.E			
1035.4985	1036.5099	4	131	139	K.KEEGEAFAR.E			
1377.6598	1378.6677	0	140	151	R.EHGLIFMETSAK.T			Oxidation (M)
1493.7362	1494.7491	4	152	165	K.TASNVEEAFINTAK.E			
2156.0637	2157.0598	-5	152	170	K.TASNVEEAFINTAKEIYEK.I			

Spot 375 (20080606-A8) Park7, Cap1								
**MS+MS/MS**								
Calculated	Observed	+/-ppm	start	end	Sequence	Ion Score	C.I.%	Modification
852.4937	852.4966	3	123	130	VTSHPLAK			
874.4992	874.5023	4	149	156	DGLILTSR			
1230.7052	1230.7036	-1	146	156	VEKDGLILTSR			
1230.7052	1230.7036	-1	146	156	VEKDGLILTSR	28	99.826	
1721.8085	1721.797	-7	13	27	GAEEMETVIPVDIMR			2 Oxidation (M)
1877.9095	1877.9126	2	13	28	GAEEMETVIPVDIMRR			2 Oxidation (M)

Spot 379 (D5 20080606) – mixture of: Platelet-activating factor acetylhydrolase 1B subunit beta (Pafah1b2) and Isoamyl acetate-hydrolyzing esterase 1 homolog (Iah1)

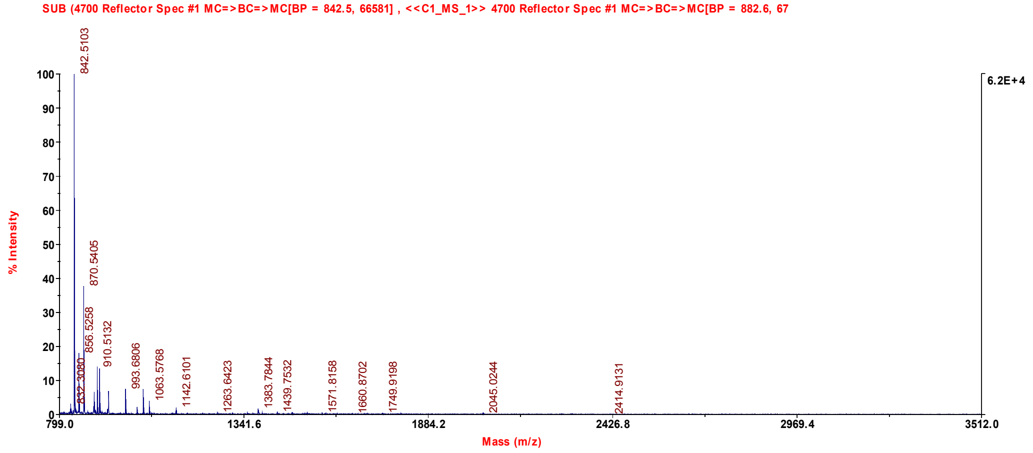

Spot 379 (20080606-D5) Pafah1b2								
MS+MS/MS								
Calculated	Observed	+/-ppm	start	end	Sequence	Ion Score	C.I.%	Modification
910.5104	910.5142	4	143	150	GEKPNPLR	29	99.824	
910.5104	910.5142	4	143	150	GEKPNPLR			
993.6818	993.682	0	134	142	IIVLGLLPR			
993.6818	993.682	0	134	142	IIVLGLLPR	26	99.656	
1027.6259	1027.6255	0	153	161	NAKVNQLLK	16	96.599	
1027.6259	1027.6255	0	153	161	NAKVNQLLK			
1141.6211	1141.6268	5	87	96	NGELENIKPK			
2045.045	2045.0251	-10	61	79	ELFSPLHALNFGIGGDTT			

Spot 379 (20080606-D5) Iah1								
MS+MS/MS								
Calculated	Observed	+/-ppm	start	end	Sequence	Ion Score	C.I.%	Modification
901.4162	901.4185	3	53	60	GFSGYNTR			
901.4162	901.4185	3	53	60	GFSGYNTR			
904.4669	904.4306	-40	46	52	KCDVLNR			Carbamidomethyl (C)
940.4556	940.4541	-2	113	119	DMVQYLR			Oxidation (M)
943.5206	943.5217	1	120	127	SVDIPKER	16	97.003	
943.5206	943.5217	1	120	127	SVDIPKER			
993.6931	993.682	-11	64	71	IILPRIIR			
993.6931	993.682	-11	64	71	IILPRIIR			
1063.5782	1063.5775	-1	155	164	LNVAVGEYAK			
1063.5782	1063.5775	-1	155	164	LNVAVGEYAK	21	98.992	
1794.8401	1794.8553	8	172	186	DCGTDVLDLWTLMQK			Carbamidomethyl (C)

Spot 381 (D24 20070719) – Pyruvate dehydrogenase E1 component subunit beta, mitochondrial precursor (Pdhb)

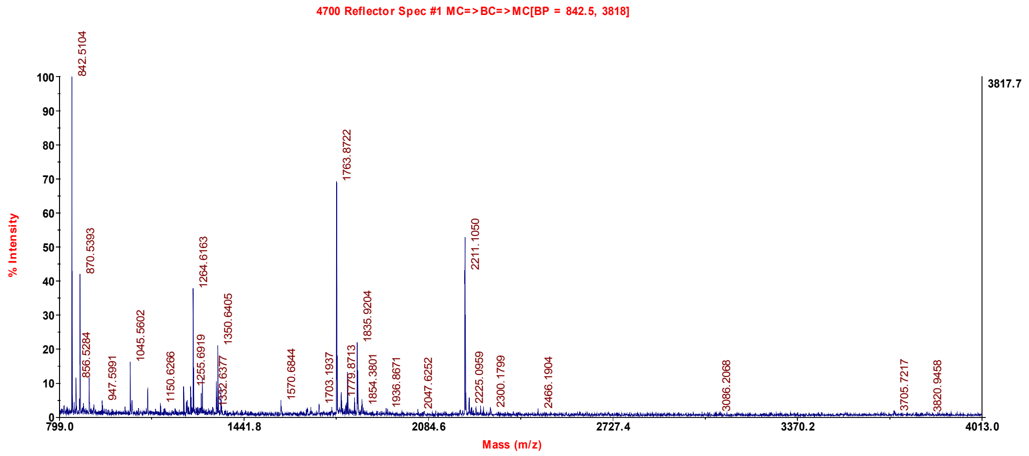

Spot 381 (20070719-D24) Pdhb								
MS+MS/MS								
Calculated	Observed	+/-ppm	start	end	Sequence	Ion Score	C.I.%	Modification
902.5345	902.5361	2	220	227	DFLIPIGK	23	99.283	
902.5345	902.5361	2	220	227	DFLIPIGK			
947.5923	947.5994	7	348	355	DIIFAIKK			
1231.5953	1231.598	2	174	184	VVSPWNSEDAK			
1255.6892	1255.697	6	337	347	ILEDNSIPQVK			
1255.6892	1255.697	6	337	347	ILEDNSIPQVK			
1264.6241	1264.6195	-4	325	336	VTGADVPMPYAK	18	97.254	Oxidation (M)
1264.6241	1264.6195	-4	325	336	VTGADVPMPYAK			
1763.8785	1763.8813	2	309	324	IMEGPAFNFLDAPAVR	42	99.991	Oxidation (M)
1763.8785	1763.8813	2	309	324	IMEGPAFNFLDAPAVR			
1801.9006	1801.9031	1	53	68	VFLLGEEVAQYDGAYK	17	96.744	
1801.9006	1801.9031	1	53	68	VFLLGEEVAQYDGAYK			
1835.9241	1835.9332	5	270	285	TIRPMDIEAIEASVMK			2 Oxidation (M)
1835.9241	1835.9332	5	270	285	TIRPMDIEAIEASVMK	11	86.675	2 Oxidation (M)

Spot 381 (20070719-D24) Pdhb								
PMF/MALDI								
Calculated	Observed	+/-ppm	start	end	Sequence	Ion Score	C.I.%	Modification
1800.8934	1801.8899	-6	53	68	K.VFLLGEEVAQYDGAYK.V			
1856.9495	1857.8979	-32	130	145	K.TYYMSAGLQPVPIVFR.G			Oxidation (M)
1230.5881	1231.5948	0	174	184	K.VVSPWNSEDAK.G			
901.5273	902.536	2	220	227	K.DFLIPIGK.A			
1331.6391	1332.6377	-7	259	269	K.EGIECEVINLR.T			
1834.9168	1835.9204	-2	270	285	R.TIRPMDIEAIEASVMK.T			2 Oxidation (M)
1746.8763	1747.8643	-11	309	324	R.IMEGPAFNFLDAPAVR.V			
1762.8712	1763.8721	-4	309	324	R.IMEGPAFNFLDAPAVR.V			Oxidation (M)
1263.6169	1264.6163	-6	325	336	R.VTGADVPMPYAK.I			Oxidation (M)
1254.6819	1255.6919	2	337	347	K.ILEDNSIPQVK.D			
